# DNA target recognition domains in the Type I restriction and modification systems of *Staphylococcus aureus*

**DOI:** 10.1093/nar/gkx067

**Published:** 2017-02-09

**Authors:** Laurie P. Cooper, Gareth A. Roberts, John H. White, Yvette A. Luyten, Edward K.M. Bower, Richard D. Morgan, Richard J. Roberts, Jodi A. Lindsay, David T.F. Dryden

**Affiliations:** 1 EaStCHEM School of Chemistry, University of Edinburgh, The King's Buildings, Edinburgh, EH9 3FJ, UK; 2 New England Biolabs, 240 County Road, Ipswich, MA 01938-2723, USA; 3 Institute of Infection and Immunity, St George's, University of London, Cranmer Terrace, London, SW17 0RE, UK; 4 Department of Biosciences, Durham University, Stockton Road, Durham, DH1 3LE, UK

## Abstract

*Staphylococcus aureus* displays a clonal population structure in which horizontal gene transfer between different lineages is extremely rare. This is due, in part, to the presence of a Type I DNA restriction–modification (RM) system given the generic name of *Sau1*, which maintains different patterns of methylation on specific target sequences on the genomes of different lineages. We have determined the target sequences recognized by the *Sau1* Type I RM systems present in a wide range of the most prevalent *S. aureus* lineages and assigned the sequences recognized to particular target recognition domains within the RM enzymes. We used a range of biochemical assays on purified enzymes and single molecule real-time sequencing on genomic DNA to determine these target sequences and their patterns of methylation. Knowledge of the main target sequences for *Sau1* will facilitate the synthesis of new vectors for transformation of the most prevalent lineages of this ‘untransformable’ bacterium.

## INTRODUCTION

Type I DNA restriction–modification (RM) systems are found in about half of the sequenced prokaryotic genomes (1–4). They present a formidable barrier to the invasion of the host cell by foreign DNA whether by transduction, transformation or conjugation and thus exercise control over horizontal gene transfer (HGT) (1,4–8). As an example of their effectiveness, less than 1 in 10^4^ or 10^5^ phage infections can successfully avoid the classical EcoKI Type I RM system of *Escherichia coli* K12. In some circumstances, such as when anti-restriction systems are absent (9), when there are multiple target sites on the phage (10) or when RM expression is raised (11), the barrier due to this single RM system can be even greater. RM systems operate by methylating defined target sequences on the host genome and they maintain this methylation pattern through each round of DNA replication (modification). Foreign DNA entering the cell often contains the same target sequence but in an unmethylated state. These unmethylated target sequences are targeted for endonucleolytic cleavage by the RM system (restriction). The Type I RM system comprises three *hsd* (host specificity for DNA) genes, *hsdR, hsdM* and *hsdS* for restriction, modification and target sequence specificity respectively. The gene products form an R_2_M_2_S_1_ complex in which HsdS (or S) recognizes the target sequence, HsdM (or M) recognizes the methylation status of the target and methylates hemimethylated targets while HsdR (or R) cleaves the DNA containing unmethylated targets after a complex reaction involving adenosine triphosphate (ATP) hydrolysis and DNA translocation (12). An M_2_S_1_ complex can act solely as a methyltransferase (MTase) (13). Type I RM enzymes almost always recognize and methylate adenine nucleotides in their target sequences to form N6-methyl adenine (6mA) although a few forming N4-methyl cytosine (m4C) are now known (3,14). In addition to the protection offered by Type I, II and III RM systems, Type IV restriction systems can attack foreign DNA containing methylated sequences not found in the host (15).

**Figure 1.**
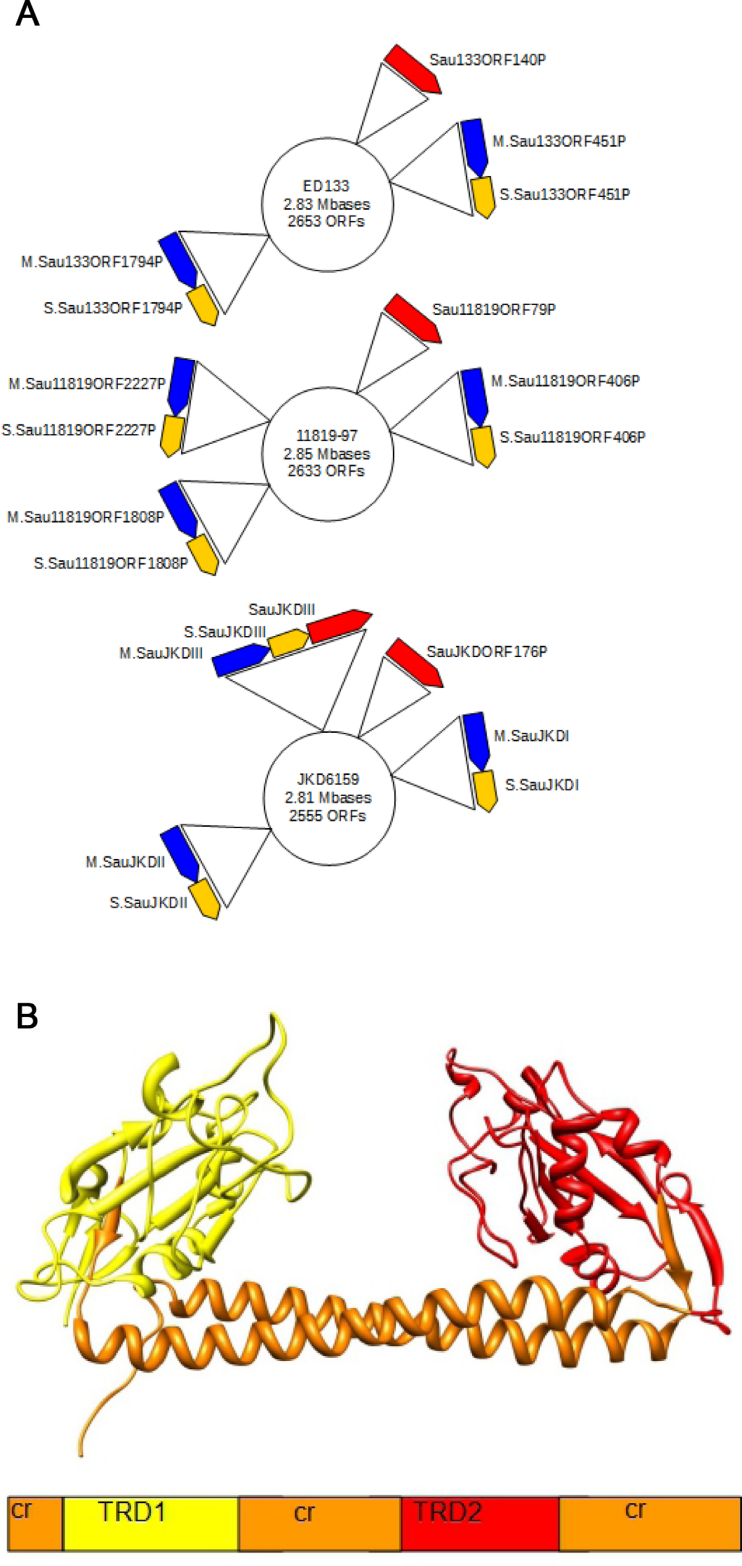
*Staphylococcus aureus* genomes showing the genes and the typical organization of target recognition domains (TRDs) in the HsdS DNA sequence specificity subunit. (**A**) Strain ED133 (CC133) has two *hsdS*; strain 11819–97 (CC80) has three *hsdS* (CC80) and strain JKD6159 (CC93) contains an extra Type I RM system from a different Type I RM family. From top to bottom: ED133, 11819–97, JKD6159. *hsdR* (red), *hsdM* (blue), *hsdS* (yellow). (**B**) The structural organization of the HsdS specificity subunit. The conserved regions (cr) are common to all S subunits within a family. The two TRDs (TRD1 and TRD2) define the target sequences recognized by the RM enzyme and can be swapped between S subunits of the same family to generate new specificities.

The presence of multiple RM systems in a single host can increase the barrier to HGT still further. For instance, *Staphylococcus aureus* often contains two related Type I RM systems making its transformation extremely inefficient and hindering the genetic analysis of this organism (16–19). These genomes contain two *hsdM* and two *hsdS* and share a single *hsdR*, although some *S. aureus* strains have different numbers of *hsdM* and *hsdS* (Figure 1A). The presence of only a single *hsdR* is not a problem as it can interact with each *hsdMhsdS* pair. It has long been known that *S. aureus* displays a clonal population structure (20) in which HGT between different clonal complexes is exceedingly rare. Multilocus sequence typing, microarray analysis and whole genome sequencing divides lineages of *S. aureus* and close relatives into the clonal complexes (CC) (20–23), each of which carries a different range of mobile genetic elements and antibiotic resistance genes on the genome (24–27). Each CC can be further subdivided into sequence types (ST) (22). Waldron and Lindsay (16) first realized that each CC of *S. aureus* contained a unique pair of Type I RM systems. A Type IV restriction system, SauUSI, was also identified later and recognized as a methyl-dependent restriction enzyme which would prevent the uptake of foreign DNA containing C5-methyl cytosine (5mC) (28,29). Thus most genetic manipulation of *S. aureus* is confined to strain RN4220, which has a defective Type I RM system due to a premature stop codon in *hsdR*. Furthermore, to avoid the Type IV system, DNA needs to be prepared from an *E. coli* strain, such as *E. coli* ER2796, lacking the Dcm 5mC MTase (30).

The Type I RM systems in different strains of *S. aureus* were given the informal name of *Sau1* by Waldron and Lindsay (16) and it is clear from not only a comparison of the sequences of genes and proteins but also from the ability to use subunits from one strain to complement subunits from other strains (31) that the term *Sau1* describes a classical ‘family’ of Type I RM systems. Type I RM families, Type IA to Type IE, were originally defined in *E. coli* and *Salmonella enterica* by DNA hybridization, antibody cross reactivity and subunit complementation (32,33), although now it is more usual to use the high levels of sequence identity (over 90%) in HsdM and HsdR to define a family *in silico*. Although the name *Sau1* for this family of Type I RM systems in *S. aureus* is an informal one not following the usual conventions (34), we retain it as it is established in the literature. However, it is important to note that some strains of *S. aureus* show additional Type I RM systems, which show limited amino acid sequence identity to the HsdR, HsdM and HsdS of *Sau1* (Figure 1A). For instance, Monk *et al*. (35) identified an active Type I RM system, SauJKDIII, in *S. aureus* JKD6159 which showed low sequence identity to members of the *Sau1* family. This is clearly a member of a new and different Type I RM family whose subunits will be unable to interact with the *Sau1* HsdM and HsdR (D. T. F. Dryden, J. A. Lindsay and M. T. G. Holden, in preparation).

The *Sau1* Type I RM systems are so effective because they show great variability in the target sequences recognized thus preventing HGT between CC but allowing HGT between strains within a CC (31,35,36). This variability in target sequences is due to the modular construction of the Type I RM systems (Figure 1B). The S subunit contains two target recognition domains (TRDs) each of which recognizes one half of a bipartite target, for example the first Type I RM system in CC1, given the generic name CC1-1, recognizes CCAYNNNNNTTAA (adenine methylation sites are underlined) (35,36). Swapping TRDs between S subunits generates new targets, for example the second Type I RM enzyme in CC1, termed CC1-2, couples the first TRD of CC1-1 with a different second TRD to recognize CCAYNNNNNNTGT. This swapping is easy because the DNA for S subunits contain conserved sequences bounding each TRD. Most *S. aureus* strains have two copies of *hsdS*, two of *hsdM* and one of *hsdR*. Thus, there are often four TRDs in each CC, which define the restriction barrier against HGT. Some Type I RM enzymes have half-size HsdS incorporating only a single TRD. It has been shown that these products are often able to dimerize and recognize symmetric target sequences (37–39). We have been able to recapitulate these results on ‘half-HsdS’ enzymes by manipulating the CC398-1 *S. aureus* system (E. K. M. Bower and D. T. F. Dryden, unpublished results).

Previously we have identified the target sequences recognized by several common community-associated, hospital-associated and livestock-associated MRSA clonal complexes (31,36) and recently several more have been identified (3,35,40). Monk *et al*. (35) and Jones *et al*. (40) have used this information to prepare DNA methylated by the MTase M_2_S_1_ component enzymes to aid the transformation of *S. aureus* strains that are usually resistant to transformation.

The identification of further targets recognized by the S subunits of *Sau1* Type I RM systems would in principle allow more CC to be transformed for genetic analysis. In addition, further understanding of the structural requirements for TRDs to recognize different specific DNA sequences is of intense interest as the Type I RM systems are very widespread in bacteria and archaea (1,4) and exert a considerable pressure on HGT and the evolution of prokaryotes. For instance, the use of multiple TRDs being exchanged between strains has been observed in *Helicobacter* (41), *Mycoplasma* (42,43), *Streptococci* (44,45) and *Bacteroides* (46).

Here we identify many further TRDs and their targets using both biochemical and PacBio single-molecule real-time (SMRT) sequencing methods to define the barriers to HGT in a wide range of *S. aureus* CC of global importance.

## MATERIALS AND METHODS

### Nomenclature for expression plasmids encoding new MTases

As each Type I S subunit contains two TRDs and we propose to determine the targets recognized by each TRD, we have given each TRD a single letter code, Table 1, and refer to the plasmids as pSauTRD1-TRD2, e.g. pSauBI expresses an S subunit containing TRD B and TRD I and the M subunit. If the TRD combination is the same as that found in a known clonal complex, then that CC is also given in brackets. The MTase would be called M.SauBI in this example and the S subunit S.SauBI and is from CC22. All sequences are given in the Supplementary Data.

**Table 1.** TRD targets shown from 5΄ to 3΄

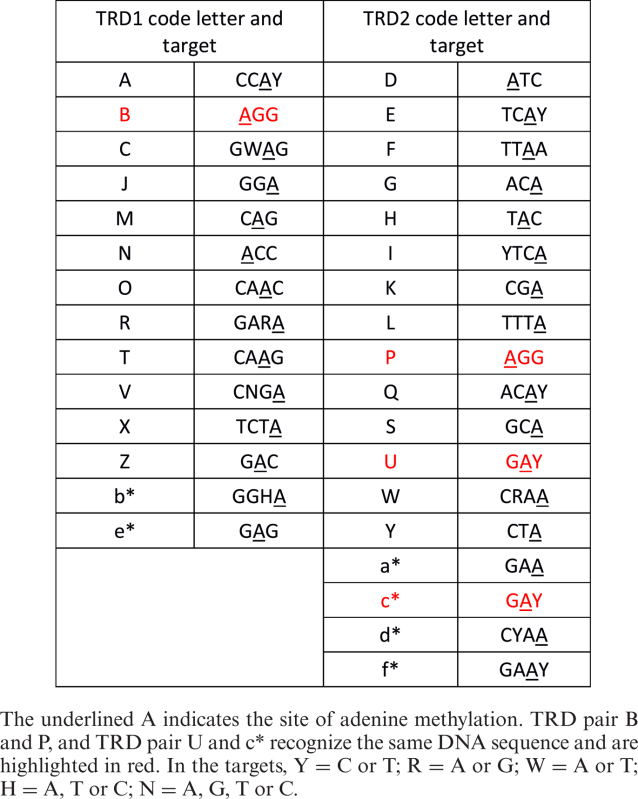

### Preparation of M.SauBI (CC22-1), M.SauCD (CC30-1), M.SauJK (CC30-2) and M.SauCL (CC45-1)

These four MTases were prepared as EGFP-His tag fusions as described in Roberts *et al*. (31). pSauBI-EGFP (CC22-1, genomic DNA from MRSA5906), pSauCD-EGFP (CC30-1, genomic DNA from MRSA252), pSauJK-EGFP (CC30-2, genomic DNA from MRSA252) and pSauCL-EGFP (CC45-1, genomic DNA from strain 70642) were all constructed by the polymerase chain reaction (PCR) with their *hsdS* fused to DNA encoding EGFP and a His-tag, with the following locus-specific oligonucleotides priming from the 3΄ end of the genes encoding the S subunits:

CC22-1 BI BS 5΄GATCGAATTCCGGATCCAATAAACATCTTTTGTAAAAACAC3΄

CC30-1 CD BS 5΄GATCGAATTCCGGATCCTAAGAACATCTTTTGTAAAAAGG3΄

CC30-2 JK BS 5΄GATCGAATTCCGGATCCTATAAAAATTTTTTGAAGTAATCCTTG3΄

CC45-1 CL R167K BS 5΄GATCGAATTCCGGATCCAATAAACATCGATTTAAGTAAGGC3΄

The sequence for CC45-1 introduced a single mutation R167K in the first TRD in the S subunit but since this change is found in other *S. aureus* isolates containing this TRD, the change is presumed to be completely neutral.

### A new vector for MTase expression: pJF118his

Although we had not experienced problems in examining the fusion proteins of S subunits and EGFP in biochemical work, we decided to construct a vector encoding *hsdS* with only a C-terminal His-tag. Vector pJF118his was made by PCR of the plasmid encoding the MTase CC5-1-EGFP constructed in Roberts *et al*. (31) with these two oligonucleotides:

pJFMShisTS 5΄AGCTTCGAGAGGATCCCATCATCATCATCATCATTAAGAATTCAGCTTGGCTGTTTTGGCGG3΄ and pJFMSEGFPhisBS 5΄GAGTGAATCCCCGGGGATCCGTCGACC3΄.

The resulting PCR product was cut with BamHI and unimolecular religation gave pJF118his into which the *hsdMS* operon could be ligated as BamHI fragments and from which all subsequent MTase clones were descended.

### Construction of an MTase plasmid to allow TRD swaps: pSaudeltaXmaI

A PCR-based strategy was devised to allow free pairwise assortment of desired TRDs in HsdS. Many, but not all of the HsdS subunits, including that encoded by the Type I system in CC398 (36), have a predicted proline-glycine sequence near the N-terminus. This dipeptide can be encoded by CCCGGG, which would be a target site for SmaI or XmaI. Oligonucleotides were designed which would introduce this motif in the N-terminus (a replacement with no amino acid changes) and at the C terminus (an insertion of two amino acids) of the S subunit of the CC398 system (36), by a two stage PCR fusion. Thus, primary PCR products were generated by reactions primed by: PromoterJF 5΄GCTTCTGGCGTCAGGCAGCC3΄ with 398SmaIOligoBS 5΄CCCATTCGCCTTCAAACCCGGGGAATCTCAACTCTGGCAC3΄ and 398SmaIOligoTS 5΄GTGCCAGAGTTGAGATTCCCCGGGTTTGAAGGCGAATGGG3΄ with 398SmaIBamHI 5΄GATCGATCGGATCCCCCGGGAATAAACATCTTTTGAAGTAATGAC3΄.

The purified PCR products were then fused in a secondary PCR reaction primed by PromoterJF with 398SmaIBamHI. The product was then cut with BamHI, and ligated into the BamHI site of pJF118his as pSauNE-XmaI. This mutated form of the CC398-1 MTase, could assemble the complete restriction enzyme that proved to be active in endonucleolytic cleavage (36). This indicated that insertion of a proline and glycine toward the C-terminus did not affect the function of the enzyme. Subsequently, on reanalyzing the DNA sequence, a single PCR mutation was discovered within the XmaI fragment. This caused a mutation A50S but this clearly did not affect the specificity or function of the S subunit in our assays. Digestion of pSauNE-XmaI with XmaI followed by intramolecular religation of the vector fragment generates pSaudeltaXmaI, into which any pairwise combination of TRDS with XmaI cohesive ends may be inserted.

### Construction of MTases M.SauNI, M.SauND, M.SauNK, M.SauNL, M.SauBE, M.SauJE and M.SauCE (ST425-1) containing hybrid S subunits

The DNA for each TRD of these S subunits was fused to the DNA for the reciprocal TRD of S.SauNE (CC398-1). This was achieved by creating primary PCRs with a short area of homology, which then allowed base pairing of single strands of each PCR, in a secondary PCR. For example, S.SauBE TRD B was generated from an appropriate plasmid template by PCR with oligonucleotides, TRD1FOR398SmaIOligoTS 5΄GTGCCAGAGTTGAGATTCCCCGGGTTTGAAGGCGAATGGG3΄ paired with TRD1nearuniversal 5΄GTTCTTCTAATTCAATTTGT3΄. TRD E was similarly generated by PCR from plasmid template with oligonucleotides TRD2nearuniversal 5΄ACAAATTGAATTAGAAGAAC3΄ and 398SmaIBamHI 5΄GATCGATCGGATCCCCCGGGAATAAACATCTTTTGAAGTAATGAC3΄. The final insert was then generated by PCR with the two gel-purified primary oligonucleotides and TRD1FOR398SmaIOligoTS 5΄GTGCCAGAGTTGAGATTCCCCGGGTTTGAAGGCGAATGGG3΄ and 398SmaIBamHI 5΄GATCGATCGGATCCCCCGGGAATAAACATCTTTTGAAGTAATGAC3΄. S.SauCL was the only subunit for which we could not use the central universal oligonucleotides for PCR and required specific substitutes: TRDLFOR/CC45-1 5΄ACAAATTGAATTAGAAGAACAAAAACTTGAATTACTTCAACAACAG3΄ and TRDC/CC45-1 5΄GTTCTTCTAATTCAATTTGTCGATCGAGTTTGCTGAAGAAG3΄. Each C-terminus is unique and where TRD2 was not TRD E, a specific oligonucleotide was employed: TRDIREV/CC22-1c-termsmaI 5΄GATCGATCGGATCCCCCGGGAATAAACATCTTTTGTAAAAACAC3΄, TRDDREV/CC30-1c-termsmaI 5΄GATCGATCGGATCCCCCGGGTAAGAACATCTTTTGTAAAAAGGATTG3΄, TRDKREV/CC30-2c-termsmaI 5΄GATCGATCGGATCCCCCGGGTATAAAAATTTTTTGAAGTAATCCTTG3΄ and TRDLREV/CC45-1c-termsmaI 5΄GATCGATCGGATCCCCCGGGAATAAACATCGATTTAAGTAAGGC3΄. Each pure secondary PCR product was cut with XmaI and ligated into the XmaI site of pSaudeltaXmaI.

### Construction of further MTases with further combinations of TRDs using synthetic genes

Additional *hsdS* sequences were obtained as synthetic genes from GeneArt (ThermoFisher Scientific) with sequences optimized for expression in *E. coli* (Supplementary Data). All the first TRDs begin with 5΄CCCGGGTTTGAAGGCGAATGGGAG3΄, except that for CC80-2 which begins with 5΄CCCGGGTTTGAAGGCGAATATTCT3΄. All the first TRDs end with 5΄CAAATTGAATTAGAAGAACAGAAG3΄. All the second TRDs begin with 3΄CAAATTGAATTAGAAGAACAGAAG5΄ and have a universal reverse oligonucleotide, Trd2unirev 5΄GATCGATCGGATCCCCCGGG3΄. These conserved sequences were used to create oligonucleotides to prime PCR reactions. Each pure secondary PCR product was cut with XmaI and ligated into the XmaI site of pSaudeltaXmaI. The orientation of the fragments was determined by PCR.

### Expression and purification of MTases

These new MTases and the R subunit of CC5 were expressed in *E. coli* BL21(DE3) and purified via HisTrap chromatography, size exclusion chromatography, diethylaminoethyl anion exchange chromatography and, if necessary, Heparin HiTrap chromatography (GE Healthcare, Uppsala, Sweden) as described previously (31).

### Nuclease and ATPase assays

Purified MTases were mixed with the CC5 R subunit and used in assays for ATP hydrolysis (ATPase) activity (coupled enzyme assay following a change in absorbance of NADH) and DNA cleavage activity (plasmid cutting assay with analysis via agarose gel electrophoresis) as previously described (31,36).

### Preparation of genomic DNA for SMRT sequencing

The expression plasmids harboring the various MTases were used to transform a non-methylating (dam^−^ dcm^−^) strain of *E. coli* ER2796 (30). Single colonies from the transformation plate of Lysogeny Broth (LB) agar medium supplemented with 10 μg/ml kanamycin, 10 μg/ml tetracycline as well as 100 μg/ml carbenicillin, which acted as a selection marker for the expression construct, were picked and used to inoculate 5 ml of LB containing the same cocktail of antibiotics. The cultures were incubated overnight with shaking at 37°C and 1 ml aliquots of the overnight culture were then pelleted by centrifugation (6000 *g*, 6 min, 4°C). The culture medium was carefully removed and the cell pellets stored at −20°C until required. Genomic DNA was prepared from each cell pellet using the Wizard Genomic DNA purification kit (Promega, Madison, WI, USA) according to the manufacturer's instructions. The quality of the genomic DNA preparations was initially assessed by agarose gel electrophoresis and from the shape of the absorbance profile from 240 to 340 nm. Genomic DNA from *S. aureus* strains LGA251 (a kind gift from Mark Holmes) and NCTC13435 (a kind gift from Angela Kearns) was prepared by using the PurElut Bacterial Genomic Kit (EdgeBio, Gaithersburg, MD 20877, USA). The DNA library for SMRT sequencing was prepared and subsequently analyzed as described in Anton *et al.* (30).

### Methylation of plasmids using M.EcoGII

M.EcoGII was kindly supplied by Dr Iain Murray (New England Biolabs) and used to modify plasmids E2, E5, E10, E11 and E12 previously described (31) and plasmid pCN36 (47). A total of 0.45 μg DNA was methylated using 2.0 U of M.EcoGII for 100 min at 37°C in a 50 μl volume. The reaction was in 1×NEB4 buffer (50 mM potassium acetate, 20 mM Tris acetate, 10 mM Mg acetate, 1 mM dithiothreitol, pH 7.9, 25°C) supplemented with 320 μM S-adenosyl-L-methionine (SAM). As a negative control, DNA was incubated in the same buffer without M.EcoGII. The DNA samples were then supplemented with ATP (20 μM) and additional SAM (160 μM) and then digested with a Type I enzyme (CC5-1, CC5-2, CC30-1, CC45-1 or the NY TRD hybrid) for 14 min at 37°C. As a control, methylated and unmethylated DNA was digested with EcoRI.

## RESULTS AND DISCUSSION

### Assigning TRDs to target sequences

**Figure 2.**
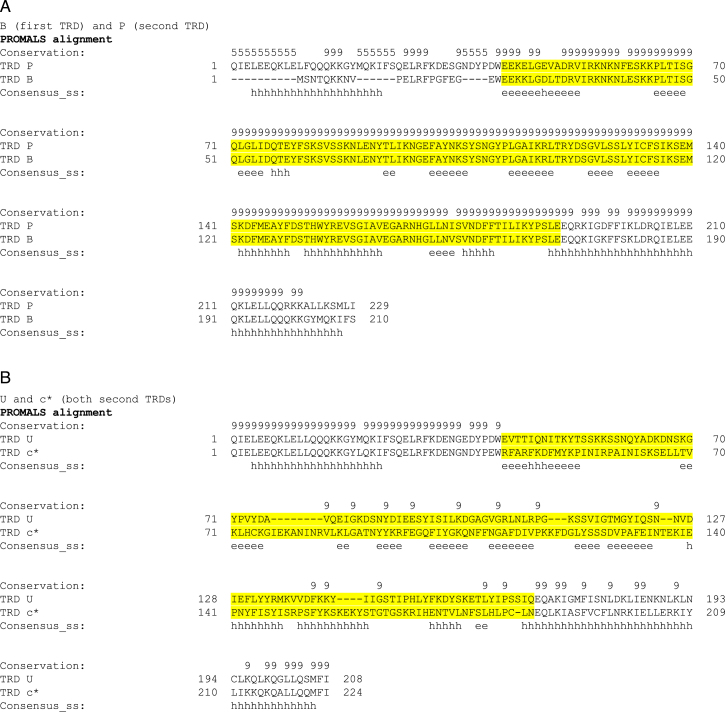
Amino acid sequence and secondary structure alignment of two pairs of TRDs recognizing the same DNA target. The TRD sequences are highlighted in yellow. Consensus secondary structure shows ‘h’ for α helix and ‘e’ for β sheet. (**A**) TRDs B and P are examples of a first and a second TRD respectively recognizing 5΄-AGG-3΄. (**B**) TRDs U and c* are both examples of second TRDs with the same specificity, 5΄-GAY-3΄. The long predicted α helices at the start and the end of the sequences are the conserved helical spacer regions in the HsdS subunits while the sequence between these helices makes up the TRD.

Each TRD was given a one letter code (A to Z and a* to f*), Table 1. There were 14 TRD1 examples and 18 TRD2 examples in our survey and these are found in 17 different CC or ST groups. Table 1 lists the target specificity and site of methylation for each TRD in our survey. These data were obtained by pairing TRDs and determining the complete target for each TRD pair as described in the next section and in full in the Supplementary Data. Of interest are the TRD pairs B and P and U and c*. These pairs recognize the same DNA sequence namely AGG and GAY respectively. Amino acid sequence comparisons of B with P and U with c* are shown in Figure 2.

TRD B and TRD P are virtually identical throughout the TRD region even though TRD B is the first TRD in the HsdS subunit and TRD P is the second TRD in the HsdS subunit, (Figure 2A). While the high level of sequence identity is expected for Type I systems in the same family, the high level of identity between TRDs found in the first or second position in the HsdS subunit is more unusual. However, such a situation has previously been observed in comparisons of the Type I systems in *Salmonella blegdam* and *E. coli* R124 (48).

In contrast, TRDs U and c* are both examples of the second TRD in the HsdS subunit recognizing 5΄-GAY-3΄ but the level of identity between them is much lower (∼36%) (Figure 2B). This level of identity between TRDs recognizing the same target is expected if the TRDs are from different Type I RM families so the low level of identity observed here is unusual. Despite this low level of sequence identity, the predicted secondary structure elements are the same as expected from the early work of Sturrock and Dryden (49). In fact, all of the TRDs in the *Sau1* family of RM systems align well when secondary structure elements are taken into consideration (50) and they will have the same protein fold (Supplementary Data: PROMALS alignments). Therefore, it should in future be possible to predict the precise amino acid to nucleotide contacts involved in sequence recognition as was done for the Type IIG TRDs (51,52).

### Determination of complete target sequences recognized by pairs of TRDs

Tables 2, 3 and 4 show the TRD combinations investigated in this work and those investigated previously by ourselves and others along with their combined target sequences, methylation specificity and the methods used to determine these parameters. The full experimental data are given in the Supplementary Data. Many of the TRDs were investigated in more than one MTase and in more than one assay thus our set of data represents a self-consistent set. DNA cleavage and ATP hydrolysis assays were performed on purified MTases mixed with purified R subunit while SMRT data were collected from *E. coli* genomic DNA isolated after the hosts were transformed with a plasmid expressing the MTase or directly from *S. aureus* genomic DNA. The adenines targeted for methylation were determined easily by SMRT sequencing but for systems not examined in this manner, it was assumed if there was a single adenine in the site recognized by the TRD that this was the target for methylation.

**Table 2.** The *Sau1* RM systems with published recognition sequences

Strain name and genome reference	Clonal Complex or Sequence Type	S subunit name in REBASE	Recognition sequence	TRDs assigned	Suggested generic name	Experimental method	Reference for target specificity and method
MW2 (53)	CC1	S.SauMW2I	CCAY-5-TTAA	AF	CC1-1	g, s, a	g (31)
		S.SauMW2II	CCAY-6-TGT	AG	CC1-2 (CC8-2)	g, s, a	a (36)
N315 (54)	CC5	S.SauN315II	CCAY-6-GTA	AH	CC5-2	g, s, a	s (CC8-1 and CC8-2 in strain NRS384 are from ref. 35)
		S.SauN315I	AGG-5-GAT	BD	CC5-1 (CC8-1)	g, s, a	
MRSA252 (55)	CC30	S.SauMRSII	GWAG-5-GAT	CD	CC30-1	g, s	s (35) g, s (this work)
		S.SauMRSI	GGA-7-TCG	JK	CC30-2	s	s (35) s (this work)
JKD6159 (56)	CC93	S.SauJKDIII	GAAG-5-TAC or complement	Not a *Sau1* system	CC93-3	s	s (35)
		S.SauJKDII	GGHA-7-TCG	b*K	CC93-2	s	Note the ambiguity in assigning CC93-1 and CC93-3 is clarified with strains ED133 and 32320 and from Table 3.
		S.SauJKDI	CAG-6-TTC	Ma*	CC93-1	s	
ED133 (57)	CC133	S.Sau133ORF451P	CAG-5-RTGA	ME	CC133-1	g	g (36)
		S.Sau133ORF1794P	GGA-7-TTRG	Jd*	CC133-2	s	s (this work)
32320 (58)	CC133	S.Sau32320ORFAP	CAG-5-RTGA	ME	CC133-1	g	g (36)
S0385 (59)	CC398	S.SauSTORF499P	ACC-5-RTGA	NE	CC398-1	g, s	g (36) s (this work)

Target sites are shown from 5΄ to 3΄ with the length of the non-specific spacer shown as a number. Underlined A or T indicates the site of adenine methylation on the top or bottom strands respectively. The experimental methods used are indicated as g = target obtained by DNA cleavage with a purified enzyme, s = target obtained by SMRT sequencing of E. coli ER2796 genomic DNA, a = target obtained by ATPase assay with a purified enzyme. Full details are given in the Supplementary Data. S.Sau133ORF1794P is characterized in this work but is included here as it is part of the RM system found in strain ED133. SauMRSI and SauMRSII characterized by Monk *et al.* and S.SauSTORF499P characterized by Chen *et al.* are also further characterized in this work.

**Table 3.** The ‘artificial’ *Sau1* systems containing novel pairings of TRDs

‘Artificial’ *Sau1* RM systems.					
Recognition sequence	TRDs assigned	Experimental method	Recognition sequence	TRDs assigned	Experimental method
AGG-5-RTGA	BE	a	ACC-6-TTC	Na*	s
GGA-6-RTGA	JE	g, s	ACC-6-RTC	Nc*	s
ACC-6-TGAR	NI	g	ACC-6-TTRG	Nd*	g, s
ACC-6-TCG	NK	g	GARA-6-RTGA	RE	s
ACC-6-TAAA	NL	g	CAAG-5-RTGA	TE	s
ACC-5-CCT	NP	s	CNGA-6-RTGA	VE	s
ACC-5-RTGT	NQ	g, s	TCTA-6-RTGA	XE	g, s
ACC-6-TGC	NS	s	GAC-5-RTGA	ZE	a
ACC-5-RTC	NU	g, s	GAC-6-TGC	ZS	a
ACC-6-TTYG	NW	g, s	GGHA-6-RTGA	b*E	s
ACC-6-TAG	NY	g, s	GAG-6-RTGA	e*E	g, s

Target sites are shown from 5΄ to 3΄ with the length of the non-specific spacer shown as a number. Underlined A or T indicates the site of adenine methylation on the top or bottom strands respectively. The experimental methods used are indicated as g = target obtained by DNA cleavage with a purified enzyme, s = target obtained by SMRT sequencing of *E. coli* ER2796 genomic DNA, a = target obtained by ATPase assay with a purified enzyme. Full details are given in the Supplementary Data.

**Table 4.** The *Sau1* RM systems investigated in this project

Strain name and genome reference	Clonal Complex or Sequence Type	S subunit name in REBASE	Recognition sequence	TRDs assigned	Suggested generic name	Experimental method
CO1791 (58)	CC97	S.SauC01791ORFAP	CCAY-6-RTC	Ac*	CC97-1	s
HO5096 (60)	CC22	S.Sau5096I	AGG-6-TGAR	BI	CC22-1	g, s
LGA251 (61)	ST425	S.Sau251I	GWAG-5-RTGA	CE	ST425-1	g, s*
		S.Sau251ORF16900P	GAG-?-RTTC	e*f*	ST425-2	Not expressed, no signature with s*.
		S.Sau251II	GAAG-5-TAC or complement	Not a *Sau1* system	Same as CC93-3	s*
Isolate 3 (19)	CC51	S.SauL3ORFAP	GGA-6-CCT	JP	CC51-1	s
Isolate 3067 (19)	CC45	S.Sau347I	GWAG-6-TAAA	CL	CC45-1	g
Isolate 3150 (19)	CC15	S.SauL315ORFAP	CAAC-5-RTGA	OE	CC15-1	s
SA40 (62)	CC59	S.SauSA40ORF370P	GGA-6-RTGT	JQ	CC59-1	a
CN1 (63)	CC72	S.SauCN1ORF415P	GARA-6-RTGT	RQ	CC72-1	a
		S.SauCN1ORF1757P	GGA-7-TGC	JS	CC72-2	a
MSHR1132 (64)	CC75	S.Sau1132ORF3780P	CAAG-5-RTC	TU	CC75-1	g
		S.Sau1132ORF16570P	CNGA-7-TTYG	VW	CC75-2	s
NCTC13435 NCBI Biosample identifier:	ST80	S.Sau13435ORF394P	TCTA-?-TAG	XY	ST80-1	Not expressed, no signature with s or s*.
SAMEA2479566		S.Sau13435ORF1751P	GAC-6-TTYG	ZW	ST80-2	a, s*
		S.Sau13435ORF2165P	TCTA-6-RTTC	Xf*	ST80-3	s, s*
32326 (58)	CC873	S.Sau32326ORFAP	GAG-6-GAT	e*D	CC873-1	a

Target sites are shown from 5΄ to 3΄ with the length of the non-specific spacer shown as a number. Underlined A or T indicates the site of adenine methylation on the top or bottom strands respectively. TRD pair e*f* in strain LGA251 was not cloned in *E. coli* while TRD pair XY was cloned. However, no target modification was observed using SMRT on genomic DNA from either *E. coli* or *S. aureus* for these TRD pairs. If the genes are translated, their target is inferred from other TRDs in this table although the spacer length remains undefined. The experimental methods used are indicated as g = target obtained by DNA cleavage with a purified enzyme, s = target obtained by SMRT sequencing of *E. coli* ER2796 genomic DNA, s* = target obtained by SMRT sequencing of *S. aureus* genomic DNA, a = target obtained by ATPase assay with a purified enzyme. Full details are given in the Supplementary Data.

Table 2 contains systems from a range of CC investigated previously as well as several examined in this study. It is important to note that in our work those systems containing M.SauMRSII plus S. SauMRSII, M.Sau133ORF1794P plus S.Sau133ORF1794P and M.SauMRSI plus S.SauMRSI are paired with the HsdR (SauN315ORF189P) from the N315 strain of CC5 in DNA cleavage and ATPase assays. Those shown in Tables 3 and 4 are studied as HsdS paired with the HsdM (M.SauSTORF499P) from strain S0385 of CC398 and the HsdR (SauN315ORF189P) from the N315 strain of CC5 (if used in DNA cleavage or ATPase assays). Therefore, these HsdS are not examined in the context of their natural genome, but since they are all from the *Sau1* family of Type I RM systems and the HsdM and HsdR of these RM systems are essentially identical in all of the strains, it is reasonable to assume that the target specificities identified are those that would be recognized in their natural host.

Identifying the complete target recognized by a member of the *Sau1* Type I RM family when both TRDs have unknown targets is difficult and ambiguous as either orientation may be correct. Hence, we combined TRDs with unknown targets with TRD E or TRD N to make a protein recognizing a hybrid sequence in which one half of the target was already known (Table 3). A variety of methods were used to determine the target associated with each hybrid including DNA cleavage and ATP hydrolysis assays when the hybrid enzyme could be expressed and purified from *E. coli* and SMRT sequencing when the expression and purification levels were low, for example, the SauJK enzyme corresponding to the second Type I RM enzyme in CC30 did not express in *E. coli* despite its expression in *S. aureus* by Monk *et al*. (35). The ambiguity in assignment of targets in CC93 in Monk *et al*. (35) is resolved because the TRDs M and b* occur in more than one HsdS in our survey.

The DNA sequences for further pairs of TRDs found in a wide range of CC and ST groups were then inserted after the *hsdM* of CC398-1 in our expression vector and examined to ascertain the spacer sequence in the natural system (Table 4).

Genomic DNA from *S. aureus* strains NCTC13435 and LGA251 was prepared and examined using SMRT sequencing as these strains contain two TRD pairs, XY and e*f* respectively, which we could not express in *E. coli*. While SMRT signatures for the other Type I HsdS in these strains were very clear (Supplementary Data) and in agreement with our results from *E. coli* (Table 4) and those of Monk *et al*. (35), these TRD pairs still showed no methylation activity even in their normal host. Thus, these TRDs pairs are not active.

### Analysis of spacer sequence length in *S. aureus* Type I RM systems

It is apparent that the number of base pairs separating the adenines targeted for methylation and the number of base pairs in the non-specific spacer between the sequences recognized by the TRDs is not constant, with the former varying between 7 and 9 bp and the latter varying between 5 and 7 bp. This variation makes it very difficult to predict a Type I RM recognition sequence if one knows only the targets recognized by the two TRDs as the length of the spacer in the target is not recognized in any obvious manner by the TRDs. An example of this is the CC80-1 enzyme (Table 4) containing TRDs X and Y of known specificity. Since the enzyme did not methylate DNA *in vivo* for the SMRT analysis, the spacer and hence the complete target for CC80-1 remain unknown until the enzyme is purified and analyzed biochemically. While it has been observed that insertions of multiples of four amino acids into the alpha helical spacers separating the TRDs can increase the length of the spacer in the target sequence in a predictable manner (65–67), it is clear from the structure of HsdS subunits (Figure 1B) that the junction between the TRDs and the alpha helical spacers in the conserved region is going to be of crucial importance for determining the fine details of the length of the spacer in the target sequence as was found for some Type IIB RM enzymes which contain a subunit equivalent to HsdS (68). Perhaps even single amino acid insertions or deletions will serve to rotate the TRD with respect to the rest of the subunit and thereby change the length of the spacer. Further progress in understanding the correlation between amino acid sequence and the length of the target spacer would be greatly aided by an accurate atomic structure of a Type I enzyme with DNA as the current models (12,13) lack sufficient resolution to be informative on this point.

### Linking TRDs pairs to further clonal complexes and sequence types

After determining the recognition sequences for all of the TRDs in Table 1 by creating artificial hybrids (Table 3) we also found that some of these TRD combinations do actually occur in natural systems as given in Table 5 (and Supplementary Data) (69). As sequence databases expand, more and more of the possible TRD combinations based on the TRDs in Table 1 will be found. As mentioned above, although the sequences recognized by the TRDs are known, the length of the non-specific spacer separating them is unknown so that the complete target cannot be specified accurately without experimentation.

**Table 5.** Further TRD pairs found in sequenced strains of *Staphylococcus aureus*

TRD pair	Example strain	Clonal complex or sequence type of example strain	REBASE name
AD	FDAARGOS_159	ST5	S.Sau159ORF12345P
AL	K12S0375	ST692	S.Sau375ORFDP
AU	*Staphylococcus schweitzeri* FSA084		S.SauFSA084ORF355P
AW	FDA209P	ST464	S.Sau209ORF1697P
BG	MRSN8611	ST8	S.Sau8611ORF11430P
BH	PLAC6019	ST5	S.Sau6019ORF851P
BU	SA-083	ST101	S.Sau083ORF9680P
BY	*Staphylococcus argenteus* M260-MSHR		S.SarM260ORF2316P
Bf*	SA-083	ST101	S.Sau083ORF1720P
JE	Tager 104	ST49	S.Sau104ORF1102P
JL	W56227	ST45	S.Sau56227ORF970P
JW	CIG290	ST45	S.SauCIG290ORF2408P
JW	APS211	ST45	S.SauAPS211ORF9230P
MW	FSA037	ST1872	S.SauFSA037ORF2487P
NQ	KPL1845	ST96	S.Sau1845ORF2596P
Of*	USA300-TCH959	ST1159	S.SauTCH959ORF2844P
Rf*	Tager 104	ST49	S.Sau104ORF2433P
TY	M21126	ST2250	S.Sau21126ORF1065P
XF	21334	ST109, CC9	S.Sau21334ORF1353P
XF	RKI4	ST27	S.SauRKI4ORF1905P
XW	103564	ST80-PVL carrier	S.Sau103564ORF678P
ZY	D139	ST145	S.SauD139ORF2470P
b*W	ST20130941	CC15	S.Sau941ORF4310P
e*f*	SA-120	ST425	S.Sau120ORF4875P

Every pair of TRD1 with TRD2 in Table 1 was used in a BLASTP sequence search to identify HsdS subunit sequences in publicly accessible databases. Examples of strains containing these TRD pairs are shown. ST and CC are from the PATRIC database (69) or derived using *www.cbs.dtu.dk/services/MLST* (73). Some TRD pairs are present in many strains while others are rare.

### Further TRDs in *S. aureus* Type I RM systems

Searching the publicly available sequences in the NCBI database with individual TRD sequences revealed that some of those given in Table 1 can be found paired up with further novel TRDs. We have found four new TRDs shown in Table 6 in *S. aureus* strains 21343 and KPL1845. Strain 21343 contains ‘NOVEL 1’ paired with TRD K and the TRD pair NQ described in Table 3. Strain KPL1845 also contains the TRD pair NQ and two further systems comprised of ‘NOVEL 2’ paired with ‘NOVEL 3΄ and ‘NOVEL 4’ paired with TRD f*. Undoubtedly further TRDs will be found as sequencing continues.

**Table 6.** New TRD pairs associated with pairs shown in Tables 2, 3 and 4

Subspecies 21343 Bioproject accession: PRJNA53699
> S.Sau21343ORF2597P TRD NOVEL 1 + TRD K
MSNTQKKNVPELRFPGFEGEWEEKKLGEVATFAKGKLGAKKDVSQNGVPVILYGELYTKYGAIVSKIFSKTDIPENKLKMAKKNDVLIPSSGETAIDIATASCIYLNKGVAVGGDINILTPQKQDGRFISLSING INKNELSKYAQGKTVVHLYNNDIKNLKIAFPSEFEEQVRIGNFFSKLDRQIELEEQKLELLQQQKKGYMQKIFSQELRFKDENGNDYPKWEEKKIEDIASQVYGGGTPNTKIKEFWNGDIPWIQSSDVKVNDLIL QQCNKFISKNSIELSSAKLIPANSIAIVTRVGVGKLCLVEFDYATSQDFLSLSSLKYDKLYSLYSLLYTMKKISANLQGTSIKGITKKELLDSIIKIPHNLEEQQKIGDLFYKIDKYISFNKCKIEILKSLKQGLLKKMFI

Species KPL1845. Bioproject accession: PRJNA169473
> S.Sau1845ORF1619P TRD NOVEL 2 + NOVEL 3
MTEQINTPELRFPEFKNEWSYDLVSDVVTNKSKKFDPKKEEAKKDIELDSIEQNTGRLLDTYISNDFTSQKNKFNKGNVLYSKLRPYLNKYYYATIDGVCSSEIWVLNTLNKDVLANKFLYYFIQTNRFSSVTN KSAGSKMPRADWELVKNIRLYKGSIEEQEKIGYFFSKLDRQIELEEKKLELLEQQKKGYMQKIFAQELRFKDENGNDYPDWVTKKLGDIGKVAMNKRIYKNETTENGEIPFYKIGNFGKNADTFITREKFDEYK EKYPYPNVGDILISASGSIGRTIEYTGEDAYYQDSNIVWLNHNDEVINKYLKYFYKIVKWSGIEGTTIKRLYNKNILNTKIELPTVEEQYKMANFLSKLDKIIDIQIEKIELLKQRKQGLLQKMFV
> S.Sau1845ORF2199P TRD NOVEL 4 + TRD f*
MSNTQKKNVPELRFPEFEGEWKDVKFVSIFQEVSNKTSDLAKYPLFSLTVEKGITPKTERYKRDFLVKKSDNFKIVEPRDIVYNPMNVTLGAIDLSKYNYDIALSGYYHVMKIINSFNPDFISNFLKTEKMIIHYK KIATGSLMEKQRVHFSEFKNIIKKFPTNKEQQKIGDFFSKLDRQIELQVQKLELLQQQKKGYMQKIFSQELRFKDENGEDYPDWKEKKLGDITEQSMYGIGASATRFDSKNIYIRITDIDEKSRKLNYQNLTTPDE LNNKYKLKRNDILFARTGASTGKSYIHKEEKDIYNYYFAGFLIKFEIDEQNNPLFIYQFTLTSKFNKWVKVMSVRSGQPGINSEEYAKLPLVLPNKLEQQKIAEFLDRFDQQIELEKQKIEILQQQKKGLLQSMFI

The new TRDs of unknown specificity are termed NOVEL 1, NOVEL 2, NOVEL 3 and NOVEL 4. TRD NOVEL 3 is a second TRD while the others are first TRDs in the HsdS amino acid sequence. Subspecies 21343 and species KPL1845 also contain S.SauNQ (S.Sau21343ORF1169P and S.Sau1845ORF2596P respectively).

### Improving transformation of *S. aureus* by avoiding targets recognized by the *Sau1* Type I RM family

A general method of preparing DNA suitable for transformation of *S. aureus* which can overcome the RM barrier should be possible. Several DNA MTases belonging to Type II RM systems have been found which have extremely short target recognition sites, namely Hin1523, Nma1821 and Hia5 (70) and EcoGII recognizing and methylating adenine in the targets 5΄-A-3΄, 5΄-AB-3΄ or 5΄-BA-3΄. The methylation performed by these enzymes should protect any DNA molecule from the RM enzymes described here (or indeed any RM barrier relying upon adenine methylation). Thus, DNA methylated *in vitro* with these unusual MTases could be used in subsequent transformation experiments even when major RM barriers are present.

**Figure 3.**
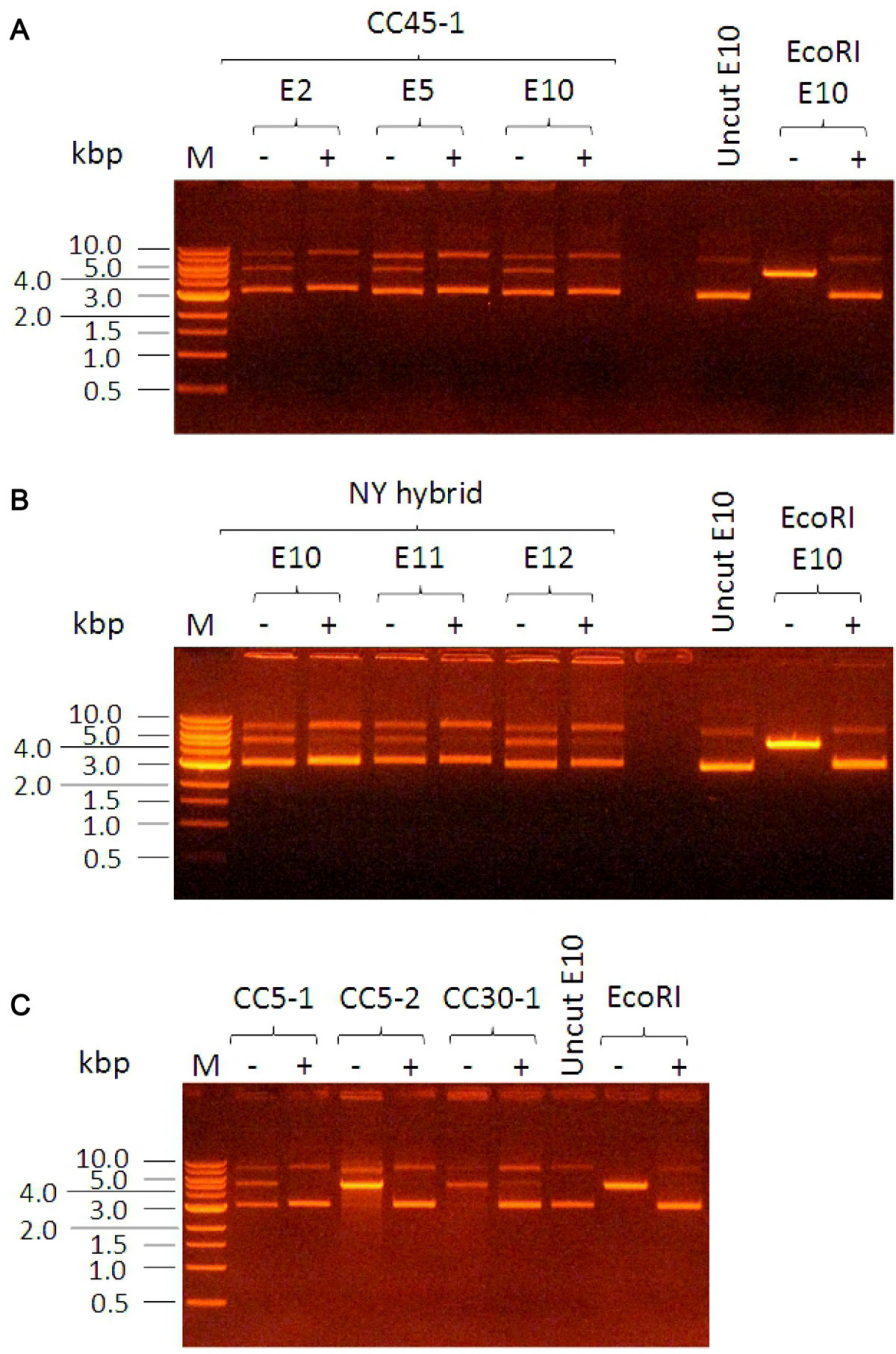
General protection from endonuclease activity using M.EcoGII MTase to methylate all adenines. Plasmid without M.EcoGII treatment is digested (− lanes) but plasmid with M.EcoGII treatment is protected from digestion (+ lanes). Panel (**A**) uses Sau347I (CC45-1, TRDs C and L) restriction enzyme against plasmids E2, E5 and E10 described in (31). Panel (**B**) uses SauNY (TRDs N and Y) against plasmids E10, E11 and E12 described in (31). Panel (**C**) uses three different enzymes, SauN315I (CC5-1, TRDs B and D), SauN315II (CC5-2, TRDs A and H) and SauMRSII (CC30-1, TRDs C and D), against plasmid E10. In each panel EcoRI restriction enzyme was used as a control and markers (M) are in kb.

We used the M.EcoGII adenine MTase (a kind gift from Iain Murray, New England Biolabs) to modify all adenines in several plasmids *in vitro*. The plasmids were from our collection of plasmids used to determine the target sequences of the *S. aureus* Type I enzymes and have been previously described (31). These plasmids were then mixed with various purified *S. aureus* Type I restriction enzymes or, as a control, the EcoRI restriction enzyme. After one hour of methylation by M.EcoGII, the plasmids were completely resistant to digestion by EcoRI and by the *S. aureus* restriction enzymes (Figure 3). Furthermore, the shuttle vector pCN36 (47) was also protected from digestion by these same enzymes (data not shown). Subsequent experiments using the methylated pCN36 to transform *S. aureus* were unfortunately entirely unsuccessful (J. A. Lindsay, unpublished results using strains HO5096 (CC22), JE2 (CC8) and RN4220 (CC8, *hsdR*^−^). The reason for the failure of transformation with the highly-methylated pCN36 when it should be resistant to all *Sau1* RM systems is not clear. This result may imply a further unrecognized barrier to transformation of *S. aureus* or some aspect of the physical properties of highly methylated DNA. Nevertheless, the method using MTases with very short target recognition sequences may be of use for transformation of other bacterial species.

## CONCLUSIONS

In conclusion, we have determined the target recognition sequences of a considerable number of TRDs and HsdS specificity subunits of the Type I RM systems in *S. aureus*. This was achieved using a combination of gene synthesis, endonuclease activity, ATP hydrolysis activity and single molecule real-time genome sequencing. The systems analyzed cover a large proportion of the known sequence types and clonal complexes of *S. aureus* and delineate more clearly the barrier to HGT within the *S. aureus* population.

The data obtained here will allow the construction of new *E. coli* strains for preparing methylated shuttle vectors (35) and MTase reagents for *in vitro* methylation of DNA (40) to assist transformation of further *S. aureus* strains. However, these approaches are time consuming and it is worth noting that the common shuttle vector used for transformation of *S. aureus*, pCN36 (47), contains a target site for almost every TRD pair investigated in this paper. This means that pCN36 is inevitably a poor vector for transformation of *S. aureus*. The construction of new shuttle vectors completely lacking *Sau1* targets via DNA synthesis, coupled with careful analysis of the fragments to be ligated into the vector so that they also lack targets, may be an effective way forward to improve transformation of *S. aureus* now that so many target specificities have been determined. Obviously, the avoidance of the sequence AN_6-9_T, although difficult to achieve without altering protein coding sequences in a vector, would be a general method to negate the effect of the Type I RM systems in *S. aureus* and other prokaryotes.

Lastly, the determination of so many recognition sequences of Type I RM systems in different lineages of *S. aureus*, in effect a ‘Rosetta Stone’, means that now the population structure of *S. aureus* can be investigated from an epigenetic/evolutionary perspective (4) as performed previously with, for example, *Helicobacter pylori* (71) and *Streptococcus pneumoniae* (72).

## Supplementary Material

Supplementary Data
