# Supplementary material for: DNA target recognition domains in the Type I restriction and modification systems of Staphylococcus aureus
Source: Nucleic Acids Res. 2017 Feb 9;45(6):3395–406. doi: 10.1093/nar/gkx067 (PMC5399793; doi:10.1093/nar/gkx067)
Supplement: Supplementary Data [file gkx067_Supp.pdf]

SUPPLEMENTARY INFORMATION

DNA target recognition domains in the Type I restriction/modification systems of Staphylococcus aureus.

Laurie P. Cooper, Gareth A. Roberts, John H. White, Yvette A. Luyten, Edward K.M. Bower, Richard D. Morgan, Richard J. Roberts, Jodi A. Lindsay, David T.F. Dryden.

Pages 2 and 3: SUPPLEMENTARY INFORMATION FOR TABLE 1.

Pages 4 to 9: Supplementary information for MATERIALS AND METHODS SECTION "Construction of further MTases with further combinations of TRDs using synthetic genes."

Pages 10 to 16: SUPPLEMENTARY INFORMATION FOR TABLE 2.

Pages 17 to 57: SUPPLEMENTARY INFORMATION FOR TABLE 3.

Pages 58 to 85: SUPPLEMENTARY INFORMATION FOR TABLE 4
Pages 86 to 91: SUPPLEMENTARY INFORMATION FOR TABLES 5 AND 6.

Pages 92 to 97: PROMALS ALIGNMENT OF TRD AMINO ACID SEQUENCES WITH SECONDARY STRUCTURE PREDICTIONS.

SUPPLEMENTARY INFORMATION FOR TABLE 1.
The amino acid sequences in FASTA format of the first TRD with its letter code, DNA target (5′ to 3′) and methylation site underlined. The TRDs labelled as NOVEL 1, NOVEL 2 and NOVEL 4 were found once all of the other TRDs had been analysed but are included in this list for completeness.
The TRD sequences are flanked by the conserved regions so to obtain the amino acid sequence of any HsdS subunit simply paste the sequence for the second TRD directly on to the end of the sequence for the first TRD.

>A CCAY
MSNTQKKNVPELRFPGFEGEWEEKQLGDLTTKIGSGKTPKGGSENYTNKGIPFLRSQNIRNGKLNLNDLVYISKDIDDEMKNSRTYYGDVLLNITGASIG
RTAINSIVETHANLNQHVCIIRLKKEYYYIFFGQYLLSRKGKRKIFLAQSGGSREGLNFKEIANLKIFTPTIFEEQQKIGKFFSKLDRQIELEEQKLELL
QQQ
>B AGG
MSNTQKKNVPELRFPGFEGEWEEKQLGDLTDRVIRKNKNLESKKPLTISGQLGLIDQTEYFSKSVSSKNLENYTLIKNGEFAYNKSYSNGYPLGAIKRLT
RYDSGVLSSLYICFSIKSEMSKDFMEAYFDSTHWYREVSGIAVEGARNHGLLNVSVNDFFTILIKYPSLEEQQKIGKFFSKLDRQIELEEQKLELLQQQ
>C GWAG
MSNTQTKNVPELRFPGFEGEWEEKQVGELLEFKNGLNKGKEYFGSGSSIVNFKDVFNNRSINTNNLTGKVNVNSKELKNYSVEKGDVFFTRTSEVIGEIG
YPSVILNDPENTVFSGFVLRGRPKSGIDLINNNFKRYVFFTNSFRKEMITKSSMTTRALTSGTAINKMKVIYPVSAKEQKKIGDFFSKLDRQIELEEQKL
ELLQQQ
>J GGA
MSNTQKKNVPELRFPEFEGEWEERKLGDLIKVNSGKDYKHLDKGDIPVYGTGGYMTSVSEPLSEIDAVGIGRKGTINKPYLLEAPFWTVDTLFYCTPEKE
ADILFILSLFRKINWKLYDESTGVPSLSKQTINKINRLVPTNKEQQKIGEFFSKLDRQIELEEQKLELLQQQ
>M CAG
MSNTQTKNVPELRFPGFEGEWEEKKLEDLGLFQKSYSFSRAKEGNGKTKHIHYGDIHSKFKTVLDSDGNIPNIIEKAVFELIQKGDIVFADASEDYSDLG
KAVMIDFEPNSLISGLHTHLFRPLNNAISNFLIFYTKTLSYKKFIRQQGTGISVLGISKKSLLNLNVLIPRSELEQQKIGQFFSKLDRQIELEEQKLELL
QQQ
>N ACC
MSNTQKKNVPELRFPGFEGEWEEKKLGEFAGKVTQKNVDKKYIETLTNSAELGIISQKDYFDKEISNIDNIKKYYVVEENDFVYNPRMSNYAPFGPVNRN
KLGKKGVMSPLYTVFKIQNIDLNFIEFYFKSSKWYRFMALNGDSGARADRFSIKDRTFMEMPLHIPCMDEQIKIGQFFSKLDRQIELEEQKLELLQQQ

>O CAAC
MSNKQKKNVPELRFPGFEGEWEEKKLGEVGTFTSGGTPLKSKSEYWNGDIPWITTGDIHNIKRENITNFITEKGLNESSAKLITNEAILIAMYGQGKTRG
MSAILNFEATTNQACAIYQTNQNINFVFQYFQKLYEFLRSLSNEGSQKNLSLSLLKEITLNYPNEQEQKKIGDFFSKLDRQIELEEQKLELLQQQ
>R GARA
MSNTQKKNVPELRFPGFEGEWEEKKLGEVAKIYDGTHQTPKYTNEGIKFLSVENIKTLNSSKYISEEAFEKEFKIRPEFGDILMTRIGDIGTPNIVSSNE
KFAYYVSLALLKTKNLNSYFLKNLILSSSIQNELWRKTLHVAFPKKINKNEIGKIKINYPKKQEQQKIGQFFSKLDRQIELEEQKLELLQQQ
>T CAAG
MSNTQTKNVPELRFPGFEGEWEEKELGEIFQIISGSTPLKSNKEFYENGNINWVKTTDLNNSKVTHSKEKITEYAMKSLKLKLVPKNSVLIAMYGGFNQI
GRTGLLKIDATINQAISALLMNHETNPEFIQAFLNYQVKGWKRYAASSRKDPNITKKDIEQFKVPYVSINEQQKIGEFFSKIDHQIELEEQKLELLQQQ
>V CNGA
MSNTGKMNVPELRFPGFEGEWEEKELRELRNPKDKYSYTGGPFGSDLKKSDYTTDGIQIIQLQNIGDGYFYNSNKVFTSNEKAEVLKSCNVFPGDIVIAK
MADPIARAAIVPDNNIGKYLMASDGIRLSVDTVHFNTKFVLECINRKSFRKKVEDNSSGSTRMRIGLSTLGSLTLKTTTLKEQQKIGQFFSKLDRQIVLE
EQKLELLQQQ
>X TCTA
MSNTQKKNVPELRFPGFEGEWEEKQFADFTKINQGLQIAINERKTEYSPELYFYITNEFLRPNSQTKYFIENPPQSVIANKEDILMTRTGNTGKVVTNVF
GAFHNNFFKIKFDKNLYDRLFLVEVLNSSKIQNKILSLAGSSTIPDLNHSDFYSISSSYPLLREQQKIGKFFSKLDRQIELEEQKLELLQQQ
>Z GAC
MSNTQTKNVPELRFPGFEGEYSLDIFGNLATNKSEKFNPQNENASIDIELDCIEQNTGRLIKIYNSKEFSSQKNKFNPQNVLYGKLRPYLNKYYFTKKSG
VCSSEIWVLKSTKEDKLLNLFLYYFIQTKRYSDVASKSAGSKMPRADWGLIENIRVYFPELCEQQKIGQFFSKLDRQIELEEQKLELLQQQ
>b* GGHA
MSNTQKKNAPELRFPEFEGEWKEKKLEDTLEFIKDGTHGTHENVNNGPWLLSAKNIKNNKIIISSDDRKISESDYKKIYKNYKLEKGDLLLTIVGTIGRA
AIVKNPNNIAFQRSVAILKTKATYDVGFIFQLFQTKYFKNLLLRKQVVSAQPGLYLGDIRKIKISITNIIEEQRKIGIFFSKLDRQIELEEQKLELLQQQ
>e* GAG
MSNTQKKNVPELRFPGFEGEWEEKSISSFLKESKIKGSNGSHAKKLTVKLWGKGVVPKKETFKGSDNTQYYKRKAGQLMYGKLDFLNCAFGIVPDSLNNY
ESTIDSPSFDFINGDSKFLLERIKLKSFYKKFGDIANGSRKAKRINQDTFLSLPVFAPKYDEQLRIGEFFSKLDRQIELQKQKLELLQQQ
>NOVEL 1
MSNTQKKNVPELRFPGFEGEWEEKKLGEVATFAKGKLGAKKDVSQNGVPVILYGELYTKYGAIVSKIFSKTDIPENKLKMAKKNDVLIPSSGETAIDIAT
ASCIYLNKGVAVGGDINILTPQKQDGRFISLSINGINKNELSKYAQGKTVVHLYNNDIKNLKIAFPSEFEEQVRIGNFFSKLDRQIELEEQKLELLQQQ
>NOVEL 2
MSNTQKKNVPELRFPEFEGEWKDVKFVSIFQEVSNKTSDLAKYPLFSLTVEKGITPKTERYKRDFLVKKSDNFKIVEPRDIVYNPMNVTLGAIDLSKYNY
DIALSGYYHVMKIINSFNPDFISNFLKTEKMIIHYKKIATGSLMEKQRVHFSEFKNIIKKFPTNKEQQKIGDFFSKLDRQIELQVQKLELLQQQ
>NOVEL 4
MTEQINTPELRFPEFKNEWSYDLVSDVVTNKSKKFDPKKEEAKKDIELDSIEQNTGRLLDTYISNDFTSQKNKFNKGNVLYSKLRPYLNKYYYATIDGVC
SSEIWVLNTLNKDVLANKFLYYFIQTNRFSSVTNKSAGSKMPRADWELVKNIRLYKGSIEEQEKIGYFFSKLDRQIELEEKKLELLEQQQ

The amino acid sequences in FASTA format of the second TRD with its letter code, DNA target (5′ to 3′) and its methylation site underlined. The TRD labelled as NOVEL 3 was found once all of the other TRDs had been analysed but is included in this list for completeness.

>D ATC
KKGYMQKIFSQELRFKDENGEDYPHWENSKIEKYLKERNERSDKGQMLSVTINSGIIKFSELDRKDNSSKNKSNYKVVRKNDIAYNSMRMWQGASGKSNY
NGIVSPAYTVLYPTQNTSSLFIGYKFKTHRMIHKFKINSQGLTSDTWNLKYKQLKNINIDIPVLEEQEKIGDFFKKMDILISKQKIKIEILEKEKQSFLQ
KMFL
>E TCAY
KKGYMQKIFSQELRFKDENGNDYPEWEETTIKEIAQINXGKKDTKDAITNGSYDFYVRSPIVYKINTFSYEGEAILTVGDGVGVGKVFHYVNGKFDYHQR
VYKISDFKNYYGLLLFYYFSQNFLKETKKYSAKTSVDSVRKDMIANMKVPRPIYIEQKKIGQFIKRVDNKTKIQKQVIELLKQRKKALLQKMFI
>F TTAA
KKGYMQKIFSQELRFKDEEGKDYPDWKSKSIQEIFENKGGTALETEFNFDGNYKVISIGSYSINSTYNDQNIRVNKNKKTEKYILSKGDLAMVLNDKTKD
GKIIGRSIFIDKDNQYIYNQRTERLIPFAENDNKFLWFLMNTDLIRNKIKGMMQGATQVYINYSSIKLISIQLPLLEEQQKIRGFLEVLSGITTKQLHKI
DQLKERKKAFLQKMFI
>G ACA
KKGYMQKIFTQELRFKDENGEEYPEWENKFIKDIFIFENNRRKPITSSLREKGLYPYYGATGIIDYVKDYLFNNEERLLIGEDGAKWGQFETSSFIANGQ
YWVNNHAHVVKSNDHNLFFMNYYLNFKELRAFVTGNAPAKLTHANLCNINLKIPCLTEQDKVSALLKSIDNKMNNQMNRIELLKERKKELLQKMFI
>H TAC
KKCYIQKIFSQELRFKDEEGNYYKGWNKKQLKDVLEFSNKRTINENEYPVLTSSRQGLILQSDYYKDRKTFAESNIGYFILPKNHITYRSRSDDGIFKFN
LNLMIDVGIISKYYPVFKGIDANQYYLTLHLNYQLKKEYIKYATGTSQLVLSQKDLQNIKTKLPSYEEQQKIGDFFSEIDRLVEKQSSKVGRLKVRKKEL
LQKMFV
>I YTCA
KKGYMQKIFSQELRFKNENGNDYPDWERIKFFDVIDKVIDFRGRTPKKLNMEWSDEGYLALSAVNVKKGYIDFNVEAKYGNLDLYTRWMRGNELYKGQVL
FTTEAPMGNVAQVPDNKGYILSQRTIAFNSNEKITDNFLASLLSSENVYNDLLKLCSGATAKGVSQKNLNRLYVTIPHSISEQEEIAEFFRKINQLVELQ
KYKIEHTKSQKQVFLQKMFI
>K CGA
KKGYMQKIFSQELRFKDENGNDYPKWEEKKIEDIASQVYGGGTPNTKIKEFWNGDIPWIQSSDVKVNDLILQQCNKFISKNSIELSSAKLIPANSIAIVT
RVGVGKLCLVEFDYATSQDFLSLSSLKYDKLYSLYSLLYTMKKISANLQGTSIKGITKKELLDSIIKIPHNLEEQQKIGDLFYKIDKYISFNKCKIEMLK
SLKQGLLKKMFI
>L TTTA
KKGYMQKIFSQELRFKDENGNDYPNWRTIELKNILENIVDNRGKTPDNAPSEKYPLLEVNALGYYRPAYIKVSKFVSENTYNNWFREHLKENDILFSTVG
NTGIVSLMDNYKAVIAQNIVGLRVNNNNLPSFIYYMLSYKGNQKKIKRIQMGAVQPSVKVSQFKFIKYLVPIKDEQEKVAKLLIEIDKLVNKQLIKIELL
QQRKKALLKSMFI
>P AGG
KKGYMQKIFSQELRFKDESGNDYPDWEEKELGEVADRVIRKNKNFESKKPLTISGQLGLIDQTEYFSKSVSSKNLENYTLIKNGEFAYNKSYSNGYPLGA
IKRLTRYDSGVLSSLYICFSIKSEMSKDFMEAYFDSTHWYREVSGIAVEGARNHGLLNISVNDFFTILIKYPSLEEQRKIGDFFIKLDRQIELEEQKLEL
LQQRKKALLKSMLI
>Q ACAY
KKGYMQKIFSQELRFKDENGEDYSEWEERRFADIFKFHNKLRKPIKENLRVKGSYPYYGATGIIDYVDDFIFDGNYLLIGEDGANIITRSAPLVYLVNGK
FWVNNHAHILSPLNGNIQYLYQVAELVNYEKYNTGTAQPKLNIQNLKIINVVISTNLEEQQKIGSFLSKLDRQIDLEEQKLELLQQRKKALLKSMFV
>S GCA
KKGYMQKIFSQELRFKDENGNDYPDWTNERLGEVTTVTMGQSPKSVNYTDNSNDTVLIQGNADIENGLINPRIYTREVTKLIQKDEIILTVRAPVGKLAM
AQINACIGRGVCSIKGDKFLYYFLEWFATQNKWIRFSQGSTFESISGNDIRNIHIKIPVEDERTKIIKLLNSLDVLNSKTDLKIQNLKQRKQSLLQKIFV
>U GAY
KKGYMQKIFSQELRFKDENGEDYPDWEVTTIQNITKYTSSKKSSNQYADKDNSKGYPVYDAVQEIGKDSNYDIEESYISILKDGAGVGRLNLRPGKSSVI
GTMGYIQSNNVDIEFLYYRMKVVDFKKYIIGSTIPHLYFKDYSKETLYIPSSIQEQAKIGMFISNLDKLIENKNLKLNCLKQLKQGLLQSMFI
>W CRAA
KKGYMQKIFSQELRFKDENGNDYPDWEEKQLGELSQIVRGASPRPIKDPKWFNKESDIGWLRISDVTNQNGKIYHLEQKLSIEGQEKTRVLVTTHLLLSI
AASIGKPVMNFVKTGVHDGFLIFLKPKFNLFFMYYWLEYFKDKWSKYGQPGSQVNLNSEIVKSQTLNMPSNHEQEKVGQFFNRNEKLIELQQEKIMYIKR
CKQVLLQKMFI
>Y CTA
KKGYMQKIFSQELRFKDENGNDYPDWEKKKLKEIACVYTGNTPSKKENIYWNKGEYVWVTPTDINNSKNIYESENKLTQEGYKKARQLPENTLLVTCIAS
IGKNAILRKQGSCNQQINAVVPFENINIDYLYYISDSLSTFMKSIAGKTATQIVNKNTFENLEIYLAPFEEQNKIADLISSLEELIEKQASKLIKMKSRK
QGMLQIMFI
>a* GAA
KKGYMQKIFSQELRFKDENGNDYPEWENKRIEDIANVNKGFTPSTNNNEYWDNNDKNWLSIAGMNQKYLYKGNKGISKDAAKNYMKVKNDTLIMSFKLTI
GKLAIVKAPLYTNEAICHFIWKVNKINTEFIYYYLNSLNISTFGVQAVKGVTLNNDSINSIIVKLPNEEEQNIIAKFLLEVDKTVNNQLVKTKLLKQRKK
GLLQRMFV
>c* GAY
KKGYLQKIFSQELRFKDENGNDYPEWRFARFKDFMYKPINIRPAINISKSELLTVKLHCKGIEKANINRVLKLGATNYYKRFEGQFIYGKQNFFNGAFDI
VPKKFDGLYSSSDVPAFEINTEKIEPNYFISYISRPSFYKSKEKYSTGTGSKRIHENTVLNFSLHLPCLNEQLKIASFVCFLNRKIELLERKIYLIKKQK
QALLQQMFI
>d* CYAA
KKGYMQKIFSQELRFKDENGNDYPEWENVMLQKVLKDKTEGIKRGPFGGALKKDIFVESGYAVYEQRNAIYDISNFRYYINENKYKEMQSFSVQPNDIIM
SCSGTIGRLALIPHNYTKGIINQALIRFRTNHKIRSEFFLIFMRSNQMQRKILEANPGSAITNLVPVKELKLIPFPLPVKFEQDKISQFIHIINRRIEQS
EKKIESLKNRKQGFLQKLFV
>f* GAAY
KKGYMQKIFSQELRFKDENGEDYPDWKEKKLGDITEQSMYGIGASATRFDSKNIYIRITDIDEKSRKLNYQNLTTPDELNNKYKLKRNDILFARTGASTG
KSYIHKEEKDIYNYYFAGFLIKFKINEQNSPLFIYQFTLTSKFNKWVKVMSVRSGQPGINSEEYAKLPLVLPNKLEQQKIAKFLDRFDRQIELEKQKIEI
LQQQKKGLLQSMFI
>NOVEL 3
KKGYMQKIFAQELRFKDENGNDYPDWVTKKLGDIGKVAMNKRIYKNETTENGEIPFYKIGNFGKNADTFITREKFDEYKEKYPYPNVGDILISASGSIGR
TIEYTGEDAYYQDSNIVWLNHNDEVINKYLKYFYKIVKWSGIEGTTIKRLYNKNILNTKIELPTVEEQYKMANFLSKLDKIIDIQIEKIELLKQRKQGLL
QKMFV

SUPPLEMENTARY INFORMATION FOR MATERIALS AND METHODS SECTION "CONSTRUCTION OF FURTHER MTASES WITH FURTHER COMBINATIONS OF TRDS USING SYNTHETIC GENES."

The DNA sequence is followed by the predicted amino acid sequence for each TRD. Some synthetic sequences encoded two TRDs.

CC15 TRD O

CCCGGGTTTGAAGGCGAATGGGAGGAAAAAAAACTGGGTGAAGTTGGCACCTTTACCAGCGGTGGC
ACTCCGCTGAAAAGCAAAAGCGAATATTGGAATGGTGATATTCCGTGGATTACCACAGGCGATATT
CATAACATTAAACGCGAAAACATCACCAACTTTATCACCGAAAAAGGCCTGAATGAAAGCAGCGCA
AAACTGATTACCAATGAAGCAATTCTGATTGCCATGTATGGTCAGGGTAAAACCCGTGGTATGAGC
GCCATTCTGAATTTTGAAGCAACCACCAATCAGGCCTGTGCAATTTATCAGACAAACCAGAACATC
AACTTCGTGTTCCAGTATTTCCAGAAACTGTATGAATTTCTGCGTAGCCTGAGCAATGAAGGTAGC
CAGAAAAATCTGAGCCTGAGCCTGCTGAAAGAAATTACCCTGAATTATCCGAACGAGCAAGAACAG
AAAAAAATCGGCGATTTCTTCAGCAAACTGGATCGTCAAATTGAATTAGAAGAACAGAAG

CC15 TRD O

PGFEGEWEEKKLGEVGTFTSGGTPLKSKSEYWNGDIPWITTGDIHNIKRENITNFITEKGLNESSA
KLITNEAILIAMYGQGKTRGMSAILNFEATTNQACAIYQTNQNINFVFQYFQKLYEFLRSLSNEGS
QKNLSLSLLKEITLNYPNEQEQKKIGDFFSKLDRQIELEEQK

CC51 TRD P

CAAATTGAATTAGAAGAACAGAAGCTGGAACTGTTTCAGCAGCAGAAAAAAGGCTATATGCAGAAA
ATCTTTAGCCAAGAGCTGCGCTTTAAAGATGAAAGCGGTAATGATTATCCGGATTGGGAAGAAAAA
GAACTGGGTGAAGTTGCAGATCGTGTGATTCGTAAAAACAAAAACTTTGAAAGCAAAAAACCGCTG
ACCATTAGCGGTCAGCTGGGTCTGATTGATCAGACAGAATATTTCAGCAAAAGCGTGAGCAGCAAA
AACCTGGAAAACTATACCCTGATTAAAAACGGCGAGTTCGCCTATAACAAAAGCTATAGCAATGGT
TATCCGCTGGGTGCAATTAAACGTCTGACCCGTTATGATAGCGGTGTTCTGAGCAGCCTGTATATT
TGCTTTAGCATCAAAAGCGAGATGAGCAAAGATTTCATGGAAGCCTATTTTGATAGCACCCATTGG
TATCGTGAAGTTAGCGGTATTGCAGTTGAAGGTGCACGTAATCATGGTCTGCTGAATATTAGCGTG
AACGATTTTTTCACCATCCTGATCAAATATCCGAGCCTGGAAGAACAGCGTAAAATCGGTGATTTC
TTCATTAAACTGGATCGCCAGATTGAGCTGGAAGAACAAAAACTGGAACTGCTGCAACAGCGCAAA
AAAGCACTGCTGAAAAGTATGCTGATCCCCGGGGGATCCGATCGATC

CC51 TRD P

QIELEEQKLELFQQQKKGYMQKIFSQELRFKDESGNDYPDWEEKELGEVADRVIRKNKNFESKKPL
TISGQLGLIDQTEYFSKSVSSKNLENYTLIKNGEFAYNKSYSNGYPLGAIKRLTRYDSGVLSSLYI
CFSIKSEMSKDFMEAYFDSTHWYREVSGIAVEGARNHGLLNISVNDFFTILIKYPSLEEQRKIGDF
FIKLDRQIELEEQKLELLQQRKKALLKSMLI

CC72-1 TRD R + CC59-1 TRD Q

CCCGGGTTTGAAGGCGAATGGGAGGAAAAAAAACTGGGTGAAGTGGCCAAAATCTATGATGGCACC
CATCAGACCCCGAAATATACCAATGAAGGTATCAAATTTCTGAGCGTGGAAAACATCAAAACCCTG
AATAGCAGCAAATACATTAGCGAAGAAGCCTTCGAGAAAGAATTCAAAATTCGTCCGGAATTTGGC
GATATTCTGATGACCCGTATTGGTGATATTGGCACCCCGAATATTGTTAGCAGCAATGAAAAATTC
GCCTACTATGTTAGCCTGGCACTGCTGAAAACCAAAAATCTGAACAGCTACTTCCTGAAAAACCTG
ATTCTGAGCAGCAGCATTCAGAATGAACTGTGGCGTAAAACCCTGCATGTTGCATTTCCGAAAAAA
ATCAACAAAAACGAGATCGGCAAAATCAAAATCAACTACCCGAAAAAACAAGAACAGCAGAAAATC
GGTCAGTTTTTCAGCAAACTGGATCGCCAAATTGAATTAGAAGAACAGAAGCTGGAACTGCTGCAA
CAGCAGAAAAAAGGTTATATGCAGAAAATCTTCAGCCAAGAGCTGCGCTTTAAAGATGAAAATGGT
GAAGATTATAGCGAGTGGGAAGAACGTCGTTTTGCCGATATTTTCAAATTTCACAACAAACTGCGC
AAACCGATCAAAGAAAATCTGCGTGTTAAAGGCAGCTATCCGTATTATGGTGCAACCGGCATTATT
GATTATGTGGATGATTTTATCTTCGATGGCAACTATCTGCTGATTGGCGAAGATGGTGCAAACATT
ATTACCCGTAGCGCACCGCTGGTTTATCTGGTTAATGGTAAATTTTGGGTGAACAACCATGCCCAT
ATTCTGAGTCCGCTGAATGGTAATATTCAGTATCTGTATCAGGTTGCCGAACTGGTGAACTATGAA

AAATACAATACCGGCACCGCACAGCCGAAACTGAACATTCAGAATCTGAAAATTATCAACGTGGTG
ATCAGCACCAATCTGGAAGAACAGCAAAAAATTGGTAGCTTCCTGAGCAAACTGGATCGTCAGATT
GACCTGGAAGAACAAAAACTGGAACTGCTGCAACAACGTAAAAAAGCACTGCTGAAAAGCATGTTC
GTGCCCGGGGGATCCGATCGATC

CC59-1 TRD Q

QIELEEQKLELLQQQKKGYMQKIFSQELRFKDENGEDYSEWEERRFADIFKFHNKLRKPIKENLRV
KGSYPYYGATGIIDYVDDFIFDGNYLLIGEDGANIITRSAPLVYLVNGKFWVNNHAHILSPLNGNI
QYLYQVAELVNYEKYNTGTAQPKLNIQNLKIINVVISTNLEEQQKIGSFLSKLDRQIDLEEQKLEL
LQQRKKALLKSMFV

CC72-1 TRD R

PGFEGEWEEKKLGEVAKIYDGTHQTPKYTNEGIKFLSVENIKTLNSSKYISEEAFEKEFKIRPEFG
DILMTRIGDIGTPNIVSSNEKFAYYVSLALLKTKNLNSYFLKNLILSSSIQNELWRKTLHVAFPKK
INKNEIGKIKINYPKKQEQQKIGQFFSKLDRQIELEEQK

CC75-1 TRD T and TRD U

CCCGGGTTTGAAGGCGAATGGGAGGAAAAAGAACTGGGCGAAATCTTTCAGATTATTAGCGGTAGC
ACACCGCTGAAAAGCAACAAAGAATTTTATGAGAACGGCAACATCAACTGGGTTAAAACCACCGAT
CTGAATAATAGCAAAGTGACCCATAGCAAAGAAAAAATCACCGAGTATGCAATGAAAAGCCTGAAA
CTGAAACTGGTGCCGAAAAATAGCGTTCTGATTGCAATGTATGGTGGCTTTAATCAGATTGGTCGT
ACCGGTCTGCTGAAAATTGATGCAACCATTAATCAGGCAATTAGCGCACTGCTGATGAATCATGAA
ACCAACCCGGAATTTATTCAGGCCTTTCTGAATTATCAGGTGAAAGGTTGGAAACGTTATGCAGCA
AGCAGCCGTAAAGATCCGAATATCACCAAAAAAGATATCGAACAGTTCAAAGTGCCGTACGTGAGC
ATTAATGAACAGCAGAAAATTGGCGAGTTTTTTAGCAAAATCGATCATCAAATTGAATTAGAAGAA
CAGAAGCTGGAACTGCTGCAACAGCAGAAAAAAGGTTATATGCAGAAAATCTTCAGCCAAGAGCTG
CGCTTTAAAGATGAAAATGGTGAAGATTATCCGGATTGGGAAGTTACCACCATTCAGAACATTACC
AAATACACCAGCAGCAAAAAAAGCAGCAATCAGTATGCCGATAAAGACAACAGCAAAGGTTATCCG
GTTTATGATGCCGTTCAAGAAATTGGCAAAGATAGCAACTATGACATCGAAGAGAGCTATATCAGC
ATTCTGAAAGATGGTGCCGGTGTTGGTCGTCTGAATCTGCGTCCGGGTAAAAGCAGCGTTATTGGC
ACCATGGGTTATATTCAGAGCAACAACGTGGATATCGAGTTCCTGTATTATCGTATGAAAGTGGTG
GACTTCAAAAAATACATTATCGGTAGCACCATTCCGCACCTGTATTTCAAAGATTATAGCAAAGAA
ACCCTGTACATTCCGAGCAGCATTCAAGAACAGGCAAAAATTGGTATGTTCATCAGCAACCTGGAT
AAACTGATCGAGAACAAAAACCTGAAACTGAACTGTCTGAAACAACTGAAACAGGGATTGCTACAA
TCTATGTTTATTCCCGGGGGATCCGATCGATC

CC75-1 TRD T

PGFEGEWEEKELGEIFQIISGSTPLKSNKEFYENGNINWVKTTDLNNSKVTHSKEKITEYAMKSLK
LKLVPKNSVLIAMYGGFNQIGRTGLLKIDATINQAISALLMNHETNPEFIQAFLNYQVKGWKRYAA
SSRKDPNITKKDIEQFKVPYVSINEQQKIGEFFSKIDHQIELEEQK

CC75-1 TRD U

QIELEEQKLELLQQQKKGYMQKIFSQELRFKDENGEDYPDWEVTTIQNITKYTSSKKSSNQYADKD
NSKGYPVYDAVQEIGKDSNYDIEESYISILKDGAGVGRLNLRPGKSSVIGTMGYIQSNNVDIEFLY
YRMKVVDFKKYIIGSTIPHLYFKDYSKETLYIPSSIQEQAKIGMFISNLDKLIENKNLKLNCLKQL
KQGLLQSMFI

CC75-2 TRD V

CCCGGGTTTGAAGGCGAATGGGAGGAAAAAGAACTGCGTGAACTGCGCAATCCGAAAGATAAATAC
AGCTATACCGGTGGTCCGTTTGGTAGCGATCTGAAAAAAAGCGATTATACCACCGATGGCATTCAG
ATTATTCAGCTGCAGAATATTGGTGACGGCTATTTCTATAACAGCAACAAAGTGTTTACCAGCAAC
GAAAAAGCCGAAGTTCTGAAAAGCTGTAATGTTTTTCCGGGTGATATTGTGATTGCCAAAATGGCA
GATCCGATTGCACGTGCCGCAATTGTTCCGGATAATAACATTGGTAAATACCTGATGGCCAGTGAT
GGTATTCGTCTGAGCGTTGATACCGTTCATTTTAACACCAAATTTGTGCTGGAATGCATCAACCGT
AAAAGCTTTCGTAAAAAAGTCGAGGATAATAGCAGCGGTAGCACCCGTATGCGTATTGGTCTGAGT
ACCCTGGGTAGCCTGACCCTGAAAACCACCACCCTGAAAGAACAGCAGAAAATTGGTCAGTTTTTC

AGCAAACTGGATCGTCAAATTGAATTAGAAGAACAGAAG

CC75-2 TRD V

PGFEGEWEEKELRELRNPKDKYSYTGGPFGSDLKKSDYTTDGIQIIQLQNIGDGYFYNSNKVFTSN
EKAEVLKSCNVFPGDIVIAKMADPIARAAIVPDNNIGKYLMASDGIRLSVDTVHFNTKFVLECINR
KSFRKKVEDNSSGSTRMRIGLSTLGSLTLKTTTLKEQQKIGQFFSKLDRQIELEEQK

CC75-2 TRD W

CAAATTGAATTAGAAGAACAGAAGCTGGAACTGCTGCAACAGCAGAAAAAAGGTTATATGCAGAAA
ATCTTCAGCCAAGAGCTGCGCTTTAAAGATGAAAATGGTAACGATTATCCGGATTGGGAAGAAAAA
CAGCTGGGTGAACTGAGCCAGATTGTTCGTGGTGCAAGTCCGCGTCCGATTAAAGATCCGAAATGG
TTTAACAAAGAAAGCGATATTGGTTGGCTGCGCATTAGTGATGTTACCAATCAGAATGGCAAAATC
TATCATCTGGAACAGAAACTGAGCATCGAAGGTCAAGAAAAAACCCGTGTTCTGGTTACCACCCAT
CTGCTGCTGAGCATTGCAGCAAGCATTGGTAAACCGGTTATGAACTTTGTGAAAACCGGTGTGCAT
GATGGCTTTCTGATTTTTCTGAAACCGAAATTCAACCTGTTCTTTATGTACTATTGGCTGGAATAT
TTCAAAGATAAATGGTCCAAATATGGTCAGCCTGGTAGCCAGGTTAATCTGAATAGCGAAATTGTT
AAAAGCCAGACCCTGAATATGCCGAGCAATCATGAACAAGAAAAAGTGGGCCAGTTTTTTAACCGC
AACGAAAAACTGATTGAACTGCAGCAAGAGAAAATCATGTATATCAAACGTTGCAAACAGGTGCTG
CTGCAAAAAATGTTTATTCCCGGGGGATCCGATCGATC

CC75-2 TRD W

QIELEEQKLELLQQQKKGYMQKIFSQELRFKDENGNDYPDWEEKQLGELSQIVRGASPRPIKDPKW
FNKESDIGWLRISDVTNQNGKIYHLEQKLSIEGQEKTRVLVTTHLLLSIAASIGKPVMNFVKTGVH
DGFLIFLKPKFNLFFMYYWLEYFKDKWSKYGQPGSQVNLNSEIVKSQTLNMPSNHEQEKVGQFFNR
NEKLIELQQEKIMYIKRCKQVLLQKMFI

CC80-1 TRD X and TRD Y

CCCGGGTTTGAAGGCGAATGGGAGGAAAAACAGTTTGCCGACTTCACCAAAATTAACCAGGGTCTG
CAGATTGCCATTAATGAACGTAAAACCGAATATAGCCCTGAGCTGTATTTCTATATCACCAACGAA
TTTCTGCGTCCGAATAGCCAGACCAAATATTTCATTGAAAATCCGCCTCAGAGCGTGATTGCCAAC
AAAGAAGATATTCTGATGACCCGCACCGGTAATACCGGCAAAGTTGTTACCAATGTTTTTGGTGCC
TTCCACAACAACTTTTTCAAAATCAAATTCGATAAAAACCTGTATGATCGCCTGTTTCTGGTTGAA
GTTCTGAACAGCAGCAAAATCCAGAACAAAATTCTGAGCCTGGCAGGTAGCAGCACCATTCCGGAT
CTGAATCATAGCGATTTCTATAGCATTAGCAGCAGCTATCCGCTGCTGCGCGAACAGCAAAAAATT
GGCAAATTCTTTAGCAAACTGGATCGTCAAATTGAATTAGAAGAACAGAAGCTGGAACTGCTGCAA
CAGCAGAAAAAAGGTTATATGCAGAAAATCTTCAGCCAAGAGCTGCGCTTTAAAGATGAAAATGGT
AACGATTATCCGGATTGGGAGAAAAAAAAACTGAAAGAAATTGCCTGCGTGTATACCGGTAATACC
CCGAGCAAAAAAGAAAACATCTATTGGAACAAAGGCGAGTATGTTTGGGTTACCCCGACCGATATT
AACAACAGCAAAAACATTTATGAAAGCGAAAACAAACTGACCCAAGAAGGCTACAAAAAAGCACGT
CAGCTGCCGGAAAATACCCTGCTGGTTACCTGTATTGCAAGCATTGGTAAAAATGCCATTCTGCGT
AAACAGGGTAGCTGTAATCAGCAGATTAATGCAGTTGTGCCGTTTGAGAACATCAACATCGATTAT
CTGTATTATATCAGCGATAGCCTGAGCACCTTCATGAAAAGCATTGCAGGTAAAACCGCAACCCAG
ATTGTGAACAAAAACACCTTTGAAAACCTGGAAATTTACCTGGCACCTTTTGAGGAACAGAACAAA
ATTGCAGATCTGATTAGCAGCCTGGAAGAACTGATTGAAAAACAGGCAAGCAAACTGATCAAAATG
AAAAGCCGCAAACAGGGCATGCTGCAGATTATGTTTATTCCCGGGGGATCCGATCGATC

CC80-1 TRD X

PGFEGEWEEKQFADFTKINQGLQIAINERKTEYSPELYFYITNEFLRPNSQTKYFIENPPQSVIAN
KEDILMTRTGNTGKVVTNVFGAFHNNFFKIKFDKNLYDRLFLVEVLNSSKIQNKILSLAGSSTIPD
LNHSDFYSISSSYPLLREQQKIGKFFSKLDRQIELEEQK

CC80-1 TRD Y

QIELEEQKLELLQQQKKGYMQKIFSQELRFKDENGNDYPDWEKKKLKEIACVYTGNTPSKKENIYW
NKGEYVWVTPTDINNSKNIYESENKLTQEGYKKARQLPENTLLVTCIASIGKNAILRKQGSCNQQI
NAVVPFENINIDYLYYISDSLSTFMKSIAGKTATQIVNKNTFENLEIYLAPFEEQNKIADLISSLE
ELIEKQASKLIKMKSRKQGMLQIMFI

CC80-2 TRD Z + CC72-2 TRD S

CCCGGGTTTGAAGGCGAATATTCTCTGGATATTTTTGGTAATCTGGCCACCAACAAAAGCGAAAAA
TTCAATCCGCAGAATGAAAACGCCAGCATTGATATTGAACTGGATTGCATTGAACAGAATACCGGT
CGTCTGATCAAAATCTATAACAGCAAAGAATTTAGCAGCCAGAAAAACAAATTTAACCCGCAGAAC
GTGCTGTATGGTAAACTGCGTCCGTATCTGAACAAATATTACTTCACCAAAAAAAGTGGTGTGTGC
AGCAGCGAAATTTGGGTTCTGAAAAGCACCAAAGAAGATAAACTGCTGAACCTGTTCCTGTACTAT
TTCATTCAGACCAAACGCTATAGTGATGTTGCAAGCAAAAGCGCAGGTAGCAAAATGCCTCGTGCA
GATTGGGGTCTGATTGAAAATATTCGTGTGTATTTTCCGGAACTGTGCGAACAGCAGAAAATTGGT
CAGTTTTTTAGCAAACTGGACCGTCAAATTGAATTAGAAGAACAGAAGCTGGAACTGCTGCAACAG
CAGAAAAAAGGTTATATGCAGAAAATCTTCAGCCAAGAGCTGCGCTTTAAAGATGAAAATGGTAAC
GATTATCCGGACTGGACCAATGAACGTCTGGGTGAAGTTACCACCGTTACCATGGGTCAGAGCCCG
AAAAGCGTGAATTATACCGATAATAGCAATGACACCGTTCTGATTCAGGGTAATGCCGATATTGAA
AACGGTCTGATTAATCCGCGTATCTATACCCGTGAAGTGACCAAACTGATTCAGAAAGATGAGATT
ATTCTGACCGTTCGTGCACCGGTTGGTAAACTGGCAATGGCACAGATTAATGCATGTATTGGTCGT
GGTGTTTGCAGCATTAAAGGCGATAAATTTCTGTATTATTTCCTGGAATGGTTCGCCACCCAGAAT
AAATGGATTCGTTTTAGCCAGGGTAGCACCTTTGAAAGCATTAGCGGTAATGATATTCGCAACATC
CATATCAAAATCCCGGTTGAAGATGAACGCACCAAAATTATCAAACTGCTGAATAGCCTGGATGTG
CTGAATTCAAAAACCGATCTGAAAATCCAGAATCTGAAACAGCGTAAACAGAGCCTGCTGCAAAAA
ATCTTTGTGCCCGGGGGATCCGATCGATC

CC80-2 TRD Z

PGFEGEYSLDIFGNLATNKSEKFNPQNENASIDIELDCIEQNTGRLIKIYNSKEFSSQKNKFNPQN
VLYGKLRPYLNKYYFTKKSGVCSSEIWVLKSTKEDKLLNLFLYYFIQTKRYSDVASKSAGSKMPRA
DWGLIENIRVYFPELCEQQKIGQFFSKLDRQIELEEQK

CC72-2 TRD S

QIELEEQKLELLQQQKKGYMQKIFSQELRFKDENGNDYPDWTNERLGEVTTVTMGQSPKSVNYTDN
SNDTVLIQGNADIENGLINPRIYTREVTKLIQKDEIILTVRAPVGKLAMAQINACIGRGVCSIKGD
KFLYYFLEWFATQNKWIRFSQGSTFESISGNDIRNIHIKIPVEDERTKIIKLLNSLDVLNSKTDLK
IQNLKQRKQSLLQKIFV

CC93-2 TRD b*

CCCGGGTTTGAAGGCGAATGGGAGGAGAAAAAACTGGAAGATACCCTGGAATTCATTAAAGATGGC
ACCCATGGTACACATGAAAATGTTAATAATGGTCCGTGGCTGCTGAGCGCCAAAAACATTAAAAAC
AACAAAATCATCATCAGCAGCGACGATCGCAAAATTAGCGAAAGCGATTACAAAAAAATCTACAAA
AACTATAAACTGGAAAAAGGCGATCTGCTGCTGACCATTGTTGGCACCATTGGTCGTGCAGCAATT
GTTAAAAATCCGAACAATATTGCCTTTCAGCGTAGCGTTGCAATCCTGAAAACCAAAGCAACCTAT
GATGTGGGCTTTATCTTTCAGCTGTTCCAGACCAAATACTTTAAAAACCTGCTGCTGCGTAAACAG
GTTGTTAGCGCACAGCCTGGTCTGTATCTGGGTGATATTCGTAAAATCAAAATCAGCATTACCAAC
ATCATCGAAGAACAGCGCAAAATCGGTATCTTTTTCAGCAAACTGGATCGTCAAATTGAATTAGAA
GAACAGAAG

CC93-2 TRD b*

PGFEGEWEEKKLEDTLEFIKDGTHGTHENVNNGPWLLSAKNIKNNKIIISSDDRKISESDYKKIYK
NYKLEKGDLLLTIVGTIGRAAIVKNPNNIAFQRSVAILKTKATYDVGFIFQLFQTKYFKNLLLRKQ
VVSAQPGLYLGDIRKIKISITNIIEEQRKIGIFFSKLDRQIELEEQK

C93-3 TRD a*

CAAATTGAATTAGAAGAACAGAAGCTGGAACTGCTGCAACAGCAGAAAAAAGGTTATATGCAGAAA
ATCTTCAGCCAAGAGCTGCGCTTTAAAGATGAAAATGGTAACGATTATCCGGAATGGGAAAACAAA
CGCATTGAAGATATTGCCAATGTGAACAAAGGTTTTACCCCGAGCACCAACAATAACGAATATTGG
GATAACAACGATAAAAACTGGCTGAGCATTGCAGGCATGAATCAGAAATATCTGTATAAAGGCAAC
AAAGGCATCAGCAAAGATGCAGCCAAAAACTATATGAAAGTGAAAAACGACACCCCTGATCATGTCC
TTTAAACTGACCATTGGTAAACTGGCGATTGTTAAAGCACCGCTGTATACCAATGAAGCCATTTGT
CATTTTATCTGGAAAGTGAACAAAATCAACACCGAGTTCATCTACTATTACCTGAACAGCCTGAAC
ATTAGCACCTTTGGTGTTCAGGCAGTTAAAGGTGTTACCCTGAATAACGATAGCATCAACAGCATT

ATTGTGAAACTGCCGAATGAAGAGGAACAGAACATTATCGCAAAATTTCTGCTGGAAGTGGACAAA
ACCGTTAATAATCAGCTGGTGAAAACCAAACTGCTGAAACAACGTAAAAAAGGCCTGCTGCAGCGT
ATGTTTGTTCCCGGGGGATCCGATCGATC

CC93-3 TRD a*

QIELEEQKLELLQQQKKGYMQKIFSQELRFKDENGNDYPEWENKRIEDIANVNKGFTPSTNNNEYW
DNNDKNWLSIAGMNQKYLYKGNKGISKDAAKNYMKVKNDTLIMSFKLTIGKLAIVKAPLYTNEAIC
HFIWKVNKINTEFIYYYLNSLNISTFGVQAVKGVTLNNDSINSIIVKLPNEEEQNIIAKFLLEVDK
TVNNQLVKTKLLKQRKKGLLQRMFV

CC873 TRD e* + CC97 TRD c*

CCCGGGTTTGAAGGCGAATGGGAGGAAAAATCGATCAGCAGCTTTCTGAAAGAAAGCAAAATCAAA
GGTAGCAATGGTAGCCATGCAAAAAAACTGACCGTTAAACTGTGGGGTAAAGGTGTTGTTCCGAAA
AAAGAAACGTTTAAAGGCAGCGATAACACCCAGTATTACAAACGTAAAGCAGGTCAGCTGATGTAT
GGCAAACTGGATTTTCTGAATTGCGCCTTTGGTATTGTTCCGGATAGCCTGAATAACTATGAAAGC
ACCATTGATAGCCCGAGCTTTGATTTCATTAATGGCGATAGCAAATTTCTGCTGGAACGCATTAAA
CTGAAAAGCTTCTACAAAAAATTCGGCGATATTGCAAATGGCAGCCGTAAAGCAAAACGTATTAAT
CAGGATACCTTTCTGAGCCTGCCGGTTTTTGCACCGAAATATGATGAACAGCTGCGTATTGGTGAA
TTTTTCAGTAAACTGGATCGTCAAATTGAATTAGAAGAACAGAAGCTGGAACTGCTGCAACAGCAG
AAAAAAGGTTATCTGCAGAAAATCTTTAGCCAAGAGCTGCGCTTTAAAGATGAAAACGGTAATGAT
TATCCGGAATGGCGTTTTGCCCGTTTCAAAGATTTTATGTACAAACCGATTAATATCCGTCCGGCA
ATCAACATTAGCAAAAGCGAACTGCTGACCGTTAAACTGCATTGCAAAGGTATTGAAAAAGCCAAC
ATTAACCGTGTGCTGAAACTGGGTGCAACCAATTATTACAAACGTTTTGAAGGCCAGTTTATCTAT
GGCAAACAGAACTTTTTTAACGGTGCCTTTGATATCGTGCCGAAAAAATTCGATGGTCTGTATAGC
AGCAGTGATGTTCCGGCATTTGAAATCAATACCGAGAAAATTGAGCCGAACTACTTCATCAGCTAT
ATTAGCCGTCCGAGCTTCTATAAAAGCAAAGAGAAATATAGCACCGGCACCGGTAGCAAACGTATT
CATGAAAATACCGTGCTGAACTTTAGCCTGCATCTGCCGTGTCTGAATGAACAGCTGAAAATTGCA
AGCTTTGTGTGCTTTCTGAACCGTAAAATTGAACTGCTGGAACGCAAAATCTATCTGATCAAAAAA
CAGAAACAGGCCCTGCTGCAGCAAATGTTTATTCCCGGGGGATCCGATCGATC

CC873 TRD e*

PGFEGEWEEKSISSFLKESKIKGSNGSHAKKLTVKLWGKGVVPKKETFKGSDNTQYYKRKAGQLMY
GKLDFLNCAFGIVPDSLNNYESTIDSPSFDFINGDSKFLLERIKLKSFYKKFGDIANGSRKAKRIN
QDTFLSLPVFAPKYDEQLRIGEFFSKLDRQIELEEQK

CC97 TRD c*

QIELEEQKLELLQQQKKGYLQKIFSQELRFKDENGNDYPEWRFARFKDFMYKPINIRPAINISKSE
LLTVKLHCKGIEKANINRVLKLGATNYYKRFEGQFIYGKQNFFNGAFDIVPKKFDGLYSSSDVPAF
EINTEKIEPNYFISYISRPSFYKSKEKYSTGTGSKRIHENTVLNFSLHLPCLNEQLKIASFVCFLN
RKIELLERKIYLIKKQKQALLQQMFI

CC133-2 from ED133 TRD d*

CAAATTGAATTAGAAGAACAGAAGCTGGAACTGCTGCAACAGCAGAAAAAAGGTTATATGCAGAAA
ATCTTCAGCCAAGAGCTGCGCTTTAAAGATGAAAATGGTAACGATTATCCGGAATGGGAAAATGTG
ATGCTGCAGAAAGTTCTGAAAGATAAAACCGAAGGTATTAAACGTGGTCCGTTTGGTGGTGCACTG
AAAAAAGATATTTTTGTGGAAAGCGGCTATGCCGTTTATGAACAGCGTAATGCCATTTATGATATC
AGCAACTTCCGCTACTATATCAACGAGAACAAATACAAAGAGATGCAGAGCTTTAGCGTTCAGCCG
AATGATATTATCATGAGCTGTAGCGGCACCATTGGTCGTCTGGCACTGATTCCGCATAACTATACC
AAAGGTATTATCAACCAGGCCCTGATTCGTTTTCGTACCAATCATAAAATCCGCAGCGAATTCTTT
CTGATCTTTATGCGTAGCAATCAGATGCAGCGTAAAATTCTGGAAGCAAATCCGGGTAGCGCAATT
ACCAATCTGGTTCCGGTTAAAGAACTGAAACTGATCCCGTTTCCGCTGCCGGTTAAATTTGAACAG
GATAAAATCAGCCAGTTCATCCACATTATTAACCGTCGTATTGAACAGAGCGAGAAAAAAATCGAA
AGCCTGAAAAATCGCAAACAGGGTTTCCTGCAGAAACTGTTTGTTCCCGGGGGATCCGATCGATC

CC133-2 from ED133 TRD d*

QIELEEQKLELLQQQKKGYMQKIFSQELRFKDENGNDYPEWENVMLQKVLKDKTEGIKRGPFGGAL
KKDIFVESGYAVYEQRNAIYDISNFRYYINENKYKEMQSFSVQPNDIIMSCSGTIGRLALIPHNYT

KGIINQALIRFRTNHKIRSEFFLIFMRSNQMQRKILEANPGSAITNLVPVKELKLIPFPLPVKFEQ
DKISQFIHIINRRIEQSEKKIESLKNRKQGFLQKLFV

ST80-3 TRD X + TRD f*

CCCGGGTTTGAAGGCGAATGGGAGGAAAAACAGTTTGCCGATTTTACCAAAATTAACCAGGGTCTG
CAGATTGCCATTAATGAACGTAAAACCGAATATAGCCCTGAGCTGTATTTCTATATCACCAACGAA
TTTCTGCGTCCGAATAGCCAGACCAAATATTTCATTGAAAATCCGCCTCAGAGCGTGATTGCCAAC
AAAGAAGATATTCTGATGACCCGCACCGGTAATACCGGCAAAGTTGTTACCAATGTTTTTGGTGCC
TTCCACAACAACTTTTTCAAAATCAAATTCGATAAAAACCTGTATGATCGCCTGTTTCTGGTTGAA
GTTCTGAACAGCAGCAAAATCCAGAACAAAATTCTGAGCCTGGCAGGTAGCAGCACCATTCCGGAT
CTGAATCATAGCGATTTCTATAGCATTAGCAGCAGCTATCCGCTGCTGCGCGAACAGCAAAAAATT
GGCAAATTCTTTAGCAAACTGGATCGCCAGATTGAACTGGAAGAACAGAAACTGGAACTGCTGCAA
CAGCAGAAAAAAGGCTATATGCAGAAAATCTTTAGCCAAGAGCTGCGCTTTAAAGATGAAAACGGT
GAAGATTATCCGGATTGGAAAGAAAAAAAACTGGGCGATATTACCGAGCAGAGCATGTATGGTATT
GGTGCAAGCGCAACCGTTTTGATAGCAAAAATATCTATATCCGCATCACCGACATCGATGAAAAAA
GCCGTAAACTGAATTATCAGAATCTGACCACACCGGATGAACTGAACAATAAATACAAACTGAAAC
GCAACGACATCCTGTTTGCACGTACCGGTGCAAGTACCGGTAAAAGCTATATTCATAAAGAAGAGA
AAGACATCTACAACTACTACTTTGCGGGTTTTCTGATCAAATTCAAAATTAACGAACAGAACAGTC
CGCTGTTCATCTATCAGTTTACCCTGACCAGCAAATTCAACAAATGGGTTAAAGTTATGAGCGTGC
GTAGCGGTCAGCCTGGTATTAATAGCGAAGAATATGCAAAACTGCCGCTGGTTCTGCCGAATAAAC
TGGAACAACAAAAAATCGCGAAATTCCTGGATCGTTTTGATCGTCAGATCGAGCTGGAAAAACAAA
AAATTGAAATTCTGCAGCAACAAAAAAAAGGCCTGCTGCAGAGTATGTTTATTCCCGGGGGATCCG
ATCGATC

ST80-3 TRD X

MSNTQKKNVPELRFPGFEGEWEEKQFADFTKINQGLQIAINERKTEYSPELYFYITNEFLRPNSQT
KYFIENPPQSVIANKEDILMTRTGNTGKVVTNVFGAFHNNFFKIKFDKNLYDRLFLVEVLNSSKIQ
NKILSLAGSSTIPDLNHSDFYSISSSYPLLREQQKIGKFFSKLDR

ST80-3 TRD f*

QIELEEQKLELLQQQKKGYMQKIFSQELRFKDENGEDYPDWKEKKLGDITEQSMYGIGASATRFDS
KNIYIRITDIDEKSRKLNYQNLTTPDELNNKYKLKRNDILFARTGASTGKSYIHKEEKDIYNYYFA
GFLIKFKINEQNSPLFIYQFTLTSKFNKWVKVMSVRSGQPGINSEEYAKLPLVLPNKLEQQKIAKF
LDRFDRQIELEKQKIEILQQQKKGLLQSMFIPGGSHHHHHH

SUPPLEMENTARY INFORMATION FOR TABLE 2.

S.SauCD-EGFP

CC30-1   GW<u>A</u>G-5-GA<u>T</u>

This MTase was expressed and purified as a fusion with EGFP.

MSNTQTKNVPELRFPGFEGEWEEKKVGELLEFKNGLNKGKEYFGSGSSIVNFKDVFNNRSLNTNNL
TGKVNVNSKELKNYSVEKGDVFFTRTSEVIGEIGYPSVILNDPENTVFSGFVLRGRPKSGIDLINN
NFKRYVFFTNSFRKEMITKSSMTTRALTSGSAINKMKVIYPVSAKEQRKIGDFFSKLDRQIELEEQ
KLELLQQQKKGYMQKIFSQELRFKDENSEDYPHWENSKIEKYLKERNERSDKGQMLSVTINSGIIK
FSELDRKDNSSKDKSNYKVVRKNDIAYNSMRMWQGASGRSNYNGIVSPAYTVLYPTQNTSSLFIGY
KFKTHRMIHKFKINSQGLTSDTWNLKYKQLKNINIDIPVLEEQEKIGDFFKKMDILISKQKIKIEI
LEKEKQSFLQKMFLGSMVSKGEELFTGVVPILVELDGDVNGHKFSVSGEGEGDATYGKLTLKFICT
TGKLPVPWPTLVTTLTYGVQCFSRYPDHMKQHDFFKSAMPEGYVQERTIFFKDDGNYKTRAEVKFE
GDTLVNRIELKGIDFKEDGNILGHKLEYNYNSHNVYIMADKQKNGIKVNFKIRHNIEDGSVQLADH
YQQNTPIGDGPVLLPDNHYLSTQSALSKDPNEKRDHMVLLEFVTAAGITLGMDELYKHHHHHH

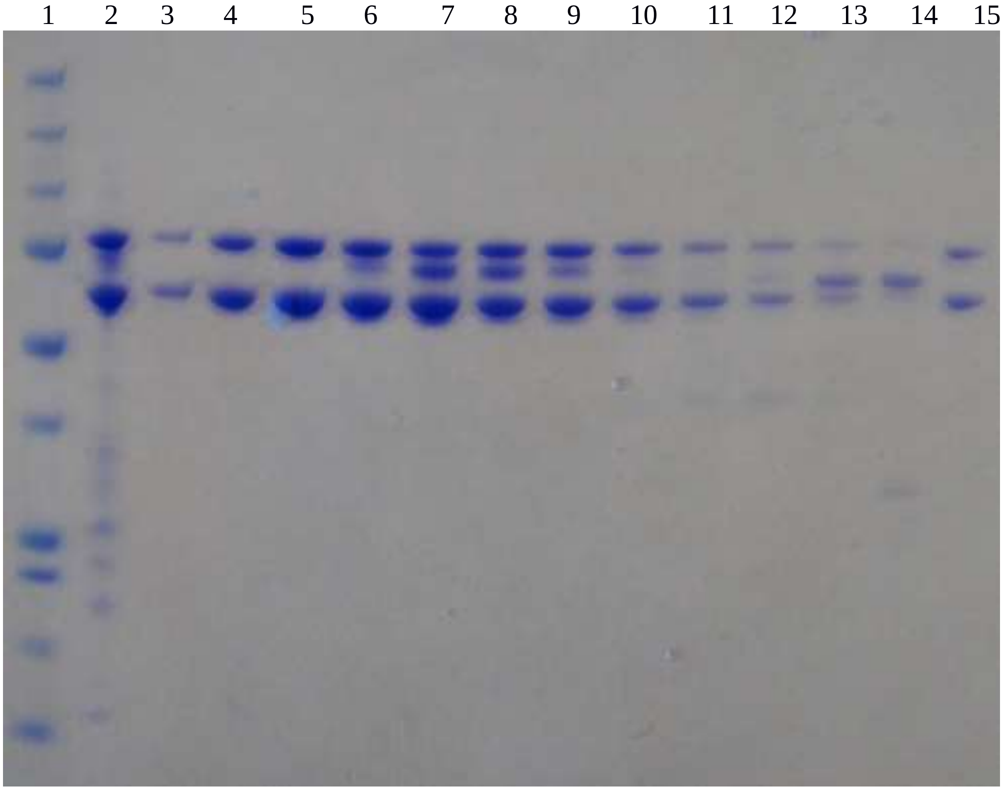

1- marker   2- Nickel column eluate   3-14   Fractions from gel filtration column
15- CC5-1 Purified protein marker

Nuclease assay on the plasmid library.

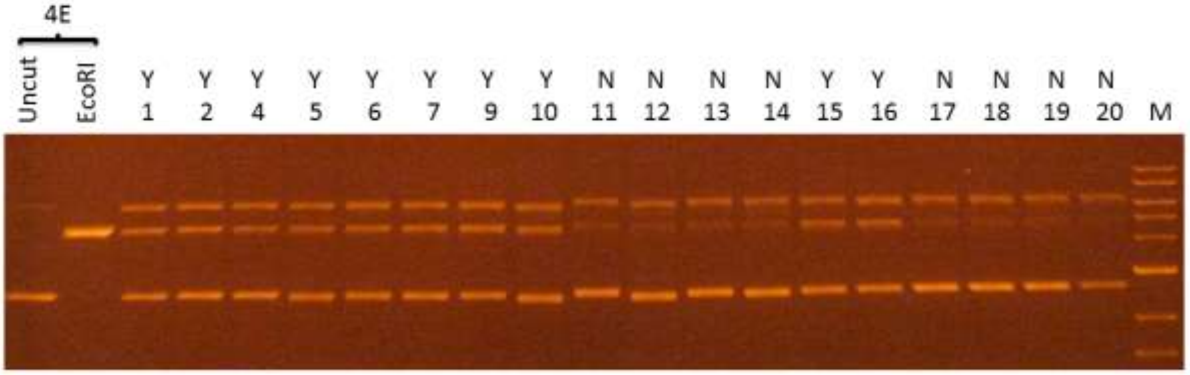

S.SauCD-EGFP
CC30-1   GWAG-5-GAT

SMRT did not work for the CC30-1 system when looking for methylation of genomic DNA from *E. coli*.

Motif	Modified Position	Modification Type	% Motifs Detected	# Of Motifs Detected	# Of Motifs In Genome	Mean Modification QV	Mean Motif Coverage	Partner Motif
BNNGGTAMH	4	unknown	7.05	780	11058	37.7	88.6	
ANNNNNNDNNNH	1	unknown	0.11	1312	1235059	36.0	100.7	
Not Clustered	0		0.19	14583	7880091	36.1	107.4	

Modification QV Histogram By Motif

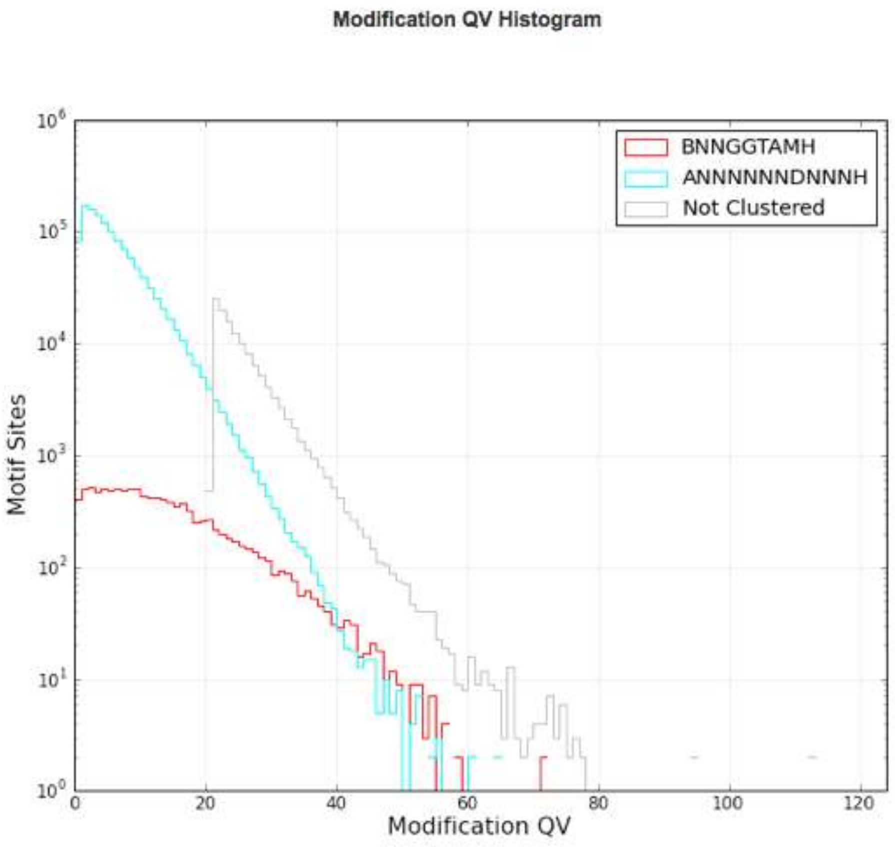

S.SauJK-EGFP
CC30-2   GGA-7-TCG
This MTase was a fusion with EGFP.
MSNTQKKNVPELRFPEFEGEWEERKLGDLIKVNSGKDYKHLDKGDIPVYGTGGYMTSVSEPLSEID
AVGIGRKGTINKPYLLEAPFWTVDTLFYCTPEKEADILFILSLFRKINWKLYDESTGVPSLSKQTI
NKINRLVPTNKEQQKIGEFFSKLDRQIELEEQKLELLQQQKKGYMQKIFSQELRFKDENGNDYPKW
EEKKIEDIASQVYGGGTPNTKIKEFWNGDIPWIQSSDVKVNDLILQQCNKFISKNSIELSSAKLIP
ANSIAIVTRVGVGKLCLVEFDYATSQDFLSLSSLKYDKLYSLYSLLYTMKKISANLQGTSIKGITK
KELLDSIIKIPHNLEEQQKIGDLFYKIDKYISFNKCKIEMLKSLKQGLLKKMFIGSMVSKGEELFT
GVVPILVELDGDVNGHKFSVSGEGEGDATYGKLTLKFICTTGKLPVPWPTLVTTLTYGVQCFSRYP
DHMKQHDFFKSAMPEGYVQERTIFFKDDGNYKTRAEVKFEGDTLVNRIELKGIDFKEDGNILGHKL
EYNYNSHNVYIMADKQKNGIKVNFKIRHNIEDGSVQLADHYQQNTPIGDGPVLLPDNHYLSTQSAL
SKDPNEKRDHMVLLEFVTAAGITLGMDELYKHHHHHH

This system could not be expressed so was used for SMRT sequencing only.
SMRT analysis of genomic DNA from *E. coli*.

Motif	Modified Position	Modification Type	% Motifs Detected	# Of Motifs Detected	# Of Motifs In Genome	Mean Modification QV	Mean Motif Coverage	Partner Motif
CGANNNNNNNTCC	3	m6A	98.76	1439	1457	89.3	76.9	GGANNNNNNNTCG
GGANNNNNNNTCG	3	m6A	98.56	1436	1457	91.2	76.8	CGANNNNNNNTCC
Not Clustered	0		0.09	8260	9123294	35.7	87.7	

Modification QV Histogram By Motif

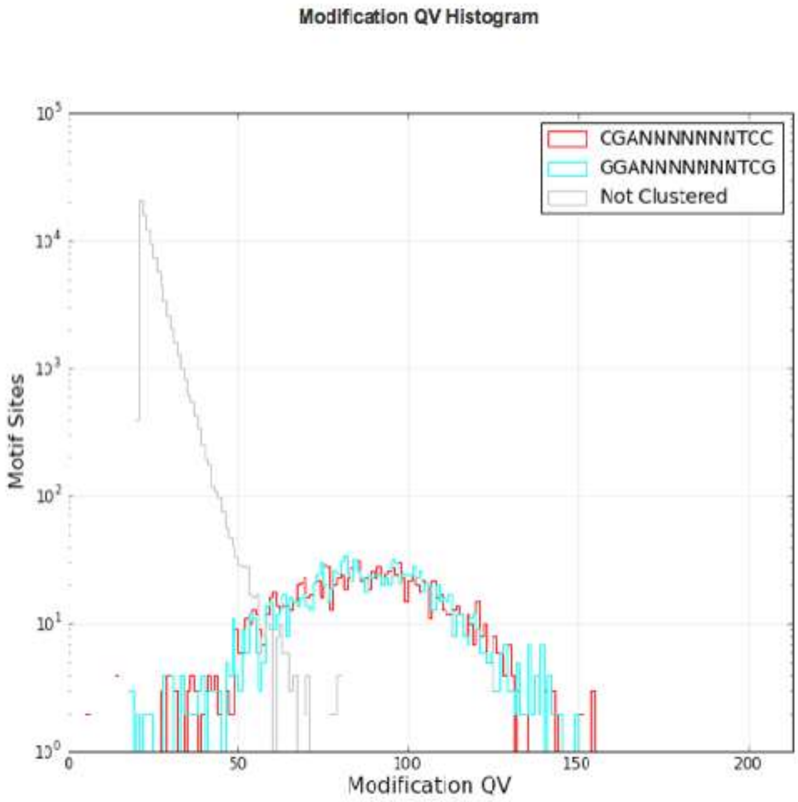

S.SauJd*

CC133-2 from ED133 GGA-7-TTRG

This enzyme was studied using the SMRT assay. There are minor variations in S subunit sequence in CC133-2.

Recombinant S.SauJd* CC133-2

MSNTQKKNVPELRFPGFEGEWEEKKLGDLIKVNSGKDYKHLEKGDIPVYGTGGYMTSVSEPLSEID
AVGIGRKGTINKPYLLEAPFWTVDTLFYCTPKKETDILFILSLFRKINWKVYDESTGVPSLSKQTI
NKINRFVPSNKEQQKIGEFFIKLDRQIELEEQKLELLQQQKKGYMQKIFSQELRFKDENGNDYPEW
ENVMLQKVLKDKTEGIKRGPFGGALKKDIFVESGYAVYEQRNAIYDISNFRYYINENKYKEMQSFS
VQPNDIIMSCSGTIGRLALIPHNYTKGIINQALIRFRTNHKIRSEFFLIFMRSNQMQRKILEANPG
SAITNLVPVKELKLIPFPLPVKFEQDKISQFIHIINRRIEQSEKKIESLKNRKQGFLQKLFVPGGS
HHHHHH

Wild type S.SauJd*

MSNTQKKNVPELRFPGFEGEWEEKKLESIIKVNSGKDYKHLDKGDIPVYGTGGYMTSVSEPLSEID
AVGIGRKGTINKPYLLEAPFWTVDTLFYCTPKKETDILFILSLFRKINWKVYDESTGVPSLSKQTI
NKINRFVPTNKEQQKIGKFFSKLDRQIELQEQKLELLQQQKKGYMQKIFSQELRFKDENGNDYPEW
ENVMLQKVLKDKTEGIKRGPFGGALKKDIFVESGYAVYEQRNAIYDISNFRYYINENKYKEMQSFS
VQPNDIIMSCSGTIGRLALIPHNYTKGIINQALIRFRTNHKIRSEFFLIFMRSNQMQRKILEANPG
SAITNLVPVKELKLIPFPLPVKFEQDKISQFIHIINRRIEQSEKKIESLKNRKQGFLQKLFV*

Reports for Job Dryden_J_delta_MODs PACIFIC BIOSCIENCES

SMRT Cells: 1 Movies: 1

Motif Summary

Motifs	Modified Position	Type	% Motifs Detected	# Of Motifs Detected	# Of Motifs In Genome	Mean Modification QV	Mean Motif Coverage	Partner Motif
CYAANNNNNNNTCC	4	m6A	90.36%	422	467	51.19	30.33	
GGANNNNNNNHTRG	3	m6A	47.67%	1114	2337	45.72	32.24	
CYAAYBNNNNNMCC	4	m6A	25.68%	169	658	42.89	34.51	
CCBANTNNNNNTCC	4	m6A	20.39%	42	206	44.40	32.14	GGANNNNNANTVGG
GGANNNNNANTVGG	3	m6A	18.45%	38	206	44.76	31.37	CCBANTNNNNNTCC

Modification QVs

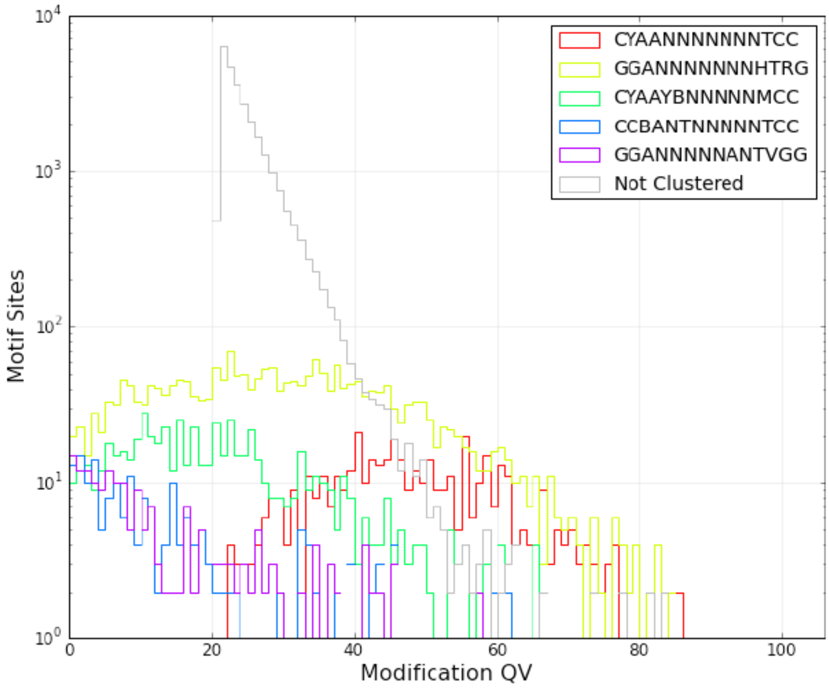

S.SauNE

CC398-1   ACC-5-RTGA

The clone obtained contained a single amino acid substitution A50S which did not affect activity. The enzyme was expressed using plasmid pSauNE-XmaI.

S.SauNEXmaI "Expected" sequence

MSNTQKKNVPELRFPGFEGEWEEKKLGEFAGKVTQKNVDKKYIETLTNS**A**ELGIISQKDYFDKEIS
NIDNIKKYYVVEENDFVYNPRMSNYAPFGPVNRNKLGKKGVMSPLYTVFKIQNIDLNFIEFYFKSS
KWYRFMALNGDSGARADRFSIKDRTFMEMPLHIPCMDEQIKIGQFFSKLDRQIELEEQKLELLQQQ
KKGYMQKIFSQELRFKDENGKDYPEWEETTIKEIAQINTGKKDTKDAITNGSYDFYVRSPIVYKIN
TFSYEGEAILTVGDGVGVGKVFHYVNGKFDYHQRVYKISDFKNYYGLLLFYYFSQNFLKETKKYSA
KTSVDSVRKDMIANMKVPRPIYIEQKKIGQFIKRVDNKTKIQKQVIELLKQRKKSLLQKMFIPGGS
HHHHHH*

S.SauNEXmaI "Actual" sequence

MSNTQKKNVPELRFPGFEGEWEEKKLGEFAGKVTQKNVDKKYIETLTNS**S**ELGIISQKDYFDKEIS
NIDNIKKYYVVEENDFVYNPRMSNYAPFGPVNRNKLGKKGVMSPLYTVFKIQNIDLNFIEFYFKSS
KWYRFMALNGDSGARADRFSIKDRTFMEMPLHIPCMDEQIKIGQFFSKLDRQIELEEQKLELLQQQ
KKGYMQKIFSQELRFKDENGKDYPEWEETTIKEIAQINTGKKDTKDAITNGSYDFYVRSPIVYKIN
TFSYEGEAILTVGDGVGVGKVFHYVNGKFDYHQRVYKISDFKNYYGLLLFYYFSQNFLKETKKYSA
KTSVDSVRKDMIANMKVPRPIYIEQKKIGQFIKRVDNKTKIQKQVIELLKQRKKSLLQKMFIPGGS
HHHHHH*

1 2 3 4 5 6 7 8 9 10 11 12 13 14 15

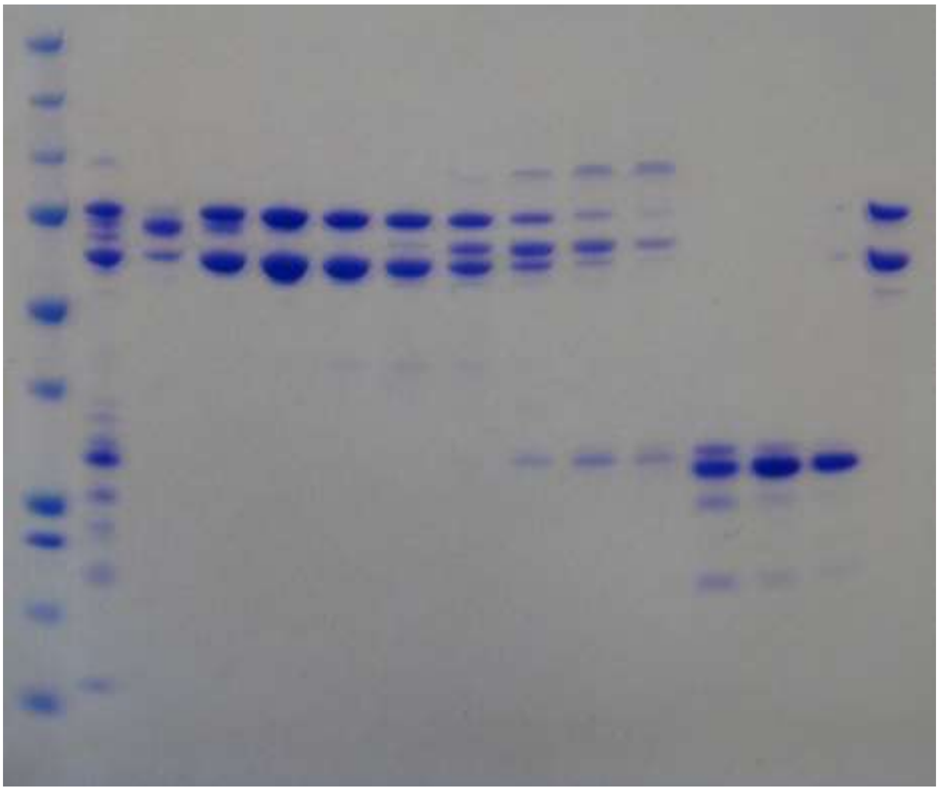

1- marker 2- Nickel column eluate 3-14 Fractions from gel filtration column
15- CC5-1 purified protein marker

S.SauNE
CC398-1 ACC-5-RTGA

Reports for Job Ed_1_Dryden_MODs

SMRT Cells: 1 Movies: 1

Motif Summary

Motifs	Modified Position	Type	% Motifs Detected	# Of Motifs Detected	# Of Motifs In Genome	Mean Modification QV	Mean Motif Coverage	Partner Motif
ACCNNNNNRTGA	1	m6A	99.69%	971	974	89.04	57.17	TCAYNNNNNGGT
TCAYNNNNNGGT	3	m6A	99.69%	971	974	90.00	57.86	ACCNNNNNRTGA
ACCNNNNHRTGGB	1	m6A	49.07%	291	593	54.17	60.71	VCCAYDNNNNGGT
VCCAYDNNNNGGT	4	m6A	45.36%	269	593	54.62	61.85	ACCNNNNHRTGGB
ACCNNNNHRAGA	1	m6A	41.75%	200	479	48.38	61.76	
HTCARNNNNNGGTNV	4	m6A	36.31%	264	727	51.22	62.33	
ACCNNNNNYTGAD	1	m6A	34.9%	320	917	49.93	60.88	

Modification QVs

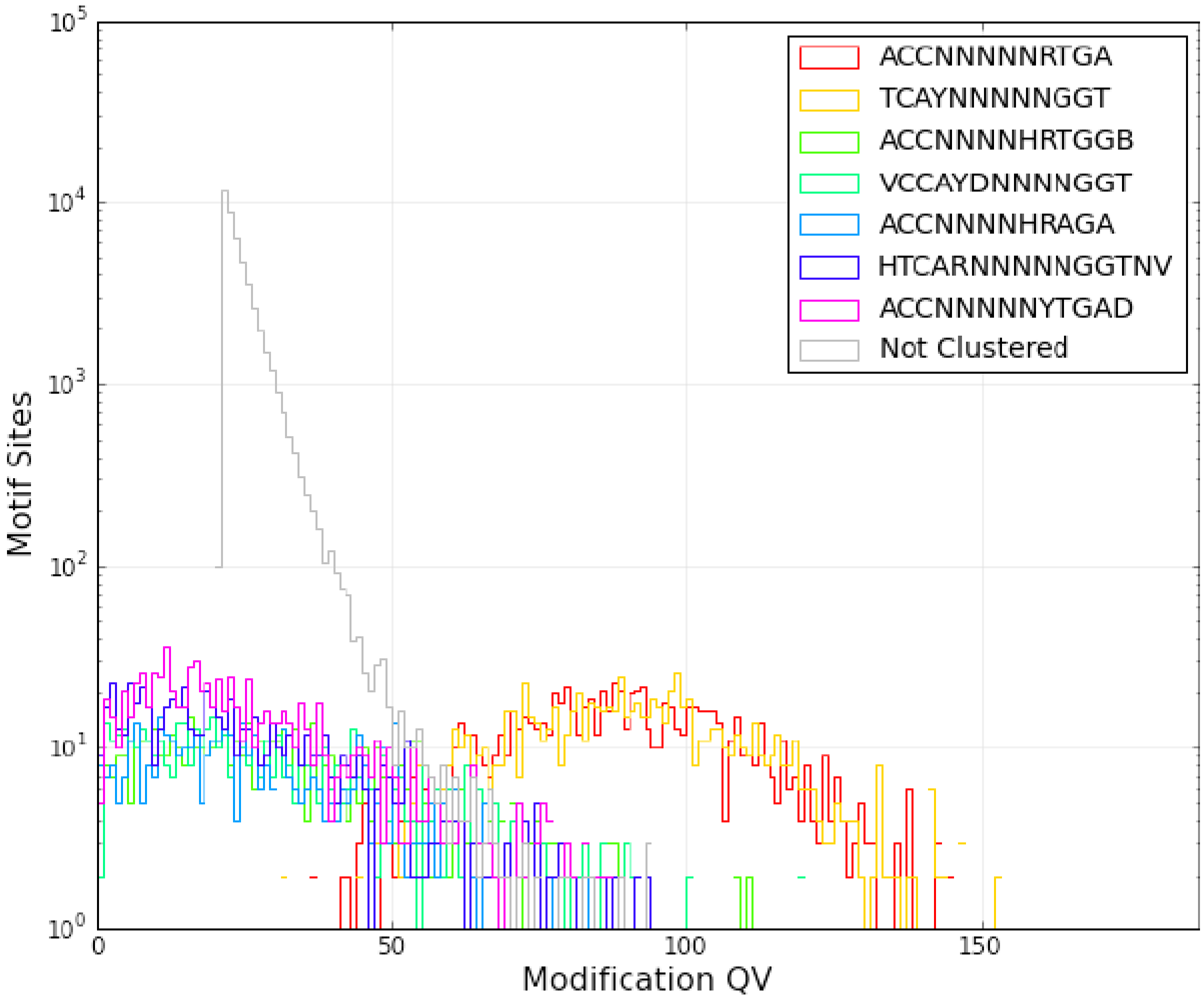

SUPPLEMENTARY INFORMATION FOR TABLE 3.

S.SauBE   AGG-5-RTGA

This MTase was purified but cut all the plasmids in the nuclease assay. Therefore once the targets for each TRD had been determined from other MTases, we used the ATPase assay to verify the length of the non-specific spacer.

MSNTQKKNVPELRFPGFEGEWEEKKLGDLTDRVIRKNKNLESKKPLTISGQLGLIDQTEYFSKSVS
SKNLENYTLIKNGEFAYNKSYSNGYPLGAIKRLTRYDSGVLSSLYICFSIKSEMSKDFMEAYFDST
HWYREVSGIAVEGARNHGLLNVSVNDFFTILIKYPSLEEQQKIGKFFSKLDRQIELEEQKLELLQQ
QKKGYMQKIFSQELRFKDENGKDYPEWEETTIKEIAQINTGKKDTKDAITNGSYDFYVRSPIVYKI
NTFSYEGEAILTVGDGVGVGKVFHYVNGKFDYHQRVYKISDFKNYYGLLLFYYFSQNFLKETKKYS
AKTSVDSVRKDMIANMKVPRPIYIEQKKIGQFIKRVDNKTKIQKQVIELLKQRKKSLLQKMFIPGG
SHHHHHH

1 2 3 4 5 6 7 8 9 10 11 12 13 14 15

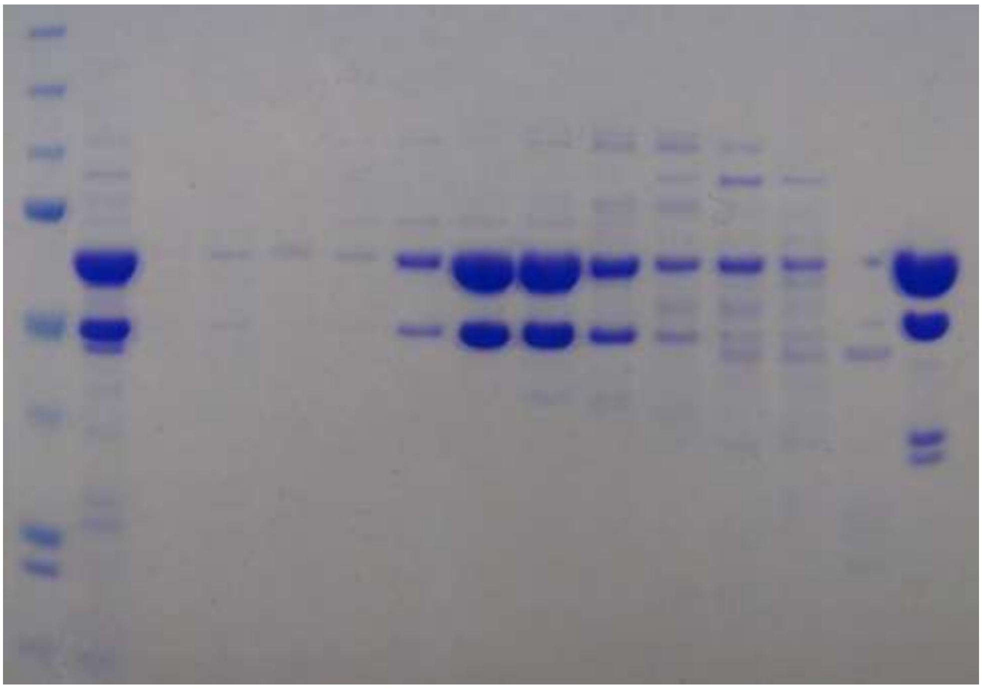

1- marker 2- Nickel column eluate 3-14 Fractions from gel filtration column
15- CC398-1 purified protein marker

Oligonucleotides for checking BE target site using ATPase assay.

Underlined refers to methylated bases.

5’-AGG-N-RTGA-3’

3’-TCC-N-YACT -5’

N values may be 4-6 i.e., number of base pairs between methylated adenines of 7-9. However, DNA digests show that pUC19 contains the site. This rules out the possibility of N=4 (i.e., no site in pUC19 for N value of 4). Therefore we checked for N5 and N6 only.

Oligonucleotide name	DNA sequence (5' to 3')
BE5for	AGATGATGGAATCAATGCAGGTTCCAGTGAGCCCTATACGATATAA
BE5rev	TTATATCGTATAGGGCTCACTGGAACCTGCATTGATTCCATCATCT
BE6for	AGATGATGGAATCAATGCAGGTTCACAGTGAGCCCTATACGATATAA
BE6rev	TTATATCGTATAGGGCTCACTGTGAACCTGCATTGATTCCATCATCT

S.SauBE   AGG-5-RTGA
N=5 gives the most activity therefore we **c**onclude from the ATPase assay that the site for the BE TRD combination is AGG-5-RTGA.

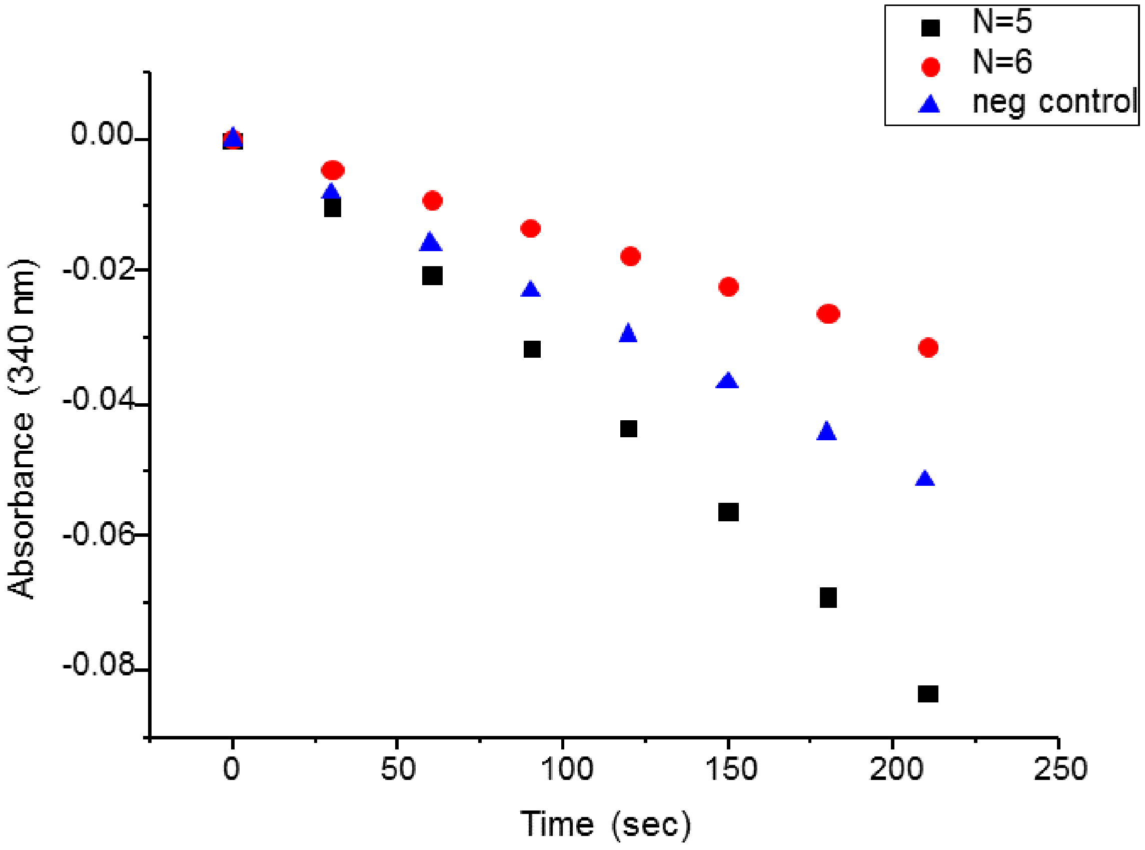

S.SauJE GGA-6-RTGA

This MTase was used in both nuclease and SMRT assays. The TRD pair JE occurs in other ST groups namely ST49 and ST50.

MSNTQKKNVPELRFPGFEGEWEEKKLGDLIKVNSGKDYKHLEKGDIPVYGTGGYMTSVSEPLSEID
AVGIGRKGTINKPYLLEAPFWTVDTLFYCTPKKETDILFILSLFRKINWKVYDESTGVPSLSKQTI
NKINRFVPSNKEQQKIGEFFIKLDRQIELEEQKLELLQQQKKGYMQKIFSQELRFKDENGKDYPEW
EETTIKEIAQINTGKKDTKDAITNGSYDFYVRSPIVYKINTFSYEGEAILTVGDGVGVGKVFHYVN
GKFDYHQRVYKISDFKNYYGLLLFYYFSQNFLKETKKYSAKTSVDSVRKDMIANMKVPRPIYIEQK
KIGQFIKRVDNKTKIQKQVIELLKQRKKSLLQKMFIPGGSHHHHHH

1 2 3 4 5 6 7 8 9 10 11 12 13 14 15

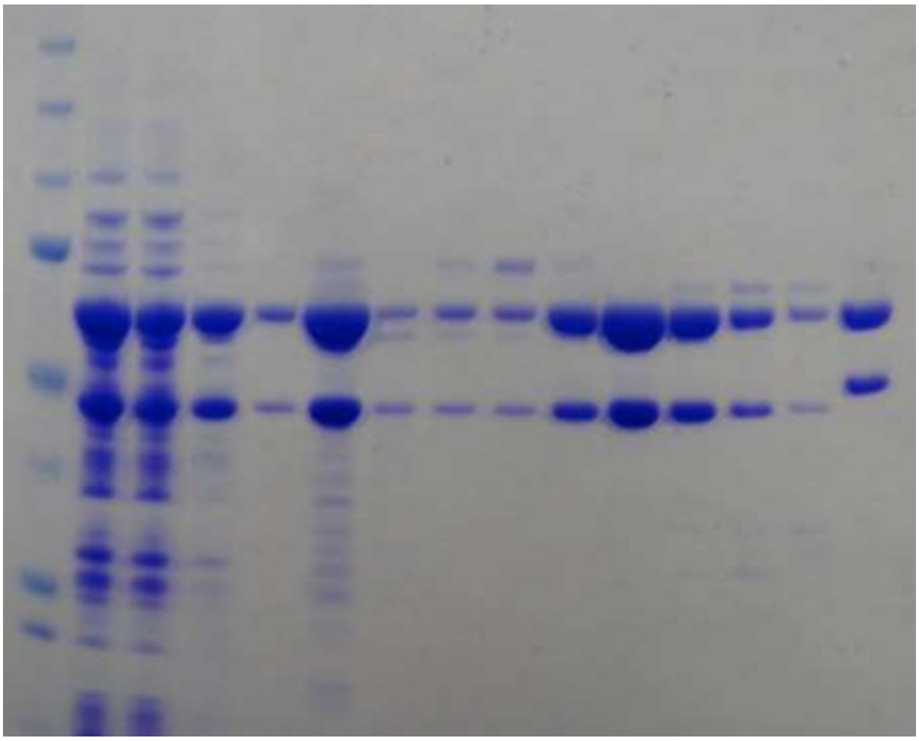

1- marker 2- soluble cell extract 3- Nickel column flow through 4- Nickel column wash 1
5- Nickel column wash 2 6- Nickel column eluate 7-14 Fractions from gel filtration column
15- CC398-1 purified protein marker

Possible site: GGANNNNNNRTGA Note that the background linearisation may be due to the enzyme displaying star activity against a similar site (i.e., a single GGAN7RTGA site is found in pUC19) to the real site (GGAN6RTGA). Repeated digests generate an identical pattern of digestion.

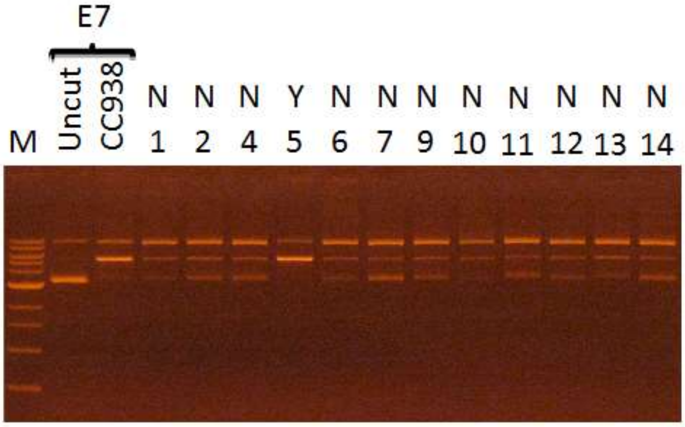

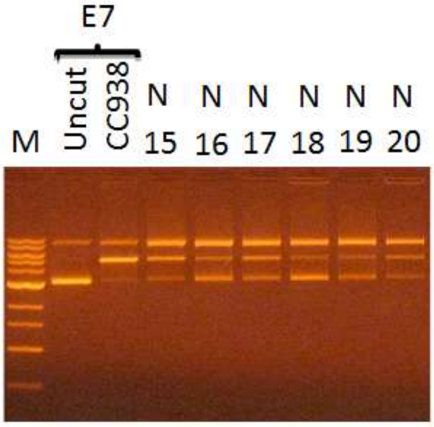

S.SauJE GGA-6-RTGA

SMRT data showed only the N=6 spacer giving modification.

SMRT Cells: 2 Movies: 2 Print Download Email

Motif	Modified Position	Modification Type	% Motifs Detected	# Of Motifs Detected	# Of Motifs In Genome	Mean Modification QV	Mean Motif Coverage	Partner Motif
TCAYNNNNNNTCC	3	m6A	31.44	305	970	38.3	17.1	GGANNNNNNRTGA
GGANNNNNNRTGA	3	m6A	24.43	237	970	38.3	17.5	TCAYNNNNNNTCC
Not Clustered	0		0.00	324	9,124,268	34.2	15.8	

S.SauNI ACC-6-TGAR

MSNTQKKNVPELRFPGFEGEWEEKKLGEFAGKVTQKNVDKKYIETLTNSAELGIISQKDYFDKEIS
NIDNIKKYYVVEENDFVYNPRMSNYAPFGPVNRNKLGKKGVMSPLYTVFKIQNIDLNFIEFYFKSS
KWYRFMALNGDSGARADRFSIKDRTFMEMPLHIPCMDEQIKIGQFFSKLDRQIELEEQKLELLQQQ
KKGYMQKIFSQELRFKNENGNDYPDWERIKFFDVIDKVIDFRGRTPKKLNMEWSDEGYLALSAVNV
KKGYIDFNVEAKYGNLDLYTRWMRGNELYKGQVLFTTEAPMGNVAQVPDNKGYILSQRTIAFNSNE
KITDNFLASLLSSENVYNDLLKLCSGATAKGVSQKNLNRLYVTIPHSISEQEEIAEFFRKINQLVE
LQKYKIEHTKSQKQVFLQKMFIPGGSHHHHHH

1 2 3 4 5 6 7 8 9 10 11 12 13 14 15

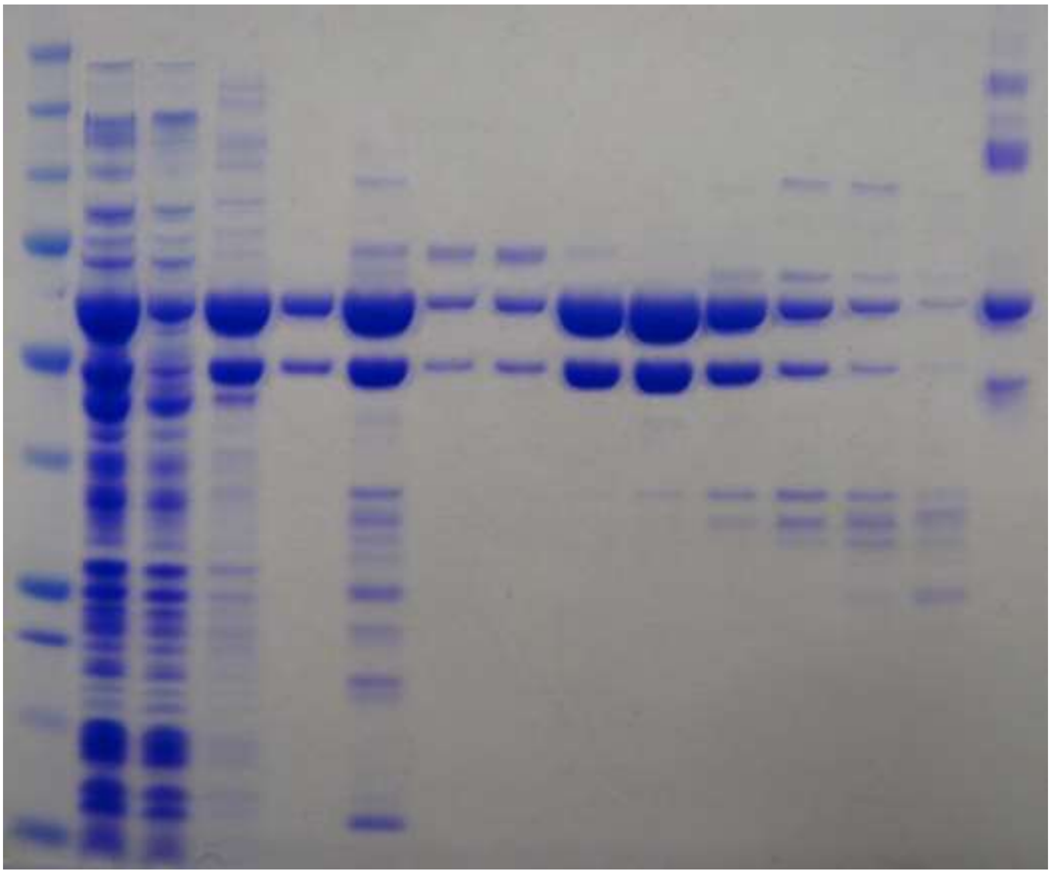

1- marker 2- soluble cell extract 3- Nickel column flow through 4- Nickel column wash 1
5- Nickel column wash 2 6- Nickel column eluate 7-14 Fractions from gel filtration column
15- CC398-1 purified protein marker

Nuclease assay on the plasmid library gave a clear result.

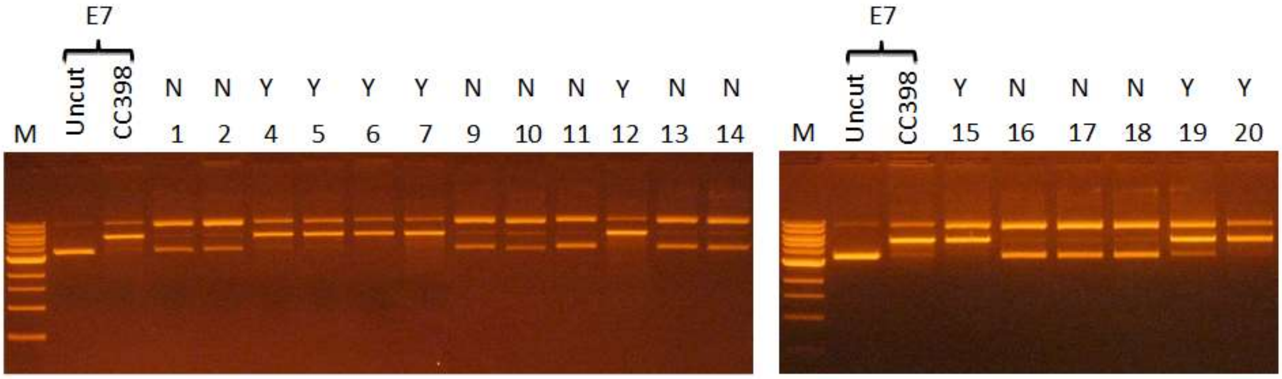

S.SauNK   ACC-6-TCG

MSNTQKKNVPELRFPGFEGEWEEKKLGEFAGKVTQKNVDKKYIETLTNSAELGIISQKDYFDKEIS
NIDNIKKYYVVEENDFVYNPRMSNYAPFGPVNRNKLGKKGVMSPLYTVFKIQNIDLNFIEFYFKSS
KWYRFMALNGDSGARADRFSIKDRTFMEMPLHIPCMDEQIKIGQFFSKLDRQIELEEQKLELLQQQ
KKGYMQKIFSQELRFKDENGNDYPNWEEKKIEDIASQVYGGGTPNTKIKEFWNGDIPWIQSSDVKV
NDLILRQCNKFISKNSIELSSAKLIPANSIAIVTRVGVGKLCLVEFDYATSQDFLSLSSLKYDKLY
SLYSLLYTMKKISANLQGTSIKGITKKELLDSIIKIPHNLEEQQKIGDLFYKIDKYISFNKCKIEI
LKSLKQGLLQKIFIPGGSHHHHHH

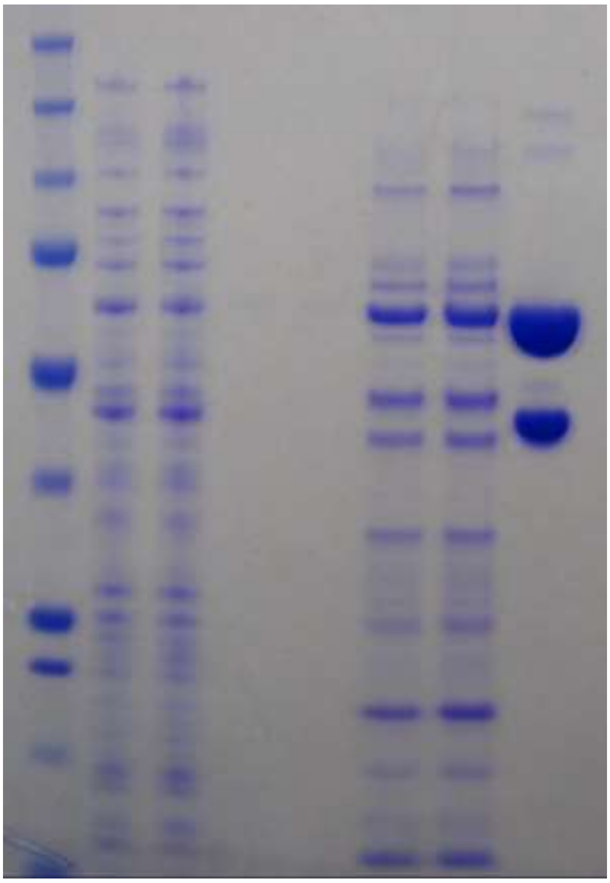

1- marker   2- soluble cell extract   3- Nickel column flow through
4- Nickel column wash 1   5- Nickel column wash 2   6- Nickel column eluate
7- Eluate after PD10 desalting   8- CC398-1 purified protein marker

Nuclease assay gave a clear result.

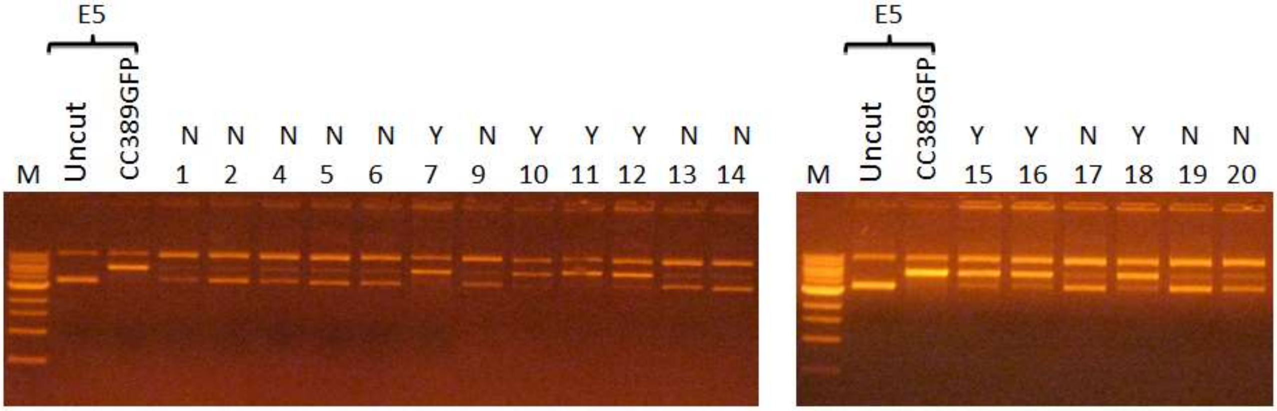

S.SauNL   <u>A</u>CC-6-<u>T</u>AAA

MSNTQKKNVPELRFPGFEGEWEEKKLGEFAGKVTQKNVDKKYIETLTNSAELGIISQKDYFDKEIS
NIDNIKKYYVVEENDFVYNPRMSNYAPFGPVNRNKLGKKGVMSPLYTVFKIQNIDLNFIEFYFKSS
KWYRFMALNGDSGARADRFSIKDRTFMEMPLHIPCMDEQIKIGQFFSKLDRQIELEEQKLELLQQQ
KKGYMQKIFSQELRFKDENGNDYPNWRTIELKNILENIVDNRGKTPDNAPSEKYPLLEVNALGYYR
PAYIKVSKFVSENTYNNWFREHLKENDILFSTVGNTGIVSLMDNYKAVIAQNIVGLRVNNNNLPSF
IYYMLSYKGNQKKIKRIQMGAVQPSVKVSQFKFIKYLVPIKDEQEKVAKLLIEIDKLVNKQLIKIE
LLQQRKKALLKSMFIPGGSHHHHHH

1 2 3 4 5 6 7 8 9 10 11 12 13 14 15

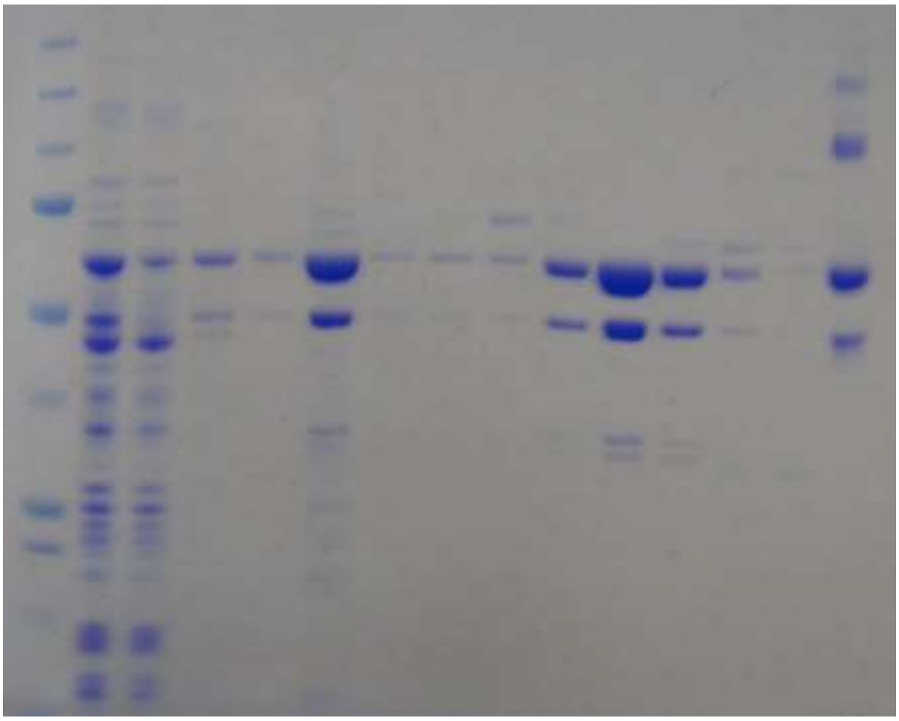

1- marker   2- soluble cell extract   3- Nickel column flow through   4- Nickel column wash 1
5- Nickel column wash 2   6- Nickel column eluate   7-14 Fractions from gel filtration column
15- CC398-1 purified protein marker

Nuclease assay gave a clear result.

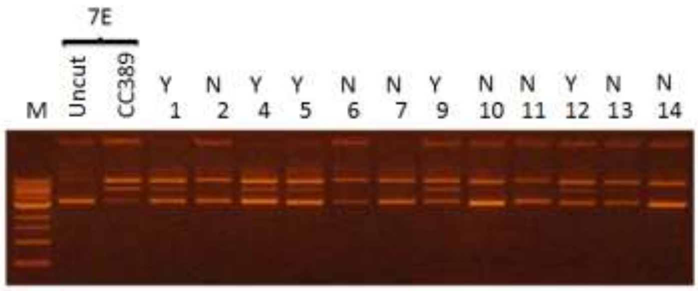

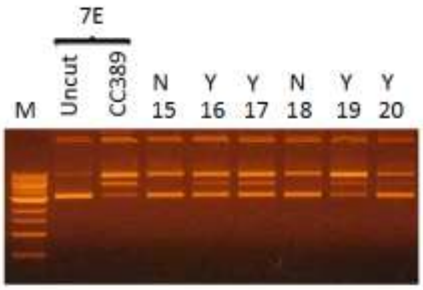

S.SauNP   ACC-5-CCT

MSNTQKKNVPELRFPGFEGEWEEKKLGEFAGKVTQKNVDKKYIETLTNSAELGIISQKDYFDKEIS
NIDNIKKYYVVEENDFVYNPRMSNYAPFGPVNRNKLGKKGVMSPLYTVFKIQNIDLNFIEFYFKSS
KWYRFMALNGDSGARADRFSIKDRTFMEMPLHIPCMDEQIKIGQFFSKLDRQIELEEQKLELFQQQ
KKGYMQKIFSQELRFKDESGNDYPDWEEKELGEVADRVIRKNKNFESKKPLTISGQLGLIDQTEYF
SKSVSSKNLENYTLIKNGEFAYNKSYSNGYPLGAIKRLTRYDSGVLSSLYICFSIKSEMSKDFMEA
YFDSTHWYREVSGIAVEGARNHGLLNISVNDFFTILIKYPSLEEQRKIGDFFIKLDRQIELEEQKL
ELLQQRKKALLKSMLIPGGSHHHHHH

1 2 3 4 5 6 7 8

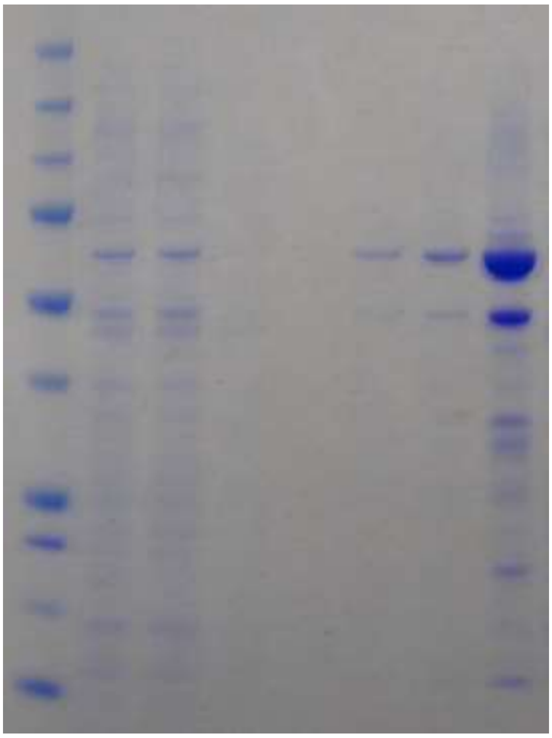

1- marker, 2- soluble cell extract, 3- Nickel column flow through, 4- Nickel column wash 1, 5- Nickel column wash 2, 6- Nickel column eluate, 7- eluate after conc. and PD10 desalting, 8- Final concentrated protein

Although purified this MTase was only assayed via SMRT.

S.SauNP ACC-5-CCT

Motifs

Motif	Modified Position	Modification Type	% Motifs Detected	# Of Motifs Detected	# Of Motifs In Genome	Mean Modification QV	Mean Motif Coverage	Partner Motif
ACCNNNNNCCT	1	m6A	91.03	1320	1450	49.8	29.6	AGGNNNNNGGT
AGGNNNNNGGT	1	m6A	89.79	1302	1450	50.0	29.7	ACCNNNNNCCT
HCCACCNNNNNCCM	4	m6A	17.39	52	299	40.0	34.2	
Not Clustered	0		0.01	737	9114127	34.7	34.2	

Modification QV Histogram By Motif

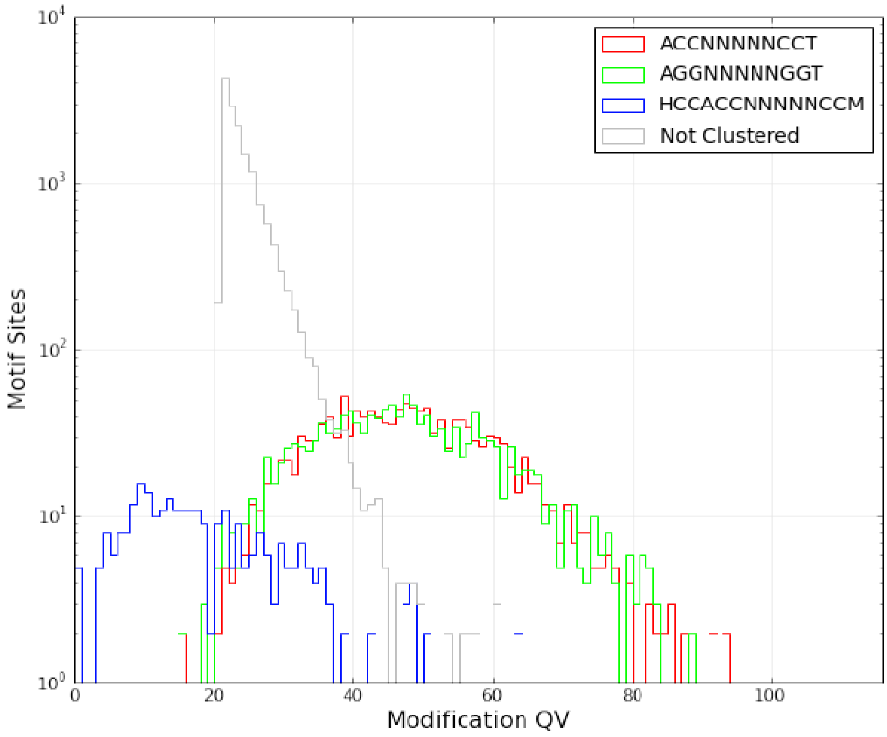

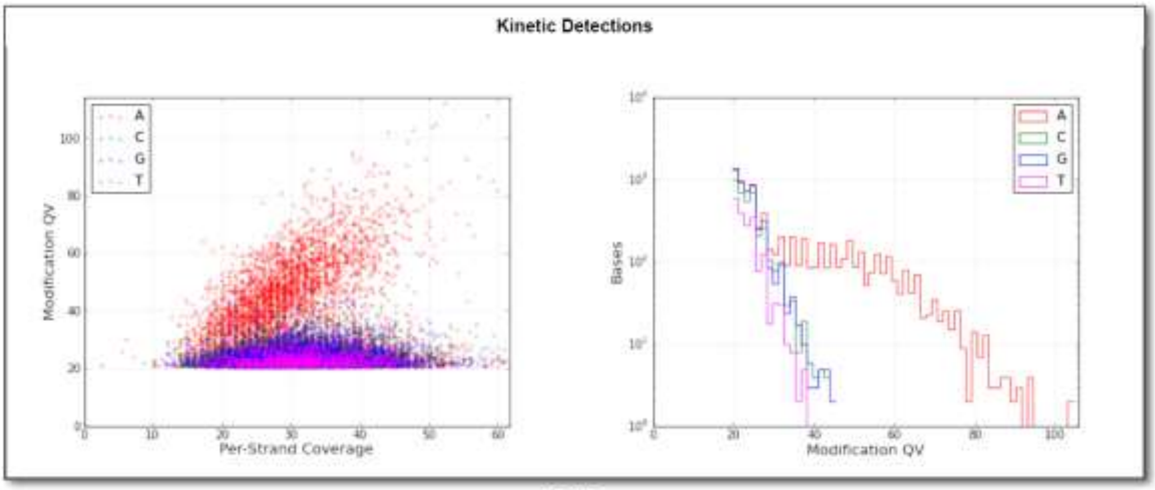

Motifs

Motif	Modified Position	Modification Type	% Motifs Detected	# Of Motifs Detected	# Of Motifs In Genome	Mean Modification QV	Mean Motif Coverage	Partner Motif
ACCNNNNNCCT	1	m6A	91.03	1320	1450	49.8	29.6	AGGNNNNNGGT
AGGNNNNNGGT	1	m6A	89.79	1302	1450	50.0	29.7	ACCNNNNNCCT
HCCACCNNNNNCCM	4	m6A	17.39	52	299	40.0	34.2	
Not Clustered	0		0.01	737	9114127	34.7	34.2	

S.SauNQ   ACC-5-RTGT

MSNTQKKNVPELRFPGFEGEWEEKKLGEFAGKVTQKNVDKKYIETLTNSAELGIISQKDYFDKEIS
NIDNIKKYYVVEENDFVYNPRMSNYAPFGPVNRNKLGKKGVMSPLYTVFKIQNIDLNFIEFYFKSS
KWYRFMALNGDSGARADRFSIKDRTFMEMPLHIPCMDEQIKIGQFFSKLDRQIELEEQKLELLQQQ
KKGYMQKIFSQELRFKDENGEDYSEWEERRFADIFKFHNKLRKPIKENLRVKGSYPYYGATGIIDY
VDDFIFDGNYLLIGEDGANIITRSAPLVYLVNGKFWVNNHAHILSPLNGNIQYLYQVAELVNYEKY
NTGTAQPKLNIQNLKIINVVISTNLEEQQKIGSFLSKLDRQIDLEEQKLELLQQRKKALLKSMFVP
GGSHHHHHH

1 2 3 4 5 6 7

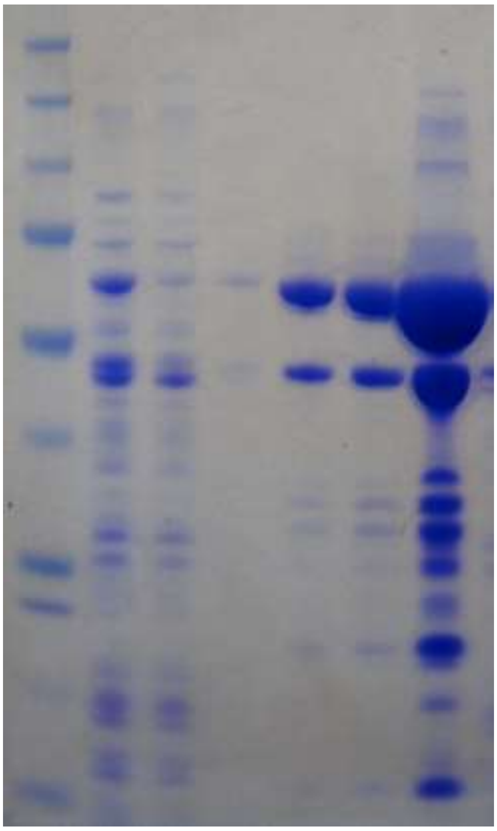

1- marker 2- soluble cell extract 3- Nickel column flow through
4- Nickel column wash 5- Nickel column eluate 6- eluate after conc. and PD10 desalting
7- Final concentrated protein

DNA cleavage assay and SMRT assay agreed.

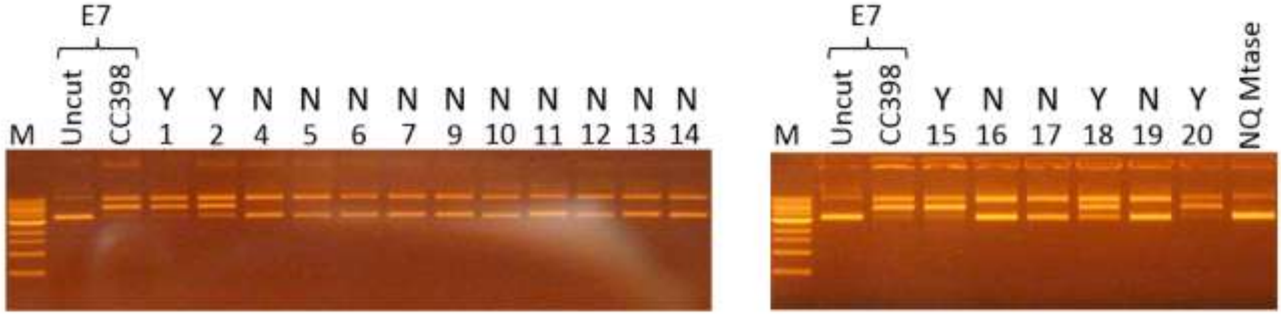

S.SauNQ ACC-5-RTGT

Motifs

Motif	Modified Position	Modification Type	% Motifs Detected	# Of Motifs Detected	# Of Motifs In Genome	Mean Modification QV	Mean Motif Coverage	Partner Motif
ACAYNNNNNGGT	3	m6A	99.85	654	655	83.5	56.3	ACCNNNNNRTGT
ACCNNNNNRTGT	1	m6A	99.85	654	655	80.7	55.5	ACAYNNNNNGGT
ACCNNNNNRAGTH	1	m6A	55.56	215	387	54.3	56.5	
BNACCNNNNNRGGTH	3	m6A	23.74	118	497	45.8	57.6	
DACAGNNNNNGGTNR	4	m6A	21.65	50	231	42.8	55.5	
HACCNNNNNDAGTG	2	m6A	21.03	41	195	44.8	57.9	
YNACCVNNNNCTGT	3	m6A	20.52	47	229	42.9	57.7	
Not Clustered	0		0.03	3095	9114477	35.3	62.5	

Modification QV Histogram By Motif

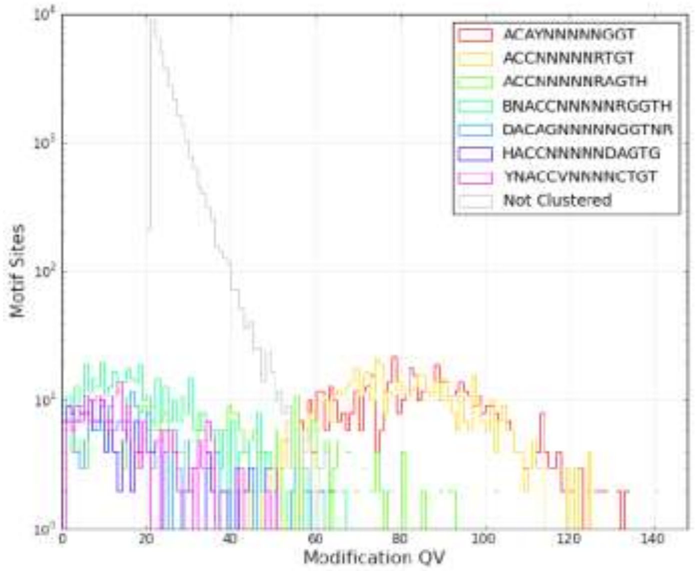

Kinetic Detections

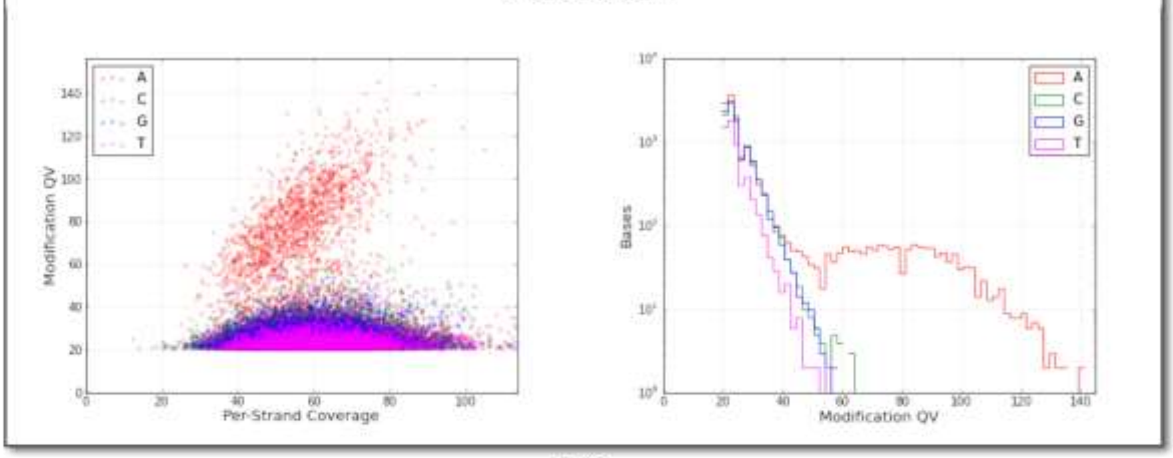

Motifs

Motif	Modified Position	Modification Type	% Motifs Detected	# Of Motifs Detected	# Of Motifs In Genome	Mean Modification QV	Mean Motif Coverage	Partner Motif
ACAYNNNNNGGT	3	m6A	99.85	654	655	83.5	56.3	ACCNNNNNRTGT
ACCNNNNNRTGT	1	m6A	99.85	654	655	80.7	55.5	ACAYNNNNNGGT
ACCNNNNNRAGTH	1	m6A	55.56	215	387	54.3	56.5	
BNACCNNNNNRGGTH	3	m6A	23.74	118	497	45.8	57.6	
DACAGNNNNNGGTNR	4	m6A	21.65	50	231	42.8	55.5	
HACCNNNNNDAGTG	2	m6A	21.03	41	195	44.8	57.9	
YNACCVNNNNCTGT	3	m6A	20.52	47	229	42.9	57.7	
Not Clustered	0		0.03	3095	9114477	35.3	62.5	

S.SauNS **ACC-6-TGC**

MSNTQKKNVPELRFPGFEGEWEEKKLGEFAGKVTQKNVDKKYIETLTNSAELGIISQKDYFDKEIS
NIDNIKKYYVVEENDFVYNPRMSNYAPFGPVNRNKLGKKGVMSPLYTVFKIQNIDLNFIEFYFKSS
KWYRFMALNGDSGARADRFSIKDRTFMEMPLHIPCMDEQIKIGQFFSKLDRQIELEEQKLELLQQQ
KKGYMQKIFSQELRFKDENGNDYPDWTNERLGEVTTVTMGQSPKSVNYTDNSNDTVLIQGNADIEN
GLINPRIYTREVTKLIQKDEIILTVRAPVGKLAMAQINACIGRGVCSIKGDKFLYYFLEWFATQNK
WIRFSQGSTFESISGNDIRNIHIKIPVEDERTKIIKLLNSLDVLNSKTDLKIQNLKQRKQSLLQKI
FVPGGSHHHHHH

1 2 3 4 5 6 7

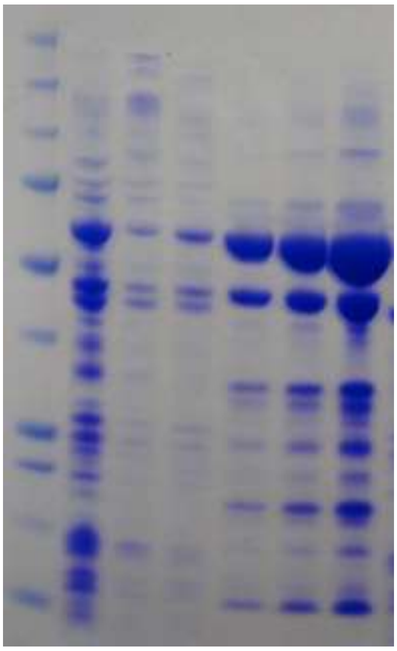

1- marker 2- soluble cell extract
3- Nickel column flow through 4- Nickel column wash
5- Nickel column eluate 6- eluate after conc. and PD10 desalting
7- final protein after concentration

Although purified this MTase was only assayed via SMRT.

Motifs

Motif	Modified Position	Modification Type	% Motifs Detected	# Of Motifs Detected	# Of Motifs In Genome	Mean Modification QV	Mean Motif Coverage	Partner Motif
ACCNNNNNNTGC	1	m6A	100.00	2938	2938	118.8	81.7	GCANNNNNNGGT
GCANNNNNNGGT	3	m6A	99.90	2935	2938	120.7	83.9	ACCNNNNNNTGC
ACCNNNNHHTMCD	1	m6A	57.03	925	1622	71.1	83.7	HGKADDNNNNGGT
HGKADDNNNNGGT	4	m6A	48.83	792	1622	68.8	86.4	ACCNNNNHHTMCD
YNACCNNNNHNVGC	3	m6A	46.49	1925	4141	57.7	84.6	
CCACCNNNNNNTGG	3	m6A	39.15	74	189	55.7	85.9	
TNCCANDNNNNGGTNR	5	m6A	31.60	73	231	53.5	82.5	
YNACCNNNNGHMGC	3	m6A	31.35	195	622	53.2	87.4	
YRNACCNNNNHNVGC	4	m6A	28.65	465	1623	48.9	86.3	
ACCNNNNHHTMCC	1	m6A	27.58	131	475	58.1	84.3	
Not Clustered	0		0.09	8284	9100925	38.6	92.5	

S.SauNS ACC-6-TGC

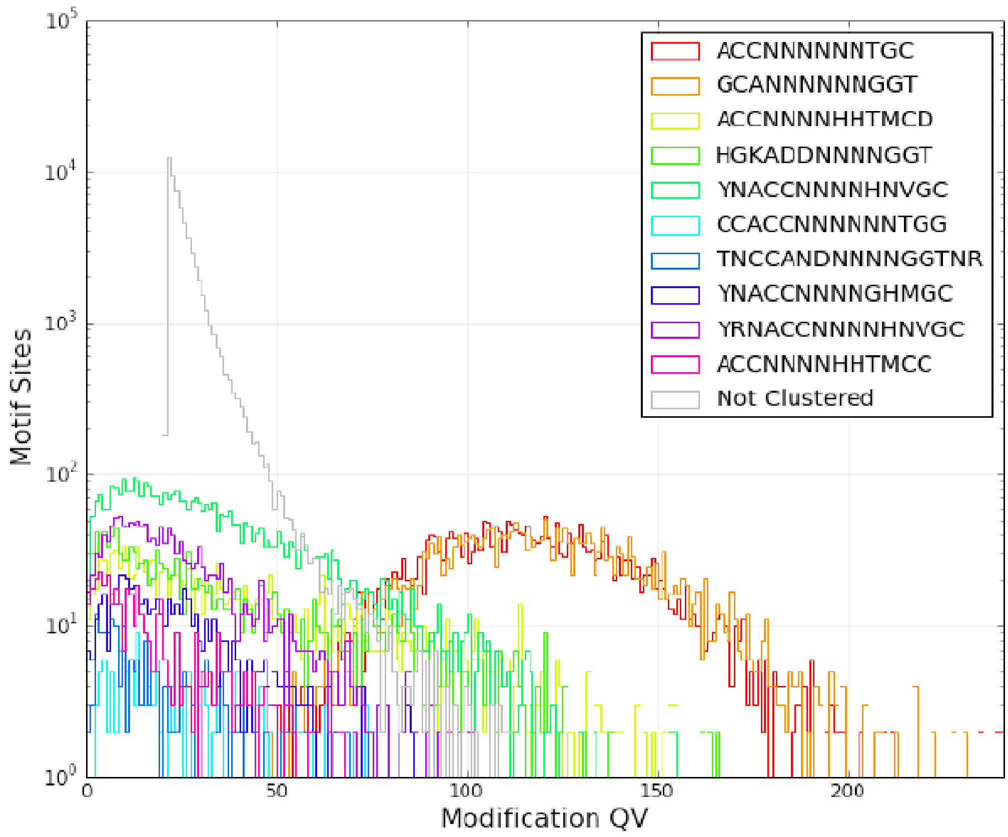

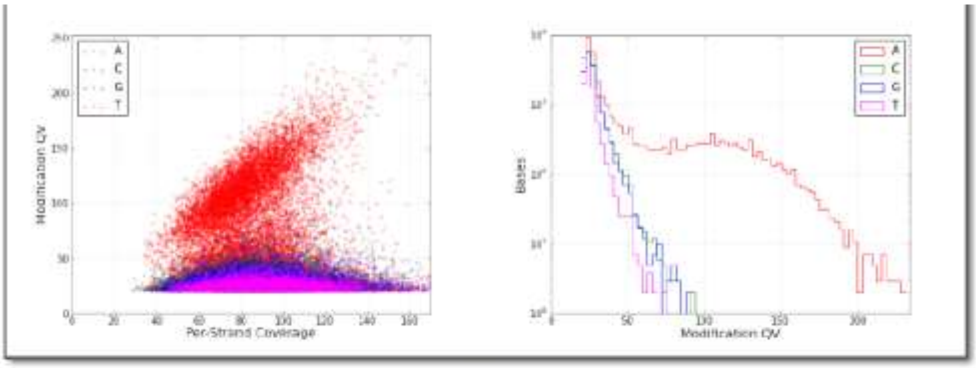

Motifs

Motif	Modified Position	Modification Type	% Motifs Detected	# Of Motifs Detected	# Of Motifs in Genome	Mean Modification QV	Mean Motif Coverage	Partner Motif
ACCNNNNNNTGC	1	m6A	100.00	2938	2938	118.8	81.7	GCANNNNNNGGT
GCANNNNNNGGT	3	m6A	99.90	2935	2938	120.7	83.9	ACCNNNNNNTGC
ACCNNNNHHTMCD	1	m6A	57.03	925	1622	71.1	83.7	HGKADDNNNNGGT
HGKADDNNNNGGT	4	m6A	48.83	792	1622	68.8	86.4	ACCNNNNHHTMCD
YNACCNNNNHNVGC	3	m6A	46.49	1925	4141	57.7	84.6	
CCACCNNNNNNTGG	3	m6A	39.15	74	189	55.7	85.9	
TNCCANDNNNNGGTNR	5	m6A	31.60	73	231	53.5	82.5	
YNACCNNNNGHMGC	3	m6A	31.35	195	622	53.2	87.4	
YRNACCNNNNHNVGC	4	m6A	28.65	465	1623	48.9	86.3	
ACCNNNNHHTMCC	1	m6A	27.58	131	475	58.1	84.3	
Not Clustered	0		0.09	8204	9100925	38.6	92.5	

S.SauNU   <u>A</u>CC-5-R<u>T</u>C

MSNTQKKNVPELRFPGFEGEWEEKKLGEFAGKVTQKNVDKKYIETLTNSAELGIISQKDYFDKEIS
NIDNIKKYYVVEENDFVYNPRMSNYAPFGPVNRNKLGKKGVMSPLYTVFKIQNIDLNFIEFYFKSS
KWYRFMALNGDSGARADRFSIKDRTFMEMPLHIPCMDEQIKIGQFFSKLDRQIELEEQKLELLQQQ
KKGYMQKIFSQELRFKDENGEDYPDWEVTTIQNITKYTSSKKSSNQYADKDNSKGYPVYDAVQEIG
KDSNYDIEESYISILKDGAGVGRLNLRPGKSSVIGTMGYIQSNNVDIEFLYYRMKVVDFKKYIIGS
TIPHLYFKDYSKETLYIPSSIQEQAKIGMFISNLDKLIENKNLKLNCLKQLKQGLLQSMFIPGGSH
HHHHH

1 2 3 4 5 6 7

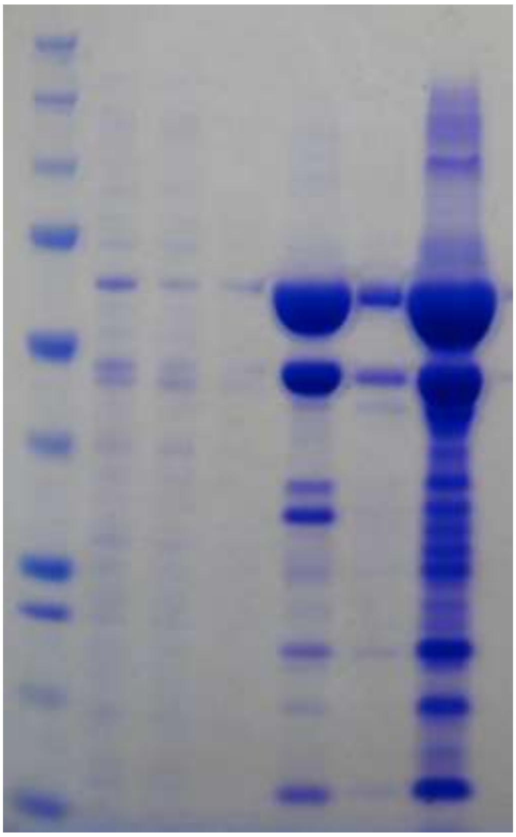

1- marker 2- soluble cell extract
3- Nickel column flow through 4- Nickel column wash
5- Nickel column eluate 6- eluate after PD10 desalting
7- final protein after concentration

DNA cleavage assay worked despite there being one site in pUC19 <u>but</u> this site was subject to dam methylation and therefore not cut.

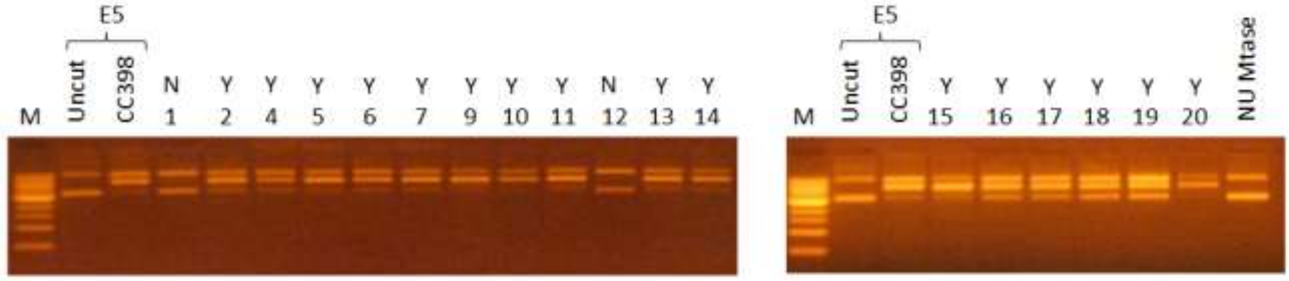

S.SauNU ACC-5-RTC

Modification QV Histogram By Motif

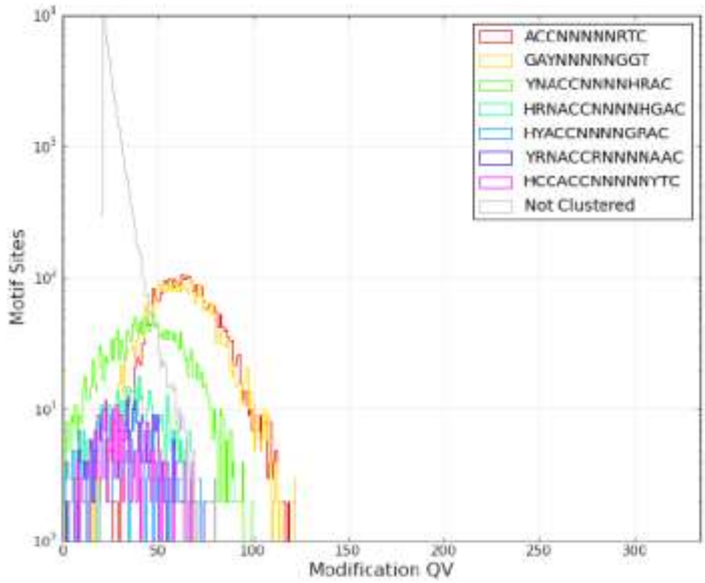

Motifs

Motif	Modified Position	Modification Type	% Motifs Detected	# Of Motifs Detected	# Of Motifs In Genome	Mean Modification QV	Mean Motif Coverage	Partner Motif
ACCNNNNNRTC	1	m6A	81.87	3162	3862	70.1	41.5	GAYNNNNNGGT
GAYNNNNNGGT	2	m6A	75.14	2902	3862	70.8	41.9	ACCNNNNNRTC
YNACCNNNNHRAC	3	m6A	37.48	820	2188	64.9	43.3	
HRNACCNNNNHGAC	4	m6A	26.22	140	534	63.7	43.0	
HYACCNNNNGRAC	3	m6A	19.23	50	260	63.3	44.4	
YRNACCRNNNNAAC	4	m6A	17.15	59	344	63.7	45.8	
HCCACCNNNNNYTC	4	m6A	16.81	39	232	61.9	45.9	
Not Clustered	0		0.00	229	9106044	58.3	48.5	

Kinetic Detections

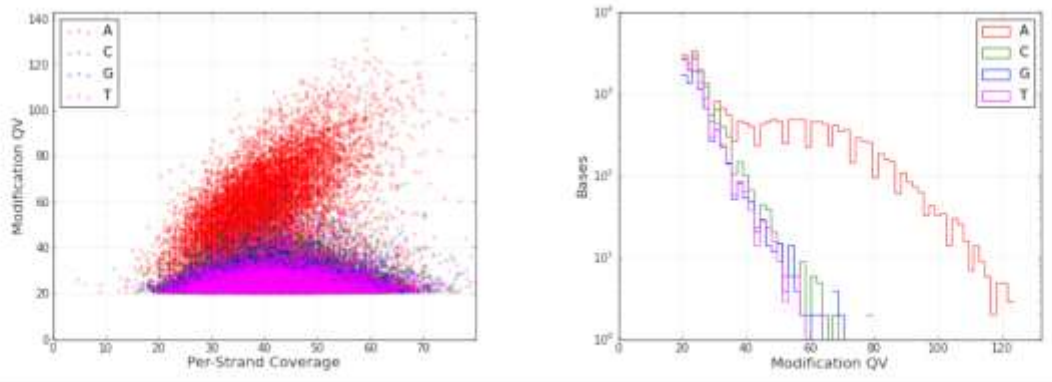

Motifs

Motif	Modified Position	Modification Type	% Motifs Detected	# Of Motifs Detected	# Of Motifs In Genome	Mean Modification QV	Mean Motif Coverage	Partner Motif
ACCNNNNNRTC	1	m6A	81.87	3162	3862	70.1	41.5	GAYNNNNNGGT
GAYNNNNNGGT	2	m6A	75.14	2902	3862	70.8	41.9	ACCNNNNNRTC
YNACCNNNNHRAC	3	m6A	37.48	820	2188	64.9	43.3	
HRNACCNNNNHGAC	4	m6A	26.22	140	534	63.7	43.0	
HYACCNNNNGRAC	3	m6A	19.23	50	260	63.3	44.4	
YRNACCRNNNNAAC	4	m6A	17.15	59	344	63.7	45.8	
HCCACCNNNNNYTC	4	m6A	16.81	39	232	61.9	45.9	
Not Clustered	0		0.00	229	9106044	58.3	48.5	

S.SauNW   ACC-6-TTYG

MSNTQKKNVPELRFPGFEGEWEEKKLGEFAGKVTQKNVDKKYIETLTNSAELGIISQKDYFDKEIS
NIDNIKKYYVVEENDFVYNPRMSNYAPFGPVNRNKLGKKGVMSPLYTVFKIQNIDLNFIEFYFKSS
KWYRFMALNGDSGARADRFSIKDRTFMEMPLHIPCMDEQIKIGQFFSKLDRQIELEEQKLELLQQQ
KKGYMQKIFSQELRFKDENGNDYPDWEEKQLGELSQIVRGASPRPIKDPKWFNKESDIGWLRISDV
TNQNGKIYHLEQKLSIEGQEKTRVLVTTHLLLSIAASIGKPVMNFVKTGVHDGFLIFLKPKFNLFF
MYYWLEYFKDKWSKYGQPGSQVNLNSEIVKSQTLNMPSNHEQEKVGQFFNRNEKLIELQQEKIMYI
KRCKQVLLQKMFIPGGSHHHHHH

1 2 3 4 5 6 7

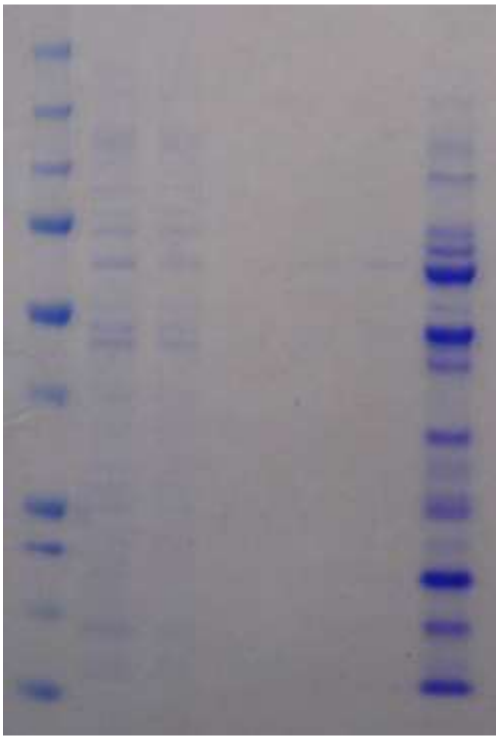

1- marker   2- soluble cell extract
3- Nickel column flow through   4- Nickel column wash
5- Nickel column eluate   6- eluate after PD10 desalting
7- final protein after concentration

DNA cleavage assay.

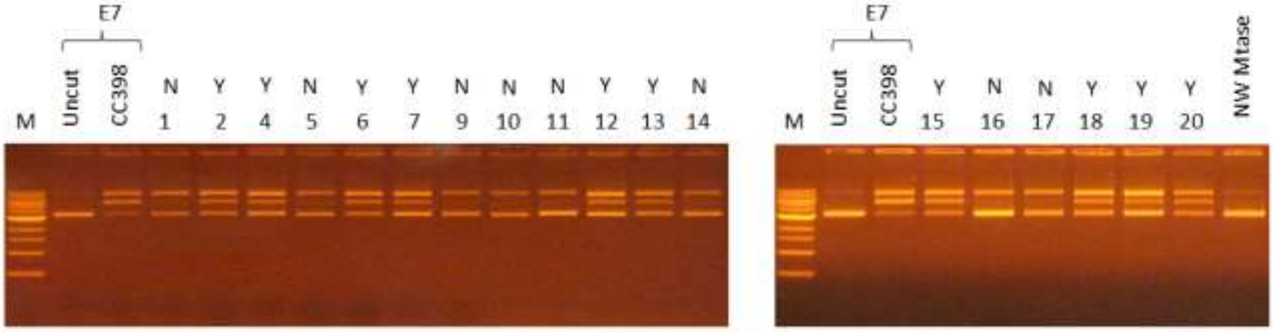

S.SauNW   ACC-6-TTYG

Motifs

Motif	Modified Position	Modification Type	% Motifs Detected	# Of Motifs Detected	# Of Motifs In Genome	Mean Modification QV	Mean Motif Coverage	Partner Motif
ACCNNNNNNTTYG	1	m6A	99.86	1461	1463	80.7	49.6	CRAANNNNNNGGT
CRAANNNNNNGGT	4	m6A	99.59	1457	1463	74.7	48.6	ACCNNNNNNTTYG
YNACCNNNNNNTSCG	3	m6A	39.52	313	792	52.0	52.7	CGSANNNNNNGGTNR
CGSANNNNNNGGTNR	4	m6A	35.23	279	792	50.1	52.3	YNACCNNNNNNTSCG
ACCNNNNNBRTYG	1	m6A	28.16	680	2415	49.3	51.3	
Not Clustered	0		0.08	6917	9110401	37.6	55.8	

Modification QV Histogram By Motif

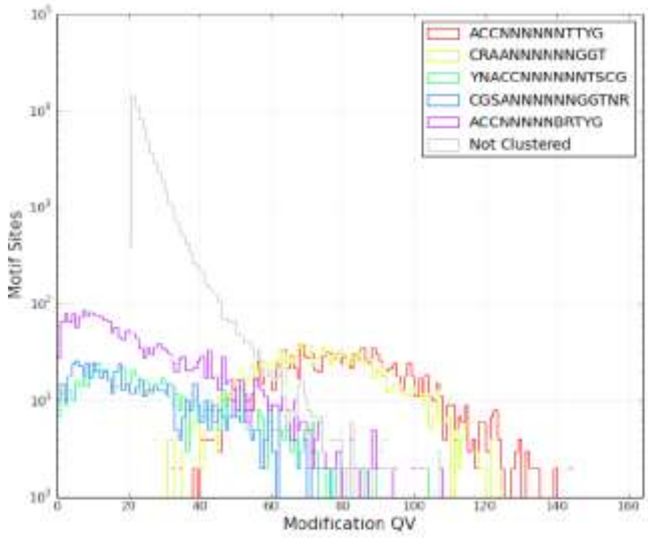

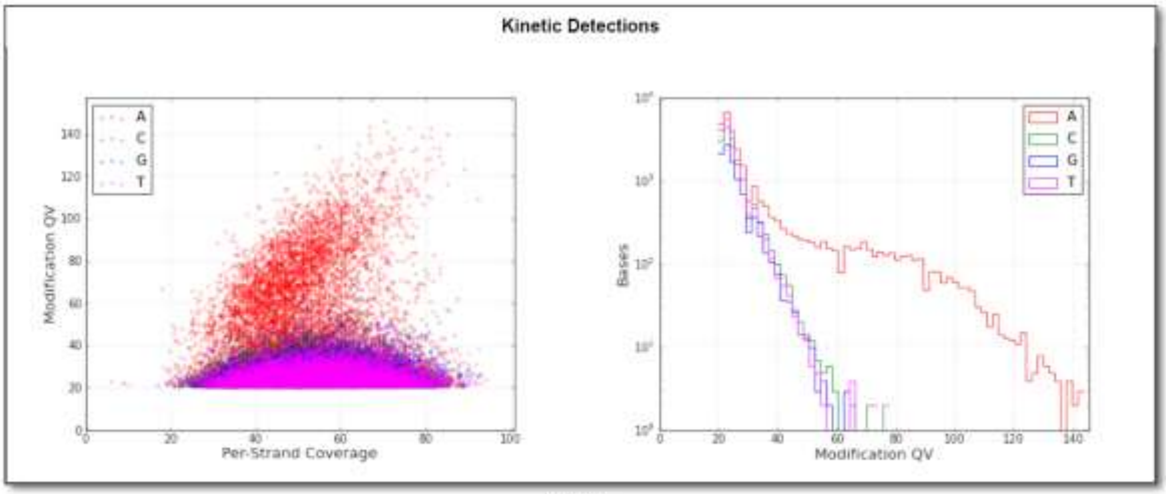

Motifs

Motif	Modified Position	Modification Type	% Motifs Detected	# Of Motifs Detected	# Of Motifs In Genome	Mean Modification QV	Mean Motif Coverage	Partner Motif
ACCNNNNNNTTYG	1	m6A	99.86	1461	1463	80.7	49.6	CRAANNNNNNGGT
CRAANNNNNNGGT	4	m6A	99.59	1457	1463	74.7	48.6	ACCNNNNNNTTYG
YNACCNNNNNNTSCG	3	m6A	39.52	313	792	52.0	52.7	CGSANNNNNNGGTNR
CGSANNNNNNGGTNR	4	m6A	35.23	279	792	50.1	52.3	YNACCNNNNNNTSCG
ACCNNNNNBRTYG	1	m6A	28.16	680	2415	49.3	51.3	
Not Clustered	0		0.08	6917	9110401	37.6	55.8	

S.SauNY ACC-6-TAG

MSNTQKKNVPELRFPGFEGEWEEKKLGEFAGKVTQKNVDKKYIETLTNSAELGIISQKDYFDKEIS
NIDNIKKYYVVEENDFVYNPRMSNYAPFGPVNRNKLGKKGVMSPLYTVFKIQNIDLNFIEFYFKSS
KWYRFMALNGDSGARADRFSIKDRTFMEMPLHIPCMDEQIKIGQFFSKLDRQIELEEQKLELLQQQ
KKGYMQKIFSQELRFKDENGNDYPDWEKKKLKEIACVYTGNTPSKKENIYWNKGEYVWVTPTDINN
SKNIYESENKLTQEGYKKARQLPENTLLVTCIASIGKNAILRKQGSCNQQINAVVPFENINIDYLY
YISDSLSTFMKSIAGKTATQIVNKNTFENLEIYLAPFEEQNKIADLISSLEELIEKQASKLIKMKS
RKQGMLQIMFIPGGSHHHHHH

1 2 3 4 5 6

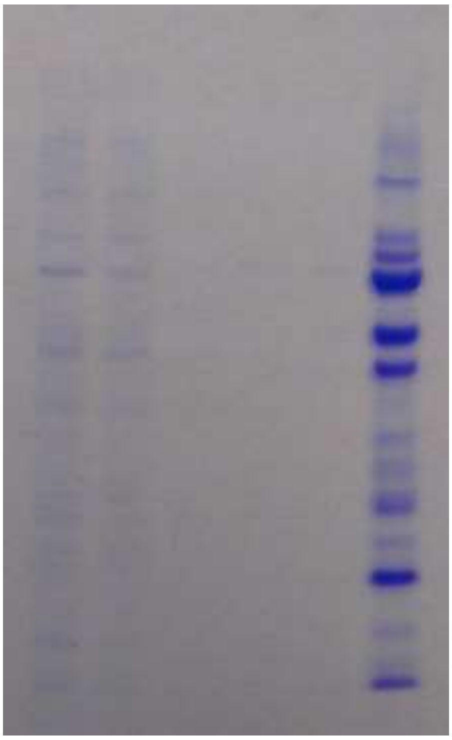

1- soluble cell extract 2- Nickel column flow through
3- Nickel column wash 1 4- Nickel column wash 2 5- Nickel column eluate
6- final protein after PD10 desalting and concentration of eluate

DNA cleavage assay.

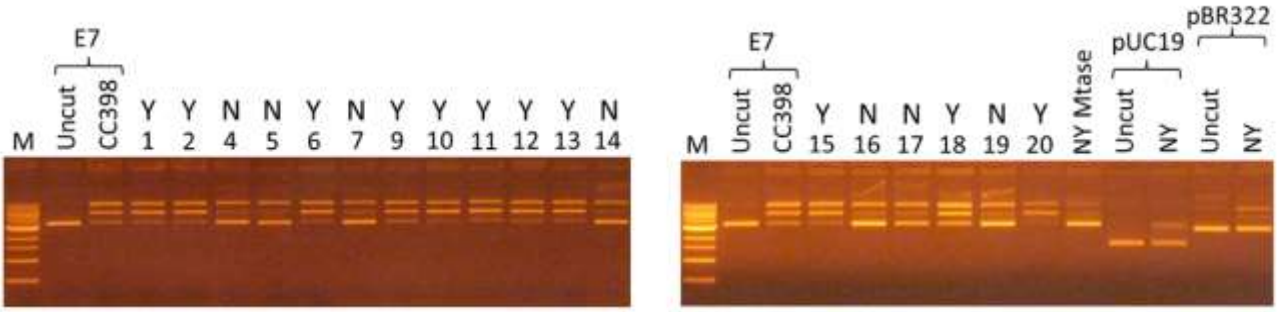

S.SauNY ACC-6-TAG

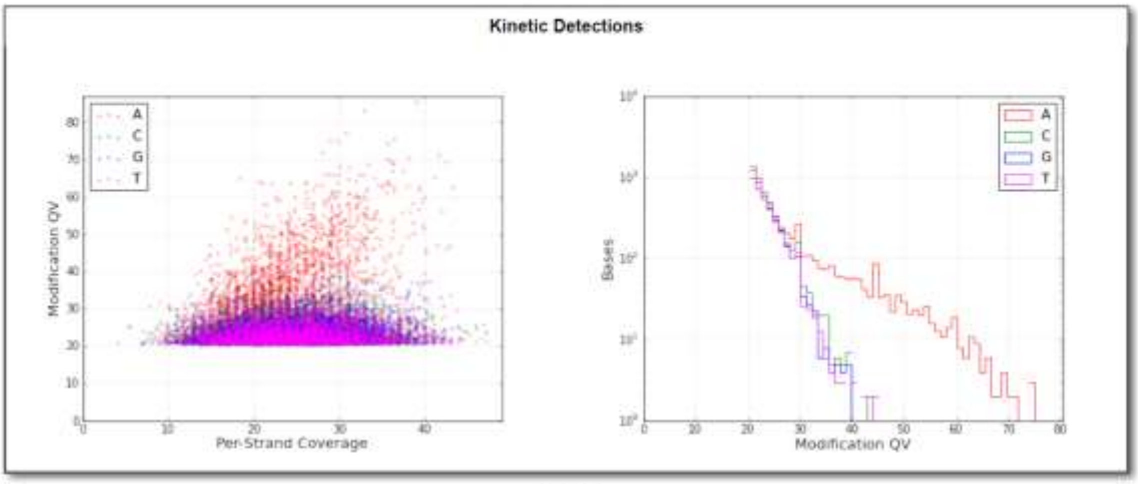

Motifs

Motif	Modified Position	Modification Type	% Motifs Detected	# Of Motifs Detected	# Of Motifs In Genome	Mean Modification QV	Mean Motif Coverage	Partner Motif
ACCNNNNNNTAG	1	m6A	75.92	539	710	43.3	24.7	CTANNNNNNGGT
CTANNNNNNGGT	3	m6A	72.39	514	710	42.9	24.7	ACCNNNNNNTAG
BNACCNNNNNTAAG	3	m6A	34.00	68	200	39.3	25.0	
CCACCNNNNNNMAGND	3	m6A	23.68	85	359	38.3	26.1	
Not Clustered	0		0.01	622	9115347	34.4	27.6	

Motifs

Motif	Modified Position	Modification Type	% Motifs Detected	# Of Motifs Detected	# Of Motifs In Genome	Mean Modification QV	Mean Motif Coverage	Partner Motif
ACCNNNNNNTAG	1	m6A	75.92	539	710	43.3	24.7	CTANNNNNNGGT
CTANNNNNNGGT	3	m6A	72.39	514	710	42.9	24.7	ACCNNNNNNTAG
BNACCNNNNNTAAG	3	m6A	34.00	68	200	39.3	25.0	
CCACCNNNNNNMAGND	3	m6A	23.68	85	359	38.3	26.1	
Not Clustered	0		0.01	622	9115347	34.4	27.6	

Modification QV Histogram By Motif

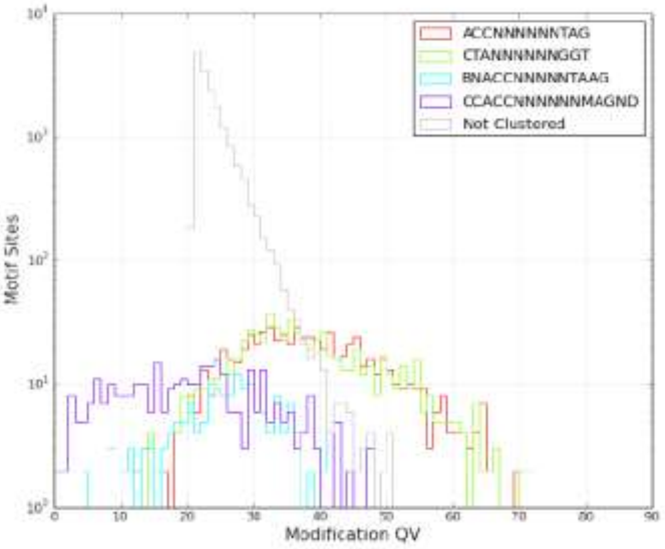

S.SauNa* ACC-6-TTC

Motifs

Motif	Modified Position	Modification Type	% Motifs Detected	# Of Motifs Detected	# Of Motifs In Genome	Mean Modification QV	Mean Motif Coverage	Partner Motif
ACCNNNNNNTTC	1	m6A	85.91	2567	2988	46.4	26.2	GAANNNNNNGGT
GAANNNNNNGGT	3	m6A	78.11	2334	2988	44.7	26.2	ACCNNNNNNTTC
RGAANNNNNNGGVGD	4	m6A	16.09	107	665	37.1	27.8	
Not Clustered	0		0.01	1034	9110685	35.6	31.8	

MSNTQKKNVPELRFPGFEGEWEEKKLGEFAGKVTQKNVDKKYIETLTNSAELGIISQKDYFDKEIS
NIDNIKKYYVVEENDFVYNPRMSNYAPFGPVNRNKLGKKGVMSPLYTVFKIQNIDLNFIEFYFKSS
KWYRFMALNGDSGARADRFSIKDRTFMEMPLHIPCMDEQIKIGQFFSKLDRQIELEEQKLELLQQQ
KKGYMQKIFSQELRFKDENGNDYPEWENKRIEDIANVNKGFTPSTNNNEYWDNNDKNWLSIAGMNQ
KYLYKGNKGISKDAAKNYMKVKNDTLIMSFKLTIGKLAIVKAPLYTNEAICHFIWKVNKINTEFIY
YYLNSLNISTFGVQAVKGVTLNNDSINSIIVKLPNEEEQNIIAKFLLEVDKTVNNQLVKTKLLKQR
KKGLLQRMFVPGGSHHHHHH

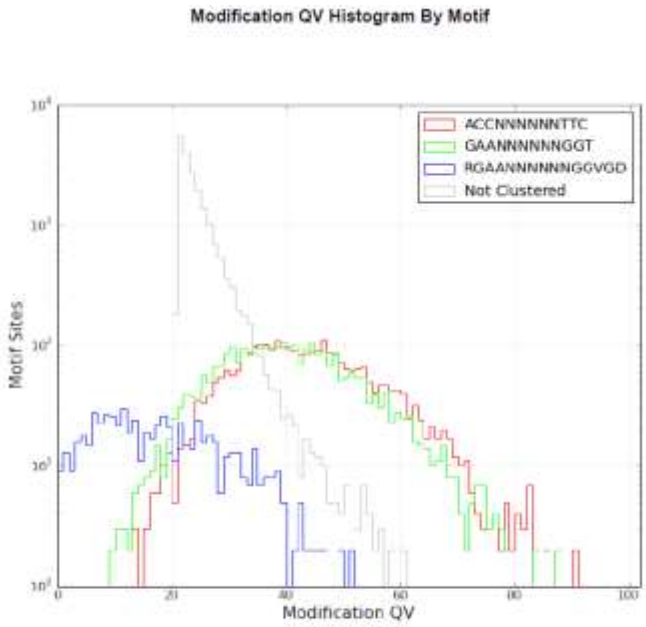

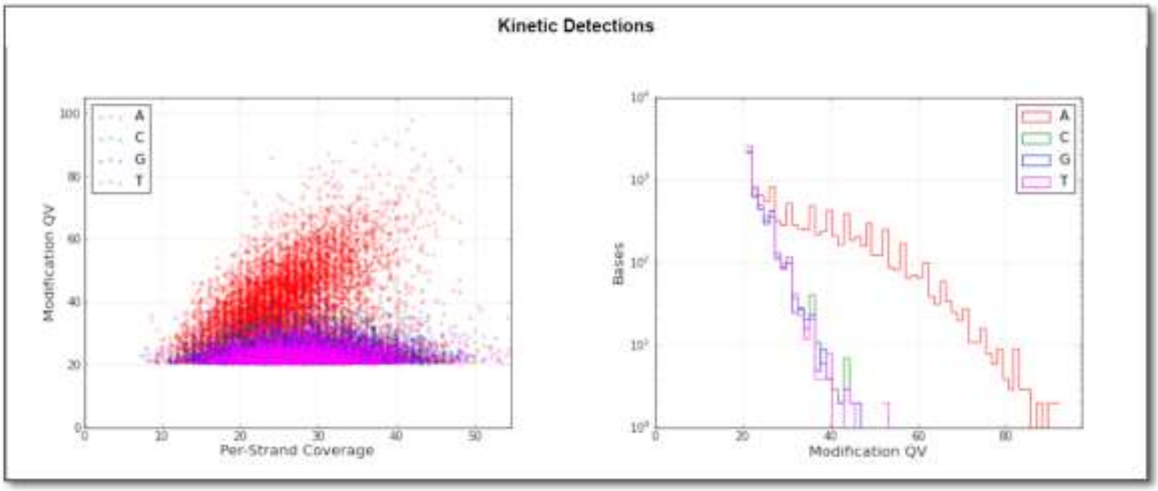

Motifs

Motif	Modified Position	Modification Type	% Motifs Detected	# Of Motifs Detected	# Of Motifs In Genome	Mean Modification QV	Mean Motif Coverage	Partner Motif
ACCNNNNNNTTC	1	m6A	85.91	2567	2988	46.4	26.2	GAANNNNNNGGT
GAANNNNNNGGT	3	m6A	78.11	2334	2988	44.7	26.2	ACCNNNNNNTTC
RGAANNNNNNGGVGD	4	m6A	16.09	107	665	37.1	27.8	
Not Clustered	0		0.01	1034	9110685	35.6	31.8	

S.SauNc* ACC-6-RTC

MSNTQKKNVPELRFPGFEGEWEEKKLGEFAGKVTQKNVDKKYIETLTNSAELGIISQKDYFDKEIS
NIDNIKKYYVVEENDFVYNPRMSNYAPFGPVNRNKLGKKGVMSPLYTVFKIQNIDLNFIEFYFKSS
KWYRFMALNGDSGARADRFSIKDRTFMEMPLHIPCMDEQIKIGQFFSKLDRQIELEEQKLELLQQQ
KKGYLQKIFSQELRFKDENGNDYPEWRFARFKDFMYKPINIRPAINISKSELLTVKLHCKGIEKAN
INRVLKLGATNYYKRFEGQFIYGKQNFFNGAFDIVPKKFDGLYSSSDVPAFEINTEKIEPNYFISY
ISRPSFYKSKEKYSTGTGSKRIHENTVLNFSLHLPCLNEQLKIASFVCFLNRKIELLERKIYLIKK
QKQALLQQMFIPGGSHHHHHH

1 2 3 4 5 6 7 8

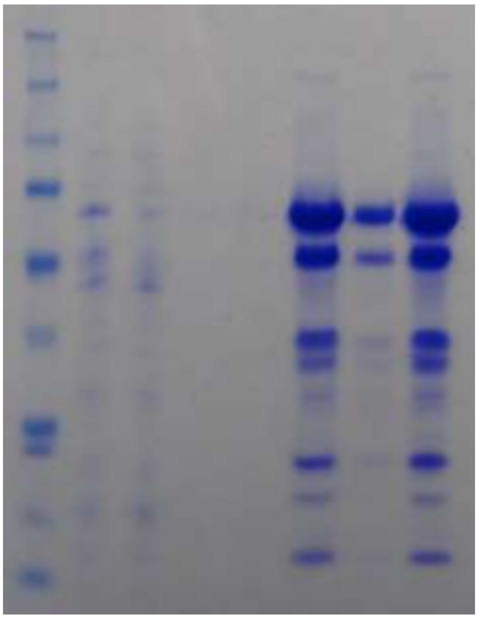

1- marker 2- soluble cell extract
3- Nickel column flow through 4- Nickel column wash 1
5- Nickel column wash 2 6- Nickel column eluate
7- eluate after PD10 desalting
8- final protein after concentration

Although purified, this MTase was only assayed by SMRT.

Motifs

Motif	Modified Position	Modification Type	% Motifs Detected	# Of Motifs Detected	# Of Motifs In Genome	Mean Modification QV	Mean Motif Coverage	Partner Motif
ACCNNNNNNRTC	1	m6A	90.98	4680	5144	48.9	27.4	GAYNNNNNNGGT
GAYNNNNNNGGT	2	m6A	90.18	4639	5144	50.0	27.8	ACCNNNNNNRTC
HACCVNNNNNCTCD	2	m6A	16.64	117	703	40.7	30.2	
HGAGNNNNNYGGTD	3	m6A	16.60	86	518	41.5	29.5	
HACCVNNNHNRMC	2	m6A	14.94	936	6265	40.2	29.1	
Not Clustered	0		0.01	1163	9099552	35.6	31.2	

S.SauNc* ACC-6-RTC

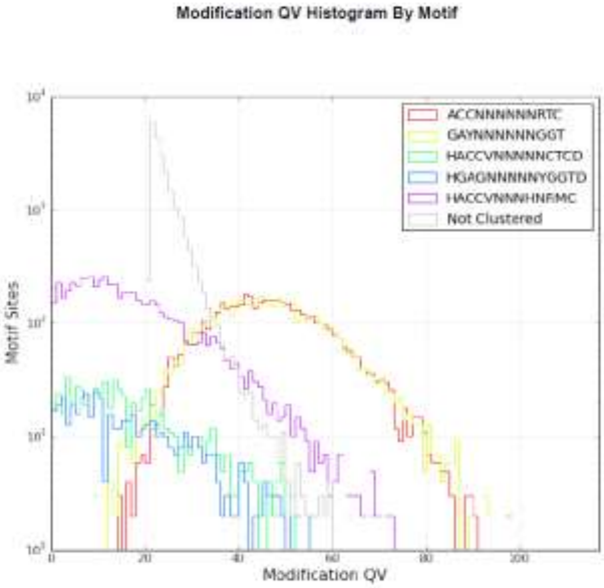

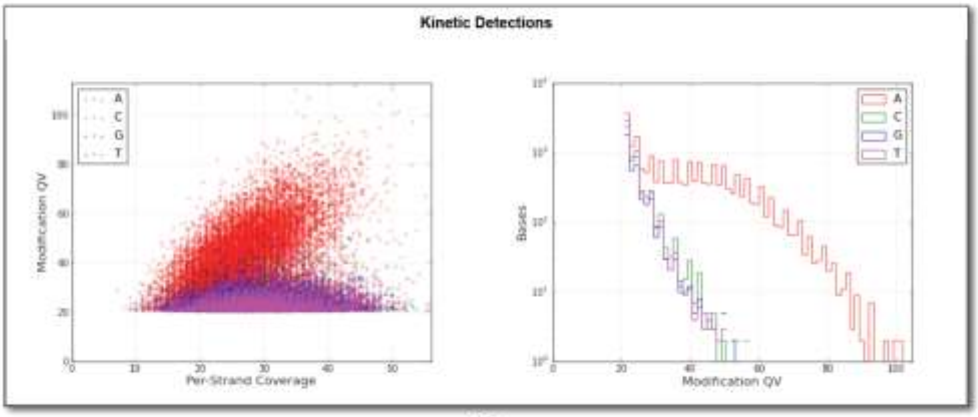

Motifs

Motif	Modified Position	Modification Type	% Motifs Detected	# Of Motifs Detected	# Of Motifs In Genome	Mean Modification QV	Mean Motif Coverage	Partner Motif
ACCNNNNNNRTC	1	m6A	90.98	4680	5144	48.9	27.4	GAYNNNNNNGGT
GAYNNNNNNGGT	2	m6A	90.18	4639	5144	50.0	27.8	ACCNNNNNNRTC
HACCVNNNNNCTCD	2	m6A	16.64	117	703	40.7	30.2	
HGAGNNNNNYGGTD	3	m6A	16.60	86	518	41.5	29.5	
HACCVNNNHNRMC	2	m6A	14.94	936	6265	40.2	29.1	
Not Clustered	0		0.01	1163	9099552	35.6	31.2	

S.SauNd* <u>A</u>CC-6-<u>T</u>TRG

MSNTQKKNVPELRFPGFEGEWEEKKLGEFAGKVTQKNVDKKYIETLTNSAELGIISQKDYFDKEIS
NIDNIKKYYVVEENDFVYNPRMSNYAPFGPVNRNKLGKKGVMSPLYTVFKIQNIDLNFIEFYFKSS
KWYRFMALNGDSGARADRFSIKDRTFMEMPLHIPCMDEQIKIGQFFSKLDRQIELEEQKLELLQQQ
KKGYMQKIFSQELRFKDENGNDYPEWENVMLQKVLKDKTEGIKRGPFGGALKKDIFVESGYAVYEQ
RNAIYDISNFRYYINENKYKEMQSFSVQPNDIIMSCSGTIGRLALIPHNYTKGIINQALIRFRTNH
KIRSEFFLIFMRSNQMQRKILEANPGSAITNLVPVKELKLIPFPLPVKFEQDKISQFIHIINRRIE
QSEKKIESLKNRKQGFLQKLFVPGGSHHHHHH

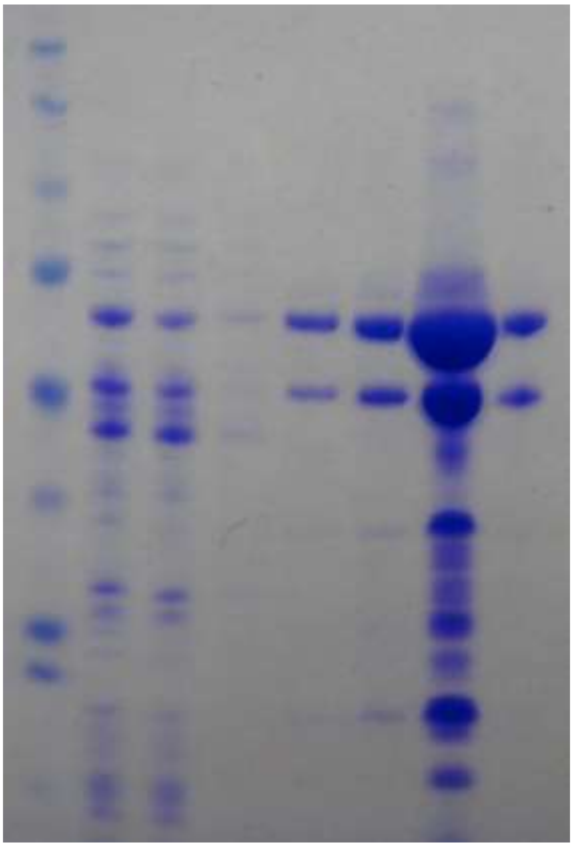

1- marker 2- soluble cell extract 3- Nickel column flow through
4- Nickel column wash 5- Nickel column eluate
6- eluate after conc. and PD10 desalting
7- final protein after concentration 8- NP purified protein marker

DNA cleavage assay.

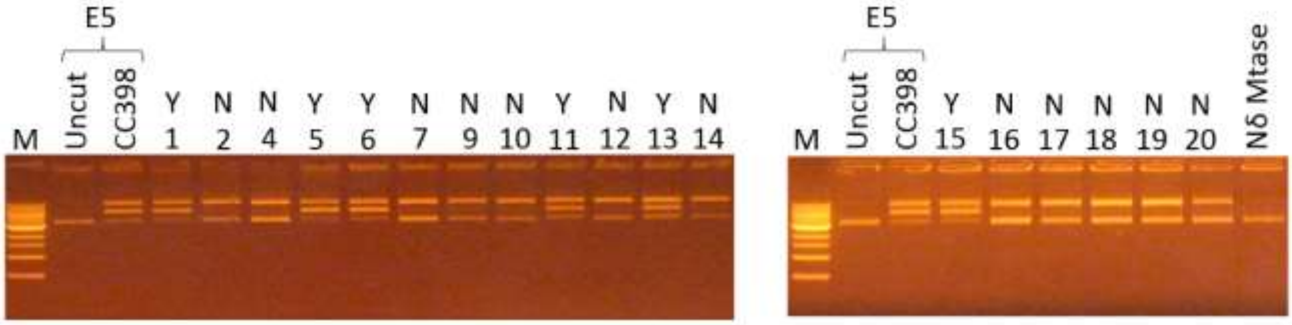

Site determined to be <u>ACC-6-TTRG</u> or ACC-6-YTRG. Note that the underlined site was determined by SMRT and is accepted since if Y is a cytosine, then it can't be methylated.

S.SauNd* <u>A</u>CC-6-<u>T</u>TRG

Motifs

Motif	Modified Position	Modification Type	% Motifs Detected	# Of Motifs Detected	# Of Motifs In Genome	Mean Modification QV	Mean Motif Coverage	Partner Motif
CYAANNNNNNGGT	4	m6A	89.98	557	619	50.0	29.9	
ACCNNNNNNHTRG	1	m6A	67.06	2013	3002	47.5	31.0	
YHACCNNNNNNGTGG	3	m6A	30.03	88	293	40.5	34.0	
CYYANNNNNNGGTDR	4	m6A	20.24	102	504	42.0	32.9	
HNHACCNNNNNNTRRG	4	m6A	17.94	127	708	41.5	32.6	
Not Clustered	0		0.02	1435	9112200	35.8	37.4	

Modification QV Histogram By Motif

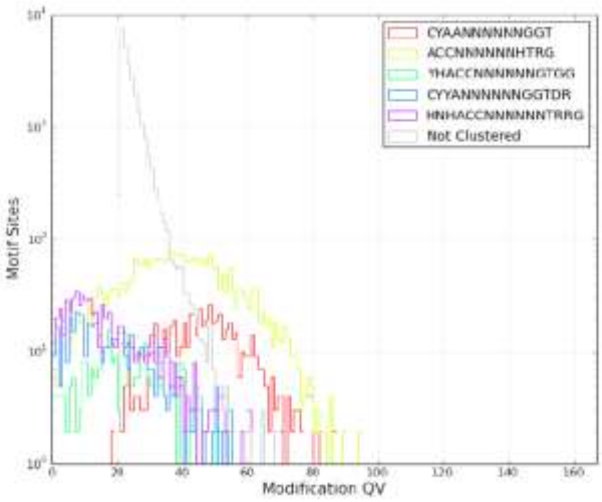

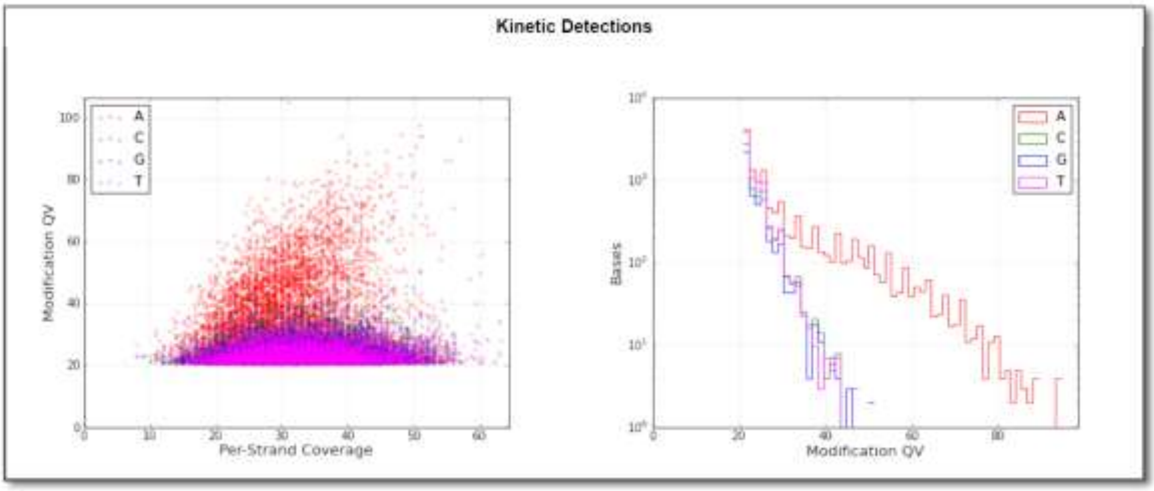

Motifs

Motif	Modified Position	Modification Type	% Motifs Detected	# Of Motifs Detected	# Of Motifs In Genome	Mean Modification QV	Mean Motif Coverage	Partner Motif
CYAANNNNNNGGT	4	m6A	89.98	557	619	50.0	29.9	
ACCNNNNNNHTRG	1	m6A	67.06	2013	3002	47.5	31.0	
YHACCNNNNNNGTGG	3	m6A	30.03	88	293	40.5	34.0	
CYYANNNNNNGGTDR	4	m6A	20.24	102	504	42.0	32.9	
HNHACCNNNNNNTRRG	4	m6A	17.94	127	708	41.5	32.6	
Not Clustered	0		0.02	1435	9112200	35.8	37.4	

S.SauRE GAR<u>A</u>-6-R<u>T</u>GA

MSNTQKKNVPELRFPGFEGEWEEKKLGEVAKIYDGTHQTPKYTNEGIKFLSVENIKTLNSSKYISE
EAFEKEFKIRPEFGDILMTRIGDIGTPNIVSSNEKFAYYVSLALLKTKNLNSYFLKNLILSSSIQN
ELWRKTLHVAFPKKINKNEIGKIKINYPKKQEQQKIGQFFSKLDRQIELEEQKLELLQQQKKGYMQ
KIFSQELRFKDENGKDYPEWEETTIKEIAQINTGKKDTKDAITNGSYDFYVRSPIVYKINTFSYEG
EAILTVGDGVGVGKVFHYVNGKFDYHQRVYKISDFKNYYGLLLFYYFSQNFLKETKKYSAKTSVDS
VRKDMIANMKVPRPIYIEQKKIGQFIKRVDNKTKIQKQVIELLKQRKKSLLQKMFIPGGSHHHHHH

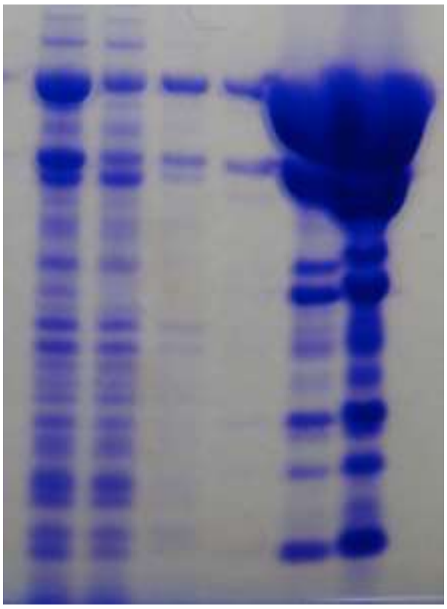

1- soluble cell extract, 2- Nickel column flow through, 3-Nickel column wash 1, 4- Nickel column wash 2, 5- Nickel column eluate, 6- Final protein after PD10 desalting and concentration

Although purified, this MTase was only used in SMRT.

Motifs

Motif	Modified Position	Modification Type	% Motifs Detected	# Of Motifs Detected	# Of Motifs In Genome	Mean Modification QV	Mean Motif Coverage	Partner Motif
TCAYNNNNNNTYTC	3	m6A	91.88	792	862	104.6	62.5	GARANNNNNNRTGA
GARANNNNNNRTGA	4	m6A	79.58	686	862	96.7	61.7	TCAYNNNNNNTYTC
CCACDNNNNNTYTC	3	m6A	15.87	30	189	90.4	65.5	
TCAGNNTNNNTNTCNB	3	m6A	15.03	29	193	91.0	67.7	
Not Clustered	0		0.00	129	9115220	85.4	67.9	

S.SauRE GARA-6-RTGA

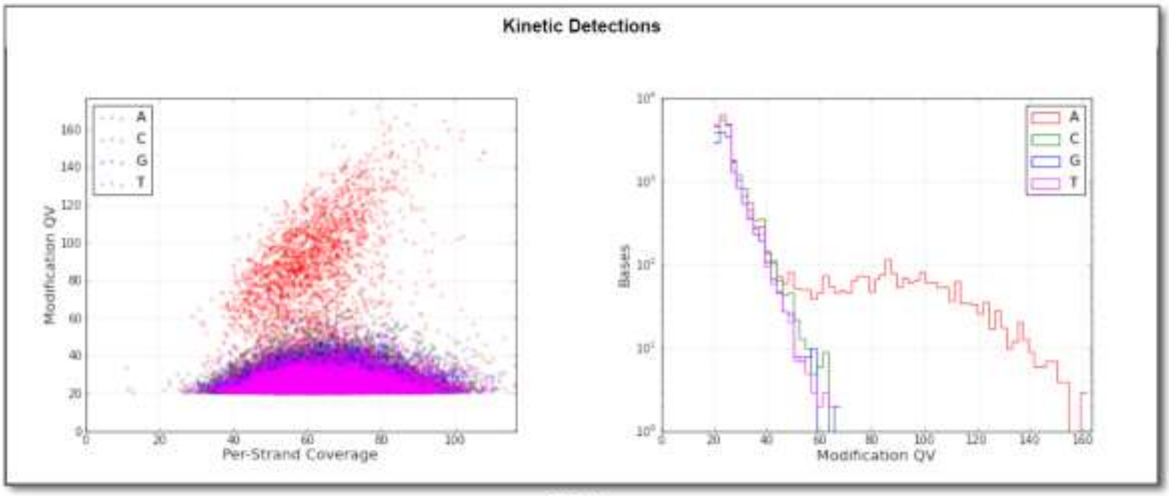

Motifs

Motif	Modified Position	Modification Type	% Motifs Detected	# Of Motifs Detected	# Of Motifs In Genome	Mean Modification QV	Mean Motif Coverage	Partner Motif
TCAYNNNNNNTYTC	3	m6A	91.88	792	862	104.6	62.5	GARANNNNNNRTGA
GARANNNNNNRTGA	4	m6A	79.58	686	862	96.7	61.7	TCAYNNNNNNTYTC
CCACDNNNNNTYTC	3	m6A	15.87	30	189	90.4	65.5	
TCAGNNTNNNTNTCNB	3	m6A	15.03	29	193	91.0	67.7	
Not Clustered	0		0.00	129	9115220	85.4	67.9	

Modification QV Histogram By Motif

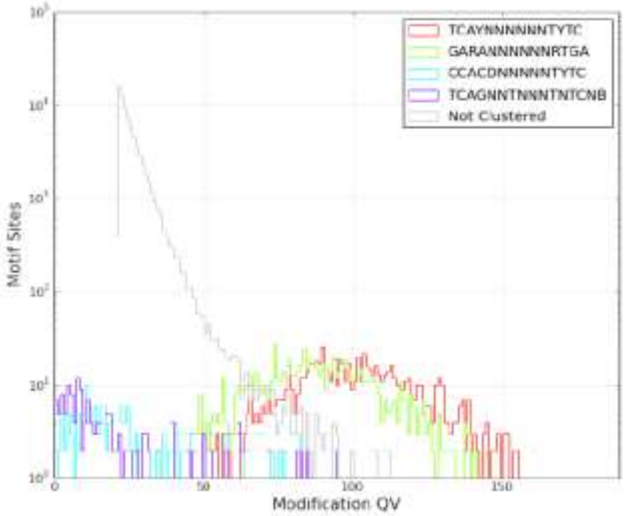

S.SauTE CAAG-5-RTGA

MSNTQKKNVPELRFPGFEGEWEEKELGEIFQIISGSTPLKSNKEFYENGNINWVKTTDLNNSKVTH
SKEKITEYAMKSLKLKLVPKNSVLIAMYGGFNQIGRTGLLKIDATINQAISALLMNHETNPEFIQA
FLNYQVKGWKRYAASSRKDPNITKKDIEQFKVPYVSINEQQKIGEFFSKIDHQIELEEQKLELLQQ
QKKGYMQKIFSQELRFKDENGKDYPEWEETTIKEIAQINTGKKDTKDAITNGSYDFYVRSPIVYKI
NTFSYEGEAILTVGDGVGVGKVFHYVNGKFDYHQRVYKISDFKNYYGLLLFYYFSQNFLKETKKYS
AKTSVDSVRKDMIANMKVPRPIYIEQKKIGQFIKRVDNKTKIQKQVIELLKQRKKSLLQKMFIPGG
SHHHHHH

1 2 3 4 5 6 7

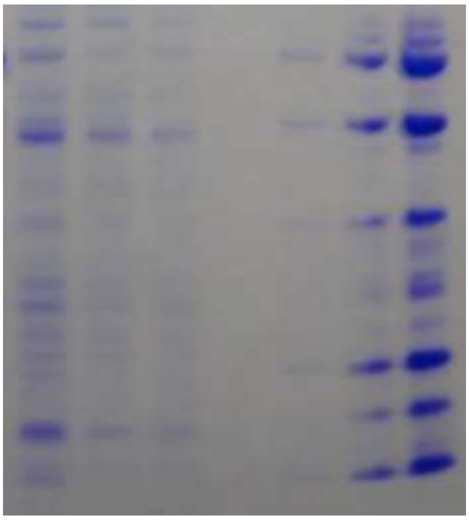

1- soluble cell extract 2- Nickel column flow through
3-Nickel column wash 1 4- Nickel column wash 2
5- Nickel column eluate 6- eluate after concentrating and PD10 step
7- Final concentrated protein

This MTase did not purify well and was only analysed by SMRT sequencing. The degeneracy in the target determined by SMRT sequencing can be removed using results from other systems.

Motifs

Motif	Modified Position	Modification Type	% Motifs Detected	# Of Motifs Detected	# Of Motifs In Genome	Mean Modification QV	Mean Motif Coverage	Partner Motif
CAAGNNNNNRTGR	3	m6A	80.15	214	267	63.8	42.7	
YCABNNNNNCTTG	3	m6A	55.08	260	472	65.1	43.1	
HTCAYNNNNNCMTG	4	m6A	47.41	238	502	51.7	44.4	
TCAYNNDNNCTVG	3	m6A	31.07	119	383	51.0	44.3	
CNAGNNNNRVTGA	3	m6A	28.62	170	594	59.5	43.6	
TCAYTNNNNDTTG	3	m6A	16.89	38	225	45.4	44.3	
Not Clustered	0		0.04	3962	9114883	35.8	50.6	

S.SauTE CAAG-5-RTGA

Modification QV Histogram By Motif

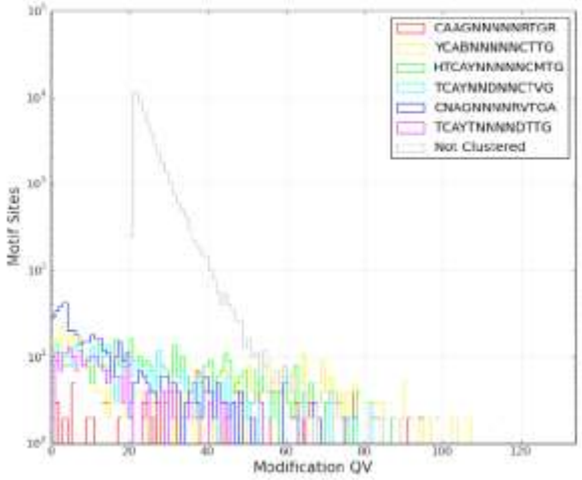

Kinetic Detections

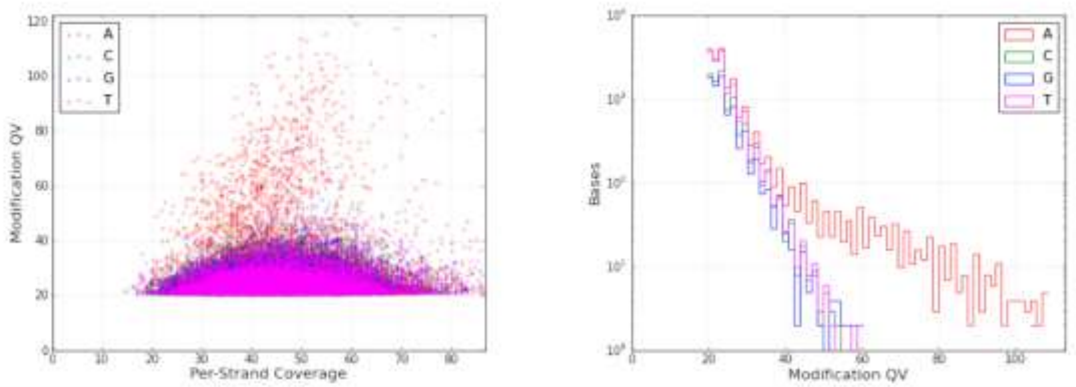

Motifs

Motif	Modified Position	Modification Type	% Motifs Detected	# Of Motifs Detected	# Of Motifs In Genome	Mean Modification QV	Mean Motif Coverage	Partner Motif
CAAGNNNNNRTGR	3	m6A	80.15	214	267	63.8	42.7	
YCABNNNNNCTTG	3	m6A	55.08	260	472	65.1	43.1	
HTCAYNNNNNCMTG	4	m6A	47.41	238	502	51.7	44.4	
TCAYNNDNNCTVG	3	m6A	31.07	119	383	51.0	44.3	
CNAGNNNNRVTGA	3	m6A	28.62	170	594	59.5	43.6	
TCAYTNNNNDTTG	3	m6A	16.89	38	225	45.4	44.3	
Not Clustered	0		0.04	3962	9114883	35.8	50.6	

S.SauVE   CNGA-6-RTGA

MSNTQKKNVPELRFPGFEGEWEEKELRELRNPKDKYSYTGGPFGSDLKKSDYTTDGIQIIQLQNIG
DGYFYNSNKVFTSNEKAEVLKSCNVFPGDIVIAKMADPIARAAIVPDNNIGKYLMASDGIRLSVDT
VHFNTKFVLECINRKSFRKKVEDNSSGSTRMRIGLSTLGSLTLKTTTLKEQQKIGQFFSKLDRQIE
LEEQKLELLQQQKKGYMQKIFSQELRFKDENGKDYPEWEETTIKEIAQINTGKKDTKDAITNGSYD
FYVRSPIVYKINTFSYEGEAILTVGDGVGVGKVFHYVNGKFDYHQRVYKISDFKNYYGLLLFYYFS
QNFLKETKKYSAKTSVDSVRKDMIANMKVPRPIYIEQKKIGQFIKRVDNKTKIQKQVIELLKQRKK
SLLQKMFIPGGSHHHHHH

1 2 3 4 5 6 7

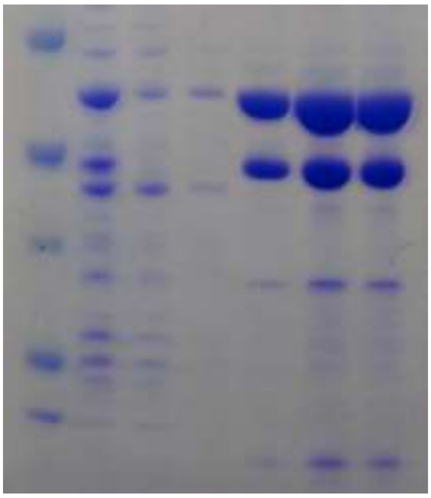

1- marker, 2- soluble cell extract, 3- Nickel column flow through, 4- Nickel column wash, 5- Nickel column eluate, 6- eluate after conc. and PD10 desalting, 7- Final protein after concentration

Although the MTase was purified, it was only analysed via SMRT sequencing.

Motifs

Motif	Modified Position	Modification Type	% Motifs Detected	# Of Motifs Detected	# Of Motifs In Genome	Mean Modification QV	Mean Motif Coverage	Partner Motif
TCAYNNNNNNTCNG	3	m6A	99.34	1354	1363	73.0	43.2	CNGANNNNNNRTGA
CNGANNNNNNRTGA	4	m6A	98.09	1337	1363	71.4	43.4	TCAYNNNNNNTCNG
TCAGBNNNNMTCNG	3	m6A	41.58	79	190	53.7	43.2	
VCCAYNNNNNVTCNG	4	m6A	33.12	211	637	50.8	44.7	CNGABNNNNNRTGGB
CNGABNNNNNRTGGB	4	m6A	29.98	191	637	50.2	45.8	VCCAYNNNNNVTCNG
CNGAYNDNNNCTGA	4	m6A	31.90	74	232	53.0	44.9	
HTCAYTNNNNNMCNG	4	m6A	23.31	131	562	44.6	45.5	
Not Clustered	0		0.05	4345	9112342	35.9	49.9	

S.SauVE CNGA-6-RTGA

Modification QV Histogram By Motif

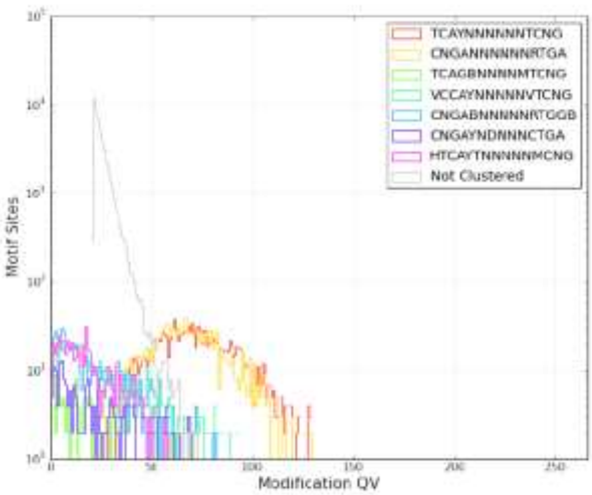

Kinetic Detections

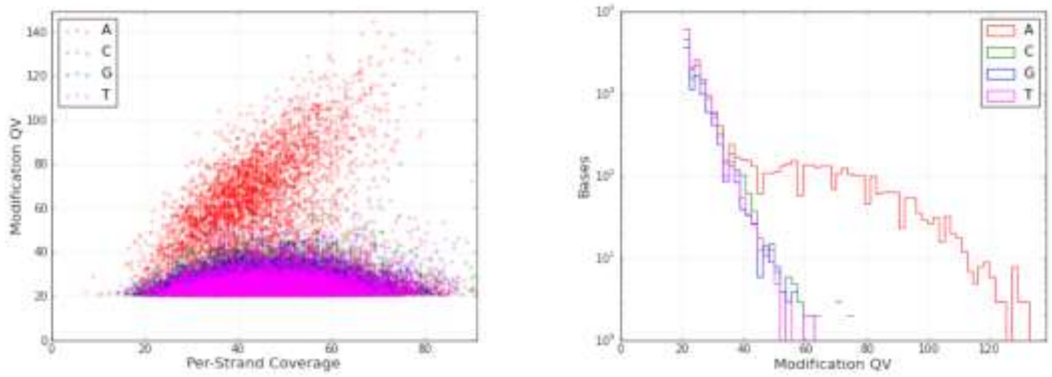

Motifs

Motif	Modified Position	Modification Type	% Motifs Detected	# Of Motifs Detected	# Of Motifs In Genome	Mean Modification QV	Mean Motif Coverage	Partner Motif
TCAYNNNNNNTCNG	3	m6A	99.34	1354	1363	73.0	43.2	CNGANNNNNNRTGA
CNGANNNNNNRTGA	4	m6A	98.09	1337	1363	71.4	43.4	TCAYNNNNNNTCNG
TCAGBNNNNMTCNG	3	m6A	41.58	79	190	53.7	43.2	
VCCAYNNNNNVTCNG	4	m6A	33.12	211	637	50.8	44.7	CNGABNNNNNRTGGB
CNGABNNNNNRTGGB	4	m6A	29.98	191	637	50.2	45.8	VCCAYNNNNNVTCNG
CNGAYNDNNNCTGA	4	m6A	31.90	74	232	53.0	44.9	
HTCAYTNNNNNMCNG	4	m6A	23.31	131	562	44.6	45.5	
Not Clustered	0		0.05	4345	9112342	35.9	49.9	

S.SauXE   TCTA-6-RTGA

MSNTQKKNVPELRFPGFEGEWEEKQFADFTKINQGLQIAINERKTEYSPELYFYITNEFLRPNSQT
KYFIENPPQSVIANKEDILMTRTGNTGKVVTNVFGAFHNNFFKIKFDKNLYDRLFLVEVLNSSKIQ
NKILSLAGSSTIPDLNHSDFYSISSSYPLLREQQKIGKFFSKLDRQIELEEQKLELLQQQKKGYMQ
KIFSQELRFKDENGKDYPEWEETTIKEIAQINTGKKDTKDAITNGSYDFYVRSPIVYKINTFSYEG
EAILTVGDGVGVGKVFHYVNGKFDYHQRVYKISDFKNYYGLLLFYYFSQNFLKETKKYSAKTSVDS
VRKDMIANMKVPRPIYIEQKKIGQFIKRVDNKTKIQKQVIELLKQRKKSLLQKMFIPGGSHHHHHH

1 2 3 4 5 6 7

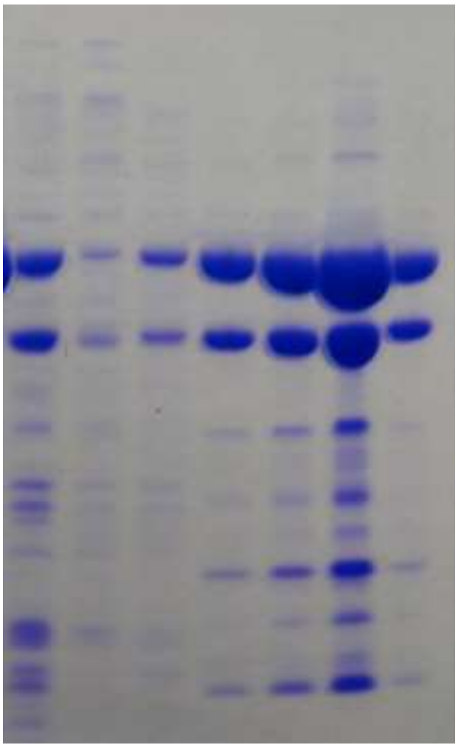

1- soluble cell extract 2- Nickel column flow through
3- Nickel column wash 4- Nickel column eluate
5- eluate after conc. and PD10 desalting
6- final protein after concentration
7- CC398-1 purified protein marker

DNA cleavage assay.

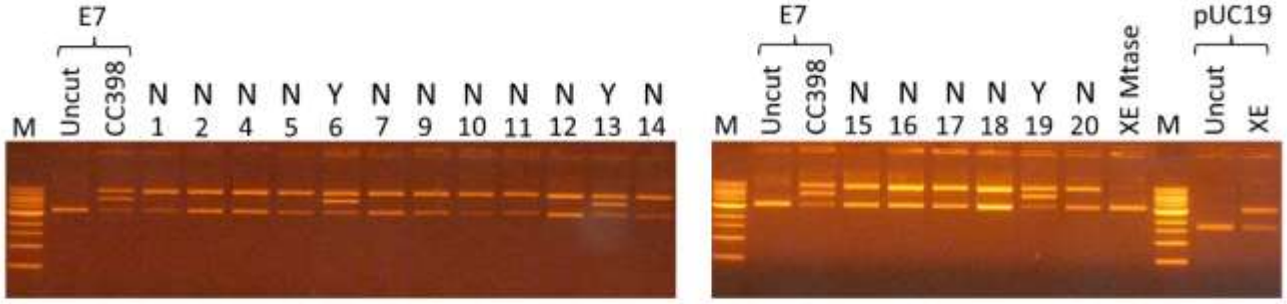

S.SauXE   TCTA-6-RTGA

The degeneracy in the target determined by SMRT sequencing can be resolved by reference to targets from other systems.

Motifs

Motif	Modified Position	Modification Type	% Motifs Detected	# Of Motifs Detected	# Of Motifs In Genome	Mean Modification QV	Mean Motif Coverage	Partner Motif
YCABNNNNNNTAGA	3	m6A	68.77	196	285	121.6	104.1	TCTANNNNNNVTGR
TCTANNNNNNVTGR	4	m6A	68.07	194	285	120.0	106.6	YCABNNNNNNTAGA
DNNTCAYNNNNNNTAGY	6	m6A	44.57	82	184	79.3	103.0	
RCTANNNNNNRTGA	4	m6A	40.41	99	245	71.8	104.1	
HTCAHNNNNNNTVGA	4	m6A	32.43	156	481	103.9	104.2	
HTCAYNNNNNNVAGA	4	m6A	32.07	211	658	67.2	107.8	
Not Clustered	0		0.12	10813	9115188	38.1	115.4	

Modification QV Histogram By Motif

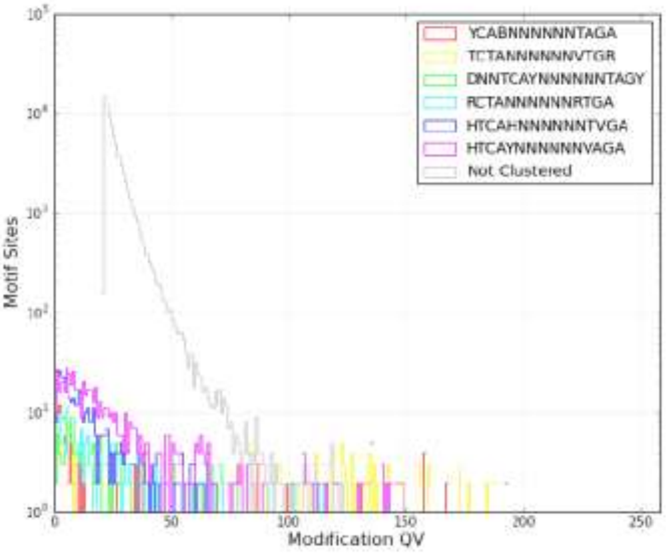

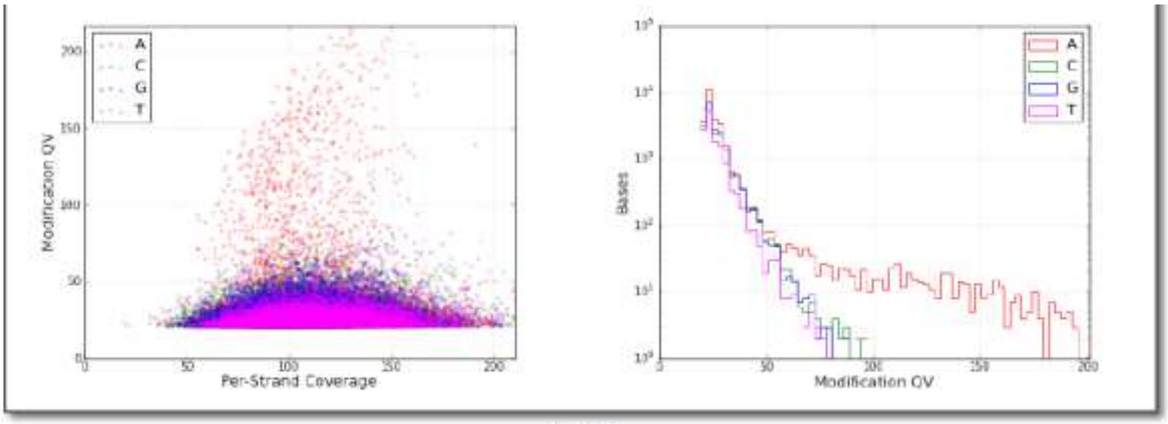

Motifs

Motif	Modified Position	Modification Type	% Motifs Detected	# Of Motifs Detected	# Of Motifs In Genome	Mean Modification QV	Mean Motif Coverage	Partner Motif
YCABNNNNNNTAGA	3	m6A	68.77	196	285	121.6	104.1	TCTANNNNNNVTGR
TCTANNNNNNVTGR	4	m6A	68.07	194	285	120.0	106.6	YCABNNNNNNTAGA
DNNTCAYNNNNNNTAGY	6	m6A	44.57	82	184	79.3	103.0	
RCTANNNNNNRTGA	4	m6A	40.41	99	245	71.8	104.1	
HTCAHNNNNNNTVGA	4	m6A	32.43	156	481	103.9	104.2	
HTCAYNNNNNNVAGA	4	m6A	32.07	211	658	67.2	107.8	
Not Clustered	0		0.12	10813	9115188	38.1	115.4	

S.SauZE GAC-5-RTGA

MSNTQKKNVPELRFPGFEGEYSLDIFGNLATNKSEKFNPQNENASIDIELDCIEQNTGRLIKIYNS
KEFSSQKNKFNPQNVLYGKLRPYLNKYYFTKKSGVCSSEIWVLKSTKEDKLLNLFLYYFIQTKRYS
DVASKSAGSKMPRADWGLIENIRVYFPELCEQQKIGQFFSKLDRQIELEEQKLELLQQQKKGYMQK
IFSQELRFKDENGKDYPEWEETTIKEIAQINTGKKDTKDAITNGSYDFYVRSPIVYKINTFSYEGE
AILTVGDGVGVGKVFHYVNGKFDYHQRVYKISDFKNYYGLLLFYYFSQNFLKETKKYSAKTSVDSV
RKDMIANMKVPRPIYIEQKKIGQFIKRVDNKTKIQKQVIELLKQRKKSLLQKMFIPGGSHHHHHH*

1 2 3 4 5 6 7 8

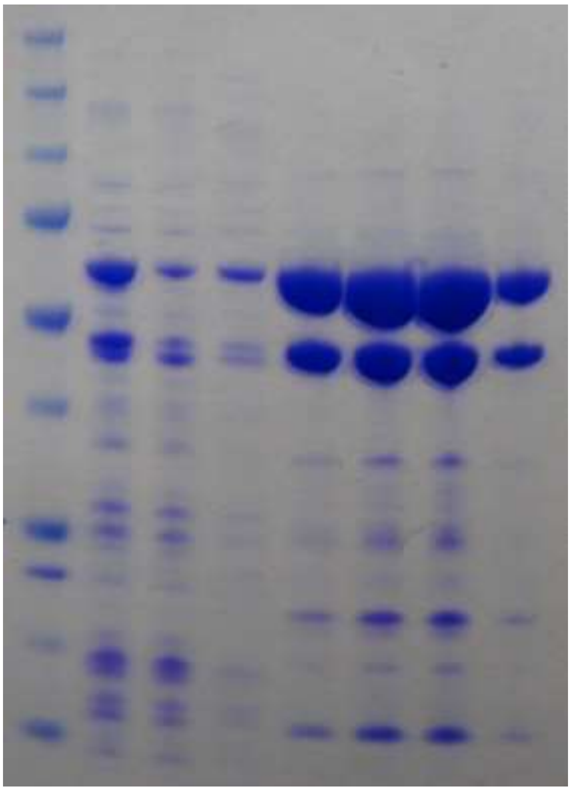

1- marker 2- soluble cell extract 3- Nickel column flow through
4- Nickel column wash 5- Nickel column eluate 6- eluate after conc. step and PD10 desalting
7- final concentrated protein 8- CC398-1 purified protein marker

DNA cleavage assay showed cutting of all plasmids so the ATPase assay was used given that we knew the individual TRD specificities.

**Oligonucleotide name**	**DNA sequence (5' to 3')**
ZE5for	AGATGATGGAATCAATGCGACTTCCAGTGAGCCCTATACGATATAA
ZE5rev	TTATATCGTATAGGGCTCACTGGAAGTCGCATTGATTCCATCATCT
ZE6for	AGATGATGGAATCAATGCGACTTCCATGTGAGCCCTATACGATATAA
ZE6rev	TTATATCGTATAGGGCTCACATGGAAGTCGCATTGATTCCATCATCT
ZE7for	AGATGATGGAATCAATGCGACTTCACATGTGAGCCCTATACGATATAA
ZE7rev	TTATATCGTATAGGGCTCACATGTGAAGTCGCATTGATTCCATCATCT

S.SauZE   GAC-5-RTGA

N=5 gives the clearest signal.

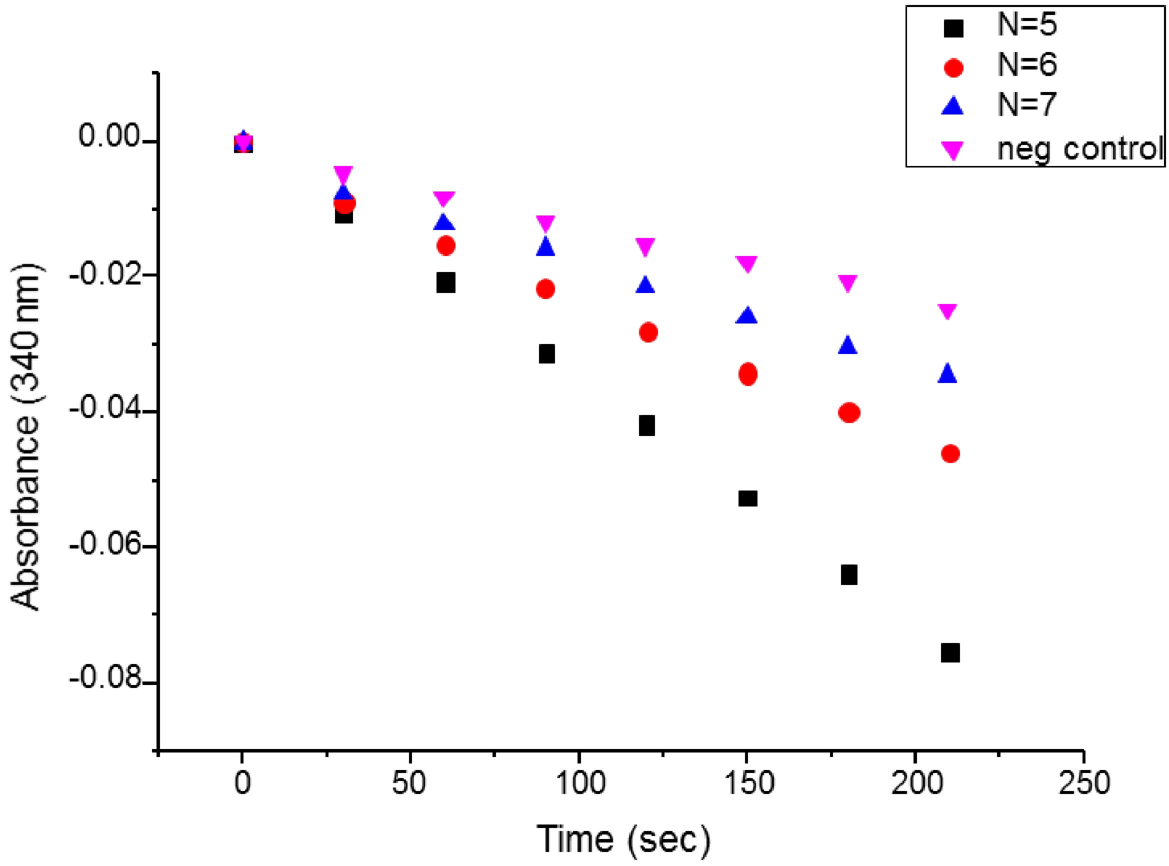

S.SauZS   GAC-6-TGC

MSNTQKKNVPELRFPGFEGEYSLDIFGNLATNKSEKFNPQNENASIDIELDCIEQNTGRLIKIYNS
KEFSSQKNKFNPQNVLYGKLRPYLNKYYFTKKSGVCSSEIWVLKSTKEDKLLNLFLYYFIQTKRYS
DVASKSAGSKMPRADWGLIENIRVYFPELCEQQKIGQFFSKLDRQIELEEQKLELLQQQKKGYMQK
IFSQELRFKDENGNDYPDWTNERLGEVTTVTMGQSPKSVNYTDNSNDTVLIQGNADIENGLINPRI
YTREVTKLIQKDEIILTVRAPVGKLAMAQINACIGRGVCSIKGDKFLYYFLEWFATQNKWIRFSQG
STFESISGNDIRNIHIKIPVEDERTKIIKLLNSLDVLNSKTDLKIQNLKQRKQSLLQKIFVPGGSH
HHHHH*

1 2 3 4 5 6 7 8 9

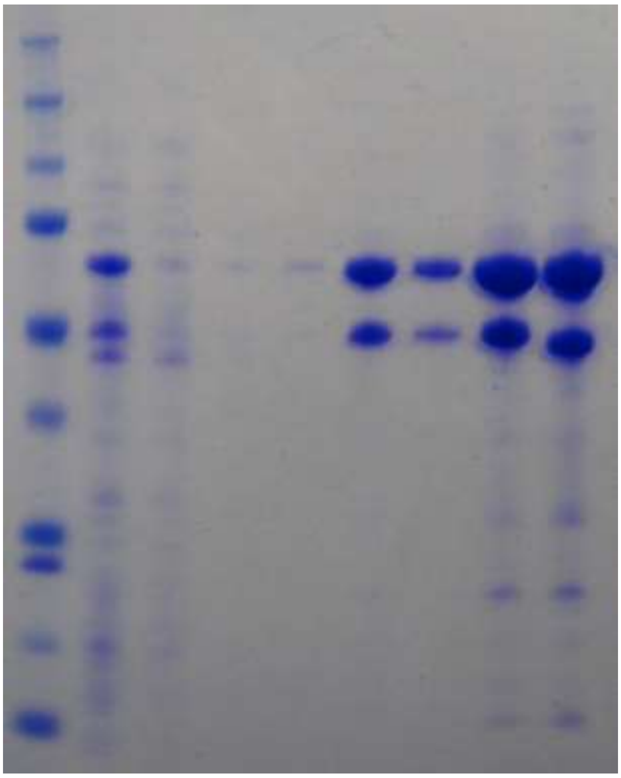

1- marker   2- soluble cell extract   3- Nickel column flow through
4- Nickel column wash 1   5- Nickel column wash 2   6- Nickel column eluate
7- eluate after PD10 desalting   8- final protein after concentration
9- NQ purified protein marker

The DNA cleavage assay showed cutting of all plasmids so the ATPase assay was used since we knew the TRD specificities.

**Oligonucleotide name**	**DNA sequence (5' to 3')**
ZS6for	AGATGATGGAATCAATGCGACTTCCATTGCGCCCTATACGATATAA
ZS6rev	TTATATCGTATAGGGCGCAATGGAAGTCGCATTGATTCCATCATCT
ZS7for	AGATGATGGAATCAATGCGACTTCACATTGCGCCCTATACGATATAA
ZS7rev	TTATATCGTATAGGGCGCAATGTGAAGTCGCATTGATTCCATCATCT

S.SauZS GAC-6-TGC

N=6 gives the greatest activity.

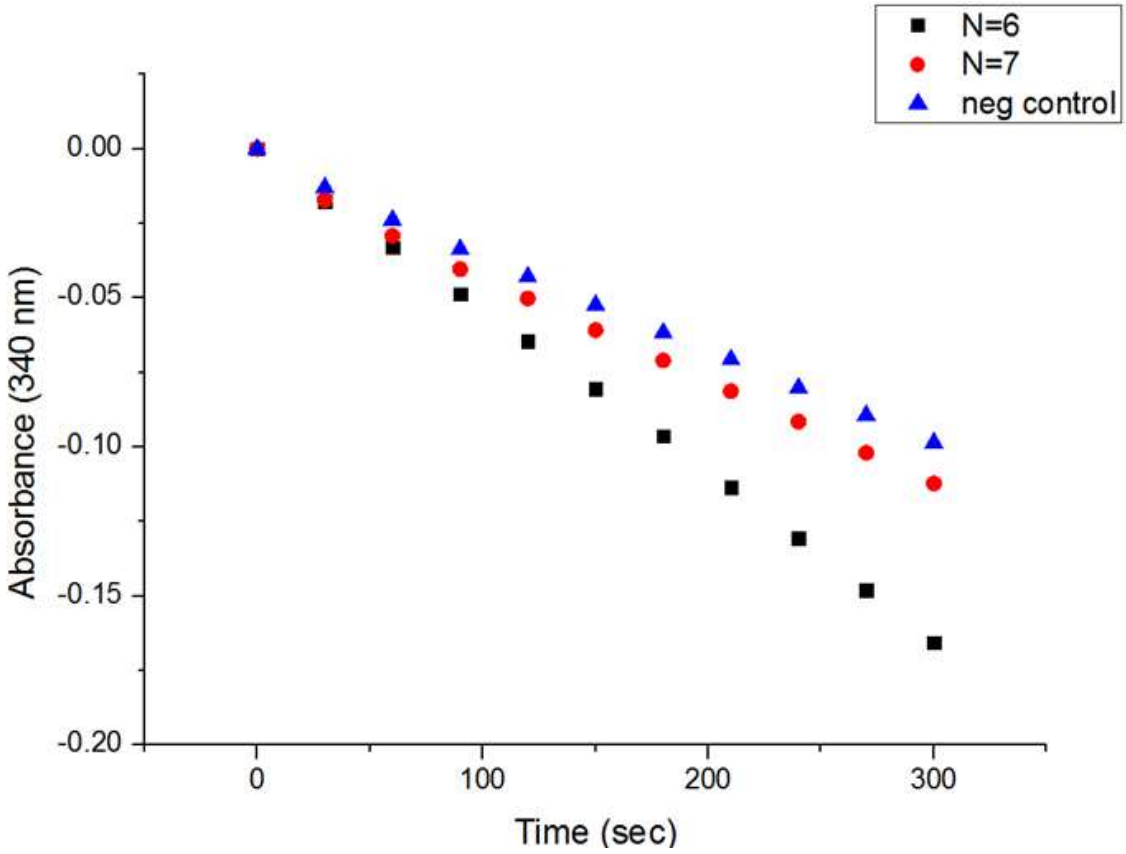

S.Saub*E GGHA-6-RTGA

MSNTQKKNVPELRFPGFEGEWEEKKLEDTLEFIKDGTHGTHENVNNGPWLLSAKNIKNNKIIISSD
DRKISESDYKKIYKNYKLEKGDLLLTIVGTIGRAAIVKNPNNIAFQRSVAILKTKATYDVGFIFQL
FQTKYFKNLLLRKQVVSAQPGLYLGDIRKIKISITNIIEEQRKIGIFFSKLDRQIELEEQKLELLQ
QQKKGYMQKIFSQELRFKDENGKDYPEWEETTIKEIAQINTGKKDTKDAITNGSYDFYVRSPIVYK
INTFSYEGEAILTVGDGVGVGKVFHYVNGKFDYHQRVYKISDFKNYYGLLLFYYFSQNFLKETKKY
SAKTSVDSVRKDMIANMKVPRPIYIEQKKIGQFIKRVDNKTKIQKQVIELLKQRKKSLLQKMFIPG
GSHHHHHH

1 2 3 4 5 6

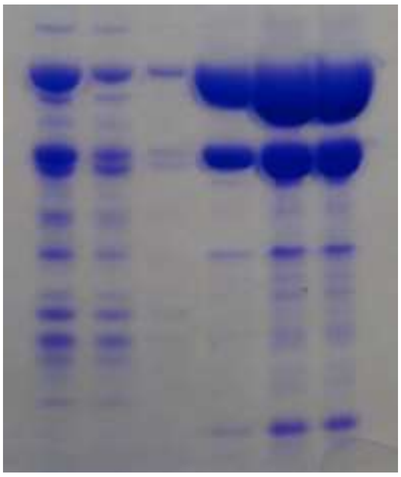

1- soluble cell extract, 2- Nickel column flow through, 3- Nickel column wash, 4- Nickel column eluate, 5- eluate after conc. step and PD10 desalting, 6- Final concentrated protein

Although this MTase was purified, it was only used in the SMRT sequencing assay.

Motifs

Motif	Modified Position	Modification Type	% Motifs Detected	# Of Motifs Detected	# Of Motifs In Genome	Mean Modification QV	Mean Motif Coverage	Partner Motif
TCAYNNNNNNTDCC	3	m6A	77.39	989	1278	44.6	24.6	GGHANNNNNNRTGA
GGHANNNNNNRTGA	4	m6A	67.68	865	1278	43.2	24.7	TCAYNNNNNNTDCC
TCAGNDNNNNTDCC	3	m6A	21.57	110	510	41.0	25.2	
Not Clustered	0		0.01	928	9114260	36.7	27.3	

S.Saub*E GGHA-6-RTGA

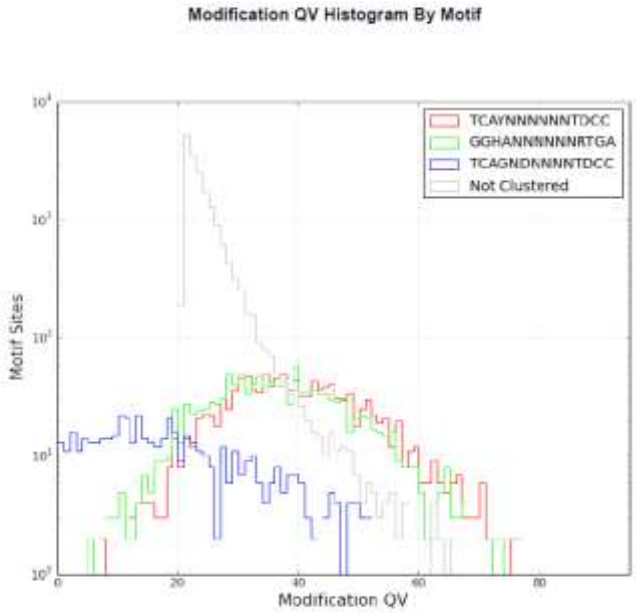

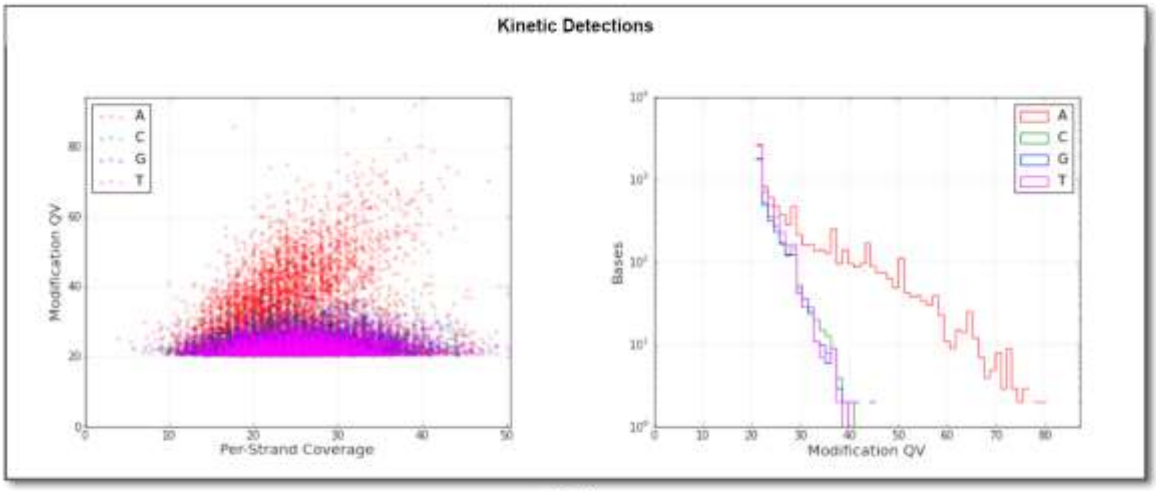

Motifs

Motif	Modified Position	Modification Type	% Motifs Detected	# Of Motifs Detected	# Of Motifs In Genome	Mean Modification QV	Mean Motif Coverage	Partner Motif
TCAYNNNNNNTDCC	3	m6A	77.39	989	1278	44.6	24.6	GGHANNNNNNRTGA
GGHANNNNNNRTGA	4	m6A	67.68	865	1278	43.2	24.7	TCAYNNNNNNTDCC
TCAGNDNNNNTDCC	3	m6A	21.57	110	510	41.0	25.2	
Not Clustered	0		0.01	928	9114260	36.7	27.3	

S.Saue*E GAG-6-RTGA

MSNTQKKNVPELRFPGFEGEWEEKSISSFLKESKIKGSNGSHAKKLTVKLWGKGVVPKKETFKGSD
NTQYYKRKAGQLMYGKLDFLNCAFGIVPDSLNNYESTIDSPSFDFINGDSKFLLERIKLKSFYKKF
GDIANGSRKAKRINQDTFLSLPVFAPKYDEQLRIGEFFSKLDRQIELEEQKLELLQQQKKGYMQKI
FSQELRFKDENGKDYPEWEETTIKEIAQINTGKKDTKDAITNGSYDFYVRSPIVYKINTFSYEGEA
ILTVGDGVGVGKVFHYVNGKFDYHQRVYKISDFKNYYGLLLFYYFSQNFLKETKKYSAKTSVDSVR
KDMIANMKVPRPIYIEQKKIGQFIKRVDNKTKIQKQVIELLKQRKKSLLQKMFIPGGSHHHHHH

1 2 3 4 5 6

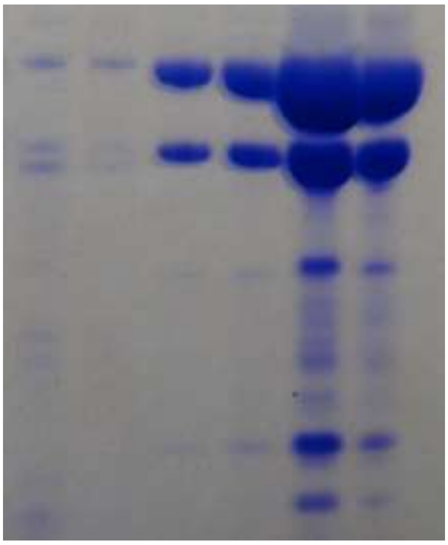

1- soluble cell extract, 2- Nickel column flow through, 3- Nickel column eluate, 4- eluate after PD10 desalting, 5- Final concentrated protein, 6- RE purified protein as marker

DNA cleavage assay.

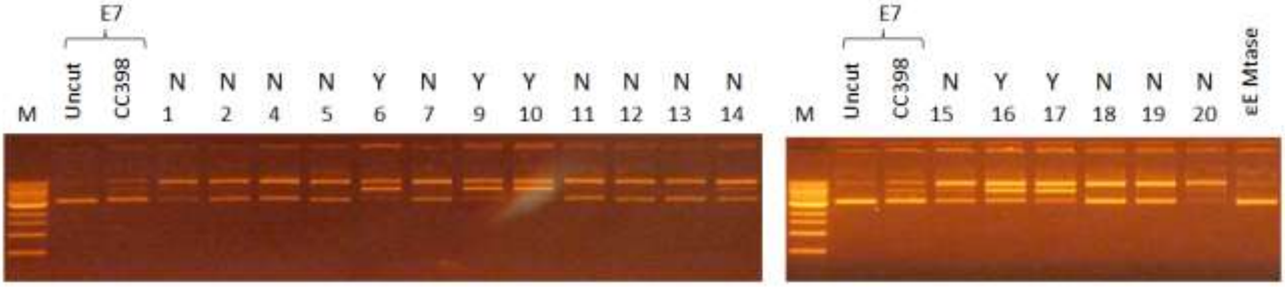

S.Saue*E GAG-6-RTGA

Motifs

Motif	Modified Position	Modification Type	% Motifs Detected	# Of Motifs Detected	# Of Motifs In Genome	Mean Modification QV	Mean Motif Coverage	Partner Motif
TCAYNNNNNNCTC	3	m6A	88.60	762	860	49.4	28.5	GAGNNNNNNRTGA
GAGNNNNNNRTGA	2	m6A	87.33	751	860	50.0	28.2	TCAYNNNNNNCTC
GAGNDNNNNGTGGB	2	m6A	20.22	37	183	40.9	29.5	
DNNGAGNDNNNNGAGA	5	m6A	18.56	36	194	39.8	32.7	
Not Clustered	0		0.01	914	9115229	34.6	36.3	

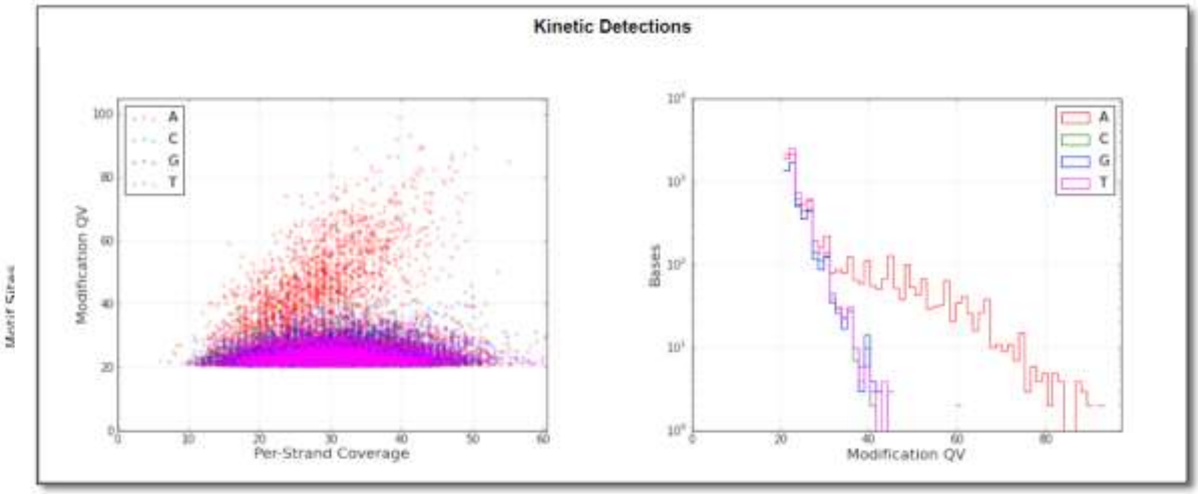

Motifs

Motif	Modified Position	Modification Type	% Motifs Detected	# Of Motifs Detected	# Of Motifs In Genome	Mean Modification QV	Mean Motif Coverage	Partner Motif
TCAYNNNNNNCTC	3	m6A	88.60	762	860	49.4	28.5	GAGNNNNNNRTGA
GAGNNNNNNRTGA	2	m6A	87.33	751	860	50.0	28.2	TCAYNNNNNNCTC
GAGNDNNNNGTGGB	2	m6A	20.22	37	183	40.9	29.5	
DNNGAGNDNNNNGAGA	5	m6A	18.56	36	194	39.8	32.7	
Not Clustered	0		0.01	914	9115229	34.6	36.3	

SUPPLEMENTARY INFORMATION FOR TABLE 4.

S.SauAc* CCAY-6-RTC

The Ac* TRD combination is found in CC97-1. The MTase was not purified but instead used to methylate the genome of E. coli ER2796 for SMRT analysis. The target is CCAYNNNNNNRTC. There are a few minor amino acid differences in the S.SauAc* between members of CC97.

CC97

Recombinant S.SauAc* CC97-1

MSNTQKKNVPELRFPGFEGEWEEKKLGDLTTKIGSGKTPKGGSENYTNKGIPFLRSQNIRNGKLNLNDLVYISKDIDDEM
KNSRTYYGDVLLNITGASIGRTAINSIVEIHANLNQHVCIIRLKKEYYYNFFGQYLLSRKGKRKIFLAQSGGSREGLNFK
EIANLKIFTPTIFEEQQKIGEFISKLDRQIELEEQKLELLQQQKKGYLQKIFSQELRFKDENGNDYPEWRFARFKDFMYK
PINIRPAINISKSELLTVKLHCKGIEKANINRVLKLGATNYYKRFEGQFIYGKQNFFNGAFDIVPKKFDGLYSSSDVPAF
EINTEKIEPNYFISYISRPSFYKSKEKYSTGTGSKRIHENTVLNFSLHLPCLNEQLKIASFVCFLNRKIELLERKIYLIK
KQKQALLQQMFIPGGSHHHHHH

Wild Type S.SauAc*

MSNTQKKNVPELRFPGFEGEWEEKQLGDLTTKIGSGKTPKGGSENYTNKGIPFLRSQNIRNGKLNLNDLVYISKDIDDEM
KNSRTYYGDVLLNITGASIGRTAINSIVETHANLNQHVCIIRLKKEYYYIFFGQYLLSRKGKRKIFLAQSGGSREGLNFK
EIANLKIFTPTIFEEQQKIGKFFSKLDRQIELEEQKLELLQQQKKGYLQKIFSQELRFKDENGNDYPEWRFARFKDFMYK
PINIRPAINISKSELLTVKLHCKGIEKANINRVLKLGATNYYKRFEGQFIYGKQNFFNGAFDIVPKKFDGLYSSSDVPAF
EINTEKIEPNYFISYISRPSFYKSKEKYSTGTGSKRIHENTVLNFSLHLPCLNEQLKIASFVCFLNRKIELLERKIYLIK
KQKQALLQQMFI*

SMRT Cells: 1 Movies: 1

Motif Summary

Motifs	Modified Position	Type	% Motifs Detected	# Of Motifs Detected	# Of Motifs In Genome	Mean Modification QV	Mean Motif Coverage	Partner Motif
CCAYNNNNNNRTC	3	m6A	97.52%	2199	2255	68.95	50.78	GAYNNNNNNRTGG
GAYNNNNNNRTGG	2	m6A	96.01%	2165	2255	68.50	51.01	CCAYNNNNNNRTC

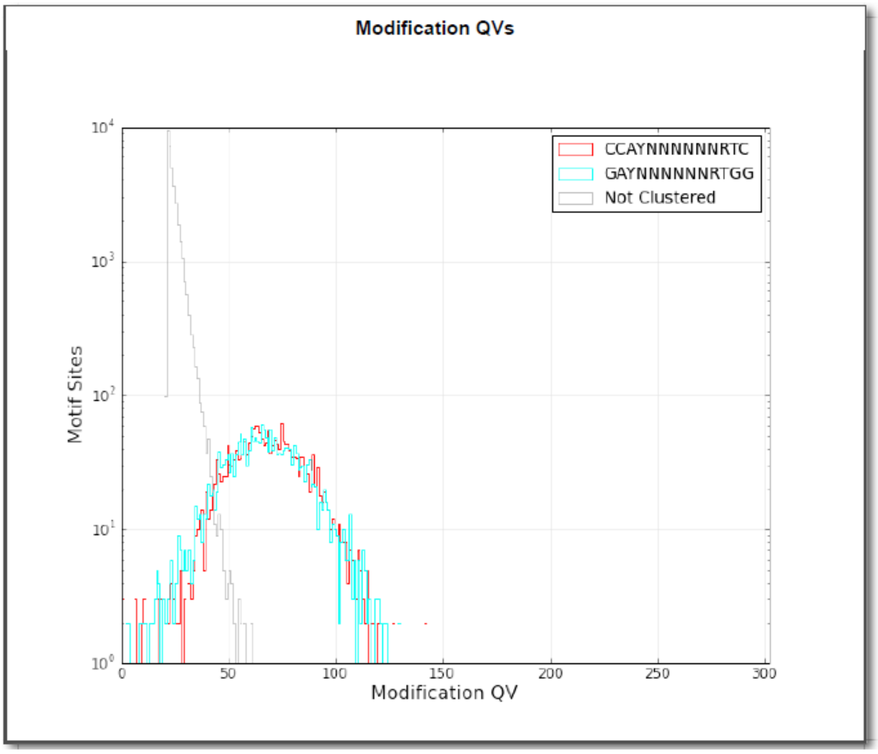

S.SauBI-EGFP

CC22-1 AGG-6-TGAR

This MTase was expressed and purified as a fusion with EGFP.
Nuclease assays and SMRT analysis gave the same target site.

MSNTQKKNVPELRFPGFEGEWEEKKLGDLTDRVIRKNKNLESKKPLTISGQLGLIDQTEYFSKSVS
SKNLENYTLIKNGEFAYNKSYSNGYPLGAIKRLTRYDSGVLSSLYICFSIKSEMSKDFMEAYFDST
HWYREVSGIAVEGARNHGLLNVSVNDFFTILIKYPSLEEQQKIGKFFSKLDRQIELEEQKLELLQQ
QKKGYMQKIFSQELRFKNENGNDYPDWERIKFFDVIDKVIDFRGRTPKKLNMEWSDEGYLALSAVN
VKKGYIDFNVEAKYGNLDLYTRWMRGNELYKGQVLFTTEAPMGNVAQVPDNKGYILSQRTIAFNSN
EKITDNFLASLLSSENVYNDLLKLCSGATAKGVSQKNLNRLYVTIPHSISEQEEIAEFFRKINQLV
ELQKYKIEHTKSQKQVFLQKMFIGSMVSKGEELFTGVVPILVELDGDVNGHKFSVSGEGEGDATYG
KLTLKFICTTGKLPVPWPTLVTTLTYGVQCFSRYPDHMKQHDFFKSAMPEGYVQERTIFFKDDGNY
KTRAEVKFEGDTLVNRIELKGIDFKEDGNILGHKLEYNYNSHNVYIMADKQKNGIKVNFKIRHNIE
DGSVQLADHYQQNTPIGDGPVLLPDNHYLSTQSALSKDPNEKRDHMVLLEFVTAAGITLGMDELYK
HHHHHH

1 2 3 4 5 6 7 8 9 10 11 12 13 14 15

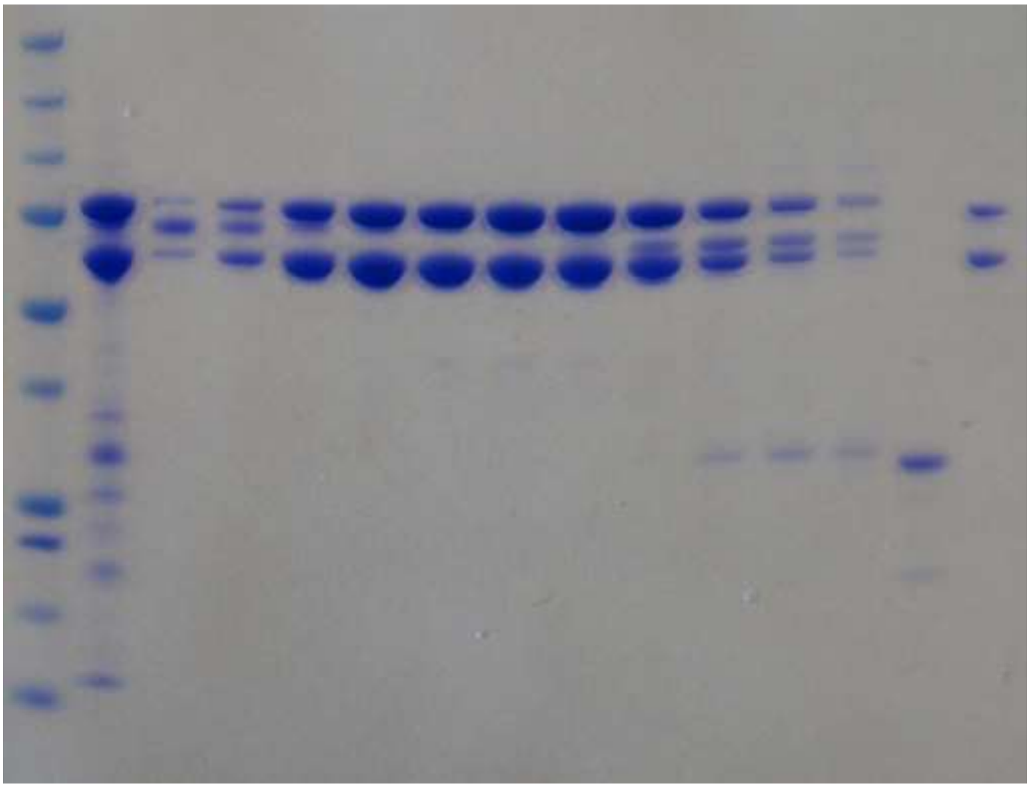

1- marker 2- Nickel column eluate 3-14 Fractions from gel filtration column
15- CC5-1 Purified protein marker

S.SauBI-EGFP
CC22-1   AGG-6-TGAR

DNA cleavage assay

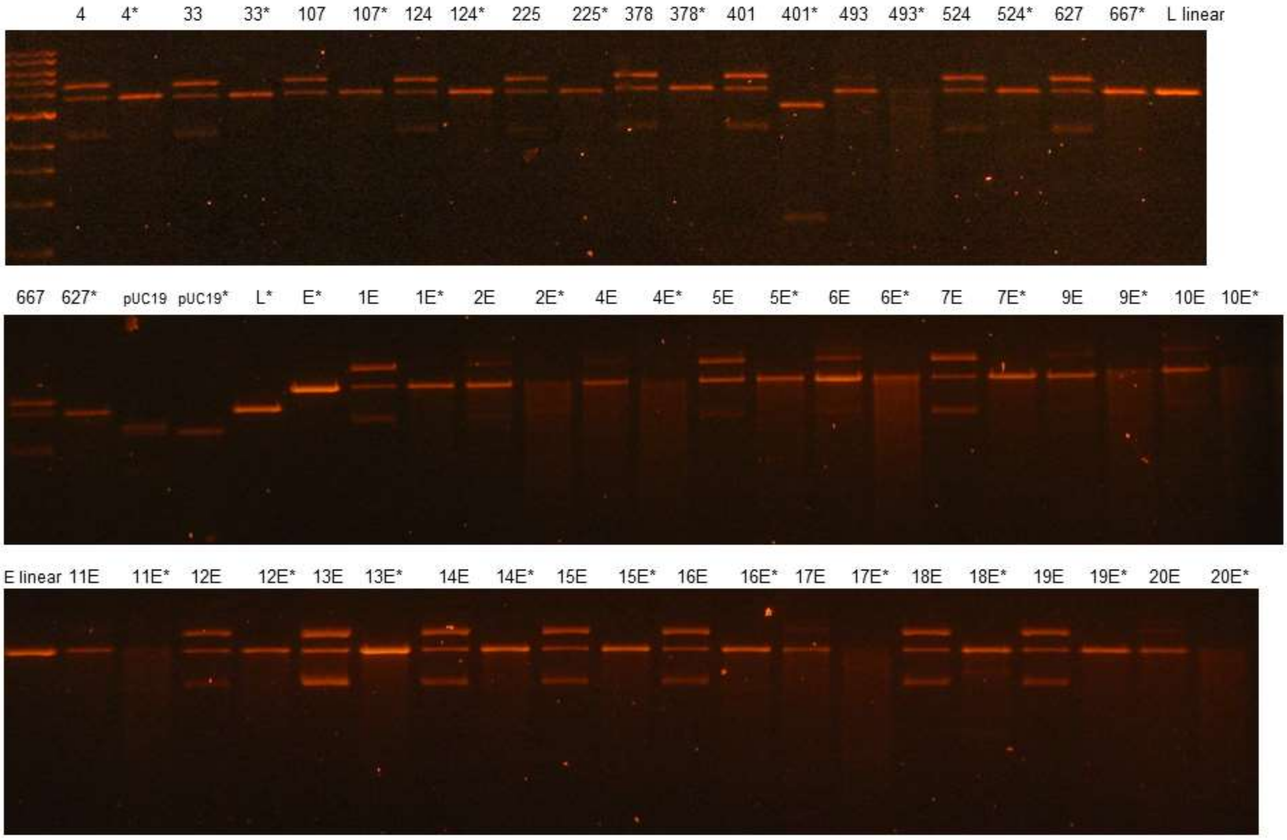

S.SauBI-EGFP
CC22-1 AGG-6-TGAR

Motif	Modified Position	Modification Type	% Motifs Detected	# Of Motifs Detected	# Of Motifs In Genome	Mean Modification QV	Mean Motif Coverage	Partner Motif
YTCANNNNNNCCT	4	m6A	99.24	919	926	86.2	56.3	AGGNNNNNNTGAR
AGGNNNNNNTGAR	1	m6A	99.24	919	926	83.9	55.7	YTCANNNNNNCCT
Not Clustered	0		0.06	5230	9124356	34.8	61.5	

Modification QV Histogram By Motif

Modification QV Histogram

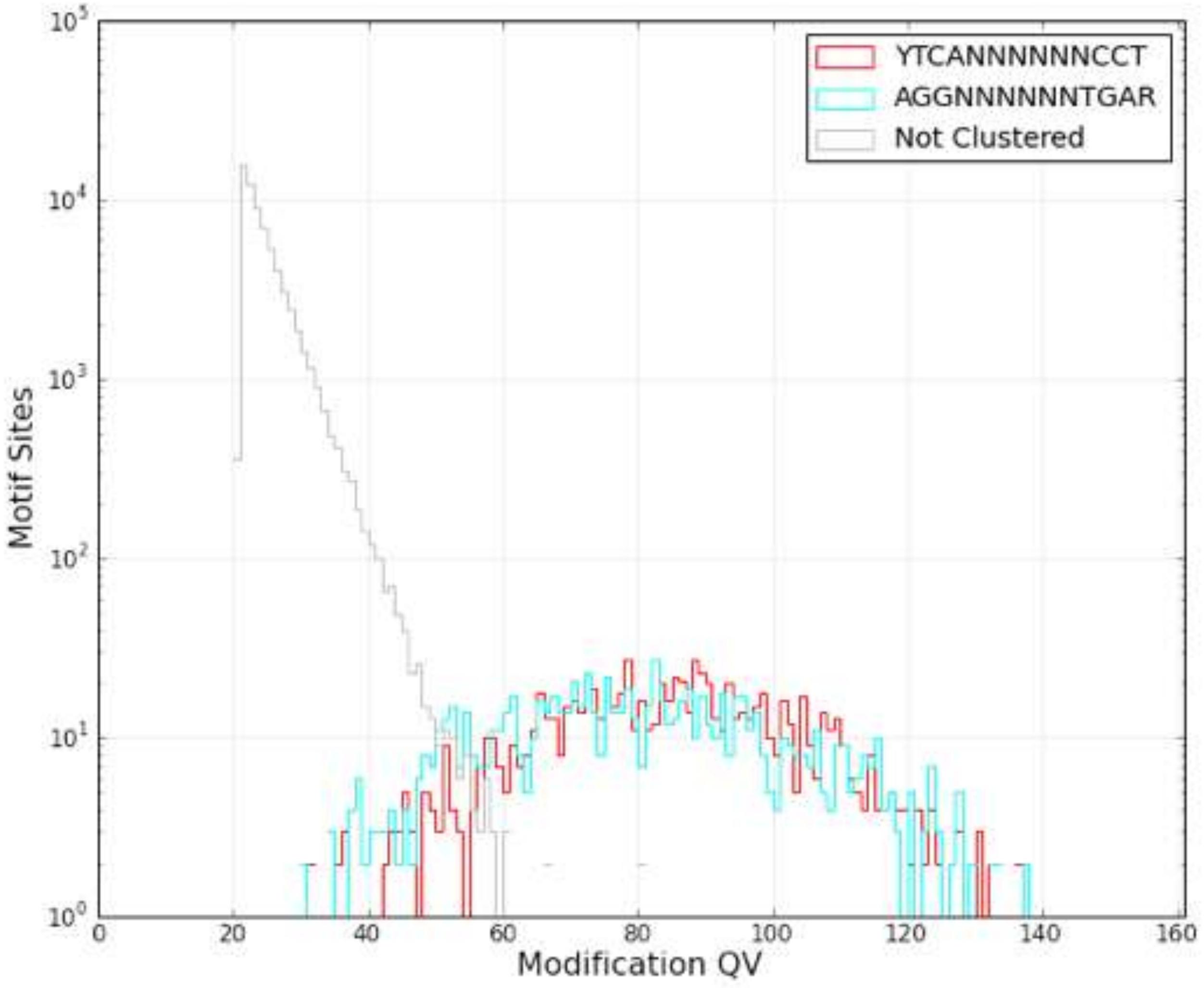

S.SauCE
ST425-1 GWAG-5-RTGA
The recombinant enzyme with TRDs C and E was purified and used in the nuclease assay. There are minor differences in amino acid sequence between members of ST425-1.
Recombinant S.SauCE CC425-1 GWAG-5-RTGA

MSNTQKKNVPELRFPGFEGEWEEKKVGELLEFKNGLNKGKEYFGSGSSIVNFKDVFNNRSINTNNL
TGKVNVNSKELKNYSVEKGDVFFTRTSEVIGEIGYPSVILNDPENTVFSGFVLRGRPKSGIDLINN
NFKRYVFFTNSFRKEMITKSSMTTRALTSGTAINRMKVIYPVSAKEQKKIGDFFSKLDRQIELEEQ
KLELLQQQKKGYMQKIFSQELRFKDENGKDYPEWEETTIKEIAQINTGKKDTKDAITNGSYDFYVR
SPIVYKINTFSYEGEAILTVGDGVGVGKVFHYVNGKFDYHQRVYKISDFKNYYGLLLFYYFSQNFL
KETKKYSAKTSVDSVRKDMIANMKVPRPIYIEQKKIGQFIKRVDNKTKIQKQVIELLKQRKKSLLQ
KMFIPGGSHHHHHH

Wild type S.SauCE

MSNTQTKNVPELRFPGFEGEWEEKQVGELLEFKNGLNKGKEYFGSGSSIVNFKDVFNNRSINTNNL
TGKVNVNSKELKNYSVEKGDVFFTRTSEVIGEIGYPSVILNDPENTVFSGFVLRGRPKSGIDLINN
NFKRYVFFTNSFRKEMITKSSMTTRALTSGTAINKMKVIYPVSAKEQKKIGDFFSKLDRQIELEEQ
KLELLQQQKKGYMQKIFTQELRFKDENGNDYPEWEETTIKEIAQINTGKKDTKDAITNGSYDFYVR
SPIVYKINTFSYEGEAILTVGDGVGVGKVFHYVNGKFDYHQRVYKISDFKNYYGLLLFYYFSQNFL
KETKKYSAKTSVDSVRKDMVANMKVPRPIYIEQEKIGQFIKKVDNKIKIQKQVIELLKQRKKALLQ
KMFI*

1 2 3 4 5 6 7 8 9 10 11 12 13 14 15

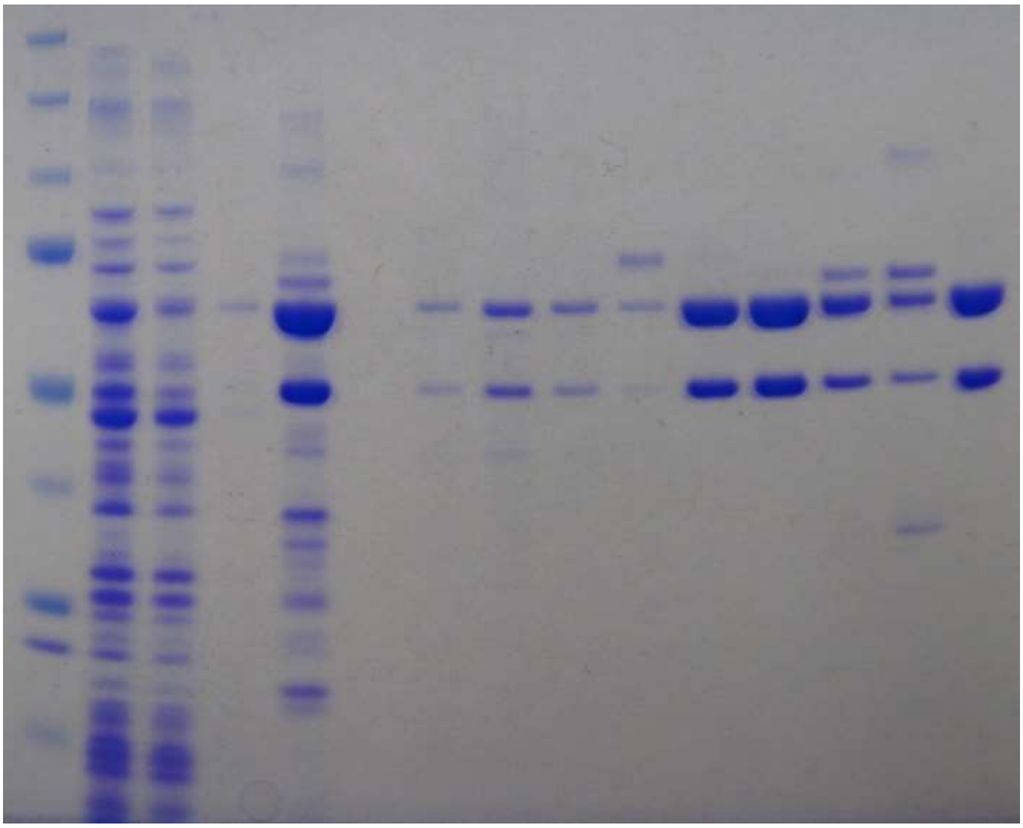

1- marker 2- soluble cell extract 3- flow through from Nickel column 4- wash from Nickel column
5- eluate from Nickel column 6-14 Fractions from gel filtration column 15- CC398-1 purified protein marker

S.SauCE
ST425-1 GWAG-5-RTGA

DNA cleavage assay.

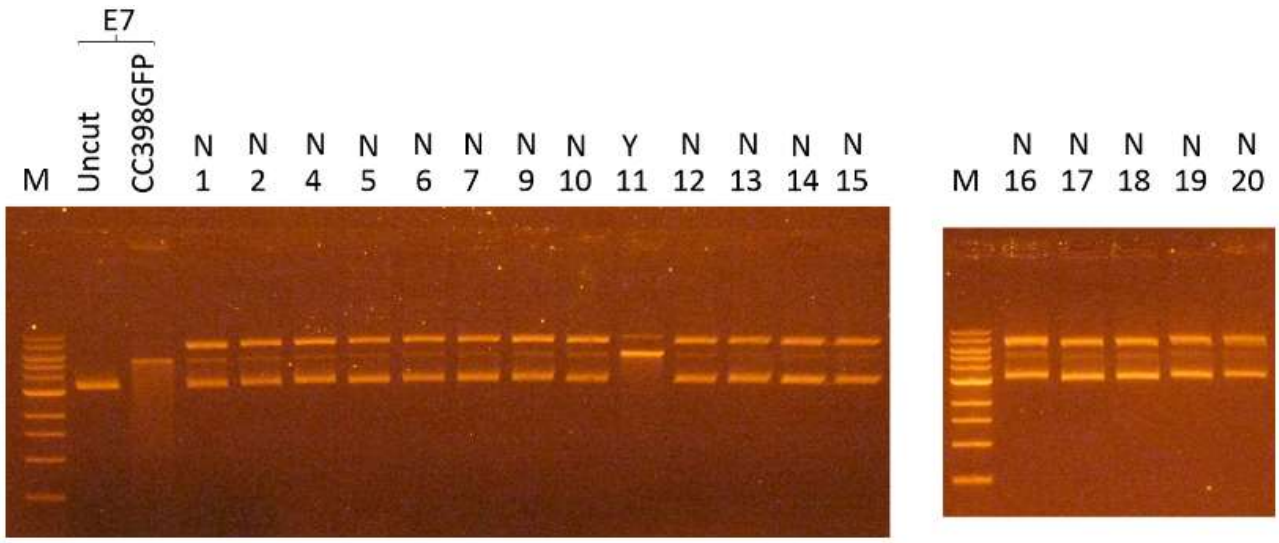

S.SauJP
CC51 GGA-6-CCT
This MTase was used in the SMRT analysis of *E. coli* ER2796.
There are minor variations in the sequences of the S subunits in CC51.
Recombinant S.SauJP CC51-1

MSNTQKKNVPELRFPGFEGEWEEKKLGDLIKVNSGKDYKHLEKGDIPVYGTGGYMTSVSEPLSEID
AVGIGRKGTINKPYLLEAPFWTVDTLFYCTPKKETDILFILSLFRKINWKVYDESTGVPSLSKQTI
NKINRFVPSNKEQQKIGEFFIKLDRQIELEEQKLELFQQQKKGYMQKIFSQELRFKDESGNDYPDW
EEKELGEVADRVIRKNKNFESKKPLTISGQLGLIDQTEYFSKSVSSKNLENYTLIKNGEFAYNKSY
SNGYPLGAIKRLTRYDSGVLSSLYICFSIKSEMSKDFMEAYFDSTHWYREVSGIAVEGARNHGLLN
ISVNDFFTILIKYPSLEEQRKIGDFFIKLDRQIELEEQKLELLQQRKKALLKSMLIPGGSHHHHHH

Wild Type S.SauJP

MSNTQTKNVPELRFPGFEGEWEEKKLEDIIKVNSGKDYKHLDKGDIPVYGTGGYMTSVSEPLSEID
AVGIGRKGTINKPYLLEAPFWTVDTLFYCTPKKETDILFILSLFRKINWKVYDESTGVPSLSKQTI
NKINRFVPTNKEQQKIGKFFSKLDRQIELEEQKLELFQQQKKGYMQKIFSQELRFKDESGNDYPDW
EEKELGEVADRVIRKNKNFESKKPLTISGQLGLIDQTEYFSKSVSSKNLENYTLIKNGEFAYNKSY
SNGYPLGAIKRLTRYDSGVLSSLYICFSIKSEMSKDFMEAYFDSTHWYREVSGIAVEGARNHGLLN
ISVNDFFTILIKYPSLEEQRKIGDFFIKLDRQIELEEQKLELLQQRKKALLKSMLI

Reports for Job Dryden_J_P_MODs PACIFIC BIOSCIENCES

SMRT Cells: 1 Movies: 1

Motif Summary

Motifs	Modified Position	Type	% Motifs Detected	# Of Motifs Detected	# Of Motifs In Genome	Mean Modification QV	Mean Motif Coverage	Partner Motif
GGANNNNNNCCT	3	m6A	98.1%	1340	1366	62.31	39.31	AGGNNNNNNTCC
AGGNNNNNNTCC	1	m6A	97.58%	1333	1366	61.04	39.18	GGANNNNNNCCT
BNAGGANNNNNACCH	3	m6A	46.26%	99	214	47.92	39.46	

S.SauJP
CC51 **GGA-6-CCT**

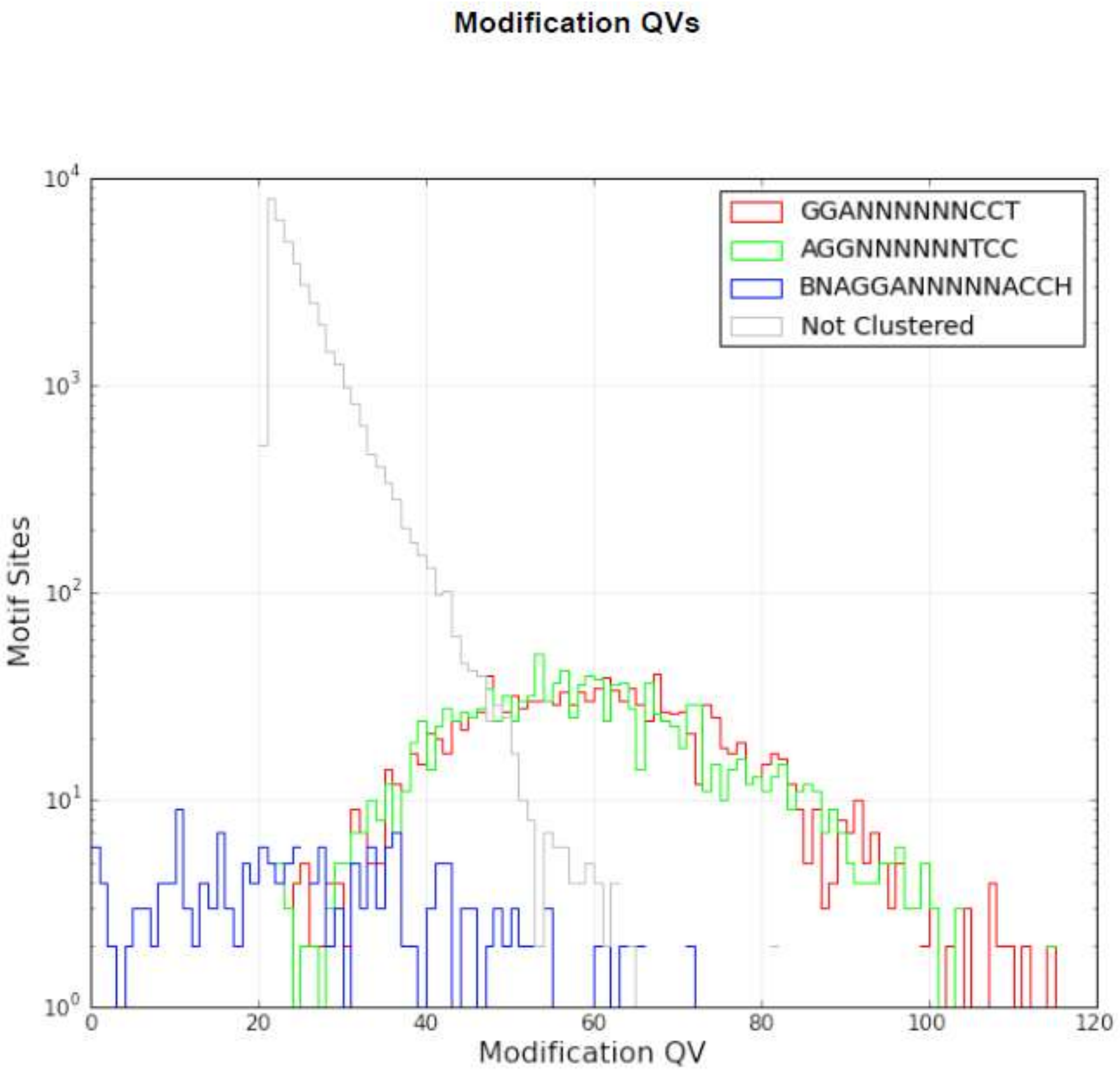

S.SauCL-EGFP

CC45-1 GWAG-6-TAAA

Two separate clones of pSauCL-EGFP encode residue 167 as Lysine (K) instead of arginine (R), but this does not affect the specificity as identical sequences are recognised in Trd C from CC30-1.

S.SauCL-EGFP "Expected" sequence

MSNTQKKNVPELRFPGFEGEWEEKKVGELLEFKNGLNKGKEYFGSGSSIVNFKDVFNNRSINTNNL
TGKVNVNSKELKNYSVEKGDVFFTRTSEVIGEIGYPSVILNDPENTVFSGFVLRGRPKSGIDLINN
NFKRYVFFTNSFRKEMITKSSMTTRALTSGTAIN**R**MKVIYPVSAKEQKKIGDFFSKLDRQIELEEQ
KLELLQQQKKGYMQKIFSQELRFKDENGNDYPNWRTIELKNILENIVDNRGKTPDNAPSEKYPLLE
VNALGYYRPAYIKVSKFVSENTYNNWFREHLKENDILFSTVGNTGIVSLMDNYKAVIAQNIVGLRV
NNNNLPSFIYYMLSYKGNQKKIKRIQMGAVQPSVKVSQFKFIKYLVPIKDEQEKVAKLLIEIDKLV
NKQLIKIELLQQRKKALLKSMFIGSMVSKGEELFTGVVPILVELDGDVNGHKFSVSGEGEGDATYG
KLTLKFICTTGKLPVPWPTLVTTLTYGVQCFSRYPDHMKQHDFFKSAMPEGYVQERTIFFKDDGNY
KTRAEVKFEGDTLVNRIELKGIDFKEDGNILGHKLEYNYNSHNVYIMADKQKNGIKVNFKIRHNIE
DGSVQLADHYQQNTPIGDGPVLLPDNHYLSTQSALSKDPNEKRDHMVLLEFVTAAGITLGMDELYK
HHHHHH

S.SauCL-EGFP "Actual" sequence

MSNTQKKNVPELRFPGFEGEWEEKKVGELLEFKNGLNKGKEYFGSGSSIVNFKDVFNNRSINTNNL
TGKVNVNSKELKNYSVEKGDVFFTRTSEVIGEIGYPSVILNDPENTVFSGFVLRGRPKSGIDLINN
NFKRYVFFTNSFRKEMITKSSMTTRALTSGTAIN**K**MKVIYPVSAKEQKKIGDFFSKLDRQIELEEQ
KLELLQQQKKGYMQKIFSQELRFKDENGNDYPNWRTIELKNILENIVDNRGKTPDNAPSEKYPLLE
VNALGYYRPAYIKVSKFVSENTYNNWFREHLKENDILFSTVGNTGIVSLMDNYKAVIAQNIVGLRV
NNNNLPSFIYYMLSYKGNQKKIKRIQMGAVQPSVKVSQFKFIKYLVPIKDEQEKVAKLLIEIDKLV
NKQLIKIELLQQRKKALLKSMFIGSMVSKGEELFTGVVPILVELDGDVNGHKFSVSGEGEGDATYG
KLTLKFICTTGKLPVPWPTLVTTLTYGVQCFSRYPDHMKQHDFFKSAMPEGYVQERTIFFKDDGNY
KTRAEVKFEGDTLVNRIELKGIDFKEDGNILGHKLEYNYNSHNVYIMADKQKNGIKVNFKIRHNIE
DGSVQLADHYQQNTPIGDGPVLLPDNHYLSTQSALSKDPNEKRDHMVLLEFVTAAGITLGMDELYK
HHHHHH

1 2 3 4 5 6 7 8 9 10 11 12 13 14 15

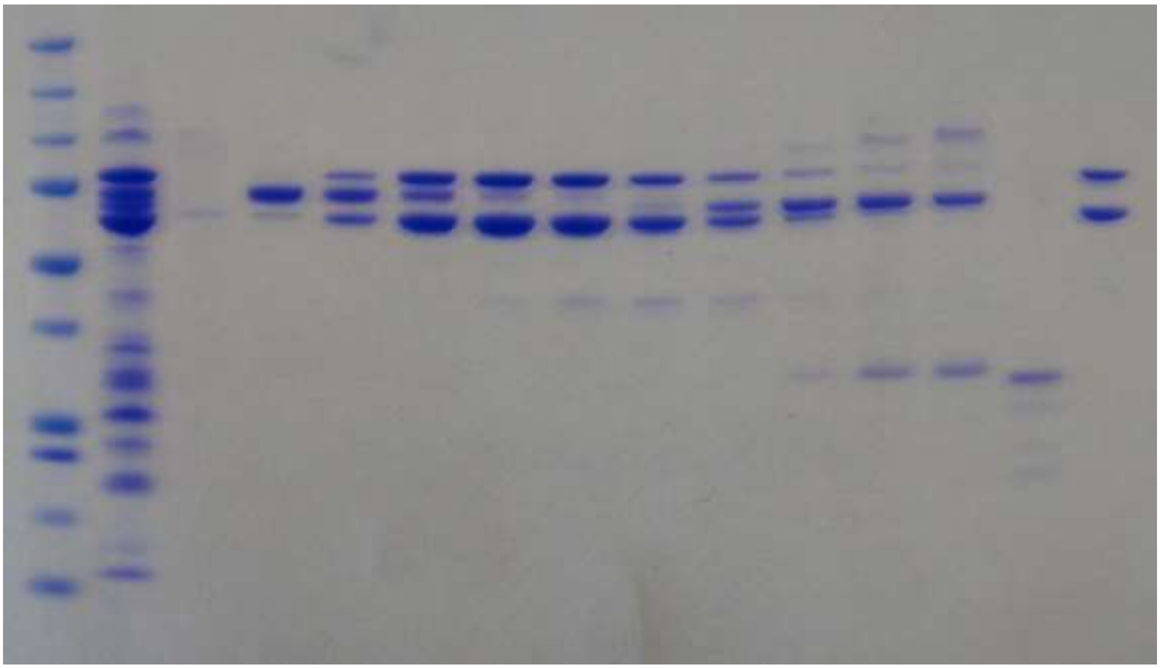

1- marker 2- Nickel column eluate 3-14 Fractions from gel filtration column
15- CC5-1 purified protein marker

S.SauCL-EGFP

CC45-1   GWAG-6-TAAA

DNA cleavage assay.

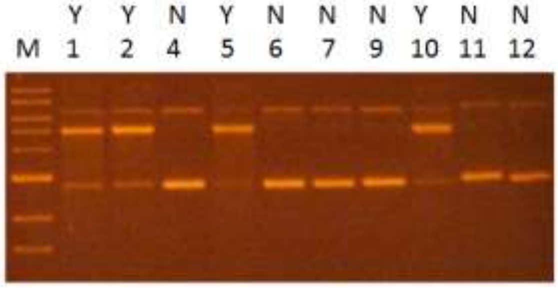

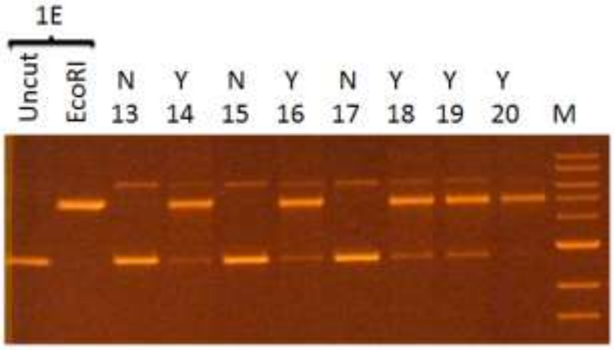

S.SauOE

CC15

Recombinant S.SauOE CC15-1 CAAC-5-RTGA

MSNTQKKNVPELRFPGFEGEWEEKKLGEVGTFTSGGTPLKSKSEYWNGDIPWITTGDIHNIKRENI
TNFITEKGLNESSAKLITNEAILIAMYGQGKTRGMSAILNFEATTNQACAIYQTNQNINFVFQYFQ
KLYEFLRSLSNEGSQKNLSLSLLKEITLNYPNEQEQKKIGDFFSKLDRQIELEEQKLELLQQQKKG
YMQKIFSQELRFKDENGKDYPEWEETTIKEIAQINTGKKDTKDAITNGSYDFYVRSPIVYKINTFS
YEGEAILTVGDGVGVGKVFHYVNGKFDYHQRVYKISDFKNYYGLLLFYYFSQNFLKETKKYSAKTS
VDSVRKDMIANMKVPRPIYIEQKKIGQFIKRVDNKTKIQKQVIELLKQRKKSLLQKMFIPGGSHHH
HHH

Wild Type S.SauOE

MSNKQKKNVPELRFPGFEGEWEEKKLGEVGTFTSGGTPLKSKSEYWNGDIPWITTGDIHNIKRENI
TNFITEKGLNESSAKLITNEAILIAMYGQGKTRGMSAILNFEATTNQACAIYQTNQNINFVFQYFQ
KLYEFLRSLSNEGSQKNLSLSLLKEITLNYPNEQEQKKIGDFFSKLDRQIELEEQKLELLQQQKKG
YMQKIFSQELRFKDENGNDYPEWEETTIKEIAQINXGKKDTKDAITNGSYDFYVRSPIVYKINTFS
YEGEAILTVGDGVGVGKVFHYVNGKFDYHQRVYKISDFKNYYGLLLFYYFSQNFLKETKKYSAKTS
VDSVRKDMIANMKVPRPIYIEQKKIGQFIKRVDNKTKIQKQVIELLKQRKKALLQKMFI

1 2 3 4 5 6 7 8 9

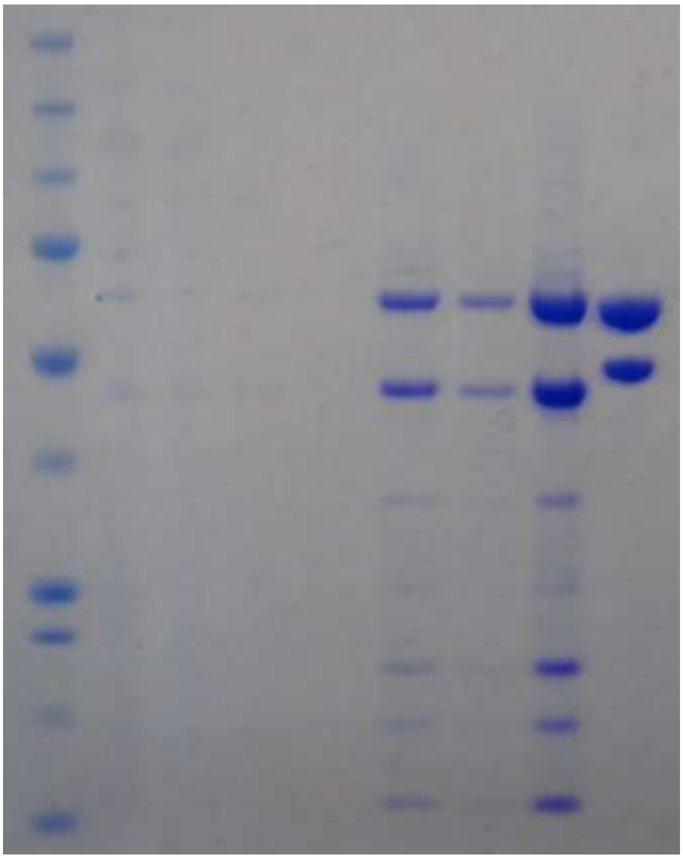

1- marker 2- soluble cell extract 3- Nickel column flow through
4- Nickel column wash 1 5- Nickel column wash 2 6- Nickel column eluate
7- eluate after PD10 desalting 8- Final concentrated protein
9- CC398-1 purified protein marker

Although purified, this MTase was only used in SMRT sequencing.

S.SauOE
CC15
Recombinant S.SauOE   CC15-1   CAAC-5-RTGA

Motifs

Motif	Modified Position	Modification Type	% Motifs Detected	# Of Motifs Detected	# Of Motifs In Genome	Mean Modification QV	Mean Motif Coverage	Partner Motif
TCAYNNNNNGTTG	3	m6A	96.67	261	270	63.4	38.3	CAACNNNNNRTGA
CAACNNNNNRTGA	3	m6A	92.22	249	270	57.7	36.1	TCAYNNNNNGTTG
TCAGNDNNNGTTG	3	m6A	23.86	47	197	48.5	38.3	CAACNNNHNCTGA
CAACNNNHNCTGA	3	m6A	17.77	35	197	49.9	38.6	TCAGNDNNNGTTG
CCAHKNNNNGTTG	3	m6A	16.93	32	189	50.6	40.4	
CAACNNNNHRTGG	3	m6A	16.28	35	215	45.3	38.2	
Not Clustered	0		0.02	2201	9115988	34.9	47.5	

Modification QV Histogram By Motif

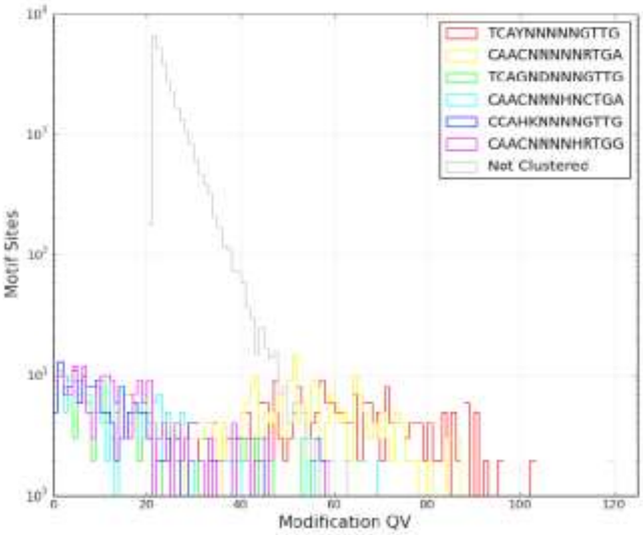

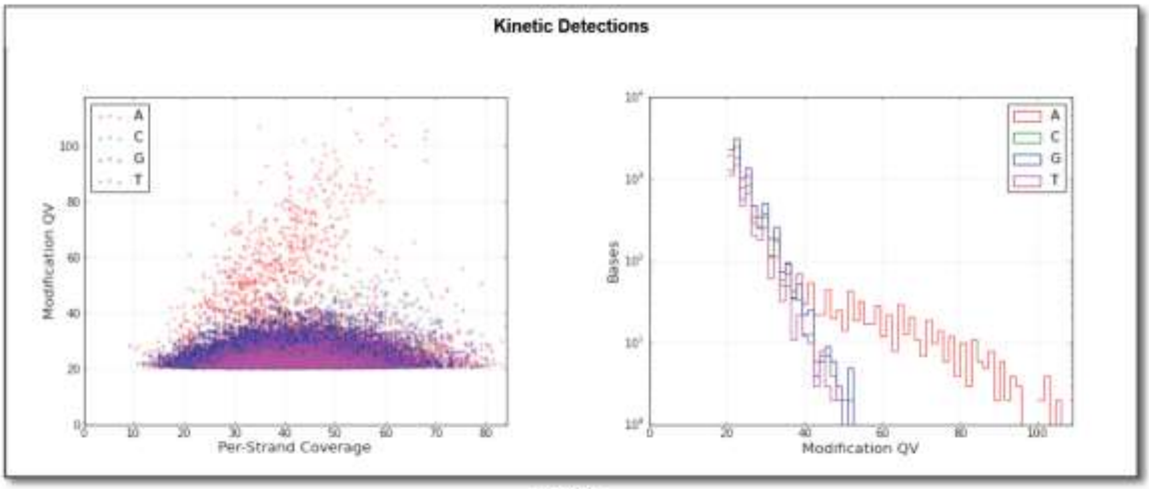

Motifs

Motif	Modified Position	Modification Type	% Motifs Detected	# Of Motifs Detected	# Of Motifs In Genome	Mean Modification QV	Mean Motif Coverage	Partner Motif
TCAYNNNNNGTTG	3	m6A	96.67	261	270	63.4	38.3	CAACNNNNNRTGA
CAACNNNNNRTGA	3	m6A	92.22	249	270	57.7	36.1	TCAYNNNNNGTTG
TCAGNDNNNGTTG	3	m6A	23.86	47	197	48.5	38.3	CAACNNNHNCTGA
CAACNNNHNCTGA	3	m6A	17.77	35	197	49.9	38.6	TCAGNDNNNGTTG
CCAHKNNNNGTTG	3	m6A	16.93	32	189	50.6	40.4	
CAACNNNNHRTGG	3	m6A	16.28	35	215	45.3	38.2	
Not Clustered	0		0.02	2201	9115988	34.9	47.5	

S.SauJQ
CC59

This enzyme was purified and analysed using the ATPase assay as both TRD specificities were known and the DNA cleavage assay showed cutting of all plasmids.

Recombinant S.SauJQ   CC59-1   GGA-6-RTGT

MSNTQKKNVPELRFPGFEGEWEEKKLGDLIKVNSGKDYKHLEKGDIPVYGTGGYMTSVSEPLSEID
AVGIGRKGTINKPYLLEAPFWTVDTLFYCTPKKETDILFILSLFRKINWKVYDESTGVPSLSKQTI
NKINRFVPSNKEQQKIGEFFIKLDRQIELEEQKLELLQQQKKGYMQKIFSQELRFKDENGEDYSEW
EERRFADIFKFHNKLRKPIKENLRVKGSYPYYGATGIIDYVDDFIFDGNYLLIGEDGANIITRSAP
LVYLVNGKFWVNNHAHILSPLNGNIQYLYQVAELVNYEKYNTGTAQPKLNIQNLKIINVVISTNLE
EQQKIGSFLSKLDRQIDLEEQKLELLQQRKKALLKSMFVPGGSHHHHHH

Wild type S.SauJQ

MSNTQKKNVPELRFPEFEGEWEERKLGDLIKVNSGKDYKHLDKGDIPVYGTGGYMTSVSEPLSEID
AVGIGRKGTINKPYLLEAPFWTVDTLFYCTPEKEADILFILSLFRKINWKLYDESTGVPSLSKQTI
NKINRLVPTNKEQQKIGEFFSKLDRQIELEEQKLELLQQQKKGYMQKIFSQELRFKDENGEDYSEW
EERRFADIFKFHNKLRKPIKENLRVKGSYPYYGATGIIDYVDDFIFDGNYLLIGEDGANIITRSAP
LVYLVNGKFWVNNHAHILSPLNGNIQYLYQVAELVNYEKYNTGTAQPKLNIQNLKIINVVISTNLE
EQQKIGSFLSKLDRQIDLEEQKLELLQQRKKALLKSMFV*

1 2 3 4 5 6 7 8 9

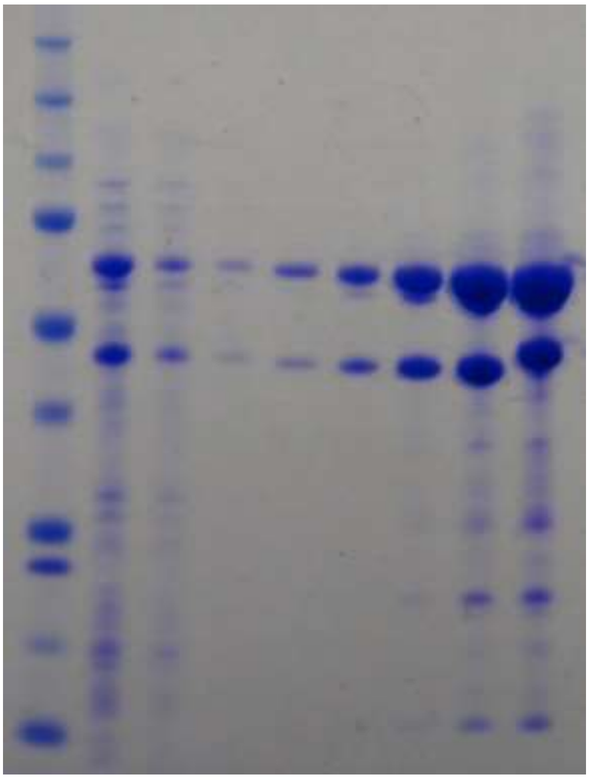

1- marker 2- soluble cell extract 3- Nickel column flow through
4- Nickel column wash 1 5- Nickel column wash 2 6- Nickel column eluate
7- eluate after conc. and PD10 desalting
8- final concentrated protein 9- XE purified protein marker

S.SauJQ
CC59
Recombinant S.SauJQ   CC59-1   GGA-6-RTGT
ATPase assay shows that N=6.

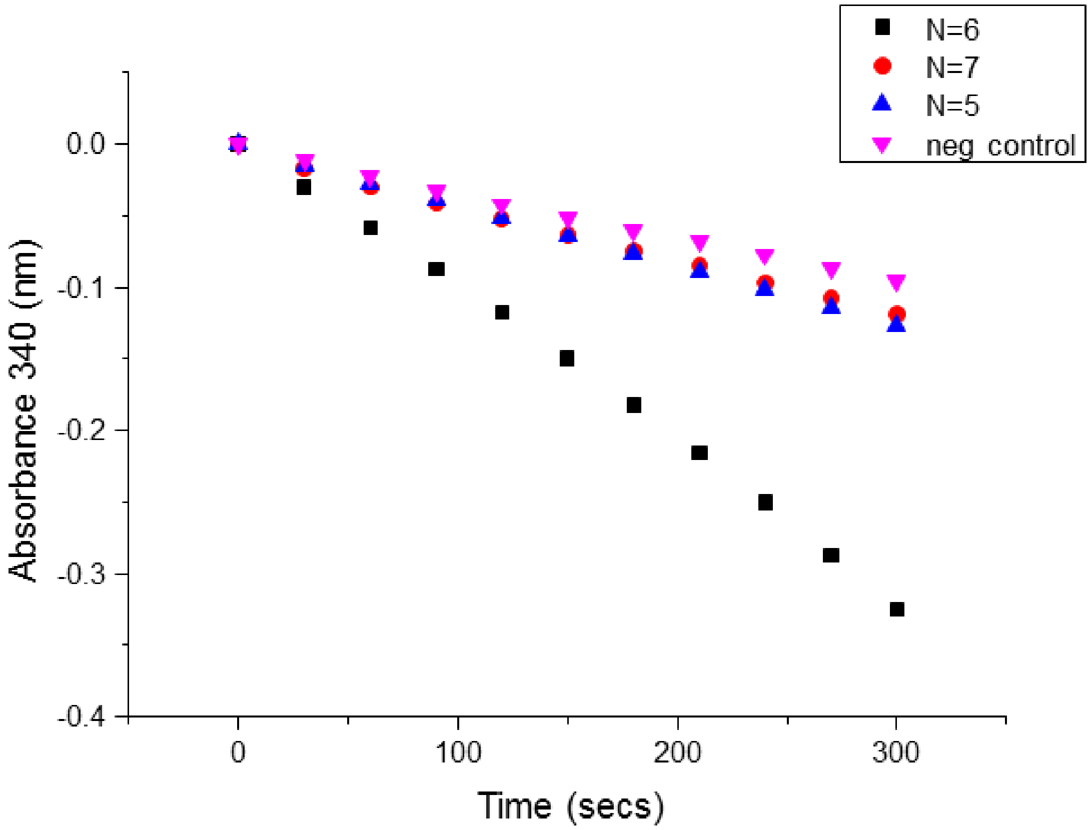

Oligonucleotide name	DNA sequence (5' to 3')
JQ5for	AGATGATGTCATCAATGCGGATTACAGTGTGCCCTATACGATATAA
JQ5rev	TTATATCGTATAGGGCACACTGTAATCCGCATTGATGACATCATCT
JQ6for	AGATGATGTCATCAATGCGGATTGACAGTGTGCCCTATACGATATAA
JQ6rev	TTATATCGTATAGGGCACACTGTCAATCCGCATTGATGACATCATCT
JQ7for	AGATGATGTCATCAATGCGGATTAGACAGTGTGCCCTATACGATATAA
JQ7rev	TTATATCGTATAGGGCACACTGTCTAATCCGCATTGATGACATCATCT

S.SauRQ

CC72

This enzyme was purified and analysed using the ATPase assay as both TRD specificities were known and the DNA cleavage assay showed cutting of all plasmids.

Recombinant S.SauRQ CC72-1 GARA-6-RTGT

MSNTQKKNVPELRFPGFEGEWEEKKLGEVAKIYDGTHQTPKYTNEGIKFLSVENIKTLNSSKYISE
EAFEKEFKIRPEFGDILMTRIGDIGTPNIVSSNEKFAYYVSLALLKTKNLNSYFLKNLILSSSIQN
ELWRKTLHVAFPKKINKNEIGKIKINYPKKQEQQKIGQFFSKLDRQIELEEQKLELLQQQKKGYMQ
KIFSQELRFKDENGEDYSEWEERRFADIFKFHNKLRKPIKENLRVKGSYPYYGATGIIDYVDDFIF
DGNYLLIGEDGANIITRSAPLVYLVNGKFWVNNHAHILSPLNGNIQYLYQVAELVNYEKYNTGTAQ
PKLNIQNLKIINVVISTNLEEQQKIGSFLSKLDRQIDLEEQKLELLQQRKKALLKSMFVPGGSHHH
HHH

Wild type S.SauRQ

MSNTQKKNVPELRFPGFEGEWEEKKLGEVAKIYDGTHQTPKYTNEGIKFLSVENIKTLNSSKYISE
EAFEKEFKIRPEFGDILMTRIGDIGTPNIVSSNEKFAYYVSLALLKTKNLNSYFLKNLILSSSIQN
ELWRKTLHVAFPKKINKNEIGKIKINYPKKQEQQKIGQFFSKLDRQIELEEQKLELLQQQKKGYMQ
KIFSQELRFKDENGNDYPEWEERRFADIFKFHNKLRKPIKENLRVKGSYPYYGATGIIDYVDDFIF
DGNYLLIGEDGANIITRSAPLVYLVNGKFWVNNHAHILSPLNGNIQYLYQVAELVNYEKYNTGTAQ
PKLNIQNLKIISVVISTNLEEQQKIGSFLSKLDRQIDLEEQKLELLQQRKKALLKSMFV*

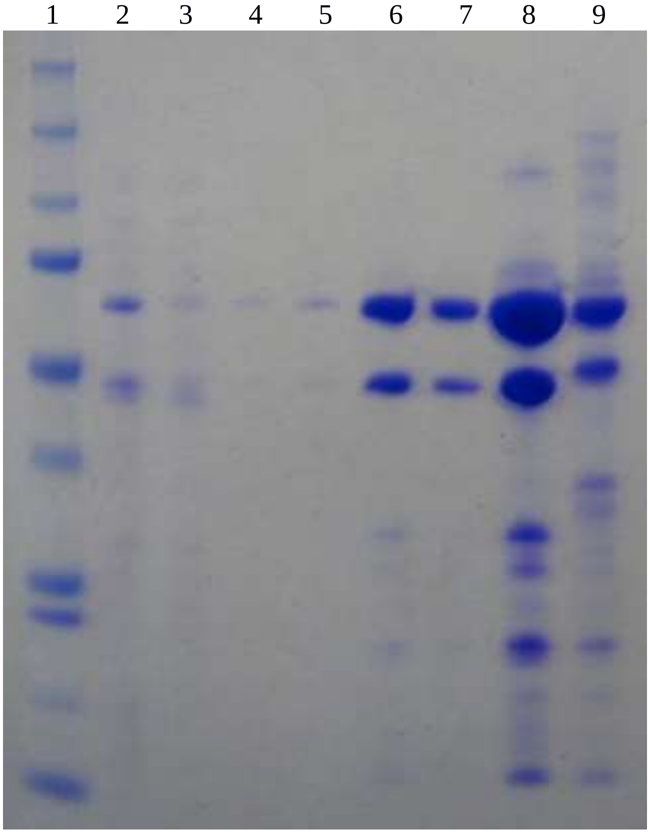

1- marker 2- soluble cell extract 3- Nickel column flow through
4- Nickel column wash 1 5- Nickel column wash 2 6- Nickel column eluate
7- eluate after PD10 desalting 8- Final protein after concentration
9- NP purified protein as marker

S.SauRQ
CC72
Recombinant S.SauRQ GARA-6-RTGT

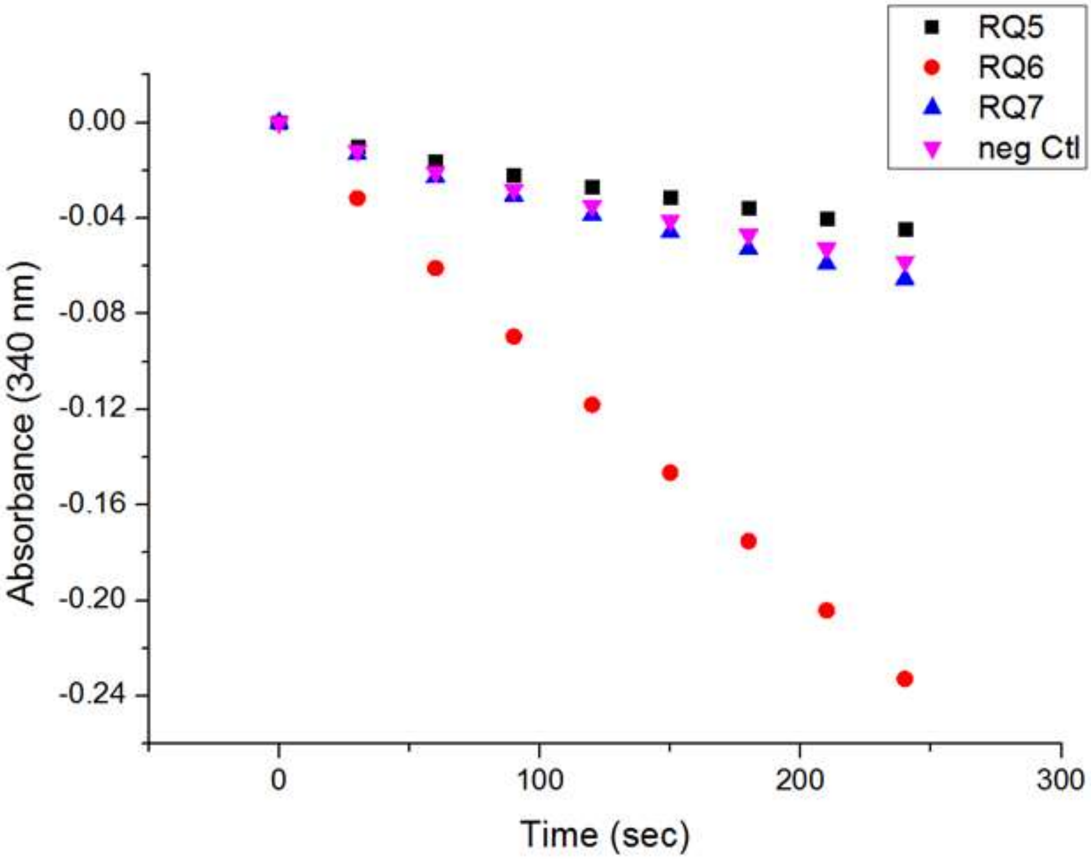

N=6 shows activity.

Oligonucleotide name	DNA sequence (5' to 3')
RQ5for	AGATGATGGAATCAATGCGAGATTCCAGTGTGCCCTATACGATATAA
RQ5rev	TTATATCGTATAGGGCACACTGGAATCTCGCATTGATTCCATCATCT
RQ6for	AGATGATGGAATCAATGCGAGATGTCCAGTGTGCCCTATACGATATAA
RQ6rev	TTATATCGTATAGGGCACACTGGACATCTCGCATTGATTCCATCATCT
RQ7for	AGATGATGGAATCAATGCGAGATGTACCAGTGTGCCCTATACGATATAA
RQ7rev	TTATATCGTATAGGGCACACTGGTACATCTCGCATTGATTCCATCATCT

S.SauJS

This second enzyme from CC72 was purified and analysed using the ATPase assay. There are minor variations between S subunit sequences in CC72-2.

CC72

Recombinant S.SauJS CC72-2 GGA-7-TGC

MSNTQKKNVPELRFPGFEGEWEEKKLGDLIKVNSGKDYKHLEKGDIPVYGTGGYMTSVSEPLSEID
AVGIGRKGTINKPYLLEAPFWTVDTLFYCTPKKETDILFILSLFRKINWKVYDESTGVPSLSKQTI
NKINRFVPSNKEQQKIGEFFIKLDRQIELEEQKLELLQQQKKGYMQKIFSQELRFKDENGNDYPDW
TNERLGEVTTVTMGQSPKSVNYTDNSNDTVLIQGNADIENGLINPRIYTREVTKLIQKDEIILTVR
APVGKLAMAQINACIGRGVCSIKGDKFLYYFLEWFATQNKWIRFSQGSTFESISGNDIRNIHIKIP
VEDERTKIIKLLNSLDVLNSKTDLKIQNLKQRKQSLLQKIFVPGGSHHHHHH

Wild Type S.SauJS

MSNTQKKNVPELRFPEFEGEWEEKQLGNIIKVNSGKDYKHLDKGDIPVYGTGGYMTSVSEPLSEID
AVGIGRKGTINKPYLLEAPFWTVDTLFYCTPKKETDILFILSLFRKINWKVYDESTGVPSLSKQTI
NKINRFVPTNKEQQKIGKFFSKLDRQIELEEQKLELLQQQKKGYMQKIFSQELRFKDENGNDYPDW
TNERLGEVTTVTMGQSPKSVNYTDNSNDTVLIQGNADIENGLINPRIYTREVTKLIQKDEIILTVR
APVGKLAMAQINACIGRGVCSIKGDKFLYYFLEWFATQNKWIRFSQGSTFESISGNDIRNIHIKIP
VEDERTKIIKLLNSLDVLNSKTDLKIQNLKQRKQSLLQKIFV

1 2 3 4 5 6 7 8 9

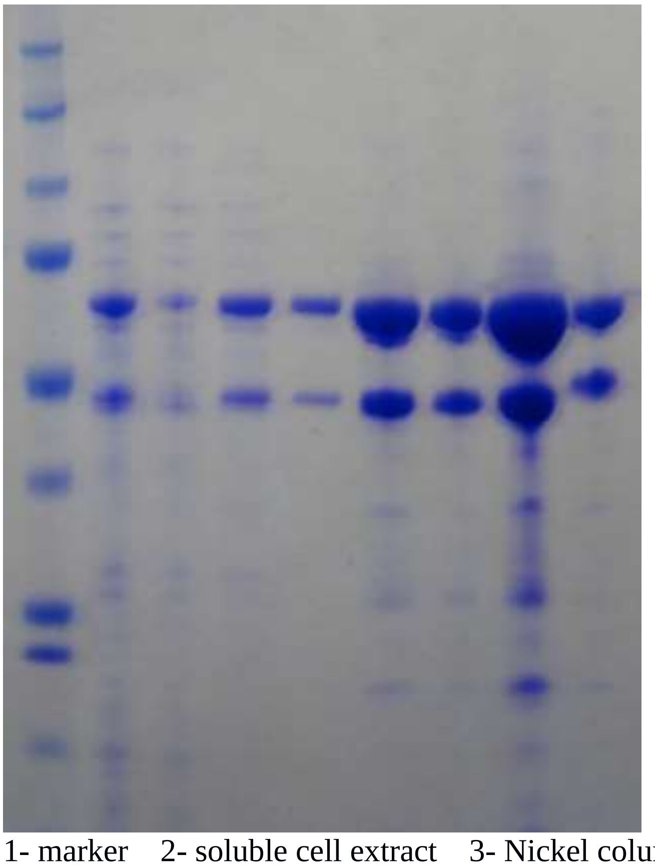

1- marker 2- soluble cell extract 3- Nickel column flow through
4- Nickel column wash 1 5- Nickel column wash 2 6- Nickel column eluate
7- eluate after PD10 desalting 8- final protein after concentration
9- NP purified protein as marker

S.SauJS
CC72
Recombinant S.SauJS CC72-2 GGA-7-TGC
N=7 shows activity.

**Oligonucleotide name**	**DNA sequence (5' to 3')**
JS6for	AGATGATGGCATCAATGCGGATTACATTGCGCCCTATACGATATAA
JS6rev	TTATATCGTATAGGGCGCAATGTAATCCGCATTGATGCCATCATCT
JS7for	AGATGATGGCATCAATGCGGATTGACATTGCGCCCTATACGATATAA
JS7rev	TTATATCGTATAGGGCGCAATGTCAATCCGCATTGATGCCATCATCT

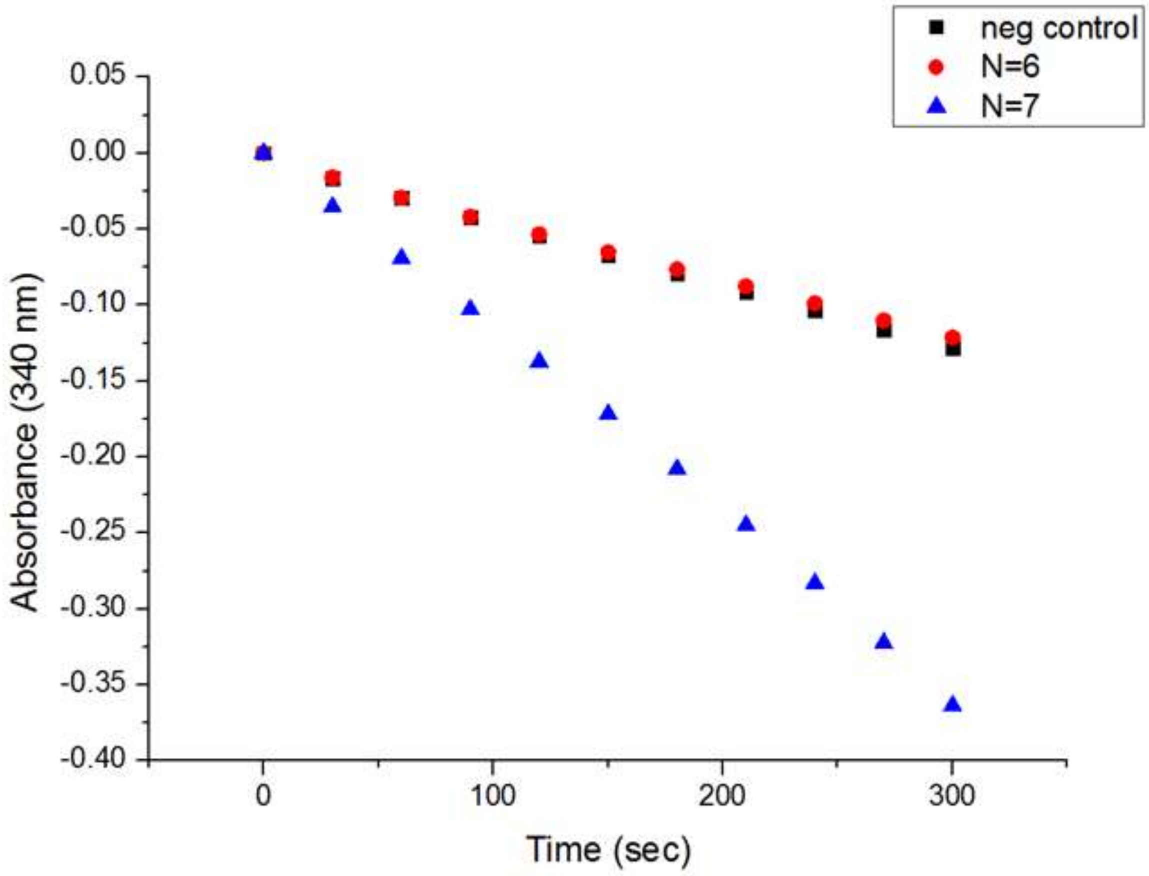

S.SauTU
CC75
Recombinant S.SauTU   CC75-1   CAAG-5-RTC

MSNTQKKNVPELRFPGFEGEWEEKELGEIFQIISGSTPLKSNKEFYENGNINWVKTTDLNNSKVTH
SKEKITEYAMKSLKLKLVPKNSVLIAMYGGFNQIGRTGLLKIDATINQAISALLMNHETNPEFIQA
FLNYQVKGWKRYAASSRKDPNITKKDIEQFKVPYVSINEQQKIGEFFSKIDHQIELEEQKLELLQQ
QKKGYMQKIFSQELRFKDENGEDYPDWEVTTIQNITKYTSSKKSSNQYADKDNSKGYPVYDAVQEI
GKDSNYDIEESYISILKDGAGVGRLNLRPGKSSVIGTMGYIQSNNVDIEFLYYRMKVVDFKKYIIG
STIPHLYFKDYSKETLYIPSSIQEQAKIGMFISNLDKLIENKNLKLNCLKQLKQGLLQSMFIPGGS
HHHHHH

Wild type S.SauTU

MSNTQTKNVPELRFPGFEGEWEEKELGEIFQIISGSTPLKSNKEFYENGNINWVKTTDLNNSKVTH
SKEKITEYAMKSLKLKLVPKNSVLIAMYGGFNQIGRTGLLKIDATINQAISALLMNHETNPEFIQA
FLNYQVKGWKRYAASSRKDPNITKKDIEQFKVPYVSINEQQKIGEFFSKIDHQIELEEQKLELLQQ
QKKGYMQKIFSQELRFKDENGEDYPDWEVTTIQNITKYTSSKKSSNQYADKDNSKGYPVYDAVQEI
GKDSNYDIEESYISILKDGAGVGRLNLRPGKSSVIGTMGYIQSNNVDIEFLYYRMKVVDFKKYIIG
STIPHLYFKDYSKETLYIPSSIQEQAKIGMFISNLDKLIENKNLKLNCLKQLKQGLLQSMFI

1 2 3 4 5 6 7 8

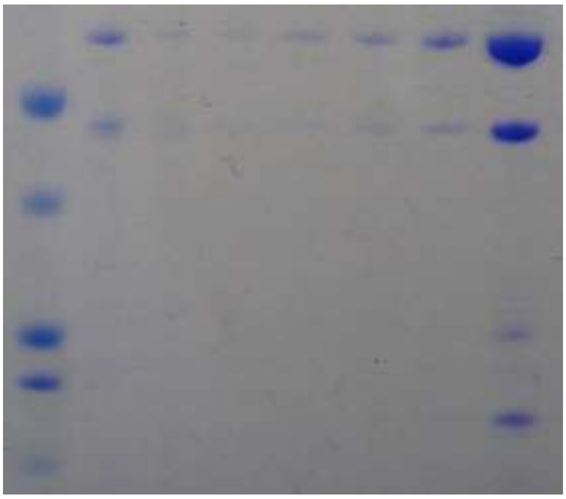

1- marker 2- soluble cell extract 3- Nickel column flow through 4- Nickel column wash 1 5- Nickel column wash 2 6- Nickel column eluate 7- eluate after conc. and PD10 desalting 8- final protein after concentration

DNA cleavage assay.

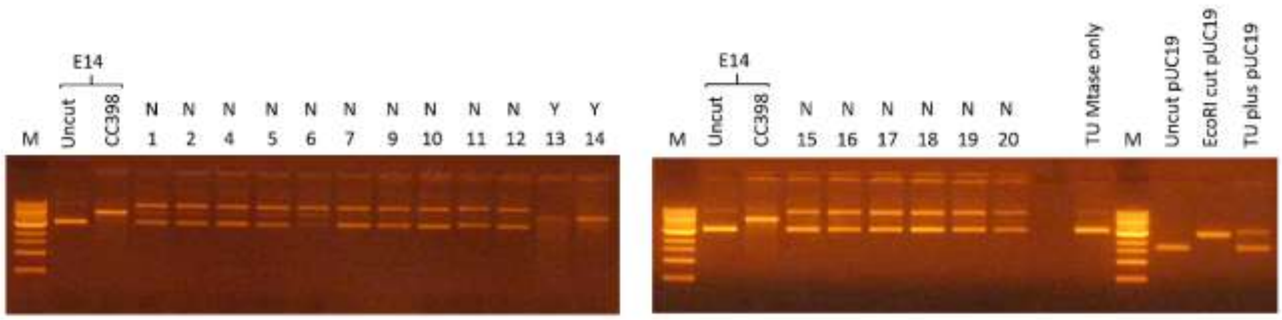

S.SauVW
CC75
Recombinant S.SauVW CC75-2 CNGA-7-TTYG

MSNTQKKNVPELRFPGFEGEWEEKELRELRNPKDKYSYTGGPFGSDLKKSDYTTDGIQIIQLQNIG
DGYFYNSNKVFTSNEKAEVLKSCNVFPGDIVIAKMADPIARAAIVPDNNIGKYLMASDGIRLSVDT
VHFNTKFVLECINRKSFRKKVEDNSSGSTRMRIGLSTLGSLTLKTTTLKEQQKIGQFFSKLDRQIE
LEEQKLELLQQQKKGYMQKIFSQELRFKDENGNDYPDWEEKQLGELSQIVRGASPRPIKDPKWFNK
ESDIGWLRISDVTNQNGKIYHLEQKLSIEGQEKTRVLVTTHLLLSIAASIGKPVMNFVKTGVHDGF
LIFLKPKFNLFFMYYWLEYFKDKWSKYGQPGSQVNLNSEIVKSQTLNMPSNHEQEKVGQFFNRNEK
LIELQQEKIMYIKRCKQVLLQKMFIPGGSHHHHHH

Wild Type S.SauVW

MSNTGKMNVPELRFPGFEGEWEEKELRELRNPKDKYSYTGGPFGSDLKKSDYTTDGIQIIQLQNIG
DGYFYNSNKVFTSNEKAEVLKSCNVFPGDIVIAKMADPIARAAIVPDNNIGKYLMASDGIRLSVDT
VHFNTKFVLECINRKSFRKKVEDNSSGSTRMRIGLSTLGSLTLKTTTLKEQQKIGQFFSKLDRQIV
LEEQKLELLQQQKKGYMQKIFSQELRFKDENGNDYPDWEEKQLGELSQIVRGASPRPIKDPKWFNK
ESDIGWLRISDVTNQNGKIYHLEQKLSIEGQEKTRVLVTTHLLLSIAASIGKPVMNFVKTGVHDGF
LIFLKPKFNLFFMYYWLEYFKDKWSKYGQPGSQVNLNSEIVKSQTLNMPSNHEQEKVGQFFNRNEK
LIELQQEKIMYIKRCKQVLLQKMFI*

Reports for Job Dryden_V_W_MODs PACIFIC BIOSCIENCES

SMRT Cells: 1 Movies: 1

Motif Summary

Motifs	Modified Position	Type	% Motifs Detected	# Of Motifs Detected	# Of Motifs In Genome	Mean Modification QV	Mean Motif Coverage	Partner Motif
CNGANNNNNNNTTYG	4	m6A	99.93%	1442	1443	97.87	66.11	CRAANNNNNNNTCNG
CRAANNNNNNNTCNG	4	m6A	99.86%	1441	1443	89.76	63.95	CNGANNNNNNNTTYG
DNNNNNNNGCCACNCAD	9	unknown	19.1%	72	377	38.56	67.49	

S.SauVW
CC75
Recombinant S.SauVW **CC75-2** **CNGA-7-TTYG**

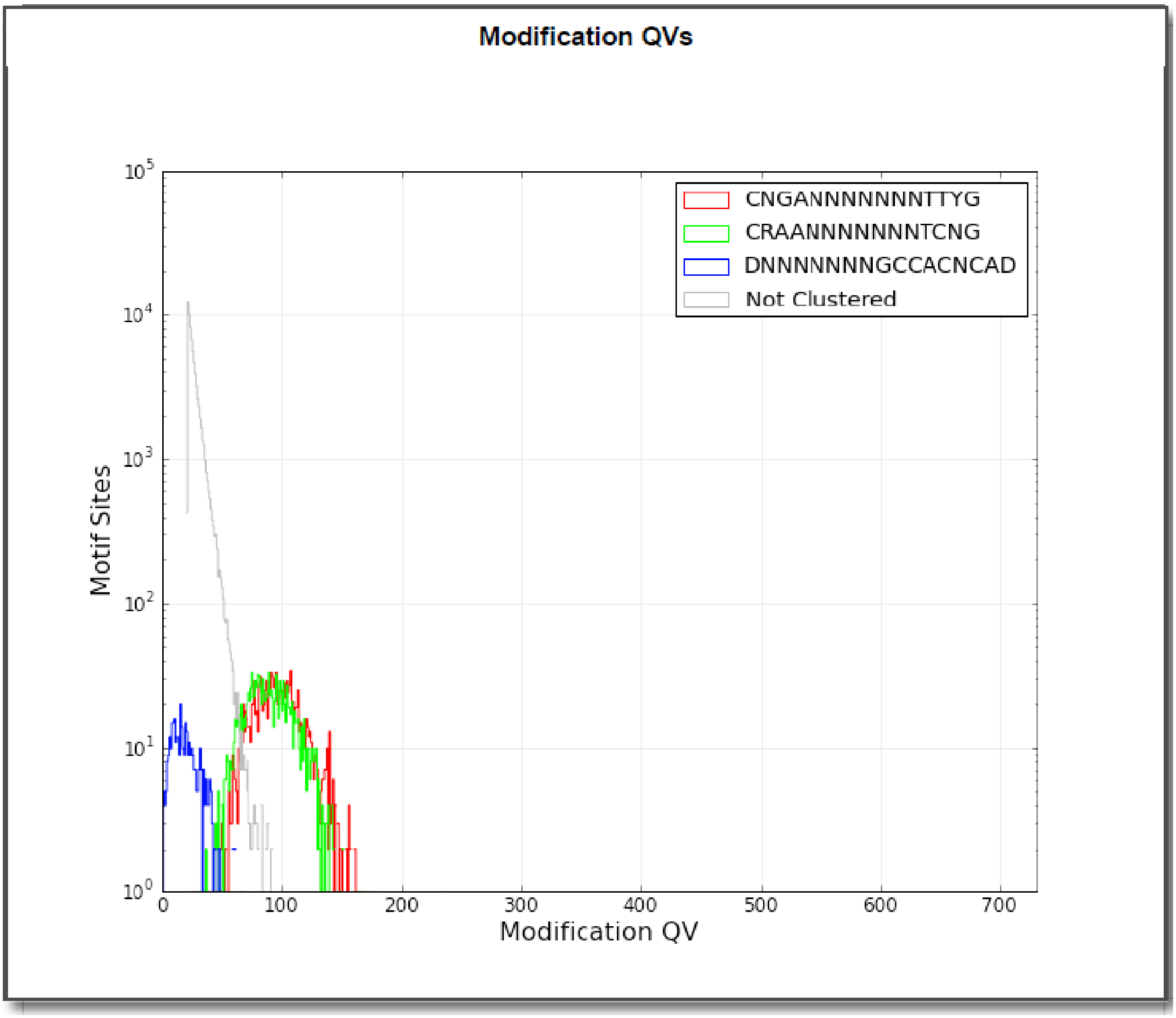

S.SauZW
CC80
Recombinant S.SauZW   CC80-2   GAC-6-TTYG

MSNTQKKNVPELRFPGFEGEYSLDIFGNLATNKSEKFNPQNENASIDIELDCIEQNTGRLIKIYNS
KEFSSQKNKFNPQNVLYGKLRPYLNKYYFTKKSGVCSSEIWVLKSTKEDKLLNLFLYYFIQTKRYS
DVASKSAGSKMPRADWGLIENIRVYFPELCEQQKIGQFFSKLDRQIELEEQKLELLQQQKKGYMQK
IFSQELRFKDENGNDYPDWEEKQLGELSQIVRGASPRPIKDPKWFNKESDIGWLRISDVTNQNGKI
YHLEQKLSIEGQEKTRVLVTTHLLLSIAASIGKPVMNFVKTGVHDGFLIFLKPKFNLFFMYYWLEY
FKDKWSKYGQPGSQVNLNSEIVKSQTLNMPSNHEQEKVGQFFNRNEKLIELQQEKIMYIKRCKQVL
LQKMFIPGGSHHHHHH

Wild Type S.SauZW

MSNTQTKNVPELRFPGFEGEYSLDIFGNLATNKSEKFNPQNENASIDIELDCIEQNTGRLIKIYNS
KEFSSQKNKFNPQNVLYGKLRPYLNKYYFTKKSGVCSSEIWVLKSTKEDKLLNLFLYYFIQTKRYS
DVASKSAGSKMPRADWGLIENIRVYFPELCEQQKIGQFFSKLDRQIELEEQKLELLQQQKKGYMQK
IFSQELRFKDENGNDYPDWEEKQLGELSQIVRGASPRPIKDPKWFNKESDIGWLRISDVTNQNGKI
YHLEQKLSIEGQEKTRVLVTTHLLLSIAASIGKPVMNFVKTGVHDGFLIFLNPKFNLFFMYYWLEY
FKDKWSKYGQPGSQVNLNTEIVKSQTLNMPSNHEQEKVGQFFNRNEKLIELQQEKIMYLKRRKQVL
LQKMFI*

1 2 3 4 5 6 7 8 9

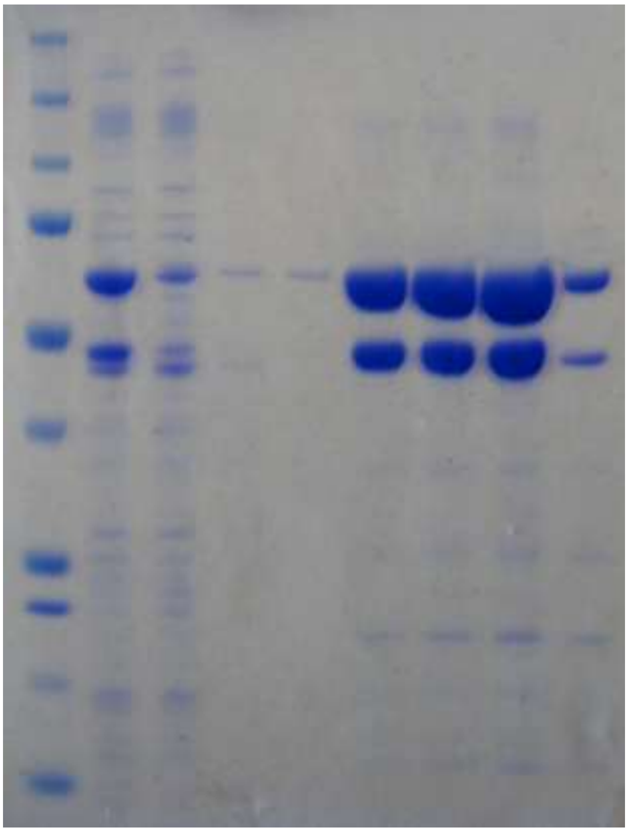

1- marker   2- soluble cell extract   3- Nickel column flow through
4- Nickel column wash 1   5- Nickel column wash 2   6- Nickel column eluate
7- eluate after conc. and PD10 desalting
8- final protein after concentration   9- CC75-1 purified protein marker

Although purified, this enzyme cut all plasmids in the DNA cleavage assay so the ATPase assay was used as we knew the specificities of the TRDs.

S.SauZW
CC80
Recombinant S.SauZW   CC80-2   GAC-6-TTYG
N=6 shows activity.

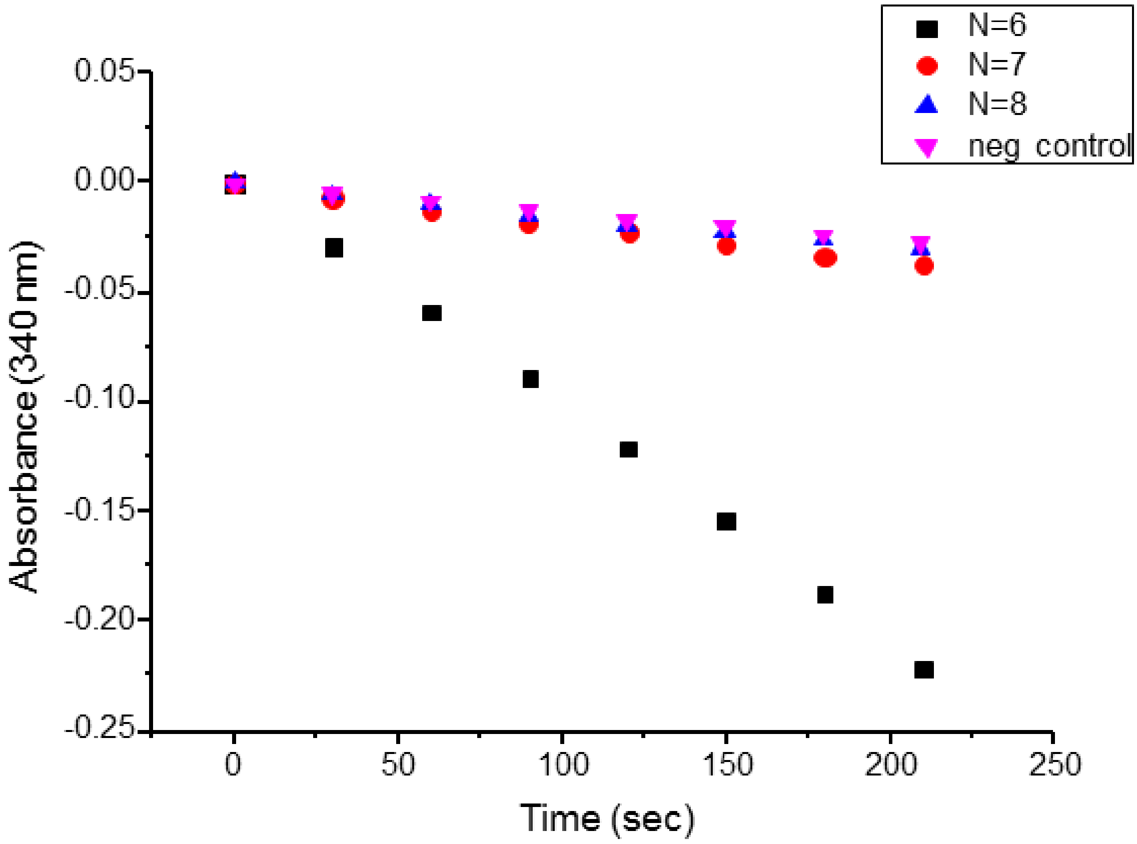

**Oligonucleotide name**	**DNA sequence (5' to 3')**
ZW6for	AGATGATGGAATCAATGCGACTTCCATTTCGGCCCTATACGATATAA
ZW6rev	TTATATCGTATAGGGCCGAAATGGAAGTCGCATTGATTCCATCATCT
ZW7for	AGATGATGGAATCAATGCGACTTCTCATTTCGGCCCTATACGATATAA
ZW7rev	TTATATCGTATAGGGCCGAAATGAGAAGTCGCATTGATTCCATCATCT
ZW8for	AGATGATGGAATCAATGCGACTTCTACATTTCGGCCCTATACGATATAA
ZW8rev	TTATATCGTATAGGGCCGAAATGTAGAAGTCGCATTGATTCCATCATCT

S.SauXf*
ST80
Recombinant S.SauXf*   **CC80-3**   **TCTA-6-RTTC**

MSNTQKKNVPELRFPGFEGEWEEKQFADFTKINQGLQIAINERKTEYSPELYFYITNEFLRPNSQT
KYFIENPPQSVIANKEDILMTRTGNTGKVVTNVFGAFHNNFFKIKFDKNLYDRLFLVEVLNSSKIQ
NKILSLAGSSTIPDLNHSDFYSISSSYPLLREQQKIGKFFSKLDRQIELEEQKLELLQQQKKGYMQ
KIFSQELRFKDENGEDYPDWKEKKLGDITEQSMYGIGASATRFDSKNIYIRITDIDEKSRKLNYQN
LTTPDELNNKYKLKRNDILFARTGASTGKSYIHKEEKDIYNYYFAGFLIKFKINEQNSPLFIYQFT
LTSKFNKWVKVMSVRSGQPGINSEEYAKLPLVLPNKLEQQKIAKFLDRFDRQIELEKQKIEILQQQ
KKGLLQSMFIPGGSHHHHHH

Wild Type S.SauXf*

MSNTQKKNVPELRFPEFEGEWEEKQFADFTKINQGLQIAINERKTEYSPELYFYITNEFLRPNSQT
KYFIENPPQSVIANKEDILMTRTGNTGKVVTNVFGAFHNNFFKIKFDKNLYDRLFLVEVLNSSKIQ
NKILSLAGSSTIPDLNHSDFYSISSSYPLLREQQKIGKFFSKLDRQIELEEQKLELLQQQKKGYMQ
KIFSQELRFKDENGEDYPDWKEKKLGDITEQSMYGIGASATRFDSKNIYIRITDIDEKSRKLNYQN
LTTPDELNNKYKLKRNDILFARTGASTGKSYIHKEEKDIYNYYFAGFLIKFKINEQNSPLFIYQFT
LTSKFNKWVKVMSVRSGQPGINSEEYAKLPLVLPNKLEQQKIAKFLDRFDRQIELEKQKIEILQQQ
KKGLLQSMFI

Reports for Job Dryden_X_zeta_MODs

SMRT Cells: 1 Movies: 1

Motif Summary

Motifs	Modified Position	Type	% Motifs Detected	# Of Motifs Detected	# Of Motifs In Genome	Mean Modification QV	Mean Motif Coverage	Partner Motif
TCTANNNNNNRTTC	4	m6A	100.0%	92	92	96.27	61.85	GAAYNNNNNNTAGA
GAAYNNNNNNTAGA	3	m6A	100.0%	92	92	90.82	60.21	TCTANNNNNNRTTC

Modification QVs

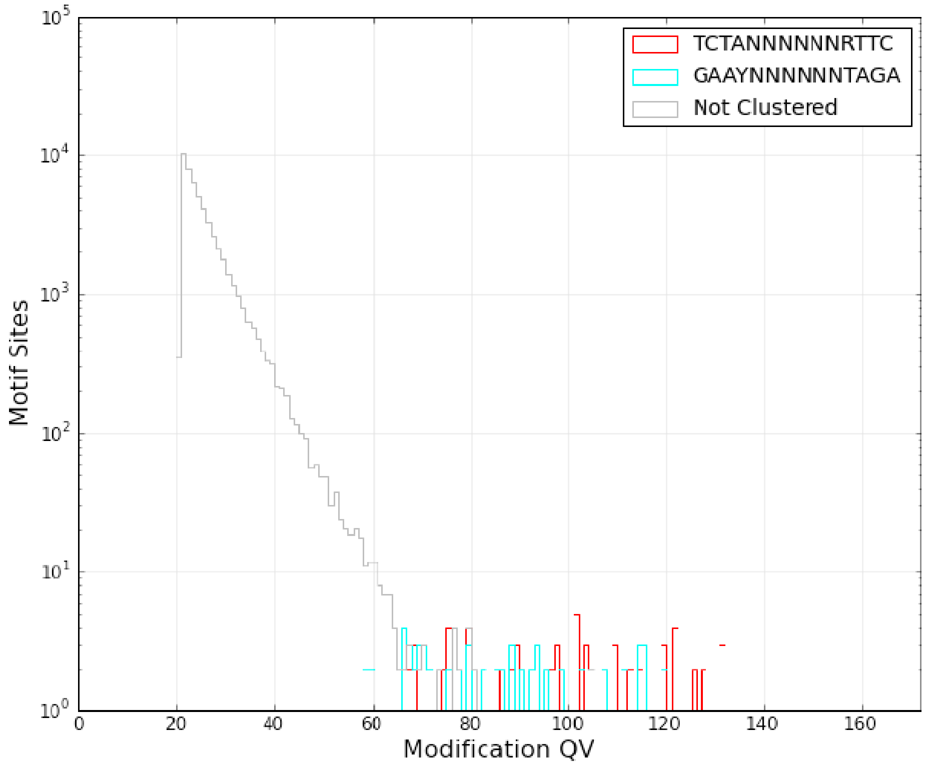

S.Saue*D

CC873

Recombinant S.Saue*D   CC873-1   GAG-6-GAT

MSNTQKKNVPELRFPGFEGEWEEKSISSFLKESKIKGSNGSHAKKLTVKLWGKGVVPKKETFKGSD
NTQYYKRKAGQLMYGKLDFLNCAFGIVPDSLNNYESTIDSPSFDFINGDSKFLLERIKLKSFYKKF
GDIANGSRKAKRINQDTFLSLPVFAPKYDEQLRIGEFFSKLDRQIELEEQKLELLQQQKKGYMQKI
FSQELRFKDENSEDYPHWENSKIEKYLKERNERSDKGQMLSVTINSGIIKFSELDRKDNSSKDKSN
YKVVRKNDIAYNSMRMWQGASGRSNYNGIVSPAYTVLYPTQNTSSLFIGYKFKTHRMIHKFKINSQ
GLTSDTWNLKYKQLKNINIDIPVLEEQEKIGDFFKKMDILISKQKIKIEILEKEKQSFLQKMFLPG
GSHHHHHH

Wild Type S.Saue*D

MSNTQKKNVPELRFPGFEGEWEEKSISSFLKESKIKGSNGSHAKKLTVKLWGKGVVPKKETFKGSD
NTQYYKRKAGQLMYGKLDFLNCAFGIVPDSLNNYESTIDSPSFDFINGDSKFLLERIKLKSFYKKF
GDIANGSRKAKRINQDTFLSLPVFAPKYDEQLRIGEFFSKLDRQIELQKQKLELLQQQKKGYMQKI
FSQELRFKDENGEDYPHWENSKIEKYLKERNERSDKGQMLSVTINSGIIKFSELDRKDNSSKNKSN
YKVVRKNDIAYNSMRMWQGASGKSNYNGIVSPAYTVLYPTQNTSSLFIGYKFKTHRMIHKFKINSQ
GLTSDTWNLKYKQLKNINIDIPVLEEQEKIGDFFKKMDILISKQKIKIEILEKEKQSFLQKMFL*

1 2 3 4 5 6 7 8 9

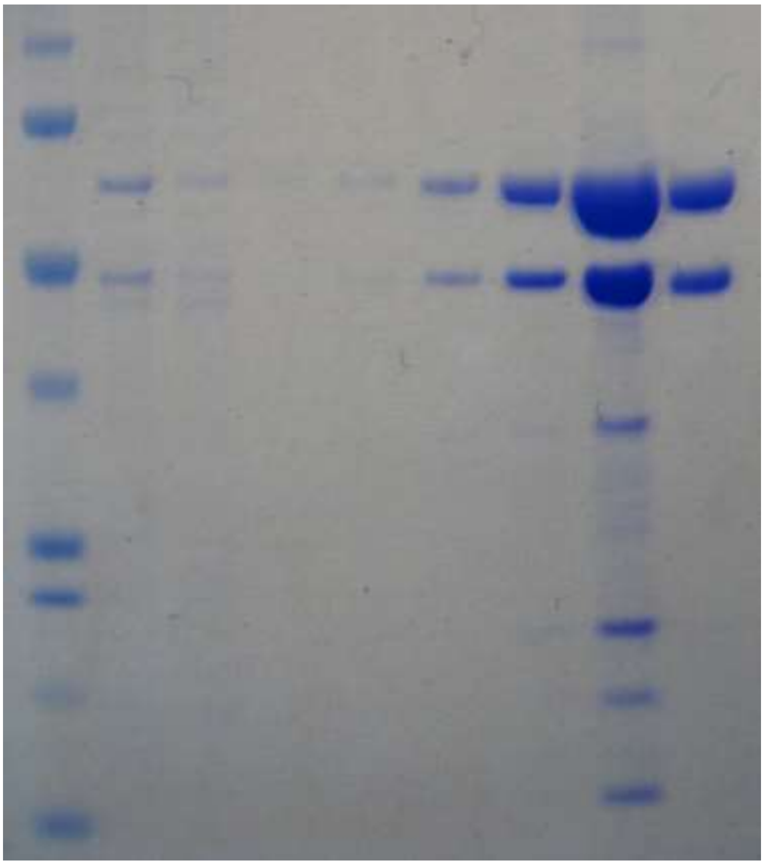

1- marker 2- soluble cell extract 3- Nickel column flow through
4- Nickel column wash 1 5- Nickel column wash 2 6- Nickel column eluate
7- eluate after PD10 desalting and concentration
8- Final concentrated protein 9- CC398-1 purified protein marker

S.Saue*D
CC873-1
Recombinant S.Saue*D **GAG-6-GAT**

e*D clearly digests pUC19 so the ATPase assay was used as we knew the specificities of both TRDs.

Likely site: GAG-N_x-GAT

GAG-4-GAT 2 sites in pUC19

GAG-5-GAT 0 sites in pUC19

GAG-6-GAT 2 sites in pUC19

GAG-7-GAT 0 sites in pUC19

**Oligonucleotide name**	**DNA sequence (5' to 3')**
e*D6for	AGATGATGGAATCAATGCGAGTTCCATGATGCCCTATACGATATAA
e*D6rev	TTATATCGTATAGGGCATCATGGAACTCGCATTGATTCCATCATCT
e*D5for	AGATGATGGAATCAATGCGAGTTCCAGATGCCCTATACGATATAA
e*D5rev	TTATATCGTATAGGGCATCTGGAACTCGCATTGATTCCATCATCT
e*D4for	AGATGATGGAATCAATGCGAGTTCAGATGCCCTATACGATATAA
e*D4rev	TTATATCGTATAGGGCATCTGAACTCGCATTGATTCCATCATCT

N=6 shows activity.

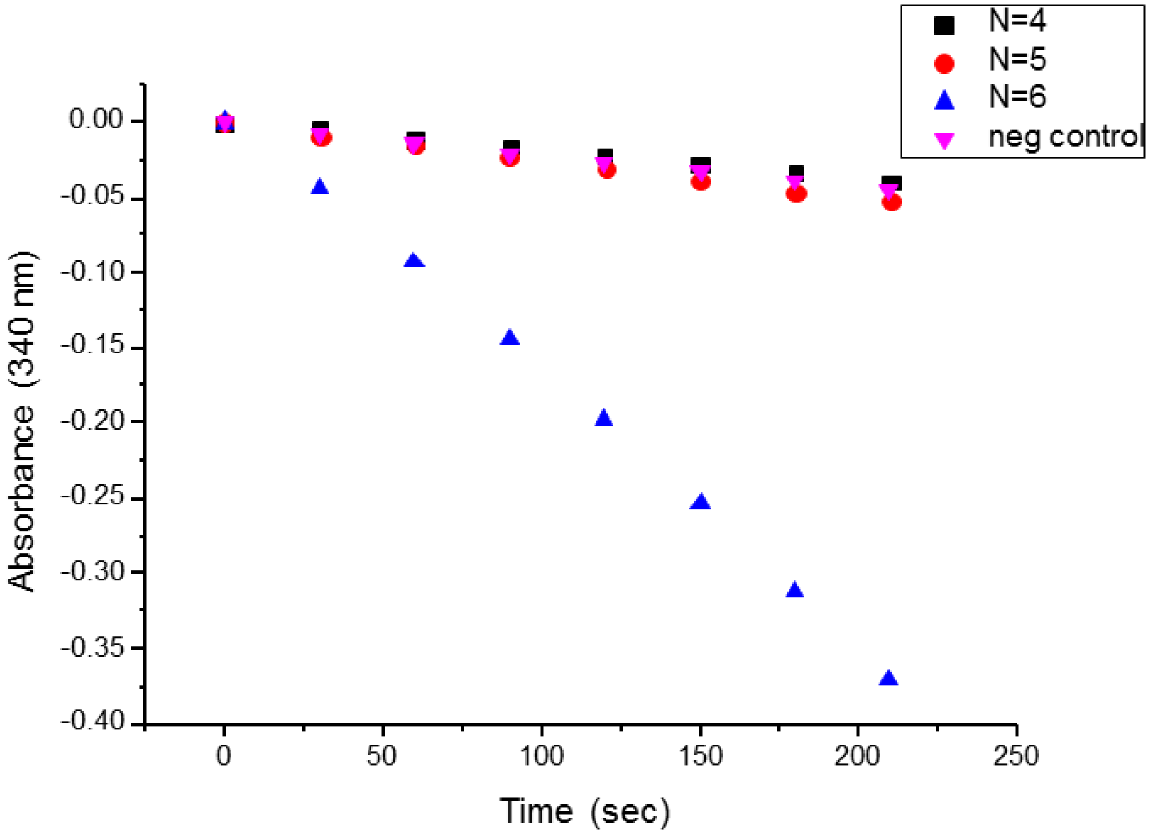

SMRT results for S. aureus strains LGA251 and NCTC13435

LGA251

SMRT® Portal Print

Reports for Job Dryden_LGA_Mods

PACIFIC BIOSCIENCES

SMRT Cells: 2 Movies: 2

Motif Summary

Motifs	Modified Position	Type	% Motifs Detected	# Of Motifs Detected	# Of Motifs In Genome	Mean Modification QV	Mean Motif Coverage	Partner Motif
TCAYNNNNNCTWC	3	m6A	100.0%	391	391	352.92	251.20	GWAGNNNNNRTGA
GWAGNNNNNRTGA	3	m6A	100.0%	391	391	349.86	243.53	TCAYNNNNNCTWC
GTANNNNNCTTC	3	m6A	99.59%	245	246	349.71	251.31	GAAGNNNNNTAC
GAAGNNNNNTAC	3	m6A	99.59%	245	246	349.91	237.85	GTANNNNNCTTC
BTTGGTAVY	2	unknown	26.29%	127	483	38.52	249.23	

NCTC13435

SMRT® Portal Print Close

Reports for Job Dryden_NTCT_Mods

PACIFIC BIOSCIENCES

SMRT Cells: 2 Movies: 2

Motif Summary

Motifs	Modified Position	Type	% Motifs Detected	# Of Motifs Detected	# Of Motifs In Genome	Mean Modification QV	Mean Motif Coverage	Partner Motif
CRAANNNNNNNGTC	4	m6A	100.0%	422	422	275.90	212.58	GACNNNNNNTTYG
GACNNNNNNTTYG	2	m6A	100.0%	422	422	315.18	220.50	CRAANNNNNNGTC
GAAYNNNNNNTAGA	3	m6A	100.0%	260	260	307.05	214.54	TCTANNNNNNRTTC
TCTANNNNNNRTTC	4	m6A	100.0%	260	260	318.07	218.98	GAAYNNNNNNTAGA
GGATG	3	m6A	100.0%	2818	2818	327.54	220.52	CATCC
CATCC	2	m6A	100.0%	2818	2818	324.65	220.07	GGATG
DNNNNNNNASNGGATG	9	m6A	26.07%	67	257	141.42	225.84	

SUPPLEMENTARY INFORMATION FOR TABLES 5 AND 6.

SUPPLEMENTARY INFORMATION FOR TABLES 5 AND 6.

By combining all TRD 1 with all TRD 2 amino acid sequences and searching sequence databases, we found that some of our “artificial hybrids” described in Table 3 were actually present in real strains of *S. aureus*. We present several examples below.

S.SauAU

A plasmid expressing S.SauAU with the M subunit was prepared but not analysed further. The S.SauAU sequence matches that of the S subunit of the Type I RM system in *S. schweitzeri* FSA084.

```
>S.SauAU
MSNTQKKNVPELRFPGFEGEWEEKKLGDLTTKIGSGKTPKGGSENYTNKGIPFLRSQNIRNGKLNL
NDLVYISKDIDDEMKNSRTYYGDVLLNITGASIGRTAINSIVEIHANLNQHVCIIRLKKEYYYNFF
GQYLLSRKGKRKIFLAQSGGSREGLNFKEIANLKIFTPTIFEEQQKIGEFISKLDRQIELEEQKLE
LLQQQKKGYMQKIFSQELRFKDENGEDYPDWEVTTIQNITKYTSSKKSSNQYADKDNSKGYPVYDA
VQEIGKDSNYDIEESYISILKDGAGVGRLNLRPGKSSVIGTMGYIQSNNVDIEFLYYRMKVVDFKK
YIIGSTIPHLYFKDYSKETLYIPSSIQEQAKIGMFISNLDKLIENKNLKLNCLKQLKQGLLQSMFI
PGGSHHHHHH
```

S. schweitzeri FSA084

CLUSTAL O(1.2.1) multiple sequence alignment

```
FSA084       msn-tqkkvpelrfpgfegeweekklgevttkigsgktpkggsenytnkgipflrsqnir
S.SauAU      MSNTQKKNVPELRFPGFEGEWEEKKLGDLTTKIGSGKTPKGGSENYTNKGIPFLRSQNIR
             ***  :*:*******************::*******************************

FSA084       ngklnlndlvyiskdiddemknsrtyygdvllnitgasigrtainsivethanlnqhvci
S.SauAU      NGKLNLNDLVYISKDIDDEMKNSRTYYGDVLLNITGASIGRTAINSIVEIHANLNQHVCI
             ************************************************* **********

FSA084       irlkkeyyynffeqyllsrkgkrkiflaqsggsreglnfkeianlkiftstifeeqqkvg
S.SauAU      IRLKKEYYYNFFGQYLLSRKGKRKIFLAQSGGSREGLNFKEIANLKIFTPTIFEEQQKIG
             ************ ************************************ ********:*

FSA084       kffskldrqieleeqklellqqqkkgymqkifsqelrfkdengneypewkvtsiqdvtky
S.SauAU      EFISKLDRQIELEEQKLELLQQQKKGYMQKIFSQELRFKDENGEDYPDWEVTTIQNITKY
             :*:****************************************::**:*:**:**::***

FSA084       tsskkssnqyadkidskgypvydavreigkdsnydieesyisilkdgagvgrlnlrpeks
S.SauAU      TSSKKSSNQYADKDNSKGYPVYDAVQEIGKDSNYDIEESYISILKDGAGVGRLNLRPGKS
             ************* :**********:******************************* **

FSA084       svigtmgylqannidleflyyrmkivdfkkyiigstiphlyfkdysketiyipssiqeqa
S.SauAU      SVIGTMGYIQSNNVDIEFLYYRMKVVDFKKYIIGSTIPHLYFKDYSKETLYIPSSIQEQA
             ********:*:**:*:********:************************:**********

FSA084       kigkfisnldkmienktrklnclkqlkqgllqgmfi----------
S.SauAU      KIGMFISNLDKLIENKNLKLNCLKQLKQGLLQSMFIPGGSHHHHHH
             *** *******:****. **************.***
```

S.SauJE   GGA-6-RTGA

S.SauJE against subspecies 21262, a member of ST49

HsdS sequences from strain 21262.

```
>EHO91218 This has TRD R + f*
MSNTQKKNVPELRFPGFEGEWEEKKLGEVAKIYDGTHQTPKYTNEGIKFLSVENIKTLNS
SKYISEEAFEKEFKIRPEFGDILMTRIGDIGTPNIVSSNEKFAYYVSLALLKTKNLNSYF
LKNLILSSSIQNELWRKTLHVAFPKKINKNEIGKIKINYPKKQEQQKIGQFFSKLDRQIE
LEEQKLELLQQQKKGYMQKIFSQELRFKDENGEDYPDWKEKKLGDITEQSMYGIGASATR
FDSKNIYIRITDIDEKSRKLNYQNLTTPDELNNKYKLKRNDILFARTGASTGKSYIHKEE
KDIYNYYFAGFLIKFEIDEQNNPLFIYQFTLTSKFNKWVKVMSVRSGQPGINSEEYAKLP
LVLPNKLEQQKIAEFLDRFDQQIELEKQKIEILQQQKKGLLQSMFI

>EHO92010 This has TRD J + E
MSNTQKKNVPELRFPGFEGEWEEKKLEDIIKVNSGKDYKHLDKGDIPVYGTGGYMTSVSE
PLSEIDAVGIGRKGTINKPYLLEAPFWTVDTLFYCTPKKETDILFILSLFRKINWKVYDE
STGVPSLSKQTINKINRFVPTNKEQQKIGKFFSKLDRQIELEEQKIELLQQQKKGYIQKI
FSQELRFKDENGDDYPEWEETTIQEIAQINTGKKDTKDAITNGSYDFYVRSPIVYKINTF
SYEGEAILTVGDGVGVGKVFHYVNGKFDYHQRVYKISDFKNYYGLLLFYYFSQNFLKETK
KYSAKTSVDSVRKDMVANMKVPRPIYIEQEKIGQFIKKVDNKIKIQKQVIELLKQRKKAL
LQKMFI
```

CLUSTAL O(1.2.1) multiple sequence alignment

```
EHO91218      msntqkknvpelrfpgfegeweekklgevakiydgthqtpkytnegikflsveniktlns
S.SauJE       MSNTQKKNVPELRFPGFEGEWEEKKLGDLIKVNSGKDYKHL---------EKGDIPVYGT
EHO92010      msntqkknvpelrfpgfegeweekklediikvnsgkdykhl---------dkgdipvygt
              ************************** :: *: .*.. .           .  :* .  :

EHO91218      skyiseeafekefkirpefgdilmtrigdigtpnivssnekfayyvslallk--tknlns
S.SauJE       GGYMTSVS-----EPLSEIDAVGIGRKGTINKPYLLEAP---FWTVDTLFYCTPKKETDI
EHO92010      ggymtsvs-----eplseidavgigrkgtinkpylleap---fwtvdtlfyctpkketdi
              . *::. :     :   *:  : : * * * .* ::.:     : *.  :    .*: :

EHO91218      yflknlilsssiqnelwrktlhvafpkkinkneigkikinypkkqeqqkigqffskldrq
S.SauJE       LF-----ILSLFRKINWKVYDESTGVPSLSKQTINKINRFVPSNKEQQKIGEFFIKLDRQ
EHO92010      lf-----ilslfrkinwkvydestgvpslskqtinkinrfvptnkeqqkigkffskldrq
               *     : * :::  *:   . :   .:.*: * **:   *.::******:** *****

EHO91218      ieleeqklellqqqkkgymqkifsqelrfkdengedypdwkekklgditeqsmygigas-
S.SauJE       IELEEQKLELLQQQKKGYMQKIFSQELRFKDENGKDYPEWEETTIKEIAQINTGKKDTKD
EHO92010      ieleeqkiellqqqkkgyiqkifsqelrfkdengddypeweettiqeiaqintgkkdtkd
              *******:**********:***************.***:*:*..: :*:: .     :.

EHO91218      atrfdskniyiritdideksrklnyqnlttpdelnnkyklkrndilfartgastgksyih
S.SauJE       AITNGSYDFYVRSPIV-YK---------------INTFSYEGEAILTVGDGVGVGKVFHY
EHO92010      aitngsydfyvrspiv-yk---------------intfsyegeailtvgdgvgvgkvfhy
              *    * ::*:*   :  *                *.:. : : ** .  *...** : :

EHO91218      keekdiynyyfagflikfeideqnnplfiyqftltskfnkwvkvmsvrsgqpginseeya
S.SauJE       VNGK--FDYHQRVYKIS-DFKNYYGLLLFYYF--SQNFLKETKKYSAKTSVDSVRKDMIA
EHO92010      vngk--fdyhqrvykis-dfknyyglllfyyf--sqnflketkkysaktsvdsvrkdmva
               : *  ::*:   : *. ::.:    *::* *  :.:* * .*  *.::.  .:..:  *

EHO91218      klplvlpnkleqqkiaefldrfdqqielekqkieilqqqkkgllqsmfi----------
S.SauJE       NMKVPRPIYIEQKKIGQFIKRVDNKTKIQKQVIELLKQRKKSLLQKMFIPGGSHHHHHH
EHO92010      nmkvprpiyieqekigqfikkvdnkikiqkqviellkqrkkallqkmfi----------
              :: :  *  :**:**.:*:.:.*:: :::** **:*:*:**.***.***
```

The above alignment shows that SauJE is identical to the EHO92010 sequence from this strain.

S.SauJE   GGA-6-RTGA
Sub species 21262, a member of ST49

CLUSTAL O(1.2.1) multiple sequence alignment
TRD R and TRD f* against EHO91218, the second HsdS in this strain.

```
CC80-3          ------------------------------------------------------------
EHO91218        msntqkknvpelrfpgfegeweekklgevakiydgthqtpkytnegikflsveniktlns
CC72-1          MSNTQKKNVPELRFPGFEGEWEEKKLGEVAKIYDGTHQTPKYTNEGIKFLSVENIKTLNS

CC80-3          ------------------------------------------------------------
EHO91218        skyiseeafekefkirpefgdilmtrigdigtpnivssnekfayyvslallktknlnsyf
CC72-1          SKYISEEAFEKEFKIRPEFGDILMTRIGDIGTPNIVSSNEKFAYYVSLALLKTKNLNSYF

CC80-3          ------------------------------------------------------------
EHO91218        lknlilsssiqnelwrktlhvafpkkinkneigkikinypkkqeqqkigqffskldrqie
CC72-1          LKNLILSSSIQNELWRKTLHVAFPKKINKNEIGKIKINYPKKQEQQKIGQFFSKLDRQIE

CC80-3          ----------------------QELRFKDENGEDYPDWKEKKLGDITEQSMYGIGASATR
EHO91218        leeqklellqqqkkgymqkifsqelrfkdengedypdwkekklgditeqsmygigasatr
CC72-1          LEEQKLELLQQQKKGYMQKIFS--------------------------------------

CC80-3          FDSKNIYIRITDIDEKSRKLNYQNLTTPDELNNKYKLKRNDILFARTGASTGKSYIHKEE
EHO91218        fdskniyiritdideksrklnyqnlttpdelnnkyklkrndilfartgastgksyihkee
CC72-1          ------------------------------------------------------------

CC80-3          KDIYNYYFAGFLIKFKINEQNSPLFIYQFTLTSKFNKWVKVMSVRSGQPGINSEEYAKLP
EHO91218        kdiynyyfagflikfeideqnnplfiyqftltskfnkwvkvmsvrsgqpginseeyaklp
CC72-1          ------------------------------------------------------------

CC80-3          LVLPNKLEQQKIAKFLDRFDRQIELEKQKIEILQQQKKGLLQSMFI
EHO91218        lvlpnkleqqkiaefldrfdqqielekqkieilqqqkkgllqsmfi
CC72-1          ----------------------------------------------
```

S.SauJE GGA-6-RTGA

S.SauJE against ST49 strain “Tager 104”

The ST49 Tager genome has the same TRD combinations as the ST49 strain 21262.

PATRIC db

>fig|1381115.3.peg.1063|VBIStaAur301678_1063| Type I restriction-modification system, specificity subunit S (EC 3.1.21.3) [Staphylococcus aureus subsp. aureus Tager 104 | 1381115.3] This is TRD R+f*
MSNTQKKNVPELRFPGFEGEWEEKKLGEVAKIYDGTHQTPKYTNEGIKFLSVENIKTLNS
SKYISEEAFEKEFKIRPEFGDILMTRIGDIGTPNIVSSNEKFAYYVSLALLKTKNLNSYF
LKNLILSSSIQNELWRKTLHVAFPKKINKNEIGKIKINYPKKQEQQKIGQFFSKLDRQIE
LEEQKLELLQQQKKGYMQKIFSQELRFKDENGEDYPDWKEKKLGDITEQSMYGIGASATR
FDSKNIYIRITDIDEKSRKLNYQNLTTPDELNNKYKLKRNDILFARTGASTGKSYIHKEE
KDIYNYYFAGFLIKFEIDEQNNPLFIYQFTLTSKFNKWVKVMSVRSGQPGINSEEYAKLP
LVLPNKLEQQKIAEFLDRFDQQIELEKQKIEILQQQKKGLLQSMFI
>fig|1381115.3.peg.2628|VBIStaAur301678_2628| Type I restriction-modification system, specificity subunit S (EC 3.1.21.3) [Staphylococcus aureus subsp. aureus Tager 104 | 1381115.3] This is TRD J+E
MSNTQKKNVPELRFPGFEGEWEEKKLEDIIKVNSGKDYKHLDKGDIPVYGTGGYMTSVSE
PLSEIDAVGIGRKGTINKPYLLEAPFWTVDTLFYCTPKKETDILFILSLFRKINWKVYDE
STGVPSLSKQTINKINRFVPTNKEQQKIGKFFSKLDRQIELEEQKIELLQQQKKGYIQKI
FSQELRFKDENGDDYPEWEETTIQEIAQINTGKKDTKDAITNGSYDFYVRSPIVYKINTF
SYEGEAILTVGDGVGVGKVFHYVNGKFDYHQRVYKISDFKNYYGLLLFYYFSQNFLKETK
KYSAKTSVDSVRKDMVANMKVPRPIYIEQEKIGQFIKKVDNKIKIQKQVIELLKQRKKAL
LQKMFI

S.SauJE against ST49 Tager 104 GGA-6-RTGA

```
CLUSTAL O(1.2.1) multiple sequence alignment


S.SauJE                                         MSNTQKKNVPELRFPGFEGEWEEKKLGDLIKVNSGKDYKHLEKGDIPVYGTGGYMTSVSE
fig|1381115.3.peg.2628|VBIStaAur301678_2628|    MSNTQKKNVPELRFPGFEGEWEEKKLEDIIKVNSGKDYKHLDKGDIPVYGTGGYMTSVSE
                                                ************************** *:************:******************

S.SauJE                                         PLSEIDAVGIGRKGTINKPYLLEAPFWTVDTLFYCTPKKETDILFILSLFRKINWKVYDE
fig|1381115.3.peg.2628|VBIStaAur301678_2628|    PLSEIDAVGIGRKGTINKPYLLEAPFWTVDTLFYCTPKKETDILFILSLFRKINWKVYDE
                                                ************************************************************

S.SauJE                                         STGVPSLSKQTINKINRFVPSNKEQQKIGEFFIKLDRQIELEEQKLELLQQQKKGYMQKI
fig|1381115.3.peg.2628|VBIStaAur301678_2628|    STGVPSLSKQTINKINRFVPTNKEQQKIGKFFSKLDRQIELEEQKIELLQQQKKGYIQKI
                                                ********************:********:** ************:**********:***

S.SauJE                                         FSQELRFKDENGKDYPEWEETTIKEIAQINTGKKDTKDAITNGSYDFYVRSPIVYKINTF
fig|1381115.3.peg.2628|VBIStaAur301678_2628|    FSQELRFKDENGDDYPEWEETTIQEIAQINTGKKDTKDAITNGSYDFYVRSPIVYKINTF
                                                ************.**********:************************************

S.SauJE                                         SYEGEAILTVGDGVGVGKVFHYVNGKFDYHQRVYKISDFKNYYGLLLFYYFSQNFLKETK
fig|1381115.3.peg.2628|VBIStaAur301678_2628|    SYEGEAILTVGDGVGVGKVFHYVNGKFDYHQRVYKISDFKNYYGLLLFYYFSQNFLKETK
                                                ************************************************************

S.SauJE                                         KYSAKTSVDSVRKDMIANMKVPRPIYIEQKKIGQFIKRVDNKTKIQKQVIELLKQRKKSL
fig|1381115.3.peg.2628|VBIStaAur301678_2628|    KYSAKTSVDSVRKDMVANMKVPRPIYIEQEKIGQFIKKVDNKIKIQKQVIELLKQRKKAL
                                                ***************:*************:*******:**** ***************:*

S.SauJE                                         LQKMFIPGGSHHHHHH
fig|1381115.3.peg.2628|VBIStaAur301678_2628|    LQKMFI----------
                                                ******
```

S.SauNQ   ACC-5-RTGT

This TRD pair was found in strains KPL1845 (ST96) and 21343(ST88).

Subspecies 21343 contains SauNQ and a novel TRD (NOVEL 1) paired with TRD K.

>EHQ67679 THIS IS TRD NOVEL 1 + TRD K

MSNTQKKNVPELRFPGFEGEWEEKKLGEVATFAKGKLGAKKDVSQNGVPVILYGELYTKY
GAIVSKIFSKTDIPENKLKMAKKNDVLIPSSGETAIDIATASCIYLNKGVAVGGDINILT
PQKQDGRFISLSINGINKNELSKYAQGKTVVHLYNNDIKNLKIAFPSEFEEQVRIGNFFS
KLDRQIELEEQKLELLQQQKKGYMQKIFSQELRFKDENGNDYPKWEEKKIEDIASQVYGG
GTPNTKIKEFWNGDIPWIQSSDVKVNDLILQQCNKFISKNSIELSSAKLIPANSIAIVTR
VGVGKLCLVEFDYATSQDFLSLSSLKYDKLYSLYSLLYTMKKISANLQGTSIKGITKKEL
LDSIIKIPHNLEEQQKIGDLFYKIDKYISFNKCKIEILKSLKQGLLKKMFI

>EHQ71248 THIS IS TRD N+Q ACC-5-RTGT

MSNTQTKNVPELKFPEFEGEWEEKKLGEFAGKVTKKNVDKKYIETLTNSAELGIISQKDY
FDKEISNIDNIKKYYVVEENDFVYNPRISNYAPFGPVNRNKLGKKGVMSPLYTVFKIQNI
DLNFIEFYFKSSKWYRFMALNGDSGARADRFSIKNRTFMEMPLHIPCMDEQIKIGQFFSK
LDRQIELEEQKLELLQQQKKGYMQKIFSQELRFKDENGNDYPEWEERRFADIFKFHNKLR
KPIKENLRVKGSYPYYGATGIIDYVDDFIFDGNYLLIGEDGANIITRSAPLVYLVNGKFW
VNNHAHILSPLNGNIQYLYQVAELVNYEKYNTGTAQPKLNIQNLKIISVVISTNLEEQQK
IGSFLSKLDRQIDLEEQKLELLQQRKKALLKSMFV

SPECIES KPL1845 CONTAINS THREE *Sau1* S SUBUNITS.

>ETD06224 THIS IS TRD N+Q ACC-5-RTGT

MSNTQTKNVPELKFPEFEGEWEEKKLGEFAGKVTKKNVDKKYIETLTNSAELGIISQKDY
FDKEISNIDNIKKYYVVEENDFVYNPRISNYAPFGPVNRNKLGKKGVMSPLYTVFKIQNI
DLNFIEFYFKSSKWYRFMALNGDSGARADRFSIKNRTFMEMPLHIPCMDEQIKIGQFFSK
LDRQIELEEQKLELLQQQKKGYMQKIFSQELRFKDENGNDYPEWEERRFADIFKFHNKLR
KPIKENLRVKGSYPYYGATGIIDYVDDFIFDGNYLLIGEDGANIITRSAPLVYLVNGKFW
VNNHAHILSPLNGNIQYLYQVAELVNYEKYNTGTAQPKLNIQNLKIISVVISTNLEEQQK
IGSFLSKLDRQIDLEEQKLELLQQRKKALLKSMFV

>ETD11204 THIS HAS TWO NOVEL TRDS, NOVEL 2 + NOVEL 3.

MTEQINTPELRFPEFKNEWSYDLVSDVVTNKSKKFDPKKEEAKKDIELDSIEQNTGRLLD
TYISNDFTSQKNKFNKGNVLYSKLRPYLNKYYYATIDGVCSSEIWVLNTLNKDVLANKFL
YYFIQTNRFSSVTNKSAGSKMPRADWELVKNIRLYKGSIEEQEKIGYFFSKLDRQIELEE
KKLELLEQQKKGYMQKIFAQELRFKDENGNDYPDWVTKKLGDIGKVAMNKRIYKNETTEN
GEIPFYKIGNFGKNADTFITREKFDEYKEKYPYPNVGDILISASGSIGRTIEYTGEDAYY
QDSNIVWLNHNDEVINKYLKYFYKIVKWSGIEGTTIKRLYNKNILNTKIELPTVEEQYKM
ANFLSKLDKIIDIQIEKIELLKQRKQGLLQKMFV

>ETD09130 THIS HAS A NOVEL TRD (NOVEL 4) PAIRED WITH TRD f*

1MSNTQKKNVPELRFPEFEGEWKDVKFVSIFQEVSNKTSDLAKYPLFSLTVEKGITPKTER
61YKRDFLVKKSDNFKIVEPRDIVYNPMNVTLGAIDLSKYNYDIALSGYYHVMKIINSFNPD
121FISNFLKTEKMIIHYKKIATGSLMEKQRVHFSEFKNIIKKFPTNKEQQKIGDFFSKLDRQ
181IELQVQKLELLQQQKKGYMQKIFSQELRFKDENGEDYPDWKEKKLGDITEQSMYGIGASA
241TRFDSKNIYIRITDIDEKSRKLNYQNLTTPDELNNKYKLKRNDILFARTGASTGKSYIHK
301EEKDIYNYYFAGFLIKFEIDEQNNPLFIYQFTLTSKFNKWVKVMSVRSGQPGINSEEYAK
361LPLVLPNKLEQQKIAEFLDRFDQQIELEKQKIEILQQQKKGLLQSMFI

PROMALS ALIGNMENT OF TRD AMINO ACID SEQUENCES WITH SECONDARY STRUCTURE PREDICTIONS.

“e” means beta strand and “h” means alpha helix in the consensus secondary structure.

PROMALS alignment of all first TRDs.

```
Conservation:             999876797999999798898665 5 5
NOVEL_4_189_           1  M--TEQINTPELRFPEFKNEWSYDLVSDVVTNKSKKFDPKK------------EEAKKDIELDSIEQNTG   56
Z_GAC_191_             1  MSNTQTKNVPELRFPGFEGEYSLDIFGNLATNKSEKFNPQN------------ENASIDIELDCIEQNTG   58
NOVEL2_194             1  MSNTQKKNVPELRFPEFEGEWKDVKFVSIFQEVSNKTSDLA------------KYPLFSLTVEKGITPKT   58
NOVEL1_199             1  MSNTQKKNVPELRFPGFEGEWEEKKLGEVATFAKGKLGAKK---------DVSQNGVPVILYGELYTKYG   61
R_GARA_192             1  MSNTQKKNVPELRFPGFEGEWEEKKLGEVAKIYDGTHQTPK----------YTNEGIKFLSVENIKTLNS   60
J_GGA_172              1  MSNTQKKNVPELRFPEFEGEWEERKLGDLIKVNSGKDYKH-----------LDKGDIPVYGTGGYMTS--   57
N_ACC_198              1  MSNTQKKNVPELRFPGFEGEWEEKKLGEFAGKVTQKNVDKKY-----IETLTNSAELGIISQKDYFDKEI   65
O_CAAC_195             1  MSNKQKKNVPELRFPGFEGEWEEKKLGEVGTFTSGGTPLKS-------KSEYWNGDIPWITTGDIHNIKR   63
T_CAAG_199             1  MSNTQTKNVPELRFPGFEGEWEEKELGEIFQIISGSTPLKSN------KEFYENGNINWVKTTDLNNSKV   64
C_GWAG_206             1  MSNTQTKNVPELRFPGFEGEWEEKQVGELLEFKNGLNKGKE----------YFGSGSSIVNFKDVFNNRS   60
M_CAG_203              1  MSNTQTKNVPELRFPGFEGEWEEKKLEDLGLFQKSYSFSRA---------KEGNGKTKHIHYGDIHSKFK   61
X_TCTA_192             1  MSNTQKKNVPELRFPGFEGEWEEKQFADFTKINQGLQIAINE------RKTEYSPELYFYITNEFLRPNS   64
B_AGG_199              1  MSNTQKKNVPELRFPGFEGEWEEKQLGDLTDRVIRKNKNLES-----KKPLTISGQLGLIDQTEYFSKSV   65
A_CCAY_203             1  MSNTQKKNVPELRFPGFEGEWEEKQLGDLTTKIGSGKTPKGG------SENYTNKGIPFLRSQNIRNGKL   64
e*_GAG_190             1  MSNTQKKNVPELRFPGFEGEWEEKSISSFLKESKIKGSNGS---------HAKKLTVKLWGKGVVPKKET   61
V_CNGA_210             1  MSNTGKMNVPELRFPGFEGEWEEKELRELRNPKDKYSYTGGPFGSDLKKSDYTTDGIQIIQLQNIGDGYF   70
b*_GGHA_200_           1  MSNTQKKNAPELRFPEFEGEWKEKKLEDTLEFIKDGTHGTH---------ENVNNGPWLLSAKNIKNNKI   61
Consensus_ss:                       eee        eeeeeeeeeeee                       eeeeeeeeee

Conservation:                                                     5
NOVEL_4_189_          57  RLLDTY-----ISNDFTSQKNKFNKGNVLYSKLRPY---LNKYYYATI----DGVCSSEIWVLNTLNK-D  113
Z_GAC_191_            59  RLIKIY-----NSKEFSSQKNKFNPQNVLYGKLRPY---LNKYYFTKK----SGVCSSEIWVLKSTKE-D  115
NOVEL2_194            59  ERYKRDFL-----VKKSDNFKIVEPRDIVYNPMNVT---LGAIDLSKYN--YDIALSGYYHVMKIIN---  115
NOVEL1_199            62  AIVSK--IFS-KTDIPENKLKMAKKNDVLIPSSGETAIDIATASCIYLN--KGVAVGGDINILTPQ----  122
R_GARA_192            61  SK-----YIS-EEAFEKEFKIRPEFGDILMTRIGDI----GTPNIVSSN--EKFAYYVSLALLKTK----  114
J_GGA_172             58  -----------------VSEPLSEIDAVGIGRKGTI----NKPYLLEA----PFWTVDTLFYCTPEK---   99
N_ACC_198             66  S-----------NIDNIKKYYVVEENDFVYNPRMSNYAPFGPVNRNKLG--KKGVMSPLYTVFKIQ----  118
O_CAAC_195            64  ENITN--FIT-EKGLNESSAKLITNEAILIAMYGQGK-TRGMSAILNF----EATTNQACAIYQ-----T  120
T_CAAG_199            65  THSKE--KIT-EYAMKSLKLKLVPKNSVLIAMYGGFN-QIGRTGLLK----IDATINQAISALLMNH---  123
C_GWAG_206            61  INTNNLTGKV-NVNSKELKNYSVEKGDVFFTRTSEVIGEIGYPSVILNDP-ENTVFSGFVLRGRPKSGID  128
M_CAG_203             62  TVLDSD-GNI-PNIIEKAVFELIQKGDIVFADASEDYSDLGKAVMIDFEP-NSLISGLHTHLFRPLN---  125
X_TCTA_192            65  QTKYF--------IENPPQSVIANKEDILMTRTGN----TGKVVTNV----FGAFHNNFFKIKFDKN---  115
B_AGG_199             66  SSK------------NLENYTLIKNGEFAYNKSYSNGYPLGAIKRLTRY--DSGVLSSLYICFSIKS---  118
A_CCAY_203            65  NLNDLV-YIS-KDIDDEMKNSRTYYGDVLLNITGAS---IGRTAINSIVE-THANLNQHVCIIRLKK---  125
e*_GAG_190            62  F-----------KGSDNTQYYKRKAGQLMYGKLDFL---NCAFGIVPDS--LNNYESTIDSPSFDFI---  112
V_CNGA_210            71  YNSNKV-FTS-NEKAEVLKSCNVFPGDIVIAKMADP---IARAAIVPDNNIGKYLMASDGIRLSVDT--V  133
b*_GGHA_200_           62  IISSDDRKISESDYKKIYKNYKLEKGDLLLTIVGTI----GRAAIVKNP--NNIAFQRSVAILKTKA---  122
Consensus_ss:                    e            ee     eeeeee       eeeeee       eeee  eeeee

Conservation:                  9                                     5    5    99 799 9999898998
NOVEL_4_189_         114  VLANKFLYYFIQTNRFSS-VTNKSAG----SKMPRADWELVKNIRLYKGS-IEEQEKIGYFFSKLDRQIE  177
Z_GAC_191_           116  KLLNLFLYYFIQTKRYSD-VASKSAG----SKMPRADWGLIENIRVYFPE-LCEQQKIGQFFSKLDRQIE  179
NOVEL2_194           116  SFNPDFISNFLKTEKMIIHYKKIATGS--LMEKQRVHFSEFKNIIKKFPT-NKEQQKIGDFFSKLDRQIE  182
NOVEL1_199           123  KQDGRFISLSINGI-NKNELSKYAQG----KTVVHLYNNDIKNLKIAFPSEFEEQVRIGNFFSKLDRQIE  187
R_GARA_192           115  NLNSYFLKNLILSSSIQNELWRKTLHV---AFPKKINKNEIGKIKINYPK-KQEQQKIGQFFSKLDRQIE  180
J_GGA_172            100  EADILFILSLFRKINWKL----YDES----TGVPSLSKQTINKINRLVPT-NKEQQKIGEFFSKLDRQIE  160
N_ACC_198            119  NIDLNFIEFYFKSSKWYRFMALNGDSGA-RADRFSIKDRTFMEMPLHIPC-MDEQIKIGQFFSKLDRQIE  186
O_CAAC_195           121  NQNINFVFQYFQK--LYEFLRSLSNE----GSQKNLSLSLLKEITLNYPN-EQEQKKIGDFFSKLDRQIE  183
T_CAAG_199           124  ETNPEFIQAFLNYQ-VKGWKRYAASS----RKDPNITKKDIEQFKVPYVS-INEQQKIGEFFSKIDHQIE  187
C_GWAG_206           129  LINNNFKRYVFFTNSFRKEMITKSSM----TTRALTSGTAINKMKVIYPVSAKEQKKIGDFFSKLDRQIE  194
M_CAG_203            126  NAISNFLIFYTKTLSYKKFIRQQGTG----ISVLGISKKSLLNLNVLIPRSELEQQKIGQFFSKLDRQIE  191
X_TCTA_192           116  LYDRLFLVEVLNSSKIQNKILSLAGS----STIPDLNHSDFYSISSSYPL-LREQQKIGKFFSKLDRQIE  180
B_AGG_199            119  EMSKDFMEAYFDSTHWYREVSGIAVEGARNHGLLNVSVNDFFTILIKYPS-LEEQQKIGKFFSKLDRQIE  187
A_CCAY_203           126  EYYYIFFGQYLLSRKGKRKIFLAQSG----GSREGLNFKEIANLKIFTPTIFEEQQKIGKFFSKLDRQIE  191
e*_GAG_190           113  NGDSKFLLERIKLKSFYKKFGDIANGS---RKAKRINQDTFLSLPVFAPK-YDEQLRIGEFFSKLDRQIE  178
V_CNGA_210           134  HFNTKFVLECINRKSFRKKVEDNSSG----STRMRIGLSTLGSLTLKTTT-LKEQQKIGQFFSKLDRQIV  198
b*_GGHA_200_         123  TYDVGFIFQLFQTKYFKNLLLRKQVV----SAQPGLYLGDIRKIKISITNIIEEQRKIGIFFSKLDRQIE  188
Consensus_ss:                hhhhhhhhh hhhhhhhhhh              hhhhh  ee     hhhhhhhhhhhhhhhhhhh

Conservation:             977899999899
NOVEL_4_189_         178  LEEKKLELLEQQ  189
Z_GAC_191_           180  LEEQKLELLQQQ  191
NOVEL2_194           183  LQVQKLELLQQQ  194
NOVEL1_199           188  LEEQKLELLQQQ  199
R_GARA_192           181  LEEQKLELLQQQ  192
J_GGA_172            161  LEEQKLELLQQQ  172
N_ACC_198            187  LEEQKLELLQQQ  198
O_CAAC_195           184  LEEQKLELLQQQ  195
T_CAAG_199           188  LEEQKLELLQQQ  199
C_GWAG_206           195  LEEQKLELLQQQ  206
M_CAG_203            192  LEEQKLELLQQQ  203
X_TCTA_192           181  LEEQKLELLQQQ  192
B_AGG_199            188  LEEQKLELLQQQ  199
A_CCAY_203           192  LEEQKLELLQQQ  203
e*_GAG_190           179  LQKQKLELLQQQ  190
V_CNGA_210           199  LEEQKLELLQQQ  210
b*_GGHA_200_         189  LEEQKLELLQQQ  200
Consensus_ss:             hhhhhhhhhh
```

PROMALS alignment of all second TRDs.

```
Conservation:       998979999799999989696797 9    5
NOVEL3_205       1  KKGYMQKIFAQELRFKDENGNDYPDWVTKKLGDIGKVAMNKRIYKNE-------TTENGEIPFYKIGNFG   63
S_GCA_200        1  KKGYMQKIFSQELRFKDENGNDYPDWTNERLGEVTTVTMGQSPKSVN-----YTDNSNDTVLIQGNADIE   65
d*_CYAA_220      1  KKGYMQKIFSQELRFKDENGNDYPEWENVMLQKVLKDKTEGIKRGPFGGALKKDIFVESGYAVYEQRNAI   70
a*_GAA_208       1  KKGYMQKIFSQELRFKDENGNDYPEWENKRIEDIANVNKGFTPSTNN-----NEYWDNNDKNWLSIAGMN   65
E_TCAY_194       1  KKGYMQKIFSQELRFKDENGNDYPEWEETTIKEIAQINXGKKDTKD--------AITNGSYDFYVRSPIV   62
W_CRAA_211       1  KKGYMQKIFSQELRFKDENGNDYPDWEEKQLGELSQIVRGASPRPIK---DPKWFNKESDIGWLRISDVT   67
Q_ACAY_197       1  KKGYMQKIFSQELRFKDENGEDYSEWEERRFADIFKFHNKLRKPIKE------NLRVKGSYPYYGATGII   64
G_ACA_196        1  KKGYMQKIFTQELRFKDENGEEYPEWENKFIKDIFIFENNRRKPITS------SLREKGLYPYYGATGII   64
f*_GAAY_224      1  KKGYMQKIFSQELRFKDENGEDYPDWKEKKLGDITEQSMYGIGASA--------TRFDSKNIYIRITDID   62
L_TTTA_213       1  KKGYMQKIFSQELRFKDENGNDYPNWRTIELKNILENIVDNRGKTPD-------NAPSEKYPLLEVNALG   63
Y_CTA_209        1  KKGYMQKIFSQELRFKDENGNDYPDWEKKKLKEIACVYTGNTPSKKE-----NIYWNKGEYVWVTPTDIN   65
U_GAY_193        1  KKGYMQKIFSQELRFKDENGEDYPDWEVTTIQNITKYTSSKKSSNQY-----ADKDNSKGYPVYDAVQEI   65
I_YTCA_220       1  KKGYMQKIFSQELRFKNENGNDYPDWERIKFFDVIDKVIDFRGRTPK---KLNMEWSDEGYLALSAVNVK   67
K_CGA_212        1  KKGYMQKIFSQELRFKDENGNDYPKWEEKKIEDIASQVYGGGTPNTK-----IKEFWNGDIPWIQSSDVK   65
D_ATC_204        1  KKGYMQKIFSQELRFKDENGEDYPHWENSKIEKYLKERNERSDK--------------GQMLSVTINSGI   56
c*_GAY_209       1  KKGYLQKIFSQELRFKDENGNDYPEWRFARFKDFMYKPINIRPAINI-----------SKSELLTVKLHC   59
P_AGG_214        1  KKGYMQKIFSQELRFKDESGNDYPDWEEKELGEVADRVIRKNKNFES----KKPLTISGQLGLIDQTEYF   66
F_TTAA_216       1  KKGYMQKIFSQELRFKDEEGKDYPDWKSKSIQEIFENKGGTALETE--------FNFDGNYKVISIGSYS   62
H_TAC_206        1  KKCYIQKIFSQELRFKDEEGNYYKGWNKKQLKDVLEFSNKRTINE-------------NEYPVLTSSRQG   57
Consensus_ss:         hhhhhh                    eeeeeeheeeee                      eeeeeeeeee

Conservation:                                        5              7
NOVEL3_205      64  KNADTFITR--EKF----DEYKEKYPYPNVGD-ILISASG------SIGRTIEYTG--EDA-YYQDSNIV  117
S_GCA_200       66  NGLINPR-----------IYTREVTKLIQKDE-IILTVRA------PVGKLAMAQIN-----ACIGRGVC  112
d*_CYAA_220     71  YDISNFRYY-INEN----KYKEMQSFSVQPND-IIMSCSG------TIGRLALIPH-NYTK-GIINQALI  126
a*_GAA_208        66  QKYLY-KGN-KGIS----KDAAKNYMKVKNDT-LIMSFKL------TIGKLAIVKAP-----LYTNEAIC  117
E_TCAY_194      63  YK--------------------INTFSYEGEAILTVGDGV------GVGKVFHYVNGK----FDYHQRVY  102
W_CRAA_211      68  NQNGKIYHLEQKLS----IEGQEKTRVLVTTH-LLLSIAA------SIGKPVMNFVK-----TGVHDGFL  121
Q_ACAY_197      65  DYV--------------------DDFIFDGNY-LLIGEDGANIITRSAPLVYLVNGK-----FWVNNHAH  108
G_ACA_196       65  DYV--------------------KDYLFNNEERLLIGEDGAK-WGQFETSSFIANGQ-----YWVNNHAH  108
f*_GAAY_224     63  EKSRKLNYQ-NLTT----PDELNNKYKLKRND-ILFARTGA-----STGKSYIHKEEKDIYNYYFAGFLI  121
L_TTTA_213      64  YYRPAYIKV-SKFVS-ENTYNNWFREHLKEND-ILFSTVG------NTGIVSLMDNYK----AVIAQNIV  120
Y_CTA_209       66  NSKNIYESE-NKLT----QEGYKKARQLPENT-LLVTCIA------SIGKNAILRKQ-----GSCNQQIN  118
U_GAY_193       66  GK--------------------DSNYDIEESY-ISILKDGA-----GVGRLNLRPGK-----SSVIGTMG  104
I_YTCA_220      68  KGYIDFNVE-AKYGNLDLYTRWMRGNELYKGQ-VLFTTEA------PMGNVAQVPDNKG---YILSQRTI  126
K_CGA_212       66  VNDLILQQCNKFISK--NSIELSSAKLIPANS-IAIVTRV------GVGKLCLVEFD-----YATSQDFL  121
D_ATC_204       57  IKFSELDR--KDNS----SKNKSNYKVVRKND-IAYNSMRM-----WQGASGKSNYN-----GIVSPAYT  109
c*_GAY_209      60  KGIEK-ANI-NRVL----KLGATNYYKRFEGQ-FIYGKQNF-----FNGAFDIVPKKFDG--LYSSSDVP  115
P_AGG_214       67  SKSVS-------------SKNLENYTLIKNGE-FAYNKSYSN--GYPLGAIKRLTRYDS---GVLSSLYI  117
F_TTAA_216      63  INSTYNDQN-IRVN----KNKKTEKYILSKGD-LAMVLNDKTKDGKIIGRSIFIDKDNQ---YIYNQRTE  123
H_TAC_206       58  LILQSDYYKDRKTF----AESNIGYFILPKNH-ITYRSRS------DDGIFKFNLNLMIDV-GIISKYYP  115
Consensus_ss:                                ee      eeeee          eeeeee        eeee  ee

Conservation:                   6                             5            7    98 76  7
NOVEL3_205     118  WLNHND-EVINKYLKYFYKI-----VKWSGIEG----TTIKRLYNKNILNTKIELPT-VEEQYKMANFLS  176
S_GCA_200      113  SIKG------DKFLYYFLEWFATQNKWIRFSQG----STFESISGNDIRNIHIKIPV-EDERTKIIKLLN  171
d*_CYAA_220    127  RFRTNH-KIRSEFFLIFMRSNQMQRKILEANPG---SAITNLVPVKELKLIPFPLPV-KFEQDKISQFIH  191
a*_GAA_208     118  HFIWKVNKINTEFIYYYLNSL---NISTFGVQA----VKGVTLNNDSINSIIVKLPN-EEEQNIIAKFLL  179
E_TCAY_194     103  KISDFK-NYYGLLLFYYFSQN-FLKETKKYSAK----TSVDSVRKDMIANMKVPRPI-YIEQKKIGQFIK  165
W_CRAA_211     122  IFLKP--KFNLFFMYYWLEYF--KDKWSKYGQP----GSQVNLNSEIVKSQTLNMPS-NHEQEKVGQFFN  182
Q_ACAY_197     109  ILSPL--NGNIQYLYQVAEL----VNYEKYNTG----TAQPKLNIQNLKIINVVISTNLEEQQKIGSFLS  168
G_ACA_196      109  VVKSN--DHNLFFMNYYLNF----KELRAFVTG----NAPAKLTHANLCNINLKIPC-LTEQDKVSALLK  167
f*_GAAY_224    122  KFKINE-QNSPLFIYQFTLTSKFNKWVKVMSVR----SGQPGINSEEYAKLPLVLPN-KLEQQKIAKFLD  185
L_TTTA_213     121  GLRVNN-NNLPSFIYYMLSYKGNQKKIKRIQMG----AVQPSVKVSQFKFIKYLVPI-KDEQEKVAKLLI  184
Y_CTA_209      119  AVVPFE-NINIDYLYYISDSL--STFMKSIAGK----TATQIVNKNTFENLEIYLAP-FEEQNKIADLIS  180
U_GAY_193      105  YIQSN--NVDIEFLYYRMKVV----DFKKYIIG----STIPHLYFKDYSKETLYIPSSIQEQAKIGMFIS  164
I_YTCA_220     127  AFNSNE-KITDNFLASLLSSENVYNDLLKLCSG----ATAKGVSQKNLNRLYVTIPHSISEQEEIAEFFR  191
K_CGA_212      122  SLSSLK--YDKLYSLYSL--LYTMKKISANLQG----TSIKGITKKELLDSIIKIPHNLEEQQKIGDLFY  183
D_ATC_204      110  VLYPTQ-NTSSLFIGYKFKTHRMIHKFKINSQGL--TSDTWNLKYKQLKNINIDIPV-LEEQEKIGDFFK  175
c*_GAY_209     116  AFEINTEKIEPNYFISYISRPSFYKSKEKYSTG----TGSKRIHENTVLNFSLHLPC-LNEQLKIASFVC  180
P_AGG_214      118  CFSIKS-EMSKDFMEAYFDSTHWYREVSGIAVEGARNHGLLNISVNDFFTILIKYPS-LEEQRKIGDFFI  185
F_TTAA_216     124  RLIPFA-ENDNKFLWFLMNTDLIRNKIKGMMQG----ATQVYINYSSIKLISIQLPL-LEEQQKIRGFLE  187
H_TAC_206      116  VFKGI--DANQYYLTLHLNYQ-LKKEYIKYATG----TSQLVLSQKDLQNIKTKLPS-YEEQQKIGDFFS  177
Consensus_ss:       eee       hhhhhhhhh hhhhhhhhhh              hhhhh  ee      hhhhhhhhhhhh
Conservation:         5        75  56  97 697 786
NOVEL3_205     177  KLDKIIDIQIEKIELLKQRKQGLLQKMFV----------  205
S_GCA_200      172  SLDVLNSKTDLKIQNLKQRKQSLLQKIFV----------  200
d*_CYAA_220    192  IINRRIEQSEKKIESLKNRKQGFLQKLFV----------  220
a*_GAA_208     180  EVDKTVNNQLVKTKLLKQRKKGLLQRMFV----------  208
E_TCAY_194     166  RVDNKTKIQKQVIELLKQRKKALLQKMFI----------  194
W_CRAA_211     183  RNEKLIELQQEKIMYIKRCKQVLLQKMFI----------  211
Q_ACAY_197     169  KLDRQIDLEEQKLELLQQRKKALLKSMFV----------  197
G_ACA_196      168  SIDNKMNNQMNRIELLKERKKELLQKMFI----------  196
f*_GAAY_224    186  RFDRQIELEKQKIEILQQQKKGLLQSMFI----------  214
L_TTTA_213     185  EIDKLVNKQLIKIELLQQRKKALLKSMFI----------  213
Y_CTA_209      181  SLEELIEKQASKLIKMKSRKQGMLQIMFI----------  209
U_GAY_193      165  NLDKLIENKNLKLNCLKQLKQGLLQSMFI----------  193
I_YTCA_220     192  KINQLVELQKYKIEHTKSQKQVFLQKMFI----------  220
K_CGA_212      184  KIDKYISFNKCKIEMLKSLKQGLLKKMFI----------  212
D_ATC_204      176  KMDILISKQKIKIEILEKEKQSFLQKMFL----------  204
c*_GAY_209     181  FLNRKIELLERKIYLIKKQKQALLQQMFI----------  209
P_AGG_214      186  KLDRQIELEEQKLELLQQRKKALLKSMLI----------  214
F_TTAA_216     188  VLSGITTKQLHKIDQLKERKKAFLQKMFI----------  216
H_TAC_206      178  EIDRLVEKQSSKVGRLKVRKKELLQKMFV----------  206
Consensus_ss:       hhhhhhhhhhhhhhhhhhhhhhhhhhh
```

PROMALS alignment of all TRDs.

```
Conservation:                                799997576    6 85   5
CC80-3 f*                       1  ----------QELRFKDENGEDYPDWKEKKLGDITEQSMYGIGASA---------TRFDSKNIYIRITDI   51
CC45-1L                         1  ----------QELRFKDENGNDYPNWRTIELKNILENIVDNRGKTP--------DNAPSEKYPLLEVNAL   52
CC97 c*                         1  ----------QELRFKDENGNDYPEWRFARFKDFMYKPINIRPAIN-------ISKSELLTVKLHCK-GI   52
CC22-1I                         1  ----------QELRFKNENGNDYPDWERIKFFDVIDKVIDFRGRTPKK----LNMEWSDEGYLALSAVNV   56
CC873D                          1  ----------QELRFKDENGEDYPHWENSKIEKYLKERNERSDKGQM------LSVTIN--SGIIKFSEL   52
CC5-1D                          1  ----------QELRFKDENGEDYPDWENSKIEKYLKERNERSDKGQM------LSVTIN--SGIIKFSEL   52
CC30-1D                         1  ----------QELRFKDENSEDYPHWENSKIEKYLKERNERSDKGQM------LSVTIN--SGIIKFSEL   52
CC5-2H                          1  ----------QELRFKDEEGNYYKGWNKKQLKDVLEFSNKRTINE--------------NEYPVLTSSRQ   46
CC133-2fromED133 d*             1  ----------QELRFKDENGNDYPEWENVMLQKVLKDKTEGIKRGPFGG-ALKKDIFVESGYAVYEQRNA   59
CC72-2S                         1  ----------QELRFKDENGNDYPDWTNERLGEVTTVTMGQSPKSVN------YTDNSNDTVLIQGNADI   54
CC93-3 a*                       1  ----------QELRFKDENGNDYPEWENKRIEDIANVNKGFTPSTNN------NEYWDNNDKNWLSIAGM   54
CC93-2K                         1  ----------QELRFKDENGNDYPKWEEKKIEDIASQVYGGGTPNTK------IKEFWNGDIPWIQSSDV   54
CC30-2K                         1  ----------QELRFKDENGNDYPNWEEKKIEDIASQVYGGGTPNTK------IKEFWNGDIPWIQSSDV   54
CC80-2W                         1  ----------QELRFKDENGNDYPDWEEKQLGELSQIVRGASPRPIKD----PKWFNKESDIGWLRISDV   56
CC75-2W                         1  ----------QELRFKDENGNDYPDWEEKQLGELSQIVRGASPRPIKD----PKWFNKESDIGWLRISDV   56
CC59Q                           1  ----------QELRFKDENGEDYSEWEERRFADIFKFHNKLRKPIK-------ENLRVKGSYPYYGATGI   53
CC72-1Q                         1  ----------QELRFKDENGNDYPEWEERRFADIFKFHNKLRKPIK-------ENLRVKGSYPYYGATGI   53
CC1-2G                          1  ----------QELRFKDENGEEYPEWENKFIKDIFIFENNRRKPIT-------SSLREKGLYPYYGATGI   53
ST425-1E                        1  ----------QELRFKDENGNDYPEWEETTIKEIAQINTGKKDTKD---------AITNGSYDFYVRSPI   51
CC15TRD2E                       1  ----------QELRFKDENGNDYPEWEETTIKEIAQINXGKKDTKD---------AITNGSYDFYVRSPI   51
CC133_771E                      1  ----------QELRFKDENGDDYPEWEETTIKEIAQINTGKKDTKD---------AITNGSYDFYVRSPI   51
CC398-1E                        1  ----------QELRFKDENGKDYPEWEETTIKEIAQINTGKKDTKD---------AITNGSYDFYVRSPI   51
CC80-1Y                         1  ----------QELRFKDENGNDYPDWEKKKLKEIACVYTGNTPSKKE------NIYWNKGEYVWVTPTDI   54
CC75-1U                         1  ----------QELRFKDENGEDYPDWEVTTIQNITKYTSSKKSSNQY------ADKDNSKGYPVYDAVQE   54
CC1-1F                          1  KKGYMQKIFSQELRFKDEEGKDYPDWKSKSIQEIFENKGGTALETE---------FNFDGNYKVISIGSY   61
CC873 e*                        1  -MSNTQKKNVPELRFPGFE----GEWEEKSISSFLKESKIKGSNGS---------HAKKLTVKLWGKGVV   56
CC80-2Z                         1  -MSNTQTKNVPELRFPGFE----GEYSLDIFGNLATNKSEKFNPQN-------ENASIDIELDCIEQNTG   58
CC80-3XS.Sau11819ORF2227P       1  -MSNTQKKNVPELRFPEFE----GEWEEKQFADFTKINQGLQIAINE------RKTEYSPELYFYITNEF   59
CC80-1X                         1  -MSNTQKKNVPELRFPGFE----GEWEEKQFADFTKINQGLQIAINE------RKTEYSPELYFYITNEF   59
CC75-1T                         1  -MSNTQTKNVPELRFPGFE----GEWEEKELGEIFQIISGSTPLKSN------KEFYENGNINWVKTTDL   59
ST130-1T                        1  -MSNTQKKNVPELRFPGFE----GEWEEKKLGEIFQIISGSTPLKSN------KKFYENGNINWVKTTDL   59
CC93-3M                         1  -MSNTQTKNVPELRFPGFE----GEWEEKKLEDLGLFQKSYSFSRA---------KEGNGKTKHIHYGDI   56
CC133_771-1strain32320Hsd       1  -MSNTQTKNVPELRFPGFE----GEWEEKKLGDLGLFQKSYSFSRA---------KEGNGKTKHIHYGDI   56
CC133-2fromED133J               1  -MSNTQKKNVPELRFPGFE----GEWEEKKLESIIKVNSGKDYKH-----------LDKGDIPVYGTGGY   54
CC72-2J                         1  -MSNTQKKNVPELRFPEFE----GEWEEKQLGNIIKVNSGKDYKH-----------LDKGDIPVYGTGGY   54
CC51TRD1J                       1  -MSNTQTKNVPELRFPGFE----GEWEEKKLEDIIKVNSGKDYKH-----------LDKGDIPVYGTGGY   54
CC30-2strainMRSA252HsdSJ        1  -MSNTQTKNVPELRFPGFE----GEWEEKKLGDLIKVNSGKDYKH-----------LEKGDIPVYGTGGY   54
CC59-1J                         1  -MSNTQKKNVPELRFPEFE----GEWEERKLGDLIKVNSGKDYKH-----------LDKGDIPVYGTGGY   54
CC72-1R                         1  -MSNTQKKNVPELRFPGFE----GEWEEKKLGEVAKIYDGTHQTPK----------YTNEGIKFLSVENI   55
CC15TRD1O                       1  -MSNKQKKNVPELRFPGFE----GEWEEKKLGEVGTFTSGGTPLKS-------KSEYWNGDIPWITTGDI   58
CC398-1strain398HsdSN           1  -MSNTQKKNVPELRFPGFE----GEWEEKKLGEFAGKVTQKNVDKKY-----IETLTNSAELGIISQKDY   60
ST425-1C                        1  -MSNTQTKNVPELRFPGFE----GEWEEKQVGELLEFKNGLNKGKE----------YFGSGSSIVNFKDV   55
CC30-1strainMRSA252HsdSC        1  -MSNTQTKNVPELRFPGFE----GEWEEKKVGELLEFKNGLNKGKE----------YFGSGSSIVNFKDV   55
CC45-1strain3067HsdSC           1  -MSNTQKKNVPELRFPGFE----GEWEEKKVGELLEFKNGLNKGKE----------YFGSGSSIVNFKDV   55
CC97A                           1  -MSNTQKKNVPELRFPGFE----GEWEEKQLGDLTTKIGSGKTPKGG------SENYTNKGIPFLRSQNI   59
CC1-2strainMW2HsdSA             1  -MSNTQTKNVPELRFPGFE----GEWEEKKLGNLTTKIGSGKTPKGG------SENYTNKGIPFLRSQNI   59
CC1-1strainMW2HsdSA             1  -MSNTQKKNVPELRFPGFE----GEWEEKKLGDLTTKIGSGKTPKGG------SENYTNKGIPFLRSQNI   59
CC5-2strainN315HsdSA            1  -MSNTQTKNVPELRFPGFE----GEWEEKKLGNLTTKIGSGKTPKGG------SENYTNKGIPFLRSQNI   59
CC75-2V                         1  -MSNTGKMNVPELRFPGFE----GEWEEKELRELRNPKDKYSYTGGPFGSDLKKSDYTTDGIQIIQLQNI   65
CC22-1strain5096HsdSB           1  -MSNTQKKNVPELRFPGFE----GEWEEKKLGDLTDRVIRKNKNLES-----KKPLTISGQLGLIDQTEY   60
CC51TRD2P                       1  ----------QELRFKDESGNDYPDWEEKELGEVADRVIRKNKNFES-----KKPLTISGQLGLIDQTEY   55
CC5-1strainN315HsdSB            1  -MSNTQKKNVPELRFPGFE----GEWEEKQLGDLTDRVIRKNKNLES-----KKPLTISGQLGLIDQTEY   60
CC93-2 b*                       1  -MSNTQKKNAPELRFPEFE----GEWKEKKLEDTLEFIKDGTHGTH---------ENVNNGPWLLSAKNI   56
Consensus_ss:                                           eeeeeeeeeeee                                   eeeeeee e
```

```
Conservation:                                                       5              5
CC80-3 f*                      52  DEKSRKLN-YQNLTTP----DELNNKYKLKRNDILFARTGAST-----GKS-YIHKEEKDIYNYYFAGFL  110
CC45-1L                        53  GYYRPAYI-KVSKFVSE-NTYNNWFREHLKENDILFSTVGNT------GIV-SLMDN----YKAVIAQNI  109
CC97 c*                      53  EKANINRV----------LKLGATNYYKRFEGQFIYGKQNFFN-----GAF-DIVPKK--FDGLYSSSDV  104
CC22-1I                        57  KKGYIDFNVEAKYGNLD-LYTRWMRGNELYKGQVLFTTEAPM------GNV-AQVPD---NKGYILSQRT  115
CC873D                         53  DRKDN-------------SSKNKSNYKVVRKNDIAYNSMRMWQ-----GAS-GKSNY-----NGIVSPAY   98
CC5-1D                         53  DRKDN-------------SSKDKSNYKVVRKNDIAYNSMRMWQ-----GAS-GKSNY-----NGIVSPAY   98
CC30-1D                        53  DRKDN-------------SSKDKSNYKVVRKNDIAYNSMRMWQ-----GAS-GRSNY-----NGIVSPAY   98
CC5-2H                         47  GLILQSD---YYKDRKT-FAESNIGYFILPKNHITYRSRSDD------GIFKFNLNLM--IDVGIISKYY  104
CC133-2fromED133 d*            60  IYDISNF----RYYINE-NKYKEMQSFSVQPNDIIMSCSGTI------GRL-ALIPHN--YTKGIINQAL  115
CC72-2S                        55  ENGL----------INP-RIYTREVTKLIQKDEIILTVRAPV------GKL-AMAQI-----NACIGRGV  101
CC93-3 a*                      55  NQKYLYK---GNKGIS---KDAAKNYMKVKNDTLIMSFKLTI------GKL-AIVKA-----PLYTNEAI  106
CC93-2K                        55  KVNDLILQ-QCNKFISK-NSIELSSAKLIPANSIAIVTRVGV------GKL-CLVEF-----DYATSQDF  110
CC30-2K                        55  KVNDLILR-QCNKFISK-NSIELSSAKLIPANSIAIVTRVGV------GKL-CLVEF-----DYATSQDF  110
CC80-2W                        57  TNQNGKIY-HLEQKLS---IEGQEKTRVLVTTHLLLSIAASI------GKP-VMNFV-----KTGVHDGF  110
CC75-2W                        57  TNQNGKIY-HLEQKLS---IEGQEKTRVLVTTHLLLSIAASI------GKP-VMNFV-----KTGVHDGF  110
CC59Q                          54  IDYV--------------------DDFIFDGNYLLIGEDGA-NIITRSAPLVYLVNG-----KFWVNNHA   97
CC72-1Q                        54  IDYV--------------------DDFIFDGNYLLIGEDGA-NIITRSAPLVYLVNG-----KFWVNNHA   97
CC1-2G                         54  IDYVK-------------------DYLFNNEERLLIGEDGA-KWGQFETSS-FIANG-----QYWVNNHA   97
ST425-1E                       52  VYKI--------------------NTFSYEGEAILTVGDGVGV-----GKV-FHYVN----GKFDYHQRV   91
CC15TRD2E                      52  VYKI--------------------NTFSYEGEAILTVGDGVGV-----GKV-FHYVN----GKFDYHQRV   91
CC133_771E                     52  VYKI--------------------NTFSYEGEAILTVGDGVGV-----GKV-FHYVN----GKFDYHQRV   91
CC398-1E                       52  VYKI--------------------NTFSYEGEAILTVGDGVGV-----GKV-FHYVN----GKFDYHQRV   91
CC80-1Y                        55  NNSKNIY--ESENKLT---QEGYKKARQLPENTLLVTCIASI------GKN-AILRK-----QGSCNQQI  107
CC75-1U                        55  IGK---------------------DSNYDIEESYISILKDGAGV-----GRL-NLRPG-----KSSVIGTM   93
CC1-1F                         62  SINSTYN--DQNIRVN---KNKKTEKYILSKGDLAMVLNDKTKDGKIIGRS-IFIDK---DNQYIYNQRT  122
CC873 e*                       57  PKKETF------------KGSDNTQYYKRKAGQLMYGKLDFLN-----CAF-GIVPD---SLNNYESTID  105
CC80-2Z                        59  RLIKIYN-----------SKEFSSQKNKFNPQNVLYGKLRPYL-----NKY-YFTKK-----SGVCSSEI  106
CC80-3XS.Sau11819ORF2227P      60  LRPNS--------QTKY-FIENPPQSVIANKEDILMTRTGNT------GKV-VTNVF-----GAFHNNFF  108
CC80-1X                        60  LRPNS--------QTKY-FIENPPQSVIANKEDILMTRTGNT------GKV-VTNVF-----GAFHNNFF  108
CC75-1T                        60  NNSKVTH---SKEKITE-YAMKSLKLKLVPKNSVLIAMYGGFNQI---GRT-GLLKI-----DATINQAI  116
ST130-1T                       60  NNSKVTH---SKEKITE-YAMNSLKLKLVPKNSVLIAMYGGFNQI---GRT-GLLKI-----DATINQAI  116
CC93-3M                        57  HSKFKTV--LDSDGNIP-NIIEKAVFELIQKGDIVFADASEDYSDL--GKA-VMIDFE--PNSLISGLHT  118
CC133_771-1strain32320Hsd      57  HSKFKTV--LDSDGNIP-NIIEKAVFELIQKGDIVFADASEDYSDL--GKA-VMIDFK--PNSLISGLHT  118
CC133-2fromED133J              55  MTS--------------------VSEPLSEIDAVGIGRKGTI------NKP-YLLEA-----PFWTVDTL   92
CC72-2J                        55  MTS--------------------VSEPLSEIDAVGIGRKGTI------NKP-YLLEA-----PFWTVDTL   92
CC51TRD1J                      55  MTS--------------------VSEPLSEIDAVGIGRKGTI------NKP-YLLEA-----PFWTVDTL   92
CC30-2strainMRSA252HsdSJ       55  MTS--------------------VSEPLSEIDAVGIGRKGTI------NKP-YLLEA-----PFWTVDTL   92
CC59-1J                        55  MTS--------------------VSEPLSEIDAVGIGRKGTI------NKP-YLLEA-----PFWTVDTL   92
CC72-1R                        56  KTLNSS------KYISE-EAFEKEFKIRPEFGDILMTRIGDI------GTP-NIVSS---NEKFAYYVSL  108
CC15TRD1O                      59  HNIKREN---ITNFITE-KGLNESSAKLITNEAILIAMYGQGKTR---GMS-AILNF-----EATTNQAC  115
CC398-1strain398HsdSN          61  FDKEIS------------NIDNIKKYYVVEENDFVYNPRMSNYAPF--GPV-NRNKL---GKKGVMSPLY  112
ST425-1C                       56  FNNRSINT-NNLTGKVN-VNSKELKNYSVEKGDVFFTRTSEVIGEI--GYP-SVILND--PENTVFSGFV  118
CC30-1strainMRSA252HsdSC       56  FNNRSLNT-NNLTGKVN-VNSKELKNYSVEKGDVFFTRTSEVIGEI--GYP-SVILND--PENTVFSGFV  118
CC45-1strain3067HsdSC          56  FNNRSINT-NNLTGKVN-VNSKELKNYSVEKGDVFFTRTSEVIGEI--GYP-SVILND--PENTVFSGFV  118
CC97A                          60  RNGKLNL--NDLVYISK-DIDDEMKNSRTYYGDVLLNITGASI-----GRT-AINSIV--ETHANLNQHV  118
CC1-2strainMW2HsdSA            60  RNGKLNL--NDLVYISK-DIDDEMKNSRTYYGDVLLNITGASI-----GRT-AINSIV--ETHANLNQHV  118
CC1-1strainMW2HsdSA            60  RNGKLNL--NDLVYISK-DIDDEMKNSRTYYGDVLLNITGASI-----GRT-AINSIV--EIHANLNQHV  118
CC5-2strainN315HsdSA           60  RNGKLNL--NDLVYISK-DIDDEMKNSRTYYGDVLLNITGASI-----GRT-AINSIV--ETHANLNQHV  118
CC75-2V                        66  GDGYFYN--SNKVFTSN-EKAEVLKSCNVFPGDIVIAKMADPI-----ARA-AIVPDN-NIGKYLMASDG  125
CC22-1strain5096HsdSB          61  FSKSVS-------------SKNLENYTLIKNGEFAYNKSYSNGYPL--GAI-KRLTR---YDSGVLSSLY  111
CC51TRD2P                      56  FSKSVS-------------SKNLENYTLIKNGEFAYNKSYSNGYPL--GAI-KRLTR---YDSGVLSSLY  106
CC5-1strainN315HsdSB           61  FSKSVS-------------SKNLENYTLIKNGEFAYNKSYSNGYPL--GAI-KRLTR---YDSGVLSSLY  111
CC93-2 b*                      57  KNNKIIIS-SDDRKISESDYKKIYKNYKLEKGDLLLTIVGTI------GRA-AIVKN---PNNIAFQRSV  115
Consensus_ss:                                  e            ee     eeeeee         eee eee        eeee  e
```

```
Conservation:                                      7                                          6    98 775
CC80-3 f*                    111  IKFKINE---QNSPLFIYQFTLTSKFNKWVKVMS----VRSGQPGINSEEYAKLPLVLPN-KLEQQKIAK  172
CC45-1L                      110  VGLRVNN---NNLPSFIYYMLSYKGNQKKIKRIQ----MGAVQPSVKVSQFKFIKYLVPI-KDEQEKVAK  171
CC97 c*                      105  PAFEINT--EKIEPNYFISYISRPSFYKSKEKYS----TGTGSKRIHENTVLNFSLHLPC-LNEQLKIAS  167
CC22-1I                      116  IAFNSNE---KITDNFLASLLSSENVYNDLLKLC----SGATAKGVSQKNLNRLYVTIPHSISEQEEIAE  178
CC873D                        99  TVLYPTQ---NTSSLFIGYKFKTHRMIHKFKINSQ--GLTSDTWNLKYKQLKNINIDIPV-LEEQEKIGD  162
CC5-1D                        99  TVLYPTQ---NTSSLFIGYKFKTHRMIHKFKINSQ--GLTSDTWNLKYKQLKNINIDIPV-LEEQEKIGD  162
CC30-1D                       99  TVLYPTQ---NTSSLFIGYKFKTHRMIHKFKINSQ--GLTSDTWNLKYKQLKNINIDIPV-LEEQEKIGD  162
CC5-2H                       105  PVFKGI----DANQYYLTLHLNYQ-LKKEYIKYA----TGTSQLVLSQKDLQNIKTKLPS-YEEQQKIGD  164
CC133-2fromED133 d*          116  IRFRTNH---KIRSEFFLIFMRSNQMQRKILEAN---PGSAITNLVPVKELKLIPFPLPV-KFEQDKISQ  178
CC72-2S                      102  CSIKGD--------KFLYYFLEWFATQNKWIRFS----QGSTFESISGNDIRNIHIKIPV-EDERTKIIK  158
CC93-3 a*                    107  CHFIWKV--NKINTEFIYYYLNS---LNISTFGV----QAVKGVTLNNDSINSIIVKLPN-EEEQNIIAK  166
CC93-2K                      111  LSLSSL----KYDKLYSLYSLLY--TMKKISANL----QGTSIKGITKKELLDSIIKIPHNLEEQQKIGD  170
CC30-2K                      111  LSLSSL----KYDKLYSLYSLLY--TMKKISANL----QGTSIKGITKKELLDSIIKIPHNLEEQQKIGD  170
CC80-2W                      111  LIFLNP----KFNLFFMYYWLEY--FKDKWSKYG----QPGSQVNLNTEIVKSQTLNMPS-NHEQEKVGQ  169
CC75-2W                      111  LIFLKP----KFNLFFMYYWLEY--FKDKWSKYG----QPGSQVNLNSEIVKSQTLNMPS-NHEQEKVGQ  169
CC59Q                         98  HILSPL----NGNIQYLYQVAEL----VNYEKYN----TGTAQPKLNIQNLKIINVVISTNLEEQQKIGS  155
CC72-1Q                       98  HILSPL----NGNIQYLYQVAEL----VNYEKYN----TGTAQPKLNIQNLKIISVVISX----------  145
CC1-2G                        98  HVVKSN----DHNLFFMNYYLNF----KELRAFV----TGNAPAKLTHANLCNINLKIPC-LTEQDKVSA  154
ST425-1E                      92  YKISDFK---NYYGLLLFYYFSQ-NFLKETKKYS----AKTSVDSVRKDMVANMKVPRPI-YIEQEKIGQ  152
CC15TRD2E                     92  YKISDFK---NYYGLLLFYYFSQ-NFLKETKKYS----AKTSVDSVRKDMIANMKVPRPI-YIEQKKIGQ  152
CC133_771E                    92  YKISDFK---NYYGLLLFYYFSQ-NFLKETKKYS----AKTSVDSVRKDMVANMKVPRPI-YIEQEKIGQ  152
CC398-1E                      92  YKISDFK---NYYGLLLFYYFSQ-NFLKETKKYS----AKTSVDSVRKDMIANMKVPRPI-YIEQKKIGQ  152
CC80-1Y                      108  NAVVPFE---NINIDYLYYISDS--LSTFMKSIA----GKTATQIVNKNTFENLEIYLAP-FEEQNKIAD  167
CC75-1U                       94  GYIQSN----NVDIEFLYYRMKV----VDFKKYI----IGSTIPHLYFKDYSKETLYIPSSIQEQAKIGM  151
CC1-1F                       123  ERLIPFA---ENDNKFLWFLMNTDLIRNKIKGMM----QGATQVYINYSSIKLISIQLPL-LEEQQKIRG  184
CC873 e*                     106  SPSFDFI---NGDSKFLLERIKLKSFYKKFGDIA---NGSRKAKRINQDTFLSLPVFAPK-YDEQLRIGE  168
CC80-2Z                      107  WVLKSTKE-DKLLNLFLYYFIQTKRYS-DVASKS----AGSKMPRADWGLIENIRVYFPE-LCEQQKIGQ  169
CC80-3XS.Sau11819ORF2227P    109  KIKFDKN---LYDRLFLVEVLNSSKIQNKILSLA----GSSTIPDLNHSDFYSISSSYPL-LREQQKIGK  170
CC80-1X                      109  KIKFDKN---LYDRLFLVEVLNSSKIQNKILSLA----GSSTIPDLNHSDFYSISSSYPL-LREQQKIGK  170
CC75-1T                      117  SALLMNH---ETNPEFIQAFLNYQV-KGWKRYAA----SSRKDPNITKKDIEQFKVPYVS-INEQQKIGE  177
ST130-1T                     117  SALLMNH---ETNPEFIQAYLNYQV-KGWKRYAA----SSRKDPNITKKDIEQFKVPYVS-INEQQKIGE  177
CC93-3M                      119  HLFRPLN---NAISNFLIFYTKTLSYKKFIRQQG----TGISVLGISKKSLLNLNVLIPRSELEQQKIGQ  181
CC133_771-1strain32320Hsd    119  HLFRPLN---NAISNFLIFYTKTLSYKKFIRQQG----TGISVLGISKKSLLNLNVLIPRSELEQQKVGK  181
CC133-2fromED133J             93  FYCTPKK---ETDILFILSLFRKIN----WKVYD----ESTGVPSLSKQTINKINRFVPT-NKEQQKIGK  150
CC72-2J                       93  FYCTPKK---ETDILFILSLFRKIN----WKVYD----ESTGVPSLSKQTINKINRFVPT-NKEQQKIGK  150
CC51TRD1J                     93  FYCTPKK---ETDILFILSLFRKIN----WKVYD----ESTGVPSLSKQTINKINRFVPT-NKEQQKIGK  150
CC30-2strainMRSA252HsdSJ      93  FYCTPKK---ETDILFILSLFRKIN----WKVYD----ESTGVPSLSKQTINKINRFVPS-NKEQQKIGE  150
CC59-1J                       93  FYCTPEK---EADILFILSLFRKIN----WKLYD----ESTGVPSLSKQTINKINRLVPT-NKEQQKIGE  150
CC72-1R                      109  ALLKTK----NLNSYFLKNLILSSSIQNELWRKT---LHVAFPKKINKNEIGKIKINYPK-KQEQQKIGQ  170
CC15TRD1O                    116  AIYQTN-----QNINFVFQYFQK--LYEFLRSLS----NEGSQKNLSLSLLKEITLNYPN-EQEQKKIGD  173
CC398-1strain398HsdSN        113  TVFKIQ----NIDLNFIEFYFKSSKWYRFMALNGD-SGARADRFSIKDRTFMEMPLHIPC-MDEQIKIGQ  176
ST425-1C                     119  LRGRPKSGIDLINNNFKRYVFFTNSFRKEMITKS----SMTTRALTSGTAINKMKVIYPVSAKEQKKIGD  184
CC30-1strainMRSA252HsdSC     119  LRGRPKSGIDLINNNFKRYVFFTNSFRKEMITKS----SMTTRALTSGSAINKMKVIYPVSAKEQRKIGD  184
CC45-1strain3067HsdSC        119  LRGRPKSGIDLINNNFKRYVFFTNSFRKEMITKS----SMTTRALTSGTAINRMKVIYPVSAKEQKKIGD  184
CC97A                        119  CIIRLKK---EYYYIFFGQYLLSRKGKRKIFLAQ----SGGSREGLNFKEIANLKIFTPTIFEEQQKIGK  181
CC1-2strainMW2HsdSA          119  CIIRLKK---EYYYNFFGQYLLSRKGKRKIFLAQ----SGGSREGLNFKEIANLKIFTPTIFEEQQKIGK  181
CC1-1strainMW2HsdSA          119  CIIRLKK---EYYYNFFGQYLLSRKGKRKIFLAQ----SGGSREGLNFKEIANLKIFTPTIFEEQQKIGE  181
CC5-2strainN315HsdSA         119  CIIRLKK---EYYYNFFGQYLLSRKGKRKIFLAQ----SGGSREGLNFKEIANLKIFTPTIFEEQQKIGQ  181
CC75-2V                      126  IRLSVDT--VHFNTKFVLECINRKSFRKKVEDNS----SGSTRMRIGLSTLGSLTLKTTT-LKEQQKIGQ  188
CC22-1strain5096HsdSB        112  ICFSIKS---EMSKDFMEAYFDSTHWYREVSGIAVEGARNHGLLNVSVNDFFTILIKYPS-LEEQQKIGK  177
CC51TRD2P                    107  ICFSIKS---EMSKDFMEAYFDSTHWYREVSGIAVEGARNHGLLNISVNDFFTILIKYPS-LEEQRKIGD  172
CC5-1strainN315HsdSB         112  ICFSIKS---EMSKDFMEAYFDSTHWYREVSGIAVEGARNHGLLNVSVNDFFTILIKYPS-LEEQQKIGK  177
CC93-2 b*                    116  AILKTKA---TYDVGFIFQLFQTKYFKNLLLRKQ----VVSAQPGLYLGDIRKIKISITNIIEEQRKIGI  178
Consensus_ss:                     eeeee        hhhhhhhh  hhhhhhhhhh              hhhhh  ee     hhhhhhhh
```

```
Conservation:                        75   6  5     76  66 597 568568
CC80-3 f*                       173  FLDRFDRQIELEKQKIEILQQQKKGLLQSMFI  204
CC45-1L                         172  LLIEIDKLVNKQLIKIELLQQRKKALLKSMFI  203
CC97 c*                         168  FVCFLNRKIELLERKIYLIKKQKQALLQQMFI  199
CC22-1I                         179  FFRKINQLVELQKYKIEHTKSQKQVFLQKMFI  210
CC873D                          163  FFKKMDILISKQKIKIEILEKEKQSFLQKMFL  194
CC5-1D                          163  FFKKMDILISKQKMKIEILEKEKQSFLQKMFL  194
CC30-1D                         163  FFKKMDILISKQKIKIEILEKEKQSFLQKMFL  194
CC5-2H                          165  FFSEIDRLVEKQSSKVGRLKVRKKELLQKMFV  196
CC133-2fromED133 d*             179  FIHIINRRIEQSEKKIESLKNRKQGFLQKLFV  210
CC72-2S                         159  LLNSLDVLNSKTDLKIQNLKQRKQSLLQKIFV  190
CC93-3 a*                       167  FLLEVDKTVNNQLVKTKLLKQRKKGLLQRMFV  198
CC93-2K                         171  LFYKIDKYISFNKCKIEMLKSLKQGLLKKMFI  202
CC30-2K                         171  LFYKIDKYISFNKCKIEILKSLKQGLLQKIFI  202
CC80-2W                         170  FFNRNEKLIELQQEKIMYLKRRKQVLLQKMFI  201
CC75-2W                         170  FFNRNEKLIELQQEKIMYIKRCKQVLLQKMFI  201
CC59Q                           156  FLSKLDRQIDLEEQKLELLQQRKKALLKSMFV  187
CC72-1Q                              --------------------------------
CC1-2G                          155  LLKSIDNKMNNQMNRIELLKERKKELLQKMFI  186
ST425-1E                        153  FIKKVDNKIKIQKQVIELLKQRKKALLQKMFI  184
CC15TRD2E                       153  FIKRVDNKTKIQKQVIELLKQRKKALLQKMFI  184
CC133_771E                      153  FIKKVDNKIKIQKQVIELLKQRKKALLQKMFI  184
CC398-1E                        153  FIKRVDNKTKIQKQVIELLKQRKKSLLQKMFI  184
CC80-1Y                         168  LISSLEELIEKQASKLIKMKSRKQGMLQIMFI  199
CC75-1U                         152  FISNLDKLIENKNLKLNCLKQLKQGLLQSMFI  183
CC1-1F                          185  FLEVLSGITTKQLHKIDQLKERKKAFLQKMFI  216
CC873 e*                        169  FFSKLDRQIELQKQKLELLQQQKKGYMQKIFS  200
CC80-2Z                         170  FFSKLDRQIELEEQKLELLQQQKKGYMQKIFS  201
CC80-3XS.Sau11819ORF2227P       171  FFSKLDRQIELEEQKLELLQQQKKGYMQKIFS  202
CC80-1X                         171  FFSKLDRQIELEEQKLELLQQQKKGYMQKIFS  202
CC75-1T                         178  FFSKIDHQIELEEQKLELLQQQKKGYMQKIFS  209
ST130-1T                        178  FFSKLDRQIELEEQKLELLQQQKK--------  201
CC93-3M                         182  FFSKLDRQIELEEQKLELLQQQKKGYMQKIFS  213
CC133_771-1strain32320Hsd       182  FFSKLDRQIELEEQKIELLQQQKKGYIQKIFS  213
CC133-2fromED133J               151  FFSKLDRQIELQEQKLELLQQQKKGYMQKIFS  182
CC72-2J                         151  FFSKLDRQIELEEQKLELLQQQKKGYMQKIFS  182
CC51TRD1J                       151  FFSKLDRQIELEEQKLELFQQQKKGYMQKIFS  182
CC30-2strainMRSA252HsdSJ        151  FFIKLDRQIELEEQKLELLQQQKKGYMQKIFS  182
CC59-1J                         151  FFSKLDRQIELEEQKLELLQQQKKGYMQKIFS  182
CC72-1R                         171  FFSKLDRQIELEEQKLELLQQQKKGYMQKIFS  202
CC15TRD1O                       174  FFSKLDRQIELEEQKLELLQQQKKGYMQKIFS  205
CC398-1strain398HsdSN           177  FFSKLDRQIELEEQKLELLQQQKKGYMQKIFS  208
ST425-1C                        185  FFSKLDRQIELEEQKLELLQQQKKGYMQKIFT  216
CC30-1strainMRSA252HsdSC        185  FFSKLDRQIELEEQKLELLQQQKKGYMQKIFS  216
CC45-1strain3067HsdSC           185  FFSKLDRQIELEEQKLELLQQQKKGYMQKIFS  216
CC97A                           182  FFSKLDRQIELEEQKLELLQQQKKGYLQKIFS  213
CC1-2strainMW2HsdSA             182  FFSKLDRQIELEEQKLELLQQQKKGYMQKIFT  213
CC1-1strainMW2HsdSA             182  FISKLDRQIELEEQKLELLQQQKKGYMQKIFS  213
CC5-2strainN315HsdSA            182  FFSKLDQQIELEEQKLELLQQQKKCYIQKIFS  213
CC75-2V                         189  FFSKLDRQIVLEEQKLELLQQQKKGYMQKIFS  220
CC22-1strain5096HsdSB           178  FFSKLDRQIELEEQKLELLQQQKKGYMQKIFS  209
CC51TRD2P                       173  FFIKLDRQIELEEQKLELLQQRKKALLKSMLI  204
CC5-1strainN315HsdSB            178  FFSKLDRQIELEEQKLELLQQQKKGYMQKIFS  209
CC93-2 b*                       179  FFSKLDRQIELEEQKLELLQQQKKGYMQKIFS  210
Consensus_ss:                        hhhhhhhhhhhhhhhhhhhhhhhhhhhhhh
```
